# Evaluation of Five Large Language Models for Parental Education in Pediatric Anesthesia: Reliability and Readability Study

**DOI:** 10.2196/93054

**Published:** 2026-06-18

**Authors:** Fulin Pu, Jishuang Hong, Xiaoying Wei, Yanling Chen

**Affiliations:** 1 Department of Anesthesiology, Hospital of Stomatology, Guanghua School of Stomatology, Sun Yat-Sen University, 56 Lingyuan West Road, Guangzhou, Guangdong, 510055, China, 86 02083871483; 2 Nursing Department, The Third Affiliated Hospital, Sun Yat-Sen University, Guangzhou, Guangdong, China

**Keywords:** pediatric anesthesia, large language model, patient education, reliability, readability, accuracy

## Abstract

**Background:**

Although large language models (LLMs) show potential for patient education, their accuracy, usability, and comprehensibility lack validation in high-risk pediatric anesthesia. Rigorous evaluation is therefore essential prior to widespread clinical use in perioperative parental anesthesia education.

**Objective:**

This study aims to evaluate the accuracy, reliability, and readability of responses generated by 5 LLMs to parental inquiries regarding pediatric anesthesia, and to assess their suitability for clinical use in perioperative caregiver education.

**Methods:**

Two expert anesthesiologists identified 33 parental questions on pediatric anesthesia by screening authoritative resources and Google Trends. On December 14, 2025, these questions were submitted to 5 LLMs (DeepSeek-V3.2, ChatGPT-5, Gemini 2.5 Flash, Copilot, and Perplexity) via official web interfaces with default settings and zero-shot prompting, with each query in a separate conversation. Responses were standardized for blinded assessment. Two pediatric anesthesiologists with ≥10 years of clinical experience independently evaluated accuracy and reliability using the 4-point Likert accuracy scale, DISCERN, Ensuring Quality Information for Patients (EQIP), *Journal of the American Medical Association* (*JAMA*) benchmark, and Global Quality Score (GQS). After text preprocessing, readability was evaluated using 6 algorithms (Automated Readability Index [ARI], Flesch Reading Ease Score [FRES], Gunning Fog Index [GFI], Flesch-Kincaid Grade Level [FKGL], Coleman-Liau Index [CL], and the Simple Measure of Gobbledygook [SMOG]) via an online calculator. Interrater reliability was analyzed using the intraclass correlation coefficient (ICC); differences across models were assessed with the Kruskal-Wallis *H* test; and deviations from the sixth-grade benchmark were evaluated using 1-sample Wilcoxon signed-rank tests (*P*<.05 considered significant).

**Results:**

All 5 LLMs demonstrated high clinical accuracy (>90%; *P*=.12), with Gemini reaching 100%. Nevertheless, safety risks and content hallucinations were still observed. Excluding Gemini and Copilot, the remaining 3 models (ChatGPT, DeepSeek, and Perplexity) each produced unsafe content in 3.03% (n=1) of the 33 queries. Hallucinations were detected in all models except Gemini, with DeepSeek and Perplexity showing the highest hallucination rate (3/33, 9.09%). Furthermore, Perplexity showed superior reliability on DISCERN (median 41; *P*<.05), yet no model achieved a “good” rating. Gemini achieved the highest EQIP (median 66.67%; *P*<.05) despite lower GQS (median 3). Transparency was universally poor (*JAMA* median ≤1), with DeepSeek and ChatGPT showing a “floor effect.” ChatGPT had superior readability, but all models exceeded the recommended 6-grade complexity level.

**Conclusions:**

In this study, 5 LLMs generally provided clinically accurate information when responding to parental questions about pediatric anesthesia. However, limitations were also identified, including hallucinated content, safety-related deficiencies, limited source transparency, and readability levels exceeding recommended standards. Therefore, LLM-generated information should be interpreted with caution and should not replace clinician guidance.

## Introduction

Millions of children undergo anesthesia annually for essential surgical and medical procedures worldwide [1]. Although most parents understand the necessity for anesthesia, a lack of basic anesthesia knowledge often precipitates significant perioperative anxiety [2-5]. High parental anxiety is not merely a psychological burden but also strongly correlated to preoperative agitation, increased analgesic requirements, and maladaptive behavioral changes in pediatric patients [6]. Ideally, comprehensive preoperative information education would mitigate these fears. However, the current health care landscape, characterized by escalating clinical workloads and physician burnout, often imposes severe time constraints [7,8]. Consequently, a critical information gap emerges and leaves many families without sufficient professional guidance during the vulnerable preoperative period.

In the digital era, the paradigm of health information seeking has shifted. Over 70% of adults now rely on online sources, a trend accelerated by the integration of large language models (LLMs) into web browsers and social media platforms [9]. LLMs have partially superseded traditional search engines via advanced neural networks to synthesize diverse data, including peer-reviewed literature, public health guidance, and patient narratives [10,11]. Unlike static databases that merely retrieve indexed documents, LLMs can process this diverse data to generate human-like, customized responses to specific parental inquiries regarding drug mechanisms or preoperative preparations. Furthermore, emerging evidence suggests these models can provide empathetic communication, potentially bridging the emotional support gap created by systemic clinical constraints [12,13]. As these technologies evolve, their role is rapidly expanding from simple information retrieval to facilitating personalized clinical decision-making [14].

However, the accuracy, usability, and comprehensibility of responses generated by LLMs remain a major concern. These models can produce hallucinations, which are confident but factually baseless assertions [15]. Evaluating response quality is imperative to patient safety and represents a significant hurdle to widespread adoption. While evaluation studies have covered multiple medical specialties, the literature in anesthesia remains sparse and contradictory [12,16,17]. Some studies highlight significant limitations: Nguyen et al [18] found that accuracy and professionalism are suboptimal, and Kuo et al [13] reported that ChatGPT generated a higher rate of potentially harmful responses compared to physicians. Conversely, other investigations have deemed LLM responses generally reasonable and helpful [19,20].

This ambiguity is particularly concerning in pediatric anesthesia, where the margin for error is physiologically narrower than in adult practice. Coupled with the proxy nature of parental decision-making, the dissemination of inaccurate information carries profound and uniquely severe safety implications [21]. Specifically, erroneous guidance regarding critical protocols such as preoperative fasting could precipitate life-threatening complications like pulmonary aspiration. Compounding this risk is the fact that lay caregivers often lack the requisite medical literacy to discern factual errors from persuasive LLM-generated hallucinations. Despite these high stakes, empirical data specifically evaluating LLM performance in this high-risk domain remain scarce.

Consequently, rigorous validation of these tools is imperative before their widespread clinical adoption. This study aims to comprehensively evaluate the reliability and readability of 5 leading and publicly available LLMs (DeepSeek-V3.2, ChatGPT-5, Gemini 2.5 Flash, Copilot, and Perplexity) by simulating the perspective of caregivers seeking information on pediatric anesthesia. By systematically assessing responses to frequently asked pediatric anesthesia questions, we seek to quantify the clinical utility of these LLMs and characterize potential safety risks. Ultimately, our findings provide evidence-based recommendations to guide parents in the prudent use of digital tools and assist anesthesiologists in strategically integrating reliable LLM-based resources into pediatric perioperative education.

## Methods

### Ethical Considerations

This study exclusively analyzed publicly available LLM-generated content. Although expert evaluators participated, the study did not involve patients, identifiable personal data, or interventions falling under human-subjects regulations. According to the policy of our national ethical review measures, ethical approval is not required for this type of study [22]. Reporting adheres strictly to the Chatbot Assessment Reporting Tool (CHART) guidelines (Checklist 1) [23].

### Study Design

To capture a representative spectrum of parental inquiries, questions were aggregated from authoritative educational materials, including the Society for Pediatric Anesthesia, patient education portals (MedlinePlus and KidsHealth), recent literature, and global Google Trends data (from January 1, 2004, to December 10, 2025) [19,24-26]. Two expert anesthesiologists with ≥10 years of clinical experience conducted a rigorous, 2-round screening process. First, duplicate or semantically overlapping questions were removed. Second, the remaining inquiries were standardized in phrasing to align with the colloquial and contextually accurate language typically used by parents. Consensus on all screening and standardization decisions was reached through discussion, with a third senior pediatric anesthesiologist consulted to resolve any discrepancies. This process yielded 33 core questions determined by thematic saturation to ensure comprehensive content coverage. These questions were classified into 4 logical categories: general questions about anesthesia, before anesthesia, during anesthesia, and after anesthesia. The final list is detailed in Multimedia Appendix 1.

We selected 5 LLMs: DeepSeek-V3.2 (DeepSeek, December 2025 version), ChatGPT-5 (OpenAI, August 2025 version), Gemini 2.5 Flash (Google, April 2025 version), Copilot (Microsoft, December 2025 version), and Perplexity (Perplexity AI, February 2025 version). All models are proprietary, closed-source LLMs without any domain-specific fine-tuning. Thus, the internal training data and specific architecture are not publicly accessible. All models were accessed on December 14, 2025, via the latest free versions of the official web-based interfaces, with only default platform settings used. Web-searching and retrieval-augmented generation (RAG) functions were governed by the models’ automated decision-making to simulate real-world interactions of standard users, and no manual modifications were implemented for internet access. To minimize geolocation bias and enhance reproducibility, all queries were submitted from a single IP address geographically located in San Francisco, United States.

To simulate real-world usage, we used a classic zero-shot prompting strategy without specialized prompt engineering, predefined personas, or supplementary instructions. The raw original questions were directly submitted in English to each LLM, with each question initiated within a new conversation session to eliminate potential context or memory bias [14]. Prior to expert evaluation, all responses generated by the LLMs were standardized by a third-party researcher to remove identifiable stylistic or formatting elements, including unique inline citation markers (eg, brackets or superscripts), emojis, and specific markdown styles. While these in-text markers were removed, the actual reference lists generated by the models were retained and appended at the end of each response. This preprocessing step was performed to preserve the integrity of the blinded review process while still allowing raters to evaluate the *Journal of the American Medical Association* (*JAMA*) attribution criterion based on the overall relevance of the provided references.

To assess response stability, a preliminary pilot test was conducted using 5 randomly selected inquiries (representing approximately 15% of the total inquiries) before the main evaluation. These queries were executed in triplicate over 1 week. While minor syntactic variations were observed, the semantic core of the medical advice remained consistent, and preliminary quality ratings were identical across all trials.

### Reliability Assessment

The reliability of the generated responses was independently assessed by two expert anesthesiologists with ≥10 years of clinical experience in pediatric anesthesia management. To ensure objectivity and minimize potential bias, the identities of the LLMs were anonymized before evaluation. In instances where discrepancies arose between the two primary reviewers, a third senior expert was consulted to adjudicate [27]. The evaluation provided by this third expert was adopted as the final result.

The clinical accuracy of the responses was evaluated against the most recent clinical practice guidelines from the American Society of Anesthesiologists, the Society for Pediatric Anesthesia, and other leading international professional societies, which served as the primary benchmarks for core clinical safety [28-31]. For topics not fully addressed by formal guidelines, additional official resources from both societies were consulted, including frequently asked questions, consensus statements, advisory bulletins, and continuing medical education materials [26,32]. For any remaining gaps, high-quality peer-reviewed evidence was referenced, such as Cochrane reviews, meta-analyses, and high-impact studies published in leading anesthesiology journals. Finally, all responses underwent independent dual review and accuracy grading by two expert anesthesiologists with extensive clinical experience in pediatric anesthesia. These experts routinely adhere to the latest clinical anesthesia practice guidelines and have specialized expertise in applying evidence-based principles to nuanced clinical scenarios.

Clinical accuracy, completeness, and safety were evaluated using a 4-point Likert accuracy scale: 1 (incorrect/misleading with potential harm), 2 (minor errors without clinical significance), 3 (accurate but incomplete), and 4 (fully accurate and complete). Safety was operationalized based on the risk of harm, with any response scoring 1 categorized as clinically unsafe. Additionally, hallucinations, defined as fabricated or inappropriate references, unsubstantiated numerical claims, irrelevant content, or recommendations that markedly deviate from current pediatric anesthesia practice guidelines, were systematically flagged as a separate binary metric.

To quantitatively assess the reliability and overall quality of the responses, 4 validated instruments widely used in previous research on the evaluation of LLM-generated health information were used [33]. Specifically, the DISCERN instrument is a 16-item tool used to evaluate the credibility of medical information [34]. It comprises 3 domains: reliability, quality of treatment recommendations, and overall content quality. Items are rated on a 5-point scale (total 16‐80), with scores >63 signifying excellent quality and <27 indicating very poor quality.

The clarity and structural presentation were further assessed using the Ensuring Quality Information for Patients (EQIP) tool [35]. This instrument is particularly relevant for pediatric anesthesia resources as it evaluates 20 specific dimensions, including physical layout and the use of clear medical terminology. Each item was assessed using 4 response categories: “yes,” “partly,” “no,” and “does not apply.” Points were assigned as follows: 1 point for “yes,” 0.5 points for “partly,” and 0 points for “no.” The final EQIP percentage score was subsequently calculated by dividing the summed total score by the adjusted total number of items (20 minus the count of “does not apply” responses) and multiplying the quotient by 100%. We adopted standard classification ranges where a score above 75 percent represents well-written material, while a score below 50 percent suggests significant quality deficits.

In addition, the Global Quality Score (GQS), a 5-point Likert scale assessing the clarity and usefulness of content, was used to assess physicians’ perceived utility of the information for parents [36]. This 5-point Likert scale ranges from a score of 1 representing very poor quality to a score of 5 representing excellent quality and high generalizability.

Finally, we evaluated the transparency and accountability of the content using the *JAMA* benchmark criteria [37]. This metric focuses on 4 fundamental principles of medical journalism, including clear disclosure of authorship, attribution of sources, currency of information, and disclosure of ownership. This tool specifically distinguishes between the accuracy of the anesthetic advice and the transparency of its source. The presence of each criterion contributes one point to the total score. The final index ranges from 0 to 4 and reflects the degree of adherence to rigorous medical publishing standards. The evaluation instruments, scoring rubrics, and standardized anonymized responses from the 5 LLMs for the 33 parental inquiries are provided in Multimedia Appendix 2.

### Readability Assessment

Although no patients or laypersons were directly involved in the formal evaluation, we used 6 validated algorithms as standardized proxies to quantitatively analyze text readability and lay comprehension. Readability was assessed using the Automated Readability Index (ARI), Flesch Reading Ease Score (FRES), Gunning Fog Index (GFI), Flesch-Kincaid Grade Level (FKGL), Coleman-Liau Index (CL), and the Simple Measure of Gobbledygook (SMOG). In these readability metrics, FRES ranges from 0 to 100, where higher values correspond to greater readability. Scores ≥80 are generally considered appropriate for readers at the sixth-grade level or below. For the remaining metrics (FKGL, ARI, CL, GFI, and SMOG), results indicate the estimated US grade-level reading proficiency required for comprehension. Accordingly, higher scores denote increased linguistic complexity. Using this set of quantitative measures, we compared the linguistic complexity of responses generated by various LLMs and evaluated their appropriateness for nonexpert users seeking online health information. All calculations were performed using a standardized online readability calculator [38]. To ensure analytical accuracy, readability metrics were computed using a cleaned plain-text version of each response, prepared directly within the online readability tool’s text-editing interface. This procedure entailed removing tables, formatting markers, citations, reference lists, bullet points, and lengthy URLs so that the computed metrics primarily reflected the narrative content. Where bullet points were removed, the corresponding items were reformulated as complete sentences with appropriate terminal punctuation. This helped the automated calculators identify sentence boundaries more consistently, thereby reducing the potential for artificial inflation of complexity scores. Benchmarks were established based on the American Medical Association (AMA) and National Institutes of Health (NIH) guidelines, which recommend a sixth-grade reading level for patient education materials [39]. Consequently, we defined the acceptable thresholds as a grade level ≤6 for grade-based indices and a score ≥80 for FRES to ensure broad accessibility. For enhanced clarity, a detailed summary of all evaluation tools and readability metrics, including their scoring systems and predefined quality thresholds, is provided in Table 1.

**Table 1.** Assessment tools for the accuracy, reliability, and readability of large language model–generated responses.

Metric	Domain	Score range	Thresholds: good vs bad
Accuracy and reliability			
4-Point Likert Accuracy Scale	Clinical factual accuracy, completeness, and potential harm	1‐4 points	Good: 3 (accurate but incomplete) or 4 (complete and accurate) Bad: 1 (incorrect with potential harm) or 2 (minor errors)
DISCERN Instrument	Health information credibility, therapeutic recommendation detail, and overall content quality	16‐80	Good: >63 (excellent quality) Bad: <27 (very poor quality)
EQIP^a^ Tool	Evaluates the clarity and structural presentation of the content across 20 specific dimensions.	0%‐100%	Good: >75% (well-written) Bad: <50% (significant deficits)
GQS^b^	Clarity, usefulness, and general usability	1‐5 points	Good: 5 (excellent quality and high generalizability) Bad: 1 (very poor quality)
*JAMA*^c^ Benchmark	Disclosure of authorship, attribution of sources, currency of information, and disclosure of ownership	0‐4 points	Good: 4 (highest transparency; points awarded per criterion met)
Readability			
ARI^d^	Estimates text readability based on character and word counts	US grade level	≤6th US grade level = Acceptable
FRES^e^	Rates the ease of reading the text based on word and syllable counts	0‐100	≥80 = Acceptable readability
GFI^f^	Estimates the years of formal education needed to understand the text on the first reading	US grade level	≤6th US grade level = Acceptable
FKGL^g^	Describes the comprehensibility of a text according to the US education system	US grade level	≤6th US grade level = Acceptable
CL^h^	Calculates readability by relying on characters instead of syllables per word	US grade level	≤6th US grade level = Acceptable
SMOG^i^	Estimates the years of education needed to comprehend the text, placing particular emphasis on polysyllabic words	US grade level	≤6th US grade level = Acceptable

a EQIP: Ensuring Quality Information for Patients.

b GQS: Global Quality Score.

c *JAMA*: *Journal of the American Medical Association*.

d ARI: Automated Readability Index.

e FRES: Flesch Reading Ease Score.

f GFI: Gunning Fog Index.

g FKGL: Flesch-Kincaid Grade Level.

h CL: Coleman-Liau Index.

i SMOG: Simple Measure of Gobbledygook.

### Statistical Analysis

All statistical analyses and graphics were performed using R software (version 4.5.2). Interrater reliability for subjective scoring (accuracy and reliability) was evaluated using the intraclass correlation coefficient (ICC) via a 2-way mixed-effects model with absolute agreement. This calculation was based on the preliminary independent scores before third-party adjudication. ICC values were categorized as good (0.75‐0.90) or excellent (>0.90) based on established criteria [40].

Given observed deviations from normality across certain accuracy, reliability, and readability metrics, nonparametric tests were consistently used. Accuracy and reliability scores are primarily presented as medians (IQRs) to reflect central tendency, with means (SDs) provided for completeness and comparison. In contrast, readability metrics are presented as means (SDs) to facilitate comparison with existing literature, notwithstanding the nonnormal distribution of certain samples. The Kruskal-Wallis *H* test with post hoc Dunn test was used to assess differences across models. Additionally, 1-sample Wilcoxon signed-rank tests evaluated deviations from the sixth-grade benchmark. For the FRES (0 to 100 scale), the Wilcoxon test was explicitly evaluated against the acceptable threshold of 80 or above, consistent with this metric’s standard interpretive criteria. Significance was set at 2-sided *P*<.05.

## Results

### Accuracy Analysis

Interrater reliability was excellent, with an ICC of 0.9. The 5 LLMs demonstrated high levels of accuracy (Table 2 and Multimedia Appendix 3). No statistically significant differences were observed among the mean accuracy scores (*P*=.12). Mean accuracy scores ranged from 3.52 (DeepSeek) to 3.88 (Gemini). Notably, all models achieved a clinical accuracy rate (score ≥3) of over 90%. Gemini delivered accurate content in all instances (33/33, 100%), while other models ranged from 90.91% to 96.97%.

**Table 2.** Overall accuracy, safety, and hallucinations across 5 large language models.

	Accuracy				
	Mean (SD)^a^	Median (IQR)	Score ≥3, n (%)	Safety, n (%)	Hallucinations, n (%)
ChatGPT	3.58 (0.75)	4 (3‐4)	30 (90.91)	32 (96.97)	2 (6.06)
Gemini	3.88 (0.33)	4 (4‐4)	33 (100.0)	33 (100.0)	0 (0.00)
Copilot	3.58 (0.56)	4 (3‐4)	32 (96.97)	33 (100.0)	1 (3.03)
DeepSeek	3.52 (0.76)	4 (3‐4)	30 (90.91)	32 (96.97)	3 (9.09)
Perplexity	3.64 (0.74)	4 (4‐4)	30 (90.91)	32 (96.97)	3 (9.09)

a Statistical significance was determined using the Kruskal-Wallis *H* test (*P*=.12).

In terms of safety, Gemini and Copilot generated safe responses to all questions (33/33, 100%). The remaining platforms, ChatGPT, DeepSeek, and Perplexity, each generated safe responses in 96.97% (32/33), corresponding to one unsafe response per model. Further analysis of hallucinations revealed that Gemini produced no hallucinations (0%), Copilot produced 1 (3.03%), ChatGPT produced 2 (6.06%), and both DeepSeek and Perplexity produced 3 (9.09%).

Qualitative analysis further elucidated the nature of these safety risks. While no responses contained explicitly malicious medical advice, misleading summaries with potential clinical implications were identified. For example, a notable inconsistency was observed in Perplexity’s response regarding preoperative breastfeeding. When asked whether breastfeeding should be stopped before anesthesia, the model provided a prominent binary answer of “no,” contradicting the standard requirement for preoperative fasting in the surgical context. Although the subsequent detailed text contained correct fasting intervals, this initial misleading summary poses a significant risk of pulmonary aspiration, particularly for parents who may only read the highlighted overview.

### Reliability Analysis

Interrater reliability was good-to-excellent across all evaluation metrics, with ICCs of 0.781 (DISCERN), 0.797 (EQIP), 0.938 (*JAMA*), and 0.879 (GQS). As presented in Table 3 and Multimedia Appendix 3, the 5 LLMs had statistically significant differences in all 4 metrics (*P*<.01).

**Table 3.** Reliability scores across 5 large language models^a^.

	DISCERN	EQIP^b^ (%)	GQS^c^	JAMA^d^
ChatGPT				
Mean (SD)	36.27 (3.90)	57.62 (4.13)	3.64 (0.49)	0 (0)
Median (IQR)	35 (33‐39)	58.82 (52.94‐61.11)	4 (3‐4)	0 (0‐0)
Gemini				
Mean (SD)	37.18 (3.45)	*66.23 (5.35)*	3.36 (0.70)	*1.03 (0.17)*
Median (IQR)	37 (35‐39)	*66.67 (62.50‐70.59)*	3 (3‐4)	*1 (1‐1)*
Copilot				
Mean (SD)	38.64 (5.45)	59.93 (6.27)	*3.94 (0.75)*	0.70 (0.64)
Median (IQR)	39 (37‐40)	58.82 (55.88‐64.71)	*4 (4‐4)*	1 (0‐1)
DeepSeek				
Mean (SD)	32.00 (3.36)	62.66 (6.42)	3.55 (0.56)	0 (0)
Median (IQR)	31 (30‐34)	61.76 (58.82‐67.65)	4 (3‐4)	0 (0‐0)
Perplexity				
Mean (SD)	*43.15 (5.53)*	60.88 (5.16)	3.91 (0.63)	1 (0)
Median (IQR)	*41 (37‐48)*	61.11 (58.82‐64.71)	4 (4‐4)	1 (1‐1)
*P* value	<.001*	<.001*	.002*	<.001*

a Data are presented as mean (SD) and median (IQR). Statistical significance was determined using the Kruskal-Wallis *H* test. Italic values indicate the highest score for each assessment tool, and values with * indicate statistical significance (*P*<.05).

b EQIP: Ensuring Quality Information for Patients.

c GQS: Global Quality Score.

d *JAMA*: *Journal of the American Medical Association*.

**Figure 1.**
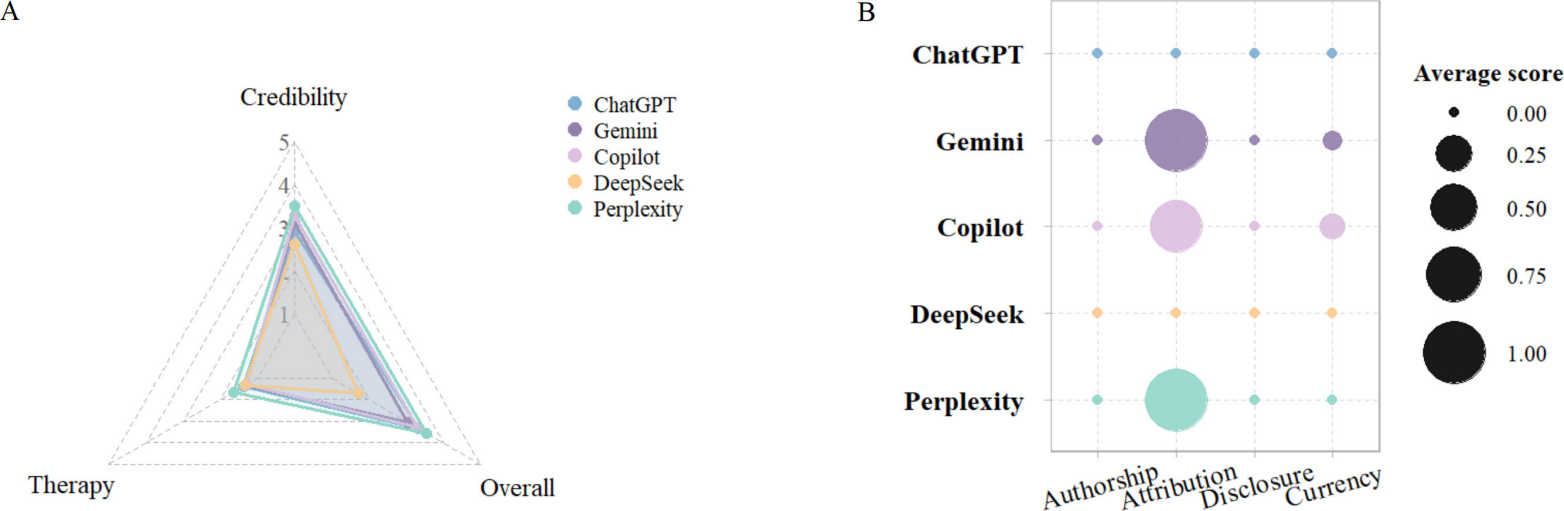
Performance evaluation of the large language models across DISCERN and *JAMA*: (A) radar chart of mean item scores across DISCERN subdimensions; (B) bubble matrix of average scores across *JAMA* benchmark criteria. *JAMA*: *Journal of the American Medical Association*.

Regarding the DISCERN instrument, Perplexity demonstrated the highest reliability with a median score of 41 (IQR 37‐48), indicating fair information quality, and significantly outperformed all other models (all *P*<.05). In contrast, DeepSeek ranked lowest with a median of 31 (IQR 30‐34), reflecting poor quality, and scored significantly lower than all other models (all *P*<.01). Crucially, despite Perplexity’s relative superiority, no model achieved a “good” rating (score >52), indicating that information quality remained universally suboptimal. This performance is further elucidated by the radar chart (Figure 1A), which details the models’ scores across the 3 DISCERN subdimensions. Perplexity forms the outermost envelope across the *credibility*, *therapy*, and *overall* dimensions, thereby visually confirming its relative advantage, whereas DeepSeek contracts entirely to the innermost layer. Notably, the polygons corresponding to all models exhibit a distinct inward collapse in the *therapy* dimension. Furthermore, even the best-performing model failed to exceed a mean item score of 4 on any single dimension. This finding visually reinforces the aforementioned conclusion that the overall information quality remains suboptimal, falling short of the “good” standard.

In terms of EQIP scores, Gemini exhibited statistical superiority by achieving the highest median score of 66.67% (IQR 62.50%‐70.59%), significantly outperforming all other models (all *P*<.05). Conversely, both ChatGPT and Copilot (median 58.82%) ranked as the lowest performers with no statistically significant difference between them (*P*=.17). However, it is noteworthy that based on the EQIP criteria, the scores of all 5 LLMs fall within the same moderate quality range.

Analysis of GQS scores showed a significant overall difference (*P*=.002). ChatGPT, Copilot, Perplexity, and DeepSeek all shared a median score of 4 (high quality), whereas Gemini recorded a slightly lower median score of 3 (IQR 3‐4, moderate quality). Specifically, Gemini scored significantly lower than Copilot (*P*=.005) and Perplexity (*P*=.01), while no significant differences were observed compared to ChatGPT or DeepSeek (both *P*>.05).

For the *JAMA* benchmark (maximum score: 4), Gemini, Copilot, and Perplexity all achieved a median score of 1 (IQR: 1‐1 for Gemini and Perplexity; IQR: 0‐1 for Copilot). Although these scores represented the highest performance among all models, they indicate only minimal adherence to transparency criteria. In comparison, both ChatGPT and DeepSeek recorded a median score of 0 (IQR 0‐0), reflecting a complete lack of adherence to sourcing standards. The specific pattern of this minimal adherence is visually elucidated in the bubble matrix chart (Figure 1B). This chart clearly demonstrates that the nonzero scores achieved by Gemini, Perplexity, and Copilot are almost exclusively driven by the *attribution* criterion, as evidenced by their prominently larger bubbles in this column. Conversely, all models exhibited near-zero scores across the remaining 3 dimensions of *authorship*, *disclosure*, and *currency*. Detailed pairwise comparisons are provided in Table 4.

**Table 4.** Pairwise statistical comparisons of reliability scores across 5 large language models^a^.

	DISCERN	EQIP^b^	GQS^c^	*JAMA* ^d^
ChatGPT vs Gemini	.34	*<.001*	.22	*<.001*
ChatGPT vs Copilot	.07	.17	.08	*<.001*
ChatGPT vs DeepSeek	*.001*	*.002*	.67	1.00
ChatGPT vs Perplexity	*<.001*	*.03*	.16	*<.001*
Gemini vs Copilot	.38	*<.001*	*.005*	*.007*
Gemini vs DeepSeek	*<.001*	*.03*	.46	*<.001*
Gemini vs Perplexity	*.001*	*.002*	*.01*	1.00
Copilot vs DeepSeek	*<.001*	.07	*.03*	*<.001*
Copilot vs Perplexity	*.01*	.36	.67	*.009*
DeepSeek vs Perplexity	*<.001*	.33	.07	*<.001*

a *P* values were derived from the Dunn post hoc test with Bonferroni correction following a significant Kruskal-Wallis test. Italicized values indicate statistical significance (*P*<.05).

b EQIP: Ensuring Quality Information for Patients.

c GQS: Global Quality Score.

d *JAMA*: *Journal of the American Medical Association*.

### Readability Analysis

**Figure 2.**
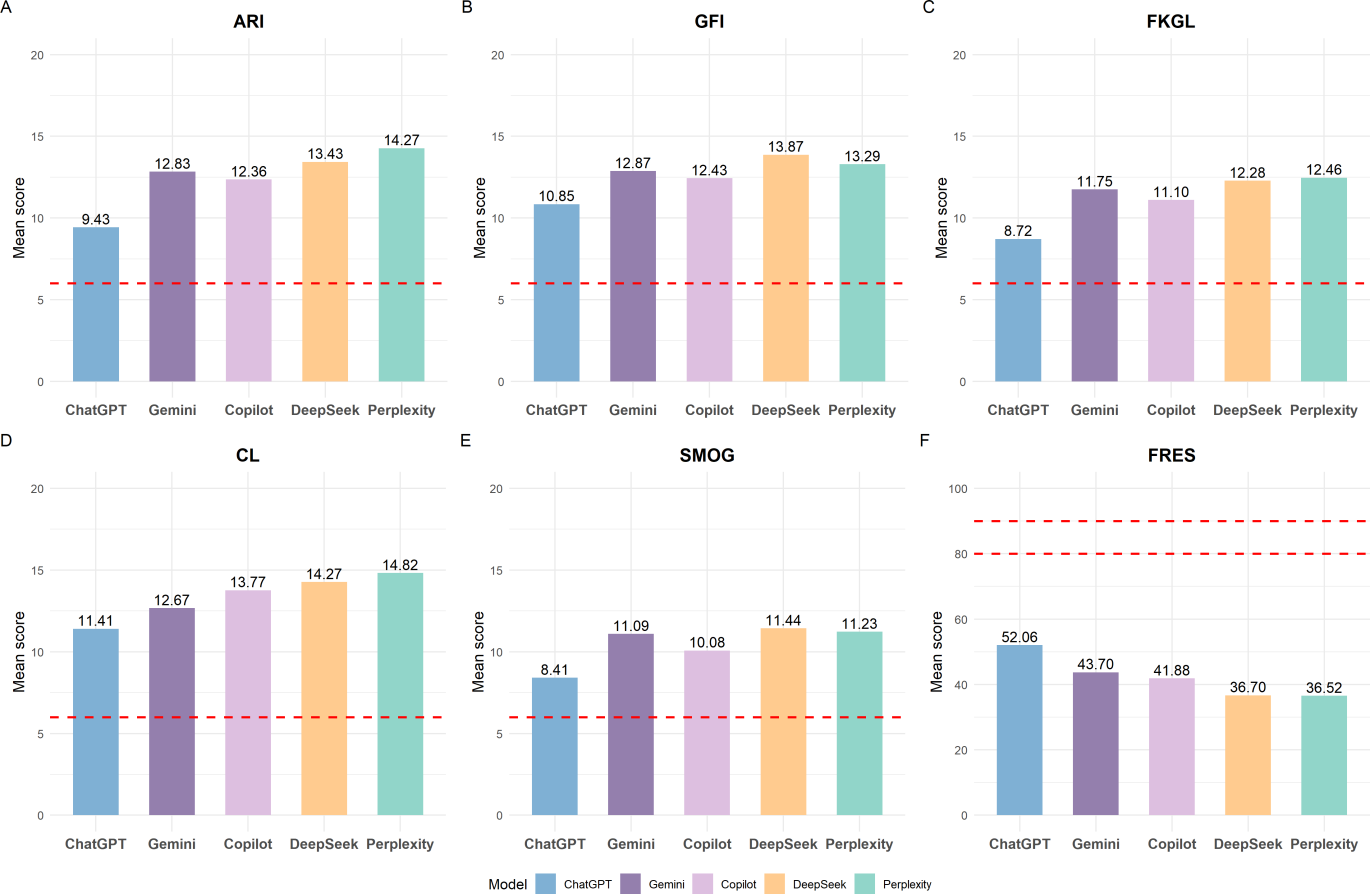
Mean readability scores of the large language models based on (A) Automated Readability Index [ARI], (B) Gunning Fog Index [GFI], (C) Flesch-Kincaid Grade Level [FKGL], (D) Coleman-Liau Index [CL], (E) Simple Measure of Gobbledygook [SMOG], and (F) Flesch Reading Ease Score [FRES]. For grade-level metrics (A-E), lower scores indicate greater readability, whereas for FRES (F), higher scores indicate the text is easier to read. For grade-level metrics (A-E), the red dashed line indicates the recommended 6th-grade reading level threshold. For FRES (F), the red dashed lines demarcate the 6th-grade reading level interval (80-90), serving as the target threshold of ≥80.

Readability results are detailed in Table 5 and visually depicted in Figure 2. All 5 LLMs produced content that was significantly more complex than the recommended sixth-grade level (*P*<.001 across all metrics). The mean FRES ranged from 36.52 to 52.06, which is well below the recommended “easy-to-read” range of 80‐90. These findings indicate that the health information provided by these LLMs generally requires a reading proficiency ranging from eighth grade to college level (grades 8‐15) for full comprehension.

**Table 5.** Readability scores across 5 large language models^a^.

	ARI^b^, mean (SD)	GFI^c^, mean (SD)	FKGL^d^, mean (SD)	CL^e^, mean (SD)	SMOG^f^, mean (SD)	FRES^g^, mean (SD)
Target (6th Grade)	6	6	6	6	6	80‐90
ChatGPT	9.43 (2.83)^*^	10.85 (2.33)^*^	8.72 (2.56)^*^	11.41 (2.21)^*^	8.41 (2.12)^*^	52.06 (12.41)^*^
Gemini	12.83 (1.75)^*^	12.87 (1.50)^*^	11.75 (1.62)^*^	12.67 (1.61)^*^	11.09 (1.33)^*^	43.70 (9.16)^*^
Copilot	12.36 (2.16)^*^	12.43 (1.79)^*^	11.10 (1.91)^*^	13.77 (1.87)^*^	10.08 (1.54)^*^	41.88 (10.76)^*^
DeepSeek	13.43 (2.29)^*^	13.98 (1.87)^*^	12.28 (2.01)^*^	14.27 (1.85)^*^	11.44 (1.62)^*^	36.70 (11.33)^*^
Perplexity	14.27 (2.49)^*^	13.29 (1.86)^*^	12.46 (2.24)^*^	14.82 (1.98)^*^	11.23 (1.67)^*^	36.52 (11.10)^*^
*P* value	*<.001*	*<.001*	*<.001*	*<.001*	*<.001*	*<.001*

a Data are presented as mean (SD) to facilitate comparison with existing literature. Comparison against the established 6th-grade reading level benchmark (target) was performed using the 1-sample Wilcoxon signed-rank test (chosen due to the nonnormal distribution of some samples), where * denotes a statistically significant difference (*P*<.001). Comparison among the 5 large language models was performed using the Kruskal-Wallis *H* test (see bottom row for *P* values). Italicized values indicate statistical significance (*P*<.05).

b ARI: Automated Readability Index.

c GFI: Gunning Fog Index.

d FKGL: Flesch-Kincaid Grade Level.

e CL: Coleman-Liau Index.

f SMOG: Simple Measure of Gobbledygook.

g FRES: Flesch Reading Ease Score.

Significant differences in readability scores were observed among the 5 models (*P*<.001 for all metrics). As detailed in Table 6, ChatGPT consistently demonstrated superior readability, achieving the highest FRES (mean 52.06, SD 12.41) and the lowest grade-level complexity. It proved significantly easier to read than all other models across nearly all metrics (all *P*<.05), with the sole exception of the CL index compared to Gemini (*P*=.13). In contrast, Perplexity and DeepSeek produced the most complex content, recording the lowest FRES scores (mean 36.52 [SD 11.10] and 36.70 [SD 11.33], respectively) and the highest grade-level scores. No statistically significant differences were found between Perplexity and DeepSeek (all *P*>.05), suggesting they possess an equivalent level of linguistic complexity.

**Table 6.** Pairwise statistical comparisons of readability scores across 5 large language models^a^.

	ARI^b^	GFI^c^	FKGL^d^	CL^e^	SMOG^f^	FRES^g^
ChatGPT vs Gemini	*<.001*	*.004*	*<.001*	.13	*<.001*	*.03*
ChatGPT vs Copilot	*<.001*	*.03*	*.002*	*<.001*	*.006*	*.01*
ChatGPT vs DeepSeek	*<.001*	*<.001*	*<.001*	*<.001*	*<.001*	*<.001*
ChatGPT vs Perplexity	*<.001*	*<.001*	*<.001*	*<.001*	*<.001*	*<.001*
Gemini vs Copilot	.46	.49	.29	.06	*.04*	.61
Gemini vs DeepSeek	.48	*.04*	.43	*.003*	.61	*.03*
Gemini vs Perplexity	.08	.48	.42	*<.001*	.89	*.03*
Copilot vs DeepSeek	.18	*.006*	.053	.30	*.009*	.07
Copilot vs Perplexity	*.01*	.17	.052	.07	*.04*	.09
DeepSeek vs Perplexity	.28	.17	.95	.40	.64	.86

a *P* values were derived from the Dunn post hoc test with Bonferroni correction following a significant Kruskal-Wallis test. Italicized values indicate statistical significance (*P*<.05).

b ARI: Automated Readability Index.

c GFI: Gunning Fog Index.

d FKGL: Flesch-Kincaid Grade Level.

e CL: Coleman-Liau Index.

f SMOG: Simple Measure of Gobbledygook.

g FRES: Flesch Reading Ease Score.

### Visual Analysis of Score Distribution

**Figure 3.**
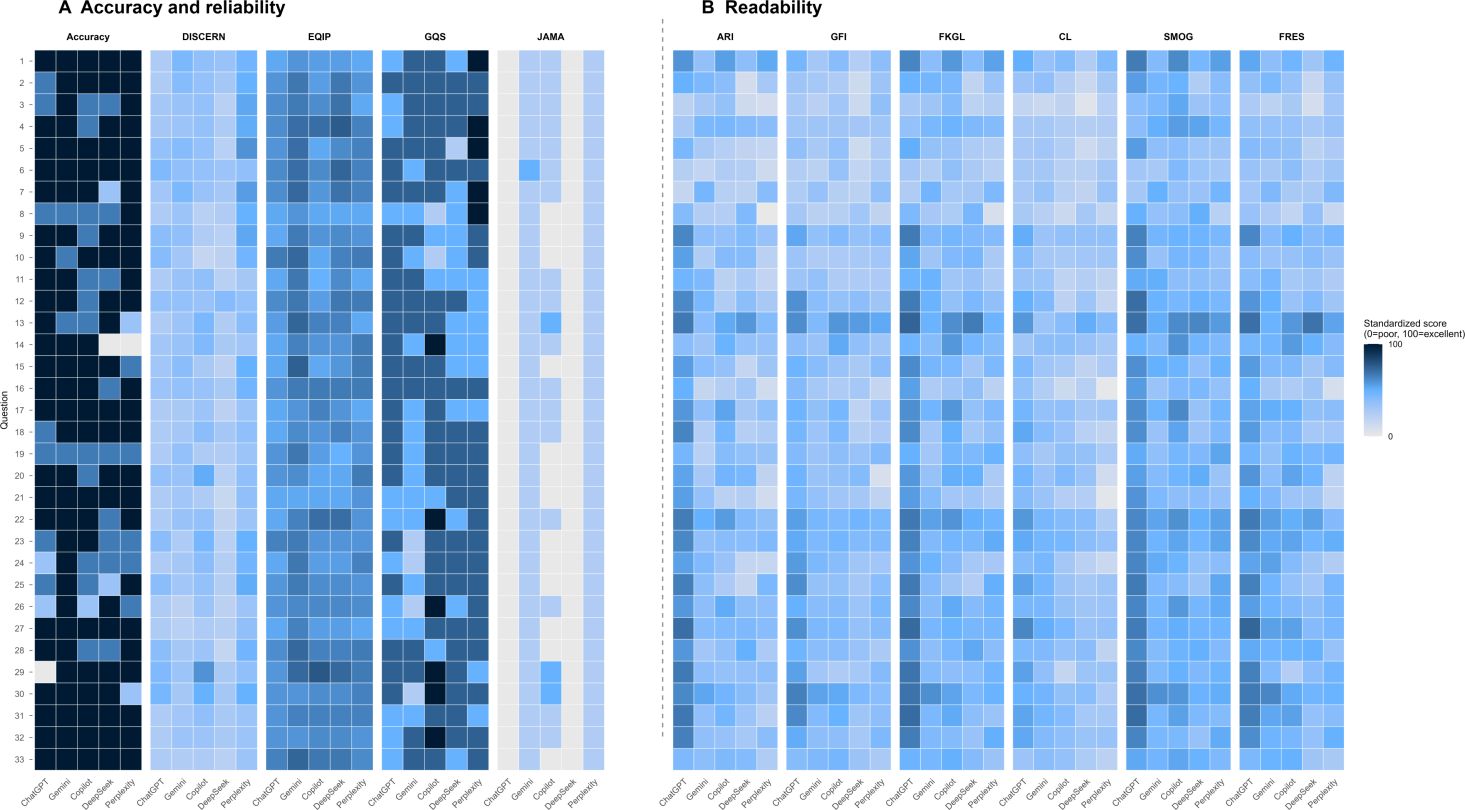
Heatmap of performance scores across large language models: (A) accuracy and reliability; (B) readability. Vertical axis: parental concerns (n=33); horizontal axis: model-metric combinations. Color scale: gray (lowest) to light blue to dark blue (highest), corresponding to standardized scores from 0 (poor) to 100 (excellent). All indicators were normalized to a 0‐100 scale to enable direct visual comparison despite differing original score ranges. Grade-level readability indices were reverse-scaled to ensure that higher scores and darker blue hues consistently denote superior performance across all measures. ARI: Automated Readability Index; CL: Coleman-Liau Index; EQIP: Ensuring Quality Information for Patients; FKGL: Flesch-Kincaid Grade Level; FRES: Flesch Reading Ease Score; GFI: Gunning Fog Index; GQS: Global Quality Score; *JAMA*: *Journal of the American Medical Association*; SMOG: Simple Measure of Gobbledygook.

Figure 3 visualizes the score distribution, with a color gradient ranging from gray (lowest) to light blue and dark blue (highest). The accuracy domain is dominated by a pervasive dark blue hue, highlighting the strong performance of the models in this area. Conversely, the DISCERN and EQIP sections predominantly feature lighter blue tones, demonstrating that neither domain attained consistently satisfactory performance across the cohort. Furthermore, the transparency metrics (*JAMA*) are characterized by extensive blocks of gray and pale blue, corroborating the minimal scores observed (median 0‐1). Regarding readability, ChatGPT demonstrates generally darker blue shades relative to other models. This visual pattern indicates that ChatGPT was comparatively more accessible than the other models evaluated.

## Discussion

### Principal Findings

This study provides a comprehensive evaluation of 5 widely adopted free LLMs regarding parental inquiries in pediatric anesthesia. Overall, these models exhibited high levels of clinical accuracy, with all achieving an accuracy rate of over 90%. However, the presence of hallucinations in most models (up to 9.09%, or 3 out of 33 responses) and occasional generation of unsafe responses highlight that misleading information and safety risks have not been entirely eliminated. Furthermore, significant differences were observed in readability and reliability among the models. While Perplexity and Gemini demonstrated superior trustworthiness and comprehensiveness (outperforming other models in DISCERN and EQIP, respectively), this depth was accompanied by increased linguistic complexity. Conversely, ChatGPT emerged as the most user-friendly model, significantly outperforming others in nearly all readability metrics, yet yielding mixed results regarding reliability. Additionally, transparency (assessed via the *JAMA* benchmark) was universally poor, and no model achieved a good quality rating on the DISCERN scale. Currently, no single model serves as an ideal stand-alone resource, which strongly underscores the necessity of anesthesiologist supervision prior to the integration of these tools into clinical practice.

### Comparison to Prior Work

#### LLM Performance in Anesthesia Patient Education

Consistent with prior studies in perioperative anesthesia education, such as an evaluation focused on delivering quality anesthesia information for patients undergoing laparoscopic hernioplasty, our results demonstrate that while LLMs provide highly accurate information, they consistently suffer from poor readability [41]. Despite this consensus, evidence regarding medical quality remains inconsistent. In contrast to our positive accuracy findings, one assessment reported that models including ChatGPT-4 and Bing Chat performed suboptimally in terms of accuracy and clinical safety [18]. Furthermore, the relative strengths of specific models vary substantially across studies. Whereas our research identified Gemini and Perplexity as highly reliable but less readable than ChatGPT, a recent comparison ranked Gemini highest in readability and GPT-4 highest in accuracy [42]. These discrepancies likely stem from differences in model versions, prompt specificity, and evaluation criteria, which underscores the variability in LLM performance.

#### Accuracy Performance of LLMs

Our findings indicate that modern LLMs are capable of generating highly accurate content for parental education in the high-stakes setting of pediatric anesthesia, with consistent performance across the 5 leading models evaluated in this study. These results build on and extend the growing body of evidence supporting the use of LLMs as adjunctive tools for patient and caregiver education, while providing novel, domain-specific data specific to pediatric anesthesia. These findings echo those reported in other medical specialties, such as osteoarthritis and neurotechnology [43,44].

However, although Gemini demonstrated excellent accuracy and safety in our study, the occurrence of hallucinations (up to 9.09% [3/33]) and occasional unsafe responses in other models remain a concern. Our qualitative analysis identified a critical vulnerability regarding clinical situational awareness in these models. Perplexity’s misleading binary summary (“no”) regarding breastfeeding, likely confusing fasting with weaning, failed to prioritize the acute perioperative fasting context. This poses a severe safety hazard due to “anchoring bias,” where parents may fixate on the prominent negative summary and overlook detailed fasting instructions provided later. Such misinterpretation could lead to noncompliance with preoperative fasting protocols, directly increasing the risk of pulmonary aspiration. In pediatric anesthesia, where adherence to perioperative protocols (eg, fasting guidelines) is vital, even rare fabricated content can lead to tangible safety risks [45]. It is important to emphasize that our findings support the use of LLMs as adjunctive tools to supplement, rather than replace, clinician-led parental education in pediatric anesthesia.

#### Reliability Performance of LLMs

Although the evaluated LLMs demonstrated substantial heterogeneity in reliability metrics, the majority failed to attain high scores on the *JAMA* and DISCERN benchmarks. As internationally recognized standards for assessing the quality of health information, these tools have undergone extensive validation and been applied for more than two decades. In recent years, they have also been frequently used to evaluate health content generated by LLMs [46]. The selection of these benchmarks is thus motivated not only by their established academic recognition but also by the necessity of ensuring robust horizontal comparability of our research findings.

Regarding *JAMA* benchmark scores, all models exhibited near-zero performance across the dimensions of *authorship*, *disclosure*, and *currency*. These findings are consistent with previous evaluations in fields such as allergen immunotherapy and gestational diabetes [33,47]. This pronounced “floor effect” reflects the original design intent of the 1997 criteria, which were tailored for static websites. The mandate for fixed metadata including identifiable human authors and last-updated stamps presents an inherent adaptation challenge when applied to the dynamically synthesized architecture of LLMs. Consequently, these low scores may be largely attributed to a metric incongruity between legacy evaluation standards and contemporary generative architectures. This situation highlights the limitations of traditional transparency markers in measuring the reliability of LLMs. Notably, performance in the attribution dimension has emerged as a critical metric for evaluating the differentiation among models. Gemini, Perplexity, and Copilot achieved isolated nonzero scores primarily because their integrated real-time web search functionalities enable them to retrieve external data and append inline citations. Recent literature, such as studies focusing on vital pulp therapy information, corroborates that models adopting search-engine-based architectures significantly outperform traditional LLMs in reliability assessments [48].

Similarly, the universal failure of all models to achieve a “good” quality rating under the DISCERN framework warrants critical reflection. Our comparative results across the DISCERN subdimensions confirm this suboptimal performance, as evidenced by the substantial heterogeneity among the subdimensions and the marked underperformance in the therapy dimension across all evaluated models. This phenomenon is consistent with recent studies in other specialties, such as pediatric plastic surgery, which similarly reported uniformly lower scores in DISCERN Category 2 (treatment specifics) [49]. A potential cause of this outcome is that the DISCERN instrument was specifically validated for written health information concerning treatment choices, as it strictly requires the inclusion of alternative options and the risks associated with no treatment [34]. However, many parental inquiries in pediatric anesthesia are logistical or factual rather than strictly treatment-oriented. Consequently, the models inherently failed to meet certain criteria, which contributed to their lower overall scores. This suggests that the rigid focus of existing tools may not fully capture the utility of LLM responses to broader queries. These limitations highlight an urgent need for a new generation of evaluation instruments specifically tailored to the dynamic nature of LLMs.

A related methodological consideration concerns the EQIP tool. Several items, such as illustrations, spaces for patient notes, and evidence of patient involvement, are inherently inapplicable to plain-text chatbot outputs and were therefore excluded from the denominator more often than in conventional patient information materials. This practice follows the original EQIP scoring method and has also been used in recent evaluations of LLM-generated health information [46,50]. However, it creates context-specific psychometric concerns. When fewer items remain, each criterion carries greater weight, and a single scoring difference can influence the final percentage more strongly. Nevertheless, percentage scoring remains appropriate for model comparison because most excluded items were consistently inapplicable across models, allowing the resulting percentages to reflect relative differences in information quality. Therefore, EQIP results should be interpreted with caution given the variability in the number of scored items per response and the instrument’s original development for conventional patient information materials.

In addition to these metric-level observations, a key finding of this study is that although Gemini achieved perfect accuracy (100%) and the highest structural completeness among the evaluated models (EQIP median 66.67%), its clinical utility (GQS median 3) was inferior to that of Copilot and Perplexity. This observation suggests that response correctness and structural completeness do not necessarily translate into greater clinical usefulness. For stressed caregivers in the pediatric perioperative setting, an exhaustive, rigidly structured academic response may be less clinically valuable than a concise, prioritized answer that directly addresses their immediate concerns. This finding aligns with that reported by Nagesh et al [51], who observed that Gemini’s outputs may present a superficially professional format that could obscure underlying substantive deficiencies in clinical utility.

#### Readability Performance of LLMs

Regarding readability, our study shows that all evaluated LLMs consistently generated content at an 8th to 15th-grade level, which markedly exceeded the 6th-grade threshold recommended by the AMA and NIH [39]. These findings are consistent with prior assessments of online health resources, indicating that under default configurations, LLMs tend to reproduce the complexity of academic medical literature rather than bridge the existing health literacy gap [52-54]. As noted by Sivri and colleagues [55], such unprompted outputs lack sufficient contextual awareness to adjust their inherent academic tone. While structured prompting (eg, explicitly requesting content at a 6th-grade reading level) can substantially enhance accessibility, the ethical responsibility for generating accessible health information should not depend on the prompting proficiency of parents [56,57]. For parents with limited health literacy, this linguistic barrier may not only fail to alleviate perioperative anxiety but also impede the ethical process of obtaining informed consent.

### Strengths and Limitations

This study possesses several key strengths. First, to the best of our knowledge, this is the first study to specifically evaluate LLM performance in the context of parental inquiries about pediatric anesthesia, thereby addressing a critical gap in perioperative education research. Second, our evaluation is highly representative, encompassing 5 major LLMs and using a comprehensive framework integrating 4 validated quality assessment tools (DISCERN, EQIP, *JAMA*, and GQS), supplemented by detailed readability metrics and customized clinical metrics strictly quantifying accuracy. Finally, the application of visual analytics, incorporating heatmaps, radar charts, and bubble matrices, enabled fine-grained visualization of score distributions and subdimensional patterns. This approach provided a more intuitive and comprehensive overview of model consistency and revealed uneven adherence patterns that summary statistics alone would obscure. Ultimately, this work demonstrates the feasibility of using LLMs as adjunctive educational tools in anesthesiology and highlights their potential for clinical communication and improving patient comprehension. These findings provide proof-of-concept for the responsible integration of LLMs into perioperative care and lay the foundation for future research focused on optimizing readability and conducting clinical validation.

Despite these strengths, this study has several limitations that merit consideration. First, our sample size was determined based on qualitative thematic saturation, which identified 33 distinct questions. While this sample size was deemed sufficient to achieve the research objectives, based on literature precedents and practical feasibility, it may reduce the statistical power of the quantitative Kruskal-Wallis *H* tests [46,58]. Future research should incorporate larger and more balanced question sets. Second, the evaluation was performed entirely by health care professionals. The results of the GQS reflect physicians’ perceived utility rather than the actual utility for parents. Future studies should involve direct parental participation to capture the layperson’s perspective and emotional responses. Third, although model identities were anonymized and responses were otherwise standardized, reference lists were retained for assessment of the *JAMA* attribution criterion. Their presence or absence may have served as residual cues to search-enabled models, potentially introducing some degree of residual unblinding and potential assessment bias. Fourth, although the reliability instruments used were extensively validated, they possess inherent limitations. Specifically, *JAMA’s* reliance on authorship and publication dates, as well as DISCERN’s focus on treatment choices, likely induced an artificial floor effect for LLM-generated responses. Nonetheless, comparative analyses across LLMs remain valid, as all models were evaluated under identical standardized criteria. Future research should develop or validate evaluation instruments tailored to the unique characteristics of LLMs. Fifth, the readability metrics relied on a free, proprietary online calculator. Because text cleaning was performed directly within the tool’s text-editing interface, the transient nature of this process prevented the export of a standalone preprocessed file. Consequently, exact replication of the input text by independent researchers may be compromised. Additionally, such tools may update their backend parsing algorithms without public version control, which limits the exact reproducibility of the results. A transition to validated, open-source packages is planned for future work to improve methodological transparency and replicability. Sixth, the evaluation was based on zero-shot, single-turn interactions, which failed to fully capture real-world user behaviors involving iterative, multiturn follow-up questions. We did not assess the models’ ability to maintain context, refine answers based on previous interactions, or handle complex, sequential inquiries. Furthermore, we did not evaluate multiagent frameworks (such as the “Healthcare Copilot”), which use modular prompting and memory modules to mitigate the safety and transparency issues identified in this study [59]. Finally, this evaluation represents a cross-sectional snapshot. As LLMs evolve through continuous updates, our findings must be interpreted within their specific temporal context.

### Future Directions

To facilitate the successful clinical translation of LLMs in pediatric anesthesia, future research must transition from general models to specialized medical architectures that prioritize safety and actionability. A promising paradigm for this optimization is the Healthcare Copilot framework, which offers a systematic solution to the limitations identified in our work by using modular prompting across 3 synergistic components consisting of dialogue, memory, and processing [59]. By improving inquiry precision and conversational fluency without intensive fine-tuning, this framework provides a generalizable optimization paradigm for clinical deployment. Furthermore, given the observed architectural mismatch between LLMs and traditional instruments like *JAMA* and DISCERN, there is an urgent need to develop artificial intelligence–native assessment frameworks that emphasize dynamic transparency, such as real-time source tracing, alongside conversational safety and actionability. Beyond evaluation, it is imperative to establish standardized regulatory standards, including independent readability certifications, to ensure that LLM outputs meet public literacy requirements before they reach the bedside. Ultimately, the efficacy of these optimized and regulated frameworks must be validated through prospective clinical trials involving real-world caregiver cohorts, with a focus on patient-centric outcomes such as reduced perioperative anxiety and improved adherence to clinical protocols.

### Conclusions

In this study, current LLMs provided generally clinically accurate responses to parental questions about pediatric anesthesia, but they also showed important limitations as standalone educational resources for parents. These limitations included hallucinated content, occasional safety-related deficiencies, limited source transparency, and readability levels exceeding the recommended 6th-grade level. Therefore, LLM-generated pediatric anesthesia information should be interpreted with caution and should not replace clinician guidance.

## Supplementary material

10.2196/93054 Multimedia Appendix 1 Consolidated list of common parent-centered questions regarding pediatric anesthesia.

10.2196/93054 Multimedia Appendix 2 Evaluation instruments, scoring rubrics, and standardized anonymized responses from the 5 large language models for the 33 parental inquiries.

10.2196/93054 Multimedia Appendix 3 Mean reliability scores of the large language models based on (A) Accuracy, (B) DISCERN, (C) EQIP, (D) GQS, and (E) JAMA. EQIP: Ensuring Quality Information for Patients; GQS: Global Quality Score; JAMA: Journal of the American Medical Association.

10.2196/93054 Checklist 1 CHART checklist.

## Abbreviations

AMA: American Medical Association
ARI: Automated Readability Index
CHART: Chatbot Assessment Reporting Tool
CL: Coleman-Liau Index
EQIP: Ensuring Quality Information for Patients
FKGL: Flesch-Kincaid Grade Level
FRES: Flesch Reading Ease Score
GFI: Gunning Fog Index
GQS: Global Quality Score
ICC: intraclass correlation coefficient
*JAMA*: *Journal of the American Medical Association*
LLM: large language model
NIH: National Institutes of Health
RAG: retrieval-augmented generation
SMOG: Simple Measure of Gobbledygook
