# Supplementary material for: Evaluation of Five Large Language Models for Parental Education in Pediatric Anesthesia: Reliability and Readability Study
Source: JMIR Med Inform. 2026 Jun 18;14:e93054. doi: 10.2196/93054 (PMC13278617; doi:10.2196/93054)
Supplement: Multimedia Appendix 1 [file medinform-v14-e93054-s001.docx]

**[Multimedia Appendix](https://medinform.jmir.org/2026/1/e68182#app1) 1**. Consolidated list of common parent-centered questions regarding pediatric anesthesia

| **Category** | **Question** |
| --- | --- |
| **General Questions about**  **Anesthesia** | 1. What are the different types of anesthesia? |
|  | 2. What is general anesthesia? |
|  | 3. How is anesthesia different for children compared to adults? |
|  | 4. How is general anesthesia different from sedation? |
|  | 5. Is general anesthesia safe for my child? |
|  | 6. Can anesthesia harm my child’s brain development? |
|  | 7. Is general anesthesia necessary for my child's dental treatment? |
|  | 8. Who will give my child anesthesia? |
|  | 9. What if my child won't cooperate for anesthesia? |
|  | 10. What if my child is allergic to anesthesia? |
|  | 11. Will my child need any evaluation before anesthesia? |
|  | 12. Can my child have anesthesia with a cold? |
| **Before Anesthesia** | 13. When does my child need to stop eating or drinking before anesthesia? |
|  | 14. If my child is breastfeeding, do we need to stop before their anesthesia? |
|  | 15. What should I do if my child takes daily medicines before anesthesia? |
|  | 16. If my child has asthma, diabetes, or other health issues, do I need to do anything special before anesthesia? |
|  | 17. When should my child and I arrive at the hospital on surgery day for anesthesia? |
|  | 18. Can my child visit the surgery and recovery areas before anesthesia? |
| **During Anesthesia** | 19. Will my child be awake or know what’s happening during anesthesia? |
|  | 20. Will my child feel any pain while under anesthesia? |
|  | 21. Will someone watch my child the whole time during anesthesia to keep them safe? |
|  | 22. Can I stay with my child during the anesthesia process? |
|  | 23. How the anesthesia will be given to my child? |
|  | 24. Will my child need help breathing during anesthesia? |
|  | 25. Will my child be sedated before getting anesthesia? |
| **After Anesthesia** | 26. How soon will my child wake up after anesthesia? |
|  | 27. When can I see my child after anesthesia and surgery? |
|  | 28. Will my child have pain after anesthesia? |
|  | 29. Will my child feel sick or throw up after anesthesia? |
|  | 30. If my child had spinal or epidural anesthesia, could they get a headache afterward? |
|  | 31. How soon can my child eat and drink after waking up from general anesthesia? |
|  | 32. How long will my child need to stay at the hospital after anesthesia? |
|  | 33. Who can I contact if I have more questions after my child’s anesthesia? |
