# Supplementary material for: Evaluation of Five Large Language Models for Parental Education in Pediatric Anesthesia: Reliability and Readability Study
Source: JMIR Med Inform. 2026 Jun 18;14:e93054. doi: 10.2196/93054 (PMC13278617; doi:10.2196/93054)
Supplement: Multimedia Appendix 2 [file medinform-v14-e93054-s002.pdf]

Multimedia Appendix 2. Evaluation instruments, scoring rubrics, and standardized anonymized responses from the five large language models for the 33 parental inquiries.

Quality and Reliability Assessment Criteria for Pediatric Anesthesia Information

Part I: Accuracy Evaluation Scale

Instructions for Evaluators: Please assess the accuracy of each response generated by the LLMs using the following 4-point scale. Focus on factual correctness, the potential for clinical harm, and the completeness of the information provided.

Item features	Points
Factually incorrect or misleading content, which may cause harm.	1
Contains minor errors with no clinical significance, and is unlikely to cause harm.	2
Accurate but incomplete, requiring additional information to be fully useful.	3
Complete and accurate, with no need for additional information to be fully useful for its intended purpose.	4

Systematic Flagging of Hallucinations

In addition to the numerical score, evaluators are required to flag any instances of the following:

- Fabricated Content: The model generates "facts," data, or sources that do not exist or are irrelevant to the topic.
- Guideline Deviation: The response suggests actions or information that contradict current ASA (American Society of Anesthesiologists) or SPA (Society for Pediatric Anesthesia) guidelines.

Total Accuracy Score (Maximum 4): _____

Hallucinations: ☐ yes ☐ no

Part II: The Journal of the American Medical Association (JAMA) benchmark criteria.

Point component		Yes	No
**Authorship**	Author and contributor credentials and their affiliations should be provided.	☐	☐

**Attribution**	Clearly lists all copyright information and states references and sources for content.	☐	☐
**Currency**	Initial date of posted content and subsequent updates to content should be provided.	☐	☐
**Disclosure**	Conflicts of interest, funding, sponsorship, advertising, support, and video ownership should be fully disclosed.	☐	☐

The criteria for each aspect were scored separately. One point was awarded for each "yes" criterion, with a total score of 4 points.

Total JAMA Score (Maximum 4): _____

Part III: Global Quality Score (GQS)

**Item features**	**Points**
Poor quality; poor flow of information; most information is missing; not at all useful for parents/caregivers.	1
Generally poor quality; some information is listed, but many important topics are missing; of very limited use to parents/caregivers.	2
Moderate quality; some important information is adequately discussed; somewhat useful for parents/caregivers.	3
Good quality; good information flow; most relevant information is covered, although some topics are not covered; useful for parents/caregivers.	4
Excellent quality; excellent information flow; very useful for parents/caregivers.	5

Final GQS Rating: _____

Part IV: Ensuring Quality Information for Patients (EQIP)

Instructions for Evaluators

Note: Before using EQIP, expert clinicians must ensure that all information being evaluated is based on research evidence and is accurate. For each question, please select the most appropriate response: **Yes**, **Partly**, **No**, or **Does Not Apply**.

	**Question**	**Yes**	**Partly**	**No**	**Does Not Apply**
**Q1**	**Does the document start by telling you what it will cover and then cover what it says?**	☐	☐	☐	☐

	Question	Yes	Partly	No	Does Not Apply
Q2	**Does it use everyday language, explaining unusual or medical words, abbreviations, or jargon?**	☐	☐	☐	☐
Q3	**Does it use short sentences of less than 15 words on average?**	☐	☐	☐	☐
Q4	**Does it personally address the reader?**	☐	☐	☐	☐
Q5	**Is the tone respectful?**	☐	☐	☐	☐
Q6	**Is the design and layout of the document satisfactory?**	☐	☐	☐	☐
Q7	**Does the document contain easy-to-understand, relevant illustrations, diagrams, or photos?**	☐	☐	☐	☐
Q8	**Is the information presented in a logical order?**	☐	☐	☐	☐
Q9	**Does the document have a named space for the reader to make notes?**	☐	☐	☐	☐
Q10	**Does the document contain contact details for healthcare services for follow-up care/treatment?**	☐	☐	☐	☐
Q11	**Does the document contain the date it was produced?**	☐	☐	☐	☐
Q12	**Does the document contain the name of the person or department that produced it?**	☐	☐	☐	☐
Q13	**Does the document say whether patients and/or their families were involved/consulted in its production?**	☐	☐	☐	☐

	Question	Yes	Partly	No	Does Not Apply
Q14	Does it use generic names for medications/products instead of (or in addition to) brand names and designate them as such?	☐	☐	☐	☐
Q15	Does the document address quality of life issues (e.g., school attendance, mobility)?	☐	☐	☐	☐
Q16	Does it contain details of other sources of information (e.g., support groups, websites)?	☐	☐	☐	☐
Q17	Is the purpose described?	☐	☐	☐	☐
Q18	Are the benefits described?	☐	☐	☐	☐
Q19	Are risks and side-effects described?	☐	☐	☐	☐
Q20	Are any alternatives described?	☐	☐	☐	☐

Part V: DISCERN Evaluation Tool

Instrument: DISCERN (An instrument for judging the quality of written consumer health information on treatment choices). **Rating Scale:** All questions are rated on a 5-point scale: **1 (No)**, **2–4 (Partially)**, and **5 (Yes)**.

Section 1: Is the Publication Reliable?

Question	Rating (1-5)	Hints for Evaluators
1. Are the aims clear?	[ ]	Look for a clear indication at the beginning of what it is about, what it covers (and excludes), and who it is for. Note: If 'No', skip to Q3.
2. Does it achieve its aims?	[ ]	Consider if the publication provides the information it aimed to as outlined in Question 1.

Question	Rating (1-5)	Hints for Evaluators
3. Is it relevant?	[ ]	Consider if it addresses questions readers might ask and if treatment recommendations are realistic/appropriate.
4. Is it clear what sources were used?	[ ]	Check for references/evidence for main claims and a bibliography or list of expert/organisational sources. Score 5 only if both are present.
5. Is it clear when the information was produced?	[ ]	Look for dates of main sources, revision dates, and copyright date. Score 5 only if source dates are found.
6. Is it balanced and unbiased?	[ ]	Look for objective tone, range of sources, and external assessment. Be wary of single-case evidence or emotive/alarmist language.
7. Does it provide details of additional sources?	[ ]	Look for suggestions for further reading or details of other organizations for advice.
8. Does it refer to areas of uncertainty?	[ ]	Look for discussion of gaps in knowledge or differences in expert opinion. Be wary of claims of 100% success.

Section 2: Quality of Information on Treatment Choices

Note: These questions apply to the treatments (including self-care) described in the publication.

Question	Rating (1-5)	Hints for Evaluators
9. Does it describe how each treatment works?	[ ]	Look for a description of how a treatment acts on the body to achieve its effect.
10. Does it describe the benefits?	[ ]	Includes controlling symptoms, preventing recurrence, or eliminating the condition (short and long-term).

**11. Does it describe the risks?**	**[ ]**	**Includes side effects, complications, and adverse reactions (short and long-term).**
**12. Does it describe what happens if no treatment is used?**	**[ ]**	**Look for risks/benefits of postponing treatment, watchful waiting, or permanently forgoing treatment.**
**13. Does it describe effects on quality of life?**	**[ ]**	**Look for effects on day-to-day activity and relationships with family, friends, and carers.**
**14. Is it clear there may be more than one choice?**	**[ ]**	**Look for who benefits most from each choice and suggestions of alternatives to consider.**
**15. Does it support shared decision-making?**	**[ ]**	**Look for suggestions of things to discuss with family, friends, or health professionals.**

Section 3: Overall Rating

16. Overall quality rating of the publication as a source of information about treatment choices:

- **Low (1–2):** Serious or extensive shortcomings.
- **Moderate (3–4):** Potentially important but not serious shortcomings.
- **High (5):** Minimal shortcomings.
  Rating [ ]

Questions and large language model responses assessed in the study

Model 1

Q1. What are the different types of anesthesia?

Answer:
The main types of anesthesia include:

1. **General Anesthesia**
   Induces a state of unconsciousness, loss of sensation, and often requires assisted breathing. Administered via intravenous drugs or inhaled gases. Used for major surgeries.
2. **Regional Anesthesia**
   - **Neuraxial anesthesia**: Includes **spinal anesthesia** (single injection into cerebrospinal fluid) and **epidural anesthesia** (catheter placement in the epidural space). Commonly used for lower abdomen, pelvic, or lower limb surgeries (e.g., cesarean section).
   - **Peripheral nerve blocks**: Anesthetic is injected near specific nerves to numb an entire limb or region (e.g., arm, leg).
3. **Local Anesthesia**
   Medication is applied or injected directly at the surgical site to numb a small area. The patient remains awake. Used for minor procedures such as skin biopsies or suturing.
4. **Monitored Anesthesia Care (MAC)**
   Combines local anesthesia with sedation and analgesia. The patient remains breathing spontaneously while vital signs are closely monitored. Often used for shorter or less invasive procedures like endoscopies.

The choice of anesthesia depends on factors such as the type of surgery, the patient's overall health, and the anesthesiologist's assessment.

Q2. What is general anesthesia？

Answer:
General anesthesia is a medically induced, reversible state characterized by the loss of consciousness, pain sensation (analgesia), memory (amnesia), and often muscle relaxation. It is used to enable surgical or other painful medical procedures to be performed without causing the patient distress, pain, or awareness.

Key Components (The "Triad of Anesthesia"):

1. **Hypnosis (Unconsciousness):** The patient is placed in a controlled, deep state of "sleep" or complete lack of awareness.
2. **Analgesia (Pain Relief):** The body's perception of and response to painful stimuli is completely blocked.

3. **Muscle Relaxation (when needed):** Medications are often used to relax skeletal muscles, which facilitates surgery, particularly for abdominal or thoracic procedures.

How It's Administered:

- **Induction:** Anesthesia is typically initiated either by intravenous drugs (injected into a vein) or by inhaling anesthetic gases through a mask.
- **Maintenance:** The anesthetized state is maintained throughout the procedure using a combination of inhaled gases and/or intravenous medications, delivered via specialized machines.
- **Monitoring & Safety:** An **anesthesiologist** or nurse anesthetist continuously monitors the patient's vital signs (heart rate, blood pressure, oxygen levels, breathing, EKG, etc.) and adjusts the anesthetic agents accordingly.
- **Recovery:** After the procedure, the administration of anesthetic drugs is stopped. The patient gradually wakes up as the medications are metabolized and eliminated from the body.

Safety:
Modern general anesthesia is very safe due to advanced drugs, standardized protocols, and sophisticated monitoring equipment. However, as a major medical intervention, it carries some risks (e.g., allergic reactions, breathing or heart complications, postoperative nausea), which are carefully managed by the anesthesia team through preoperative evaluation and vigilant care during and after surgery.

It is a cornerstone of modern surgery, allowing for complex and lengthy operations to be performed humanely and safely.

Q3. How is anesthesia different for children compared to adults?

Answer:
Anesthesia for children differs significantly from that for adults due to variations in physiology, psychology, pharmacology, and technical approach. The key distinctions are:

1. **Physiological Differences**: Children's organ systems are still developing. Their liver and kidney functions are immature, which affects the metabolism and elimination of anesthetic drugs. Additionally, pediatric airways are narrower and more prone to obstruction, and respiratory systems are more fragile. They also have a higher metabolic rate and are more susceptible to heat loss (hypothermia).
2. **Drug Dosing & Selection**: Dosing is meticulously calculated based on weight or body surface area rather than standard adult protocols. Children are often more sensitive to certain drugs (e.g., opioids, which can cause pronounced respiratory depression). Inhalational agents (like sevoflurane) are frequently used for induction, as establishing intravenous access can be challenging in distressed young children.
3. **Psychological Care**: Children often experience high levels of preoperative anxiety due to unfamiliar environments and separation from parents. Preoperative sedation (e.g., oral midazolam) and non-pharmacological techniques (play therapy, parental presence during induction) are commonly

employed. For adults, preoperative preparation relies more on verbal explanation and reassurance.

4. **Techniques & Monitoring**: Induction is often performed via inhalation (mask) to avoid the distress of needle sticks. Specialized, smaller equipment is required for airway management and monitoring. Vigilant monitoring of temperature is essential to prevent hypothermia, and fluid management must be more precise.
5. **Risks & Complications**: Pediatric anesthesia carries specific risks, including laryngospasm, bradycardia, and post-operative apnea (especially in ex-preterm infants). Anesthesiologists require specialized pediatric training to manage these acute events. In adults, concerns shift more toward cardiovascular events, pre-existing conditions, and postoperative nausea/vomiting.
6. **Recovery & Emergence**: Children are more prone to emergence delirium (post-anesthetic agitation, confusion, or crying), requiring a calm environment and often the rapid presence of a parent. Pain management must be effective but carefully titrated. In adults, recovery focuses more on pain control, nausea/vomiting, and cognitive function.

In summary, pediatric anesthesia is not merely a scaled-down version of adult anesthesia. It demands a specialized, nuanced approach that accounts for developmental physiology, precise pharmacology, and age-appropriate psychological support, all delivered by specially trained anesthesiologists in a child-friendly setting to ensure safety and comfort.

Q4.How is general anesthesia different from sedation?

Answer:
Core Differences Between General Anesthesia and Sedation

General anesthesia and sedation are both forms of anesthesia, but they differ fundamentally in **depth, goals, and clinical application**. Simply put, sedation is a "deep sleep from which you can be aroused," while general anesthesia is a "state of temporary, controlled, and reversible unconsciousness or coma."

Here is a comparative breakdown of the key distinctions:

Feature	**General Anesthesia**	**Sedation (Typically "Conscious Sedation" or Monitored Anesthesia Care)**
**Level of Consciousness**	**Complete loss.** The patient is fully unconscious with no memory of the procedure.	**Depressed to varying degrees.** Ranges from relaxed but awake (minimal) to deeply asleep but responsive to stimulation (moderate/deep). Often results in partial or no memory.

**Pain Relief**	**Complete blockade.** The central nervous system is profoundly suppressed, eliminating all pain perception.	**Incomplete.** Patients may still feel some discomfort; sedation is often combined with local anesthesia for pain control.
**Breathing**	**Usually lost or severely impaired.** Requires **airway control** (endotracheal tube or laryngeal mask) and mechanical ventilation.	**Usually maintained.** The patient breathes spontaneously. Advanced airway support is typically not needed (except in deep sedation).
**Protective Reflexes**	**All reflexes are absent** (e.g., coughing, swallowing). The airway is unprotected and must be fully managed.	**Partially preserved.** Gag and cough reflexes are often diminished but usually present, especially at lighter levels.
**Muscle Movement**	**Muscle paralysis** is typical. The patient cannot move.	**Movement is possible.** The patient may be able to follow simple commands (e.g., "turn your head").
**Recovery Time**	Longer. Requires monitoring in a Post-Anesthesia Care Unit (PACU); full recovery is slower.	Shorter. Patients often become alert quickly and can be discharged sooner after the procedure.
**Primary Applications**	Major surgery (e.g., open abdominal, thoracic, brain surgery) – lengthy, invasive procedures requiring immobility.	Minor procedures, endoscopies (colonoscopy), dental work, imaging studies, wound repair.
**Medical Supervision**	**Mandatory management by an anesthesiologist** who continuously monitors vital signs and manages airway, breathing, and circulation.	May be administered by an anesthesiologist or other trained physician/nurse. Monitoring is less intensive for mild sedation but approaches general anesthesia levels for deep sedation.

A Simple Analogy:

- **Sedation:** Like being in a very deep, intoxicated sleep. You are largely unaware but can be stirred or awakened if needed.
- **General Anesthesia:** Like a computer being shut down. All systems (awareness, memory, pain, movement) are temporarily "turned off." Life functions (like breathing) are supported by machines, and you are "rebooted" after the procedure.

Summary:
General Anesthesia induces a **controlled, reversible state of coma** to allow for pain-free, motionless surgery.
Sedation induces a **state of depression and relaxation** to reduce anxiety, discomfort,

and awareness while often preserving the patient's ability to breathe independently and respond to commands.

The choice between them is determined by the **type of procedure, the patient's health, and the clinical requirements**, with the anesthesia provider selecting the safest and most appropriate plan.

Q5.Is general anesthesia safe for my child?

Answer:

General anesthesia for children is generally considered safe when administered by a specialized medical team. Modern anesthesia techniques are highly advanced. An anesthesiologist will carefully calculate the medication dosage based on your child's age, weight, health status, and the type of surgery, while continuously monitoring vital signs (such as heart rate, breathing, blood pressure, and blood oxygen levels) throughout the procedure.

Before surgery, the medical team will conduct a thorough assessment of your child's medical history and perform any necessary examinations to develop a personalized anesthesia plan.

While potential risks exist (such as allergic reactions to medication or temporary breathing suppression), the incidence of serious complications is extremely low. Parents can proactively communicate with the anesthesiologist, providing complete information about the child's health (including recent colds, allergy history, and medication history). Following preoperative instructions, such as fasting, is crucial and helps further ensure safety.

Please be assured that the medical team always prioritizes the child's safety above all else. If you have specific concerns, it is recommended to discuss them in detail with the anesthesiology department at the treating hospital.

Q6.Can anesthesia harm my child’s brain development?

Answer:

This is an extremely important and widely concerned question. Based on current scientific evidence, the answer is: **For the vast majority of children, a single, short general anesthetic is safe. However, there are potential risks under certain circumstances that require weighing the benefits and risks.**

Here is a detailed explanation to help you understand:

Key Takeaways (Based on extensive recent research)

1. **Single, Short Exposure is Very Low Risk**: The prevailing view is that for **healthy children over the age of 3**, a single, short-duration general

anesthetic (e.g., less than 3 hours) **has no detectable long-term negative effects on intelligence, behavior, or academic performance**. The benefits of anesthesia (enabling necessary surgery) far outweigh the potential risks.

2. **Potential Risk Factors to Consider**: Research suggests potential concerns may be associated with:
    - **Multiple Exposures**: **Multiple** or **prolonged** (cumulatively over 3 hours) general anesthetics during infancy and early childhood (particularly under 3 years old).
    - **Critical Developmental Windows**: Periods of rapid brain development (late pregnancy to about 3 years of age).
    - **Major Surgery**: The surgical procedure itself can cause stress and inflammatory responses.

Context of the Scientific Evidence

- **Animal Studies**: In young animals, high doses of anesthetic drugs over long periods have been shown to interfere with brain cell development. This is the origin of the concern.
- **Human Studies**: Early retrospective studies found a slightly higher incidence of learning or behavioral problems later in life among children who had multiple anesthetics. However, **it is impossible to determine if this was caused by the anesthesia itself, or by the underlying medical conditions that necessitated multiple surgeries, surgical trauma, family environment, or other combined factors.**
- **Latest Authoritative Research**: Several major recent prospective studies (such as the GAS and PANDA studies in the US) directly compared young children who had a single short anesthetic to their siblings who did not. **The results showed no significant difference in neurodevelopmental outcomes at ages 5 and 8.** This has greatly alleviated concerns in the medical community and among parents.

Core Advice for Parents

1. **Do Not Delay Necessary Care**: If your child **requires** a surgical procedure or medical test (e.g., hernia repair, appendectomy, complex dental work, imaging), **you should not refuse or delay treatment due to excessive fear of anesthesia**. The harm of an untreated condition to a child's health is clear and immediate.
2. **Communicate Fully with the Medical Team**: This is the most critical step. Before surgery, discuss in detail with the anesthesiologist and surgeon:
    - The necessity and urgency of the surgery.
    - The anesthesia plan (they will use the lowest effective dose for the shortest time appropriate for a child).
    - Your child's specific situation and any health issues.
3. **Provide Complete Information**: Inform the doctor about any history of prematurity, neurological conditions, developmental delays, drug allergies, or family history of problems with anesthesia.
4. **Be Mindful of Multiple Procedures**: If your child has a chronic condition requiring multiple surgeries, work with the anesthesiologist to create a long-term plan to minimize the number and duration of anesthetic exposures.

Summary

For a single, necessary, and reasonably short procedure, modern pediatric anesthesia is highly sophisticated and safe. You should not be overly anxious. The anesthesiologist's primary task is to ensure your child's safety and minimize all risks.

Always follow the professional assessment of your child's surgeon and anesthesiologist. They will provide the most individualized risk-benefit analysis and the safest anesthesia plan based on your child's specific condition.

Q7.Is general anesthesia necessary for my child's dental treatment?

Answer:

In a hospital setting, pediatric anesthesia is typically provided by a **specialized anesthesiologist** (a doctor trained in anesthesia). They are medical professionals with specific training and experience in safely anesthetizing children. In some cases, a certified nurse anesthetist or an anesthesia care team may work under the supervision of the anesthesiologist.

The general process includes:

1. **Preoperative Assessment:** The anesthesiologist will review your child's medical history, allergies, and overall health before surgery.
2. **Planning the Anesthesia:** They will develop a tailored anesthetic plan based on the type of surgery and your child's specific needs.
3. **Continuous Monitoring:** Throughout the procedure, the anesthesiologist will closely monitor your child's vital signs (heart rate, breathing, blood pressure, etc.).
4. **Recovery & Pain Management:** They will oversee your child's wake-up process and help manage pain after surgery.

You will have the opportunity to speak with the anesthesiologist before the procedure to discuss the plan and address any concerns you may have.

Q8.Who will give my child anesthesia?

Answer:

Your child’s anesthesia will be given by a **trained anesthesia professional**. Who that is can vary by hospital and country, but commonly includes:

- **Anesthesiologist** – a medical doctor (MD or DO) who specializes in anesthesia.
  - For children, this is often a **pediatric anesthesiologist**, who has extra training in caring for infants and children.
- **Certified Registered Nurse Anesthetist (CRNA)** – an advanced-practice nurse trained to give anesthesia.

- **Anesthesiologist Assistant (AA)** – a specially trained clinician who works under the supervision of an anesthesiologist.

In many hospitals, especially for children:

- An **anesthesiologist leads the anesthesia care**, and
- A CRNA or AA may **administer the anesthesia while the anesthesiologist supervises and is immediately available**.

Before the procedure, the anesthesia provider will:

- Review your child's medical history
- Explain the anesthesia plan
- Answer your questions
- Monitor your child closely throughout the procedure and recovery

If you'd like, tell me **where your child is having the procedure (hospital vs. surgery center)** or **what type of surgery it is**, and I can explain more specifically what to expect.

Q9.What if my child won't cooperate for anesthesia?

Answer:

If your child resists or is uncooperative before anesthesia, the medical team is well-prepared to handle this common situation. Here's what you can expect:

1. **Specialized Pediatric Anesthesia Team**
    - Anesthesiologists and nurses experienced in pediatrics use child-friendly techniques, such as play therapy or distraction, to ease anxiety.
    - Oral sedative medications (e.g., midazolam syrup) may be offered to help your child relax before entering the operating room.
2. **How Parents Can Help**
    - Stay calm and use a reassuring tone—children often mirror parental emotions.
    - Avoid discussing medical details in front of your child; instead, prepare them in advance using picture books or role-playing.
    - Bring a familiar comfort item (like a favorite toy or blanket) for added security.
3. **Safety Protocols**
    - If a child becomes distressed, the team may gently use safety measures (e.g., soft restraint with a blanket) to prevent sudden movement and ensure a smooth, safe induction.
    - Modern pediatric anesthesia includes continuous monitoring of vital signs, allowing the team to manage induction quickly and safely even if the child is briefly upset.

Important Notes

- Inform the anesthesia team about any special needs (such as autism, anxiety disorders, or past traumatic experiences) so they can tailor their approach.
- Most cases of non-cooperation are effectively managed by experienced professionals, with the goal of ensuring both physical safety and emotional comfort for your child.
- Never hesitate to ask questions or express concerns—communication between parents and the medical team is key to a positive experience.

Q10.What if my child is allergic to anesthesia?

Answer:
If your child has a known or suspected allergy to anesthesia, it's crucial to handle the situation proactively. Here is what you should do and what medical professionals will typically do:

1. Inform the Medical Team Thoroughly:

- Clearly communicate your child's complete allergy history, including any adverse reactions to medications, foods, or environmental allergens.
- Describe any past reactions in detail (e.g., rash, hives, swelling, difficulty breathing, nausea).

2. The Medical Team Will Take Precautions:

- **Risk Assessment:** They may review the history in detail and potentially conduct or recommend allergy testing (e.g., skin tests, blood tests) for specific anesthetic agents if the risk is high.
- **Alternative Plans:** Anesthesiologists can develop a personalized plan using alternative medications that are unlikely to cause a cross-reactive response.
- **Premedication:** In some cases, they may administer antihistamines or steroids before surgery to help prevent a reaction.
- **Enhanced Monitoring:** During the procedure, your child will be closely monitored for any signs of an allergic reaction. The team is trained and equipped to manage emergencies immediately.

3. Post-Procedure Vigilance:

- Some reactions can occur after surgery. Inform nurses and doctors of any new symptoms like rash, swelling, or breathing difficulties.

Key Takeaway:
Modern anesthesia has a high safety profile. The most critical step is **open and detailed communication with your child's surgeon and anesthesiologist before the procedure.** By providing a complete history, you enable them to create the safest possible anesthetic plan tailored for your child.

Q11.Will my child need any evaluation before anesthesia?

Answer:
Yes, your child will typically need an evaluation before anesthesia.

This is a standard and crucial safety measure to ensure the anesthesia plan is tailored to your child's specific needs. The evaluation generally includes:

- **Medical History Review:** The anesthesiologist will discuss your child's overall health, past illnesses, surgeries, current medications, any allergies (especially to medications), and any previous reactions to anesthesia.
- **Physical Examination:** This focuses on your child's heart, lungs, and airway.
- **Possible Tests:** Depending on your child's health and the type of surgery, the doctor may order some tests, such as blood tests.

The anesthesiologist will use this information to create the safest possible anesthetic plan. It is very important to provide complete and accurate information about your child's health history during this evaluation.

Q12.Can my child have anesthesia with a cold?

Answer:
This is a very important and common question. In general, **it is not advisable to proceed with elective (non-urgent) surgery and anesthesia when a child has a common cold. However, the final decision must be made by the anesthesiologist after a comprehensive assessment.**

Here is a detailed explanation and action guide:

Core Principle: Safety First

The anesthesiologist's primary concern is the safety of your child's **airway**. A cold can affect the respiratory system and increase anesthesia risks.

Main Risks Include:

1. **Airway Hyperreactivity**: Airways are more sensitive during a cold, making them more prone to spasm, laryngospasm, or bronchospasm during intubation and extubation, which can lead to oxygen deprivation.
2. **Increased Secretions**: Excess mucus and phlegm can block the airway or cause aspiration pneumonia during anesthesia.
3. **Higher Risk of Postoperative Complications**: The child may be more susceptible to postoperative coughing, breathing difficulties, pneumonia, or low blood oxygen levels.
4. **Fever**: The presence of a fever indicates an active infection, which increases the body's stress response. Elective surgery should be postponed.

How is the Decision Made? It Depends on Symptoms and Surgery Type.

It is usually recommended to postpone elective surgery if the child has any of the following:

- **Fever** (temperature over 38°C / 100.4°F).

- **General malaise, lethargy.**
- **Frequent, productive, or deep chesty cough.**
- **Thick, yellow, or green nasal discharge or phlegm.**
- **Labored breathing, wheezing, or abnormal lung sounds heard by a doctor.**

Proceeding *might* be considered (subject to strict evaluation by the anesthesiologist) if:

- Symptoms are **very mild**: only a slight clear runny nose, an occasional dry cough, **no fever**, and the child is active, playful, and eating normally.
- The surgery is **urgent or medically necessary** and cannot be delayed.

Action Steps for Parents:

1. **Absolutely DO NOT hide the illness**: Before surgery, you **must** honestly inform both the surgeon and the anesthesiologist about all of your child's cold symptoms, including runny nose, cough, and fever, even if they seem minor.
2. **Be prepared for assessment**: The anesthesiologist will evaluate your child on the day of surgery. This includes asking questions and listening to their lungs with a stethoscope. They may decide to "proceed," "observe further," or "postpone."
3. **Be mentally prepared for postponement**: For your child's absolute safety, if the doctor recommends delay, please understand and cooperate. Typically, it's advised to wait **1-2 weeks after all symptoms have completely resolved** to allow airway sensitivity to return to normal.
4. **Communicate proactively**: You can ask the doctor: "My child has a cold. Do you think anesthesia and surgery are safe today, or should we postpone?" Trust their professional judgment.

Summary

For elective surgery: When in doubt, it is generally safer to postpone if the child has active cold symptoms.
The **final decision rests with the anesthesiologist**, who will weigh the urgency of the surgery against the child's current physical condition to ensure the safest outcome.

Open and honest communication with your medical team is the single most critical step in ensuring your child's safety.

Q13.When does my child need to stop eating or drinking before anesthesia?

Answer:
When does my child need to stop eating or drinking before anesthesia?

General fasting guidelines are as follows:

- **Clear liquids** (e.g., water, clear apple juice): Stop at least **2 hours** before.
- **Breast milk**: Stop at least **4 hours** before.
- **Infant formula or milk**: Stop at least **6 hours** before.
- **Solid food**: Stop at least **6 to 8 hours** before.

Important: These are general guidelines. The specific timing may vary based on your child's age, medical condition, and the hospital's protocols. **Always follow the precise instructions provided by your child's anesthesiologist or surgeon for safety.**

Q14.If my child is breastfeeding, do we need to stop before their anesthesia?

Answer:
If your child is breastfeeding, you typically do **not** need to stop breastfeeding altogether before anesthesia.

However, **fasting guidelines** must be strictly followed to prevent the risk of aspiration (breathing stomach contents into the lungs) during the procedure. The standard recommendations for clear liquids and breast milk are generally more lenient than for formula or solid foods.

General fasting guidelines for breast milk (always confirm with your anesthesiologist):

- **Breast milk:** Usually **4 hours** of fasting is required.
- **Clear liquids** (water, apple juice): Usually **2 hours** of fasting is required.

Key points to discuss with your child's anesthesiologist and surgeon:

1. **Get specific instructions:** The required fasting time can vary based on your child's age, medical condition, and the type of procedure. The hospital will give you exact, personalized guidelines.
2. **Post-procedure feeding:** Plan for how and when you can resume breastfeeding after the surgery. This is often encouraged once the child is awake and alert.
3. **Medications:** Inform the team of any medications you are taking, as some can pass through breast milk.

In summary: Do not stop breastfeeding, but you must adhere to the prescribed fasting period (usually around 4 hours for breast milk) prior to the anesthesia. Always follow the precise instructions provided by your child's medical team.

Q15.What should I do if my child takes daily medicines before anesthesia?

Answer:
If your child takes daily medications and requires anesthesia, follow these steps to ensure safety:

1. **Inform the anesthesiologist in advance**: Disclose all medications your child takes regularly, including prescription drugs, over-the-counter medicines, supplements, or herbal remedies.

2. **Follow medical instructions**: The anesthesiologist will decide whether to continue, adjust, or temporarily stop certain medications based on the type of drug, your child's health, and the anesthesia plan. Do not change or stop any medication without explicit guidance.
3. **Keep a medication record**: Provide details such as drug names, dosages, timing, and reasons for use to help the medical team make informed decisions.
4. **Confirm instructions before the procedure**: During pre-operative consultations, reconfirm any specific directions regarding medications, fasting, or fluid intake.

Important: Open communication with the medical team is essential for your child's safety. If you have any questions, consult the doctor or pharmacist in advance.

Q16.If my child has asthma, diabetes, or other health issues, do I need to do anything special before anesthesia?

Answer:
If your child has asthma, diabetes, or other health conditions, special preparations before anesthesia are often necessary to ensure their safety. Here are general guidelines, but always follow the specific instructions provided by your child's medical team.

1. Inform the Medical Team in Advance

- Provide the anesthesiologist and surgeon with a full medical history, including details about the condition (e.g., severity, recent episodes), all current medications (prescription, over-the-counter, and supplements), and any relevant recent test results (e.g., lung function tests for asthma, blood glucose records for diabetes).

2. Ensure Stable Control of the Condition

- **For asthma**: Aim to prevent acute flare-ups before surgery. If your child has recent symptoms (coughing, wheezing), consult their doctor to optimize treatment. The anesthesia team may assess lung function and have bronchodilators available during the procedure.
- **For diabetes**: Maintain stable blood glucose levels. Work with your pediatrician or endocrinologist to adjust insulin or oral diabetes medications before surgery, as directed, to avoid hypo- or hyperglycemia during anesthesia.
- **For other health issues** (e.g., heart conditions, allergies): Ensure they are well-managed and that the medical team has a tailored plan.

3. Adjust Medications as Directed

- Some medications (e.g., asthma inhalers, insulin) may need to be continued or adjusted on the day of surgery. **Do not stop or change doses without explicit guidance** from the doctor.
- If your child uses long-term corticosteroids (e.g., for severe asthma), the anesthesia team may administer additional steroids to prevent adrenal insufficiency.

4. Complete Preoperative Assessments and Tests

- Additional tests may be required, such as blood work, electrocardiogram (ECG), chest X-ray, or pulmonary function tests, to evaluate your child's fitness for anesthesia.
- For diabetes, frequent blood glucose monitoring before surgery may be needed to ensure optimal levels.

5. Follow Fasting Instructions Carefully

- Fasting (no food or drink) is usually required before anesthesia. The timing will be specified by the medical team based on your child's age and condition. For diabetic children, special arrangements (e.g., intravenous fluids or glucose) may be made to manage blood sugar during fasting.

6. Emergency Planning

- The anesthesia team will prepare for potential complications (e.g., asthma attack, blood sugar fluctuations) with appropriate medications, equipment, and monitoring.

Important Reminder:

- **The above information is general and not a substitute for professional medical advice.** Each child's situation is unique, so preparations must be tailored by the pediatrician, anesthesiologist, and relevant specialists.
- Always communicate fully with the healthcare team before the procedure and follow their specific instructions to minimize risks and ensure a safe anesthesia experience.

If you have further questions or need clarification, consult your child's doctor or the anesthesia department directly. Wishing your child a safe and smooth procedure!

Q17.When should my child and I arrive at the hospital on surgery day for anesthesia?

Answer:
Please arrive at the hospital with your child **1.5 to 2 hours before the scheduled surgery time** on the day of the procedure. This allows sufficient time for necessary preparations:

1. **Preoperative Check-in:** Registration, verification of information, and signing of consent forms.
2. **Preoperative Preparation:** Changing into surgical attire and measuring vital signs.
3. **Anesthesia Evaluation:** The anesthesiologist will re-examine your child and discuss the anesthesia plan with you.
4. **Settling In:** Time for your child to acclimate to the environment and ease any anxiety.

The most important guideline is to follow the specific instructions provided by your hospital or surgical center (as outlined in your surgery appointment letter or pre-operative instructions). If unsure, it is best to contact the surgical department or preoperative clinic 1-2 days in advance to confirm the exact arrival time.

Q18.Can my child visit the surgery and recovery areas before anesthesia?

Answer:
Typically, **hospitals do not allow children to visit the actual operating and post-anesthesia recovery areas before surgery.**

The primary reasons are:

1. **Infection Control:** Operating rooms are strictly sterile environments. Unnecessary entry increases the risk of contamination.
2. **Safety and Workflow:** These areas must remain quiet, orderly, and ready for emergencies. Unfamiliar visitors can disrupt critical procedures.
3. **Potential for Anxiety:** The sight of medical equipment and the environment can be frightening for some children, potentially increasing pre-operative anxiety and making anesthesia induction more difficult.
4. **Privacy of Other Patients:** The recovery area houses other patients waking from surgery, and their privacy must be protected.

However, most hospitals offer excellent alternative methods to prepare your child and reduce anxiety:

- **Child Life Specialists & Preoperative Programs:** Many hospitals, especially pediatric centers, have **Child Life Specialists**. They use medical play, dolls, books, and videos to explain the process in an age-appropriate way. Some hospitals host **preoperative tours or "orientation days"** where children can visit a mock operating room or a designated waiting area.
- **Meeting the Anesthesia Team:** Before surgery, the anesthesiologist or nurse anesthetist will meet with you and your child. They can explain what will happen, show the anesthesia mask (sometimes with pleasant scents), and answer your child's questions.
- **Educational Materials:** Ask your hospital for child-friendly books, brochures, or online resources about having surgery.

What you can do:

1. **Ask Your Medical Team Directly:** Inquire if the hospital has a **Child Life Service** or a **preoperative preparation program** for children.
2. **Prepare at Home:** Use play, stories, or children's books about going to the hospital to familiarize your child with the idea in a positive, reassuring way.

In summary, while direct visitation to active clinical areas is not permitted for safety reasons, healthcare teams have developed specialized, child-centered methods to effectively prepare young patients for their experience. **Always discuss your child's specific needs and fears with their surgeon and anesthesiologist.**

Q19.Will my child be awake or know what's happening during anesthesia?

Answer:
Will my child be awake or know what's happening during general anesthesia?

No. Under general anesthesia, your child will be in a state of deep, controlled unconsciousness—**they will not be awake, feel pain, or have any awareness or memory of the procedure**.

Here's what typically happens:

1. **Induction**: Anesthesia is usually started either by having your child breathe anesthetic gas through a mask or through an intravenous (IV) line. They will fall asleep very quickly, usually within seconds.
2. **Maintenance**: Throughout the surgery, the anesthesiologist continuously administers medications to keep your child in a deep sleep, ensure they feel no pain, and keep their muscles relaxed if necessary.
3. **Emergence**: After the procedure, the medications are stopped. Your child will wake up gradually in the recovery room, often feeling as if they have had a long, deep nap.

Key Safety Points:

- The anesthesiologist monitors your child's vital signs (heart rate, blood pressure, oxygen levels) and brain activity throughout to ensure an appropriate depth of anesthesia.
- Modern anesthesia techniques and medications are highly refined and safe, with the explicit goal of providing **unconsciousness, amnesia (no memory), and analgesia (pain relief)**.

As a parent, you can help by:

- Providing the medical team with your child's complete health and medication history.
- Staying calm and reassuring your child before the procedure to reduce anxiety.
- Trusting the experienced anesthesia and surgical team.

In summary, you can be confident that your child will be completely unaware and will not remember the surgery. The experience is designed to be safe, pain-free, and without psychological trauma.

Q20.Will my child feel any pain while under anesthesia?

Answer:

Here are the key points:

1. **The Primary Goal of Anesthesia:** The core purpose of anesthesia is to ensure your child is **completely unconscious and feels no pain** during the entire surgical or medical procedure. Anesthetic drugs induce a state like deep sleep while simultaneously blocking pain signals.
2. **Professional Monitoring Throughout:**
    - A dedicated anesthesiologist or nurse anesthetist will closely monitor your child's vital signs (heart rate, blood pressure, oxygen levels, etc.) from start to finish and adjust the anesthesia depth as needed.

- The anesthetic state is **continuously maintained** throughout the procedure, ensuring your child does not wake up or experience pain.

3. **The Anesthesia Process (Induction):**
    - For children, especially young or anxious ones, anesthesia is often started by having them breathe anesthetic gas through a mask. This allows them to fall asleep peacefully.
    - Necessary IV lines or other invasive steps are typically placed **only after** the child is fully asleep.
    - Therefore, your child **will not experience the pain of an IV needle insertion while awake**.
4. **Postoperative Pain Management:**
    - The anesthesia team plans for postoperative pain relief in advance. This includes using safe, age-appropriate pain medication so that any surgical discomfort is managed effectively when your child wakes up.
    - Your child will wake up in the recovery room where nurses will promptly address any discomfort.

In summary:
Under the care of a qualified anesthesia team using modern techniques, your child will not feel any pain during the surgery itself. The entire process is designed to prioritize your child's safety, comfort, and complete absence of pain.

If you have specific concerns or your child has special medical needs, it is highly recommended to discuss them with the anesthesiologist before the procedure. They can provide personalized explanations and reassurance.

Q21.Will someone watch my child the whole time during anesthesia to keep them safe?

Answer:
Yes, your child will be under constant, one-on-one supervision throughout the entire anesthesia to ensure their safety.

This is a standard medical practice strictly followed during any anesthetic procedure. Specifically:

1. **Dedicated Professional**: A **qualified anesthesiologist or certified nurse anesthetist** will be assigned solely to monitor your child from start to finish. Their primary and continuous task is to manage your child's anesthesia and safety; they do not attend to other patients simultaneously.
2. **Continuous Monitoring**: From the moment anesthesia begins (induction) until your child is fully awake after the procedure, the professional will:
    - **Provide direct observation**: Closely watch for any reactions.
    - **Use advanced equipment**: Continuously track vital signs such as heart rate, blood pressure, oxygen levels, breathing rate, and temperature.
    - **Make immediate adjustments**: Precisely manage the depth of anesthesia and medications based on the real-time data and surgical needs.

3. **Team Approach**: The anesthesiologist works collaboratively with the surgeon, nurses, and other operating room staff as a team dedicated to your child's safe outcome.
4. **Recovery Care**: After the procedure, your child will be moved to a **Post-Anesthesia Care Unit (PACU or recovery room)**, where specialized nurses will continue close monitoring until your child is fully awake and stable.

In summary, you can be assured that modern pediatric anesthesia follows rigorous safety protocols. Your child will be under uninterrupted, expert supervision and monitoring from the moment anesthesia starts until they are safely awake. If you have specific concerns, you can discuss them in detail with the anesthesiologist during the pre-operative consultation.

Q22.Can I stay with my child during the anesthesia process?

Answer:
In many cases, **parents are allowed to stay with their child during the anesthesia induction process** until the child falls asleep. Policies may vary depending on the hospital, type of anesthesia, and the child's age or medical condition. Here's what you can do:

1. **Discuss in advance with the medical team**: Before the procedure, ask the anesthesiologist and nurses about the hospital's specific policy.
2. **Follow the healthcare team's guidance**: If staying is permitted, they will explain when you should leave (usually once your child is asleep).
3. **Stay calm and comfort your child**: Your presence can help ease your child's anxiety. Try to remain calm yourself to avoid transferring any nervousness to your child.

Important: The final arrangement depends on medical safety and hospital rules, so clear communication beforehand is key. Your support is a great comfort to your child!

Q23.How the anesthesia will be given to my child?

Answer:
The anesthesia process for your child is designed to be safe, gentle, and closely monitored. It is tailored to your child's age, health, and the specific procedure. Here is a general overview of how it will be given:

1. **Pre-operative Assessment:**
   - A pediatric anesthesiologist will meet with you and your child before the surgery. They will review your child's medical history, current medications, allergies, and any past experiences with anesthesia.
   - They will perform a physical examination and answer all your questions. It's crucial to inform them if your child has a cold, cough, or fever.
2. **Pre-medication (Optional, for anxiety):**

    - To help ease anxiety and fear, a mild sedative may be given before going to the operating room. This can be administered as a pleasant-tasting liquid by mouth, a nasal spray, or a rectal suppository.
    - This helps your child feel relaxed and drowsy, making separation from you easier. Often, children have little memory of this moment.
3. **Anesthesia Induction (Going to Sleep):**
    - This is the start of anesthesia. The method is chosen based on your child's needs:
        - **Inhalation Induction (Common for young children):** The anesthesiologist will have your child breathe anesthesia gas through a flavored (e.g., fruit-scented) mask. Your child will fall asleep gently within a minute or two. This is a painless process.
        - **Intravenous (IV) Induction:** For older or more cooperative children, or when needed, a small IV catheter may be placed in the hand or arm. Once the IV is in place, anesthetic medication is injected, and your child falls asleep very quickly. To minimize any discomfort from the IV start, a numbing cream may be applied to the skin beforehand.
4. **Anesthesia Maintenance & Monitoring:**
    - Once your child is asleep, the anesthesiologist will secure their airway (often with a soft breathing device like a laryngeal mask airway) and continue to deliver anesthetic gases and/or medications through the IV to maintain a deep, pain-free sleep throughout the surgery.
    - The pediatric anesthesiologist will **remain with your child at all times**, continuously monitoring vital signs (heart rate, blood pressure, oxygen levels, breathing) and adjusting the anesthesia depth as needed.
5. **Emergence & Recovery:**
    - At the end of the surgery, the anesthesiologist will stop the anesthetic medications. Your child will be taken to the **Post-Anesthesia Care Unit (PACU or recovery room)**.
    - Specialized nurses will monitor your child closely as they wake up. Parents are often called to be at the bedside as the child wakes.
    - Upon waking, your child may feel sleepy, dizzy, or have a scratchy throat (if a breathing tube was used). These effects are temporary.

Key Points for Your Reassurance:

- **Safety First:** Pediatric anesthesia follows strict safety protocols with advanced monitoring equipment.
- **Personalized Plan:** The anesthesia plan is customized specifically for your child.
- **Parental Support:** In many hospitals, one parent is allowed to accompany the child until the onset of anesthesia induction.
- **Expert Care:** Pediatric anesthesiologists are specially trained experts in managing anesthesia for children.

Most Important Source of Information:
Please discuss all details and concerns directly with your child's anesthesiologist

during the pre-operative interview. They can provide the most personalized and accurate information for your child's specific situation.

Q24.Will my child need help breathing during anesthesia?

Answer:
Yes, your child **will always receive assistance with breathing** while under general anesthesia. This is not only normal but a critical and standard safety measure throughout the procedure.

Here's why:

1. **Effect of Anesthetic Drugs**: General anesthesia medications temporarily suppress your child's natural urge to breathe and their protective airway reflexes.
2. **Ensuring Safety**: Having the anesthesiologist control breathing guarantees a stable supply of oxygen and maintains proper levels of oxygen and carbon dioxide in the blood, which is fundamental for safety during surgery.

The specific method depends on the surgery and typically involves one of two approaches:

- **Mask Ventilation**: For shorter, simpler procedures, the anesthesiologist will deliver oxygen and anesthetic gases through a mask placed over your child's nose and mouth and manually assist their breathing.
- **Endotracheal Intubation**: For longer, more complex, or abdominal/chest surgeries, the anesthesiologist will gently place a soft, thin breathing tube into your child's windpipe (trachea). This tube is connected to a ventilator machine that manages breathing precisely. This method best protects the airway, prevents any stomach contents from entering the lungs, and allows the use of muscle relaxants if needed.

Please be assured that:

- **Constant Monitoring**: Throughout the entire process, an anesthesiologist or certified anesthesia provider will be continuously at your child's bedside, using advanced equipment to monitor vital signs like heart rate, blood pressure, and oxygen saturation.
- **Standard Procedure**: This is the standard, routine practice of modern anesthesia designed to maximize your child's safety.
- **Pre-operative Discussion**: Before the surgery, you will have a chance to speak with the anesthesiologist. They will explain the specific anesthetic plan and breathing management for your child. This is the perfect time to ask any questions you may have.

In summary, assisted breathing during your child's anesthesia is an essential, expected, and safely managed part of the process, performed by a dedicated anesthesia team.

Q25.Will my child be sedated before getting anesthesia?

Answer:

Yes, it is very common and standard practice for **children to receive sedation before undergoing general anesthesia**. This is typically done for two main reasons:

1. **To Reduce Anxiety and Distress:** It helps prevent fear, crying, or struggling when separating from parents and entering the operating room. This creates a calmer, safer, and more positive experience for the child, minimizing psychological trauma.
2. **To Ensure a Smooth and Safe Induction:** A sedated child can cooperate more easily for the actual anesthesia induction, such as by accepting an anesthesia mask without fear, making the process smoother and safer for everyone.

In simple terms, the process often involves two key stages:

- **Step 1: Preoperative Sedation:** Your child will receive medication (usually as an oral syrup, a nasal spray, or through an IV) while still with you in the pre-operative area. This helps them become drowsy, relaxed, and often fall into a light sleep *before* going to the operating room.
- **Step 2: General Anesthesia:** After your child is sedated and separated from you, the anesthesiologist will administer the main general anesthetic drugs in the operating room (via mask or IV) to achieve a deeper state of unconsciousness for the surgery itself.

The specific method and medication for preoperative sedation are carefully chosen by the pediatric anesthesiologist based on your child's age, weight, medical condition, and the type of procedure.

You can rest assured that this entire process is managed and closely monitored by a specialized anesthesia team to ensure your child's utmost safety. You are encouraged to discuss any questions or concerns with the anesthesiologist during the preoperative consultation.

Q26.How soon will my child wake up after anesthesia?

Answer:

How soon a child wakes up after anesthesia varies by individual and the type of anesthesia used. Generally, they will awaken **within minutes to a few hours** after the procedure ends. The specific timing depends on several factors:

- **Type of Anesthesia:** The effects differ between general anesthesia, regional anesthesia, or sedation.
- **Length and Complexity of Surgery:** Longer or more complex procedures may require deeper anesthesia, leading to a slightly slower wake-up.
- **Child's Age and Health:** Infants, young children, or those with underlying health conditions may metabolize the anesthetic drugs more slowly.
- **Individual Response:** Each child's sensitivity to medications is unique.

Typically, the child is taken to a recovery room for monitoring until they are awake and their vital signs are stable. The medical team will closely observe them and provide you with guidance.

Important Note: This is general information only. Always follow the specific instructions of your child's anesthesiologist and medical team. If you have concerns or

if your child exhibits any unusual symptoms after waking, notify the healthcare professionals immediately.

Q27.When can I see my child after anesthesia and surgery?

Answer:

The timing of seeing your child after surgery primarily depends on the following factors:

1. **Type of Surgery and Anesthesia**
   Minor, short procedures (e.g., tonsillectomy, wound suturing) usually allow for quicker recovery from anesthesia, while more complex surgeries (e.g., cardiac or abdominal operations) require longer recovery times.
2. **Your Child's Recovery Progress**
   After surgery, your child will be moved to the **Post-Anesthesia Care Unit (PACU or recovery room)**, where medical staff will closely monitor them until their vital signs are stable and they are fully awake. **Parents are typically allowed to see their child only after the child has been transferred back to the regular ward.** The duration varies from child to child (usually between 30 minutes to several hours).
3. **Hospital Policies**
   Some hospitals allow one parent to enter the PACU during the initial awakening phase to comfort the child and reduce anxiety. Others, to maintain a sterile environment, may require parents to wait until the child returns to the ward.
4. **Special Circumstances**
   If your child is admitted to the Intensive Care Unit (ICU), visiting hours will follow the ICU's specific regulations, which may be limited to scheduled times.

Recommendations:

- **Discuss with the surgeon or anesthesiologist in advance** to understand the expected duration of surgery and the awakening process.
- **Wait patiently for notification from the medical staff** to ensure your child recovers safely from anesthesia.
- **After waking up, children may be fussy, drowsy, or nauseous** — these are common reactions. Stay calm and follow the medical team's guidance.

In general, if your child's condition is stable, **most parents can reunite with their child within 1–2 hours after surgery**. Please follow the specific arrangements and instructions provided by the hospital.

Q28.Will my child have pain after anesthesia?

Answer:
It is possible for your child to experience some pain after anesthesia. This pain is typically related to the surgery or medical procedure itself, not the anesthesia. While anesthesia ensures your child feels no pain during the operation, discomfort may be felt as its effects wear off.

The level of pain varies significantly depending on:

- The type and extent of the surgery.
- The method of anesthesia used.
- Your child's individual pain tolerance and recovery process.

Important Notes:

- The medical team will have a **pain management plan** in place. This often includes medications (like pain relievers) to keep your child comfortable during recovery.
- **Communication is key.** You can help by observing your child, comforting them, and informing the nurses or doctors if you believe they are in pain.
- Always **follow the specific instructions** provided by your child's surgeon and anesthesiologist regarding medication and care.

This information is general. For advice tailored to your child's specific situation, you must consult directly with your child's doctor or anesthesiologist.

Q29.Will my child feel sick or throw up after anesthesia?

Answer:

This is a very common concern for parents. **Yes, it is possible for children to feel nauseous or vomit after anesthesia, but it does not happen to every child. Modern anesthesia techniques have many methods to prevent and manage this.**

Here is a detailed explanation to help you understand the situation better:

1. Likelihood

- **Possible but Not Certain:** Postoperative nausea and vomiting (PONV) is a relatively common side effect in children, with an estimated incidence of **between 10% and 30%**, depending on various factors. This means **the majority of children do not experience it.**
- **Individual Variation:** Every child reacts differently to anesthesia, similar to how some people get motion sickness while others do not.

2. Main Causes

- **Anesthetic Agents:** Certain anesthetic drugs, especially inhaled gases and opioid pain medications, are primary triggers.
- **Type of Surgery:** Some procedures carry a higher risk, such as ear surgery (middle ear), eye surgery, tonsillectomy, and abdominal surgery.
- **Child-Specific Factors:**
    - Older children (>3 years) have a slightly higher risk than infants and toddlers.
    - Children with a history of motion sickness or previous PONV are at higher risk.
    - Post-pubescent girls have a slightly higher risk than boys.

3. How the Anesthesiologist Prevents and Manages This

This is the most important part. The anesthesia team will take multiple steps before, during, and after surgery to minimize the risk:

- **Preoperative Assessment:** They will ask about your child's history of motion sickness or previous anesthesia-related nausea.
- **Drug Selection:** They will choose anesthetic medications with a lower potential for causing nausea when appropriate.
- **Preventive Medication:** For children at higher risk, it is **standard practice to administer anti-nausea medication** (such as ondansetron or dexamethasone) proactively during the procedure.
- **Intraoperative & Postoperative Care:** Ensuring proper hydration, oxygenation, and close monitoring in the recovery room.

4. What You Can Do as a Parent

- **Provide Accurate History:** Be sure to inform the anesthesiologist if your child has a history of severe motion sickness, previous PONV, or a family history of it.
- **Follow Fasting Instructions Strictly:** Adhering to the pre-operative fasting rules (no food, no drink) is **crucial**. **Having contents in the stomach is a major risk factor for vomiting and serious complications like aspiration.** Do not give your child anything to eat or drink, no matter how tempted you might be.
- **Post-Op Feeding:** After surgery, follow instructions carefully. Start with small sips of clear liquids, and only advance to light foods if there is no nausea.
- **Keep Calm:** When your child wakes up, a calm and quiet environment can help. Avoid unnecessary movement or jostling.

Summary

While postoperative nausea and vomiting is a possibility, there is no need for excessive worry. Preventing and managing this issue is a standard part of modern pediatric anesthesia. The professional anesthesia team will take all necessary measures to ensure your child's comfort and safety.

The best advice is to discuss this concern directly with your child's anesthesiologist during the pre-operative interview. They can provide a personalized risk assessment and plan based on your child's specific age, surgery type, and medical history, which will offer you the greatest peace of mind.

Wishing your child a smooth surgery and a comfortable recovery!

Q30.If my child had spinal or epidural anesthesia, could they get a headache afterward?

Answer:
Yes, it is possible for your child to develop a headache after receiving **spinal or epidural anesthesia**. This specific type of headache is known as a **Post-Dural Puncture Headache (PDPH)**.

Here is a concise overview:

1. **Cause**:
    - The headache can occur if the needle used for the anesthesia creates a small opening in the dura mater (the tough membrane surrounding the

spinal cord and nerves). If cerebrospinal fluid (CSF) leaks through this opening, it can lower pressure around the brain, leading to a headache.
    - This is more common with **spinal anesthesia**, as the procedure intentionally punctures the dura. It is less common with a standard epidural, but can happen if the dura is accidentally nicked.
    - The use of modern, finer needles and techniques has significantly reduced its incidence.
2. **Characteristics**:
    - **Postural Headache**: The key feature is that the pain worsens significantly when sitting or standing and improves or resolves when lying down flat.
    - The pain is often located in the front or back of the head.
    - It may be accompanied by nausea, vomiting, neck stiffness, or sensitivity to light/sound.
    - It typically begins within **12 hours to 5 days** after the procedure.
3. **Likelihood**:
    - The overall risk in children and adolescents is generally **lower than in adults**, but it remains a known potential complication.
    - The incidence varies with technique, needle size, and patient age. It is closer to adult rates in teenagers and relatively rare in younger children.
4. **Management and Treatment**:
    - **Conservative Measures**: Initial steps include rest, adequate hydration (increased fluid intake), and over-the-counter pain relievers (e.g., ibuprofen, acetaminophen) as directed by a doctor.
    - **Epidural Blood Patch**: If the headache is severe and does not improve with conservative treatment, the most effective remedy is a procedure called an *epidural blood patch*. A small amount of the child's own blood is drawn and injected near the original puncture site in the epidural space. This forms a clot that seals the leak, often providing rapid relief.

Important Advice:
If your child develops a headache with the features described above after anesthesia, **it is essential to inform the surgeon, anesthesiologist, or return to the hospital for evaluation**. They can confirm the diagnosis and recommend the appropriate course of action. Most cases of PDPH resolve completely with proper management and leave no long-term effects.

Please note: This information is for educational purposes only and is not a substitute for professional medical advice. Always follow the guidance of your child's healthcare team. Wishing your child a speedy recovery.

Q31.How soon can my child eat and drink after waking up from general anesthesia?

Answer:

The timing for resuming eating and drinking after general anesthesia **must be strictly followed according to the specific instructions of the anesthesiologist and surgical team**, as it depends on the type of surgery, medications used, and the child's individual response. General guidelines are as follows:

General Recommended Timeline (Always follow medical advice)

1. **Immediately after waking** (within 1–2 hours)
   - Once fully awake (able to follow commands, swallow voluntarily), offer a small amount of **clear liquid** (e.g., 1–2 teaspoons of water).
   - If no nausea, vomiting, or choking occurs, gradually increase the amount.
2. **Clear fluids stage**
   - If water is tolerated, advance to **clear fluids** (e.g., diluted apple juice, clear electrolyte drinks).
   - Offer small amounts frequently, observing for 1–2 hours.
3. **Light, easy-to-digest foods**
   - If clear fluids are well tolerated, try **bland, soft foods** (e.g., porridge, applesauce, yogurt).
   - Avoid greasy, spicy, sugary, or solid foods initially.
4. **Return to regular diet**
   - Usually **4–6 hours or more after surgery**, if the child feels well with no discomfort, a normal diet can be gradually resumed.
   - Note: Certain surgeries (e.g., abdominal, gastrointestinal, or tonsillectomy) may require longer fasting periods.

Important Cautions

- **Do not force food or drink**: If the child is drowsy, irritable, or refuses, wait and inform the medical staff.
- **Risk of vomiting**: Gastrointestinal function recovers slowly after anesthesia. Eating too early may cause vomiting and potential aspiration.
- **Special surgical requirements**: Some procedures require extended fasting—always adhere to the medical team's instructions.

Key Principles

- **Advance gradually**: Follow the step-by-step approach: water → clear fluids → soft foods → regular diet.
- **Monitor closely**: Watch for any signs of nausea, vomiting, abdominal pain, or distress at each step.
- **Follow medical orders**: Protocols can vary by hospital and surgery type—always prioritize the medical team's guidance.

Seek immediate medical help if your child experiences persistent vomiting, difficulty breathing, or confusion.

Safety first: It is better to proceed cautiously than to rush feeding after anesthesia.

Q32.How long will my child need to stay at the hospital after anesthesia?

Answer:

The length of your child's hospital stay after anesthesia **depends primarily on the type of surgery, your child's overall health, and their recovery response.** There is no single answer, but here are the general scenarios:

1. **Day Surgery / Outpatient Procedure:** This is very common for minor surgeries. If the procedure is short, minimally invasive, and your child is healthy, they can usually go home **the same day**. They will be discharged after they are fully awake, vital signs (heart rate, blood pressure, oxygen levels) are stable, they can tolerate small amounts of fluids, and they have no serious nausea, vomiting, or pain. This typically requires a few hours of observation.
2. **Inpatient Surgery:** For major or complex operations, or if your child has underlying conditions (such as a history of prematurity, significant heart/lung disease, or sleep apnea), an **overnight stay or longer is usually necessary.** The duration depends on:
   - **The Surgery Itself:** Examples include abdominal, cardiac, or neurosurgery, which require close post-operative monitoring and care.
   - **Anesthesia Recovery:** Ensuring full stability of breathing and circulation, and that pain is well-managed.
   - **Post-Operative Progress:** Monitoring for complications like bleeding, infection, or fever, and tracking the return to normal diet and activity.
3. **Special Factors:**
   - **Age:** Newborns and infants often require longer observation.
   - **Complications:** If any anesthesia or surgery-related issues arise, the hospital stay will be extended.

The most important advice is:
Always follow the specific instructions and assessment provided by your child's surgeon and anesthesiologist. They will give you the most personalized and accurate timeline based on your child's individual case.

You can ask during the pre-operative consultation: **"Based on my child's specific surgery and health, how long do you anticipate they will need to stay in the hospital afterward?"** They will give you the best direct answer.

Q33.Who can I contact if I have more questions after my child's anesthesia?

Answer:

1. **Medical Facility Where Anesthesia Was Administered**
   - Contact the hospital or surgical center where the procedure took place. Ask for the **Anesthesiology Department** or the team responsible for postoperative follow-up.
2. **Attending Physician or Anesthesiologist**
   - Reach out to the **anesthesiologist** involved in your child's care or your child's **surgeon/primary doctor**. Their contact information is often provided before or after the procedure.
3. **Hospital Hotline or Postoperative Follow-Up Service**

- Many hospitals have a dedicated **postoperative care line** or follow-up team to address recovery-related questions.

4. **In Case of Emergency**
   - If your child experiences **difficulty breathing, severe pain, confusion, or any alarming symptoms**, seek immediate medical attention by calling emergency services or going to the nearest emergency room.

Recommendation:
Keep the contact details of your child's medical team readily available. During follow-up consultations, prepare your questions in advance and take notes. If needed, request written or verbal postoperative care instructions from the hospital.

Model 2

Q1. What are the different types of anesthesia?

Answer:

Anesthesia is broadly categorized by **how much sensation or consciousness it affects** and **where it works in the body**. The main types are:

1. General Anesthesia

- **What it does:** Makes you completely unconscious and unable to feel pain.
- **How it's given:** IV medications, inhaled gases, or both.
- **Used for:** Major surgeries (e.g., heart surgery, brain surgery, abdominal surgery).
- **Effects:** Loss of awareness, pain sensation, memory, and muscle movement.

2. Regional Anesthesia

- **What it does:** Numbs a large region of the body while you may stay awake or lightly sedated.
- **How it's given:** Injection near major nerves or the spinal cord.

Common types:

- **Spinal anesthesia:** Injected into spinal fluid; numbs the lower body (e.g., C-sections).
- **Epidural anesthesia:** Injected near the spinal cord; often used in childbirth and prolonged surgeries.
- **Peripheral nerve blocks:** Target specific nerves (e.g., arm, leg, shoulder surgery).

3. Local Anesthesia

- **What it does:** Numbs a small, specific area.
- **How it's given:** Injection, cream, gel, or spray.
- **Used for:** Minor procedures (e.g., stitches, dental work, mole removal).
- **You stay awake and alert.**

4. Sedation (Monitored Anesthesia Care)

- **What it does:** Relaxes you and may cause drowsiness or partial sleep.
- **Levels:**
- **Minimal sedation:** Calm but fully awake
- **Moderate sedation ("conscious sedation"):** Sleepy but responsive
- **Deep sedation:** Mostly asleep but can be awakened
- **Used for:** Endoscopy, colonoscopy, minor surgical or diagnostic procedures.

5. Topical Anesthesia

- **What it does:** Numbs the surface of skin or mucous membranes.
- **How it's given:** Creams, sprays, patches, gels.

- **Used for:** Eye procedures, skin biopsies, sore throat numbing.

Quick Comparison

**Type**	**Conscious?**	**Area Affected**	**Common Uses**
General	No	Whole body	Major surgery
Regional	Usually yes	Large region	Childbirth, limb surgery
Local	Yes	Small area	Minor procedures
Sedation	Partially	Whole body (relaxation)	Diagnostic procedures
Topical	Yes	Surface only	Skin/eye/throat

If you'd like, I can also explain **which type is safest**, **how doctors choose**, or **what it feels like during recovery**.

Q2. What is general anesthesia?

Answer:

General anesthesia is a medically induced state in which a person is made **completely unconscious** and **unable to feel pain** during surgery or certain medical procedures.

Key features of general anesthesia

- **Unconsciousness** – you are asleep and unaware of the procedure
- **Pain control** – you do not feel pain
- **Muscle relaxation** – helps surgeons operate safely
- **No memory** – you usually don't remember the procedure afterward

How it's given

General anesthesia can be administered:

- **Through inhaled gases** (via a mask or breathing tube)
- **Through intravenous (IV) medications**

Often, both methods are used together.

What happens during anesthesia

An anesthesiologist or nurse anesthetist:

- Monitors **breathing, heart rate, blood pressure, and oxygen levels**
- Adjusts anesthesia levels throughout the procedure

- Ensures safety before, during, and after surgery

Common uses

- Major surgeries (e.g., heart, brain, abdominal surgery)
- Procedures that would be too painful or complex while awake

Recovery

After the procedure, you may experience:

- Drowsiness or confusion
- Nausea or sore throat

These effects usually wear off within hours.

If you'd like, I can also explain how general anesthesia differs from**local** or **regional anesthesia**, or what happens step by step on surgery day.

Q3. How is anesthesia different for children compared to adults?

Answer:

Anesthesia for children differs from that for adults in several important ways due to differences in their physiology, size, and development. Here are some of the key factors:

1. Dosage and Drug Metabolism

- **Smaller Dosages**: Children require much smaller doses of anesthesia than adults due to their smaller body size. However, the dosages are not just based on weight; factors like age, liver and kidney function, and overall health play a role.
- **Metabolism**: Children's bodies metabolize anesthetic drugs faster than adults, particularly in infants and young children. This means they might need more frequent dosing, but the drugs also wear off faster.

2. Airway Management

- **Anatomy**: Children have smaller airways and higher risks for airway obstruction. Anesthesia providers must be very careful about securing the airway. The equipment used (like endotracheal tubes and laryngeal mask airways) is smaller and designed for children.
- **Breathing Patterns**: Children breathe faster and more irregularly compared to adults, which requires careful monitoring and adjustment of anesthetic drugs.

3. Response to Anesthesia

- **Emotional Response**: Children can be more sensitive to the emotional aspects of anesthesia, especially pre-procedure anxiety. They may require sedatives or other strategies to help them relax before the anesthesia is administered.
- **Depth of Anesthesia**: Children may respond more quickly to changes in the depth of anesthesia, so the anesthesiologist has to be extra cautious when adjusting.

4. Types of Anesthesia

- **Inhaled Anesthetics**: For younger children, general anesthesia is often induced with inhaled agents like sevoflurane because they can be less traumatic than intravenous methods. These drugs tend to be more tolerable for children and are easier to titrate.
- **IV vs. Mask Induction**: In adults, intravenous induction is common, but in children, anesthesia is often started with a mask to avoid the pain of an IV insertion. Once the child is asleep, the anesthesiologist may then insert an IV if needed.

5. Postoperative Care

- **Recovery**: Children generally recover faster from anesthesia than adults, but they are also more prone to nausea and vomiting post-surgery. Anti-nausea medication is often given to prevent this.
- **Pain Management**: Children may need more careful monitoring and adjusted pain relief strategies because they may have trouble expressing their pain or discomfort. Pediatric anesthesiologists are skilled in using age-appropriate pain management techniques.

6. Long-Term Effects

- **Neurodevelopmental Considerations**: There is ongoing research into the effects of anesthesia on the developing brain. Some studies suggest that prolonged or repeated anesthesia in early childhood could potentially affect cognitive development. Anesthesiologists take this into account when planning procedures for very young children, especially those requiring multiple surgeries.

7. Preoperative Considerations

- **Medical History**: Children are more likely to have congenital conditions or developmental delays that need to be considered in the anesthesia plan. Pediatric anesthesiologists often work closely with other specialists to account for these factors.
- **Fasting Guidelines**: The fasting rules before anesthesia (no food or drink for a certain number of hours) differ for children compared to adults, and pediatric guidelines may allow for shorter fasting periods in younger children.

In general, the key difference is that pediatric anesthesia requires special training and experience due to the physiological differences, emotional factors, and developmental considerations of children. That's why pediatric anesthesiologists are specifically trained to care for children undergoing surgery.

Q4.How is general anesthesia different from sedation?

Answer:

The difference between general anesthesia and sedation primarily lies in the depth of consciousness and the way the body responds.

General anesthesia:

- **Depth of unconsciousness**: It causes a deep, controlled loss of consciousness, meaning the patient is completely unaware of their surroundings and cannot feel any pain. This is typically used for more invasive procedures, like surgeries.
- **Body response**: It affects the entire body, including muscles, heart rate, and breathing. For safety, breathing support (like a ventilator) may be required during the

procedure.

• **Duration**: The effects can last longer, depending on the type of procedure being done.

• **Administration**: It's usually administered intravenously or through inhalation (gases).

Sedation:

• **Depth of consciousness**: Sedation can range from mild (where you're drowsy but still awake) to deep (where you are on the edge of consciousness, but not fully asleep). Most people still respond to verbal cues, but they feel relaxed and may have little to no memory of the procedure.

• **Body response**: It typically doesn't cause the same level of systemic effects as general anesthesia. The body usually maintains its ability to breathe independently, though respiratory support might still be provided if the sedation is deep enough.

• **Duration**: Sedation tends to wear off more quickly, and the patient recovers faster than from general anesthesia.

• **Administration**: It's usually administered intravenously or orally, and can also be inhaled (like nitrous oxide or "laughing gas").

In short, **general anesthesia puts you fully to sleep and keeps you unconscious**, while **sedation keeps you relaxed and possibly drowsy** but usually allows you to stay aware to some degree.

Q5.Is general anesthesia safe for my child?

Answer:

Short Answer: **for most children, general anesthesia is very safe** when it's given by trained pediatric anesthesia professionals. Millions of children receive it every year for surgeries and procedures.

Here's a clear breakdown to help you feel more informed:

Why it's considered safe

• **Specialized care:** Pediatric anesthesiologists are specifically trained to care for infants, children, and teens.

• **Careful monitoring:** During anesthesia, your child's heart rate, blood pressure, oxygen levels, and breathing are continuously monitored.

• **Modern medications:** Today's anesthetic drugs are well-studied and tailored to a child's age, weight, and medical history.

Common, usually mild side effects

These typically resolve within hours to a day:

• Sleepiness or grogginess

• Nausea or vomiting

• Sore throat (from the breathing tube)

- Temporary mood changes (crying, confusion, irritability)

Serious risks (rare)

Serious complications are **very uncommon**, especially in healthy children:

- Breathing or heart problems
- Allergic reactions to medications

The risk is higher in children with significant medical conditions (heart, lung, neurologic issues), which is why anesthesiologists review your child's history carefully beforehand.

Brain development concerns

You may have heard about this:

- Research suggests that **repeated or prolonged anesthesia** (over ~3 hours) in **children under 3 years old** *might* be associated with subtle learning or behavior differences later.
- **Single, short exposures** have **not** been shown to cause harm.
- When surgery is necessary, the benefits usually **far outweigh** any theoretical risk.

What you can do as a parent

- Make sure your child is evaluated by a **pediatric anesthesiologist** if possible
- Follow fasting instructions exactly
- Ask questions—your care team expects and welcomes them

If you'd like, tell me:

- your child's age
- the type of procedure
- whether it's elective or urgent

I can help you understand how anesthesia applies specifically to your child's situation.

Q6.Can anesthesia harm my child's brain development?

Answer:

The potential effects of anesthesia on brain development in children have been a topic of concern and research, particularly regarding young children and infants. While general anesthesia is considered safe for most children, there is evidence suggesting that repeated or prolonged exposure to anesthesia before the age of 3 may have some impact on brain development.

Some studies have shown that animal models and human data indicate that exposure to anesthesia at a young age can cause changes in brain development, particularly in areas associated with learning, memory, and behavior. However, the clinical significance of these findings in humans is still not fully understood, and the long-term effects remain uncertain.

Here are some key points to consider:

- **Single vs. Repeated Exposure**: A single exposure to anesthesia for a brief procedure is generally considered safe and is unlikely to have significant negative effects on brain development. However, repeated or prolonged exposure may increase the risks.
- **Age Factor**: The younger the child, particularly infants under 1 year, the more sensitive their developing brain may be to potential effects of anesthesia. But again, the evidence is not definitive, and the risks must be weighed against the necessity of the procedure.
- **Current Guidelines**: Medical professionals typically weigh the risks and benefits of using anesthesia in pediatric procedures. In some cases, if a child requires surgery or a medical procedure, the benefits of the procedure may far outweigh the potential risks of anesthesia.
- **Recent Studies**: Some research, such as studies funded by the U.S. National Institutes of Health (NIH), has raised awareness about this issue. The *Moral and Ethical Considerations in Pediatric Anesthesia* study, for example, found that children who had undergone multiple exposures to anesthesia during the early years had slight delays in cognitive development.

What Should You Do?

- **Talk to the Surgeon or Anesthesiologist**: If your child requires surgery or a procedure that involves anesthesia, ask the healthcare team about the risks, especially if your child is under the age of 3. They may take steps to minimize the duration of anesthesia or choose the safest type for your child.
- **Follow-up**: If your child has undergone anesthesia, keep an eye on developmental milestones and talk to your pediatrician if you have concerns about their growth or cognitive development.

While the evidence isn’t conclusive, it's good to stay informed and make decisions based on expert advice and the specifics of your child's medical needs.

Q7.Is general anesthesia necessary for my child's dental treatment?

Answer:

Whether general anesthesia is necessary for your child’s dental treatment depends on a few factors, such as the complexity of the procedure, the child’s age, anxiety level, or any special medical needs. Here are some situations where general anesthesia might be recommended:

- **Complex Procedures**: If your child needs extensive dental work, like multiple fillings or tooth extractions, general anesthesia might be used to ensure they are comfortable and safe throughout the procedure.
- **Fear or Anxiety**: Some children have intense fear or anxiety about dental visits, and general anesthesia can help them stay calm and still for the procedure.
- **Young Age**: Very young children may not be able to cooperate for treatment without sedation or anesthesia. In these cases, general anesthesia may be necessary to ensure safety and comfort.

- **Special Needs**: Children with certain medical or developmental conditions may require general anesthesia to make the procedure easier and safer for them.

However, many dental treatments can be done with local anesthesia, sedation (like nitrous oxide, also known as "laughing gas"), or oral sedatives, depending on the child's needs and the procedure. It's a good idea to discuss with your child's dentist to weigh the risks, benefits, and alternatives specific to your child's situation. Would you be comfortable with general anesthesia for your child, or are you looking for alternatives?

Q8.Who will give my child anesthesia?

Answer:

Your child's anesthesia will be given by a **trained anesthesia professional**. Who that is can vary by hospital and country, but commonly includes:

- **Anesthesiologist** – a medical doctor (MD or DO) who specializes in anesthesia.
    - For children, this is often a **pediatric anesthesiologist**, who has extra training in caring for infants and children.
- **Certified Registered Nurse Anesthetist (CRNA)** – an advanced-practice nurse trained to give anesthesia.
- **Anesthesiologist Assistant (AA)** – a specially trained clinician who works under the supervision of an anesthesiologist.

In many hospitals, especially for children:

- An **anesthesiologist leads the anesthesia care**, and
- A CRNA or AA may **administer the anesthesia while the anesthesiologist supervises and is immediately available**.

Before the procedure, the anesthesia provider will:

- Review your child's medical history
- Explain the anesthesia plan
- Answer your questions
- Monitor your child closely throughout the procedure and recovery

If you'd like, tell me **where your child is having the procedure (hospital vs. surgery center)** or **what type of surgery it is**, and I can explain more specifically what to expect.

Q9.What if my child won't cooperate for anesthesia?

Answer:

That's a very common worry—and anesthesia teams deal with this all the time. A child not cooperating **does not mean the procedure can't be done safely**. Here's how it's usually handled and what you can do.

How anesthesia teams manage uncooperative children

1. Gentle, child-centered approaches first

- Age-appropriate explanations ("magic air," "sleepy medicine")
- Distraction (videos, games, bubbles, music)
- Letting a parent stay during induction when possible
- Allowing the child to choose things like mask scent or video

2. Medication before anesthesia (very common)

If a child is too anxious or resistant:

- A **calming medicine** may be given by mouth, nose spray, or injection
- This makes them relaxed or sleepy *before* going to the operating room
- Once relaxed, anesthesia can be started smoothly and safely

3. Physical resistance is avoided whenever possible

- Teams are trained to **minimize distress**
- If holding is necessary, it's brief, gentle, and focused on safety—not punishment
- The goal is always emotional as well as physical safety

4. This does NOT traumatize most children

- Children typically **do not remember** induction
- Long-term fear is more likely when anxiety isn't addressed than when sedation is used appropriately

What you can do as a parent

- Stay calm (kids read your emotions instantly)
- Avoid saying things like "it won't hurt" or "don't be scared"
- Use neutral phrases:

"The doctors help kids fall asleep so their body can be fixed."

- Let the medical team lead—they do this every day
- Ask ahead of time about **pre-medication options**

Important reassurance

- **Anesthesia teams are experts in anxious and uncooperative children**
- Your child will not be denied care because of fear or resistance
- Safety comes first, always

If you'd like, tell me:

- Your child's **age**

- What kind of **procedure** they're having

I can give more specific guidance on what to expect and how to prepare them

Q10.What if my child is allergic to anesthesia?

Answer:

If a child is **allergic to anesthesia**, it's understandably scary—but it *doesn't automatically mean they can't have procedures*. What matters most is **which drug caused the reaction and what kind of reaction it was**.

Here's how this is usually handled:

1. True anesthesia allergies are rare

Most children who are said to be "allergic to anesthesia" actually had:

- Nausea or vomiting
- Agitation or confusion on waking
- Low blood pressure
- Slow breathing

These are **side effects**, not allergies.

A **true allergy** usually involves:

- Hives or widespread rash
- Swelling of lips, face, or throat
- Breathing difficulty
- Sudden drop in blood pressure (anaphylaxis)

2. Identify which anesthetic caused the reaction

Anesthesia isn't one drug—it's a **combination**, such as:

- Induction agents (e.g., propofol)
- Inhaled gases
- Muscle relaxants
- Pain medications
- Antibiotics or latex exposure during surgery

An anesthesiologist or allergist may:

- Review past surgical records
- Order allergy testing (when appropriate)
- Determine which drugs are safe alternatives

3. Safe alternatives almost always exist

If a true allergy is confirmed, doctors can:

- Avoid the specific drug
- Use a different class of anesthetic
- Use regional anesthesia (nerve block, spinal, epidural) when possible
- Modify medications and monitoring plans

Children with known anesthesia allergies **routinely undergo safe surgeries** with proper planning.

4. Before any future procedure

You should:

- Inform **every doctor and anesthesiologist** in advance
- Bring documentation of the reaction if available
- Ask for a **pre-anesthesia consultation**
- Consider a medical alert bracelet if the allergy is confirmed

5. Emergency situations

Hospitals are equipped to:

- Treat allergic reactions immediately
- Adjust anesthesia plans rapidly
- Monitor children at higher risk more closely

When to seek specialist input

If your child had a severe reaction:

- Ask for referral to a **pediatric allergist**
- Ask for a **pediatric anesthesiology consult** before elective procedures

If you'd like, I can help you:

- Understand whether a past reaction sounds like a true allergy
- Prepare questions for an anesthesiologist
- Figure out what records to gather before a procedure

Just let me know.

Q11.Will my child need any evaluation before anesthesia?

Answer:

In most cases, **yes—some level of evaluation is done before a child receives anesthesia**, but how extensive it is depends on your child's age, health, and the type of procedure.

What's usually included

- **Pre-anesthesia assessment** (often by an anesthesiologist or nurse):
    - Medical history (asthma, heart conditions, sleep apnea, prior surgeries)

- Previous reactions to anesthesia (in the child or close family)
- Current medications, allergies, recent illnesses

- **Physical exam**, focusing on the airway, heart, and lungs
- **Review of recent illnesses**

Colds, flu, fever, or cough can affect anesthesia timing.

Tests—often not needed

- **Routine blood tests or imaging are usually unnecessary** for healthy children having minor or moderate procedures.
- Tests may be ordered if your child has:
    - Chronic medical conditions
    - A history of bleeding problems
    - Heart, lung, or neuromuscular disease
    - A more complex or lengthy surgery planned

Special situations

- **Premature infants**, children with **developmental or genetic conditions**, or those with **significant medical issues** may need additional evaluation or specialist input.
- If your child recently had a **respiratory infection**, the anesthesia team may delay the procedure for safety.

What you can do

- Be ready to share your child's **full medical history**
- Mention **any family history of anesthesia problems**
- Follow **fasting (NPO) instructions** carefully—they're very important for safety

If you'd like, tell me **your child's age, general health, and the type of procedure**, and I can give you a more tailored idea of what evaluation is typically required.

Q12.Can my child have anesthesia with a cold?

Answer:

Short answer: **sometimes—but it depends on how sick the child is and the type of surgery/anesthesia.**

When anesthesia may still be okay

Doctors often proceed if the child has **only mild cold symptoms**, such as:

- Clear runny nose
- Mild cough
- No fever
- Normal energy level

- Eating and drinking fairly well

This is especially true for **older children** and **short, non-airway surgeries**.

When anesthesia is usually postponed

Anesthesiologists are more likely to delay surgery if your child has:

- **Fever** (often ≥38°C / 100.4°F)
- **Wheezing or trouble breathing**
- **Deep, wet, or worsening cough**
- **Green/yellow nasal mucus**
- **Lethargy or poor feeding**
- A history of **asthma, prematurity, or recent RSV/flu/COVID**

These increase the risk of breathing problems during and after anesthesia.

Why colds matter with anesthesia

Colds can make the airways more sensitive, which raises the risk of:

- Laryngospasm (airway tightening)
- Bronchospasm (wheezing)
- Low oxygen levels
- Prolonged coughing after surgery

What you should do

- **Tell the surgeon and anesthesiologist about all symptoms**, even if they seem mild.
- The **anesthesiologist makes the final decision**, often on the day of surgery.
- If symptoms worsen before surgery day, **call the surgical team**.

Reassurance

This is very common, and postponing surgery—if needed—is done for safety, not because something is wrong.

If you'd like, tell me:

- Your child's **age**
- **Type of surgery**
- Current **symptoms and fever status**

I can give more tailored guidance.

Q13.When does my child need to stop eating or drinking before anesthesia?

Answer:

Fasting before anesthesia is very important to reduce the risk of vomiting and aspiration. The exact timing can vary a bit by hospital and by your child's age, but **these are the most commonly used guidelines**:

General fasting (NPO) guidelines for children
Before anesthesia, your child should stop:

- **Solid food & fatty meals:**
  8 hours befanesthesia
  (includes fried foods, pizza, fast food)
- **Light meals / non-human milk / formula:**
  6 hours before anesthesia
  (toast, cereal, milk, formula)
- **Breast milk:**
  4 hours before anesthesia
- **Clear liquids:**
  2 hours before anesthesia
  (water, clear apple juice, Pedialyte, clear sports drinks)
  No milk, juice with pulp, smoothies, or gum

Important notes

- "Clear liquids" means you can see through them.
- Chewing gum or candy usually counts as eating—avoid it.
- If your child accidentally eats or drinks too late, **tell the surgical team**—don't hide it. The procedure may need to be delayed for safety.

Always follow your care team's instructions

Your anesthesiologist or surgeon may give **slightly different rules** based on:

- Your child's age
- The type of procedure
- Medical conditions (e.g., reflux, diabetes)

If you'd like, tell me:

- Your child's age
- The type of procedure

and I can help you double-check what's most likely to apply.

Q14.If my child is breastfeeding, do we need to stop before their anesthesia?

Answer:
Short Answer: usually no—you don't have to stop breastfeeding entirely, but timing matters before anesthesia.
Before anesthesia (fasting rules)

For infants and young children who breastfeed, most anesthesia guidelines allow:

- **Breast milk up to ~4 hours before anesthesia**
- Clear liquids (like water or cl4ear electrolyte solution, if allowed) up to ~2 hours before

This reduces the risk of vomiting and aspiration while the child is asleep under anesthesia. Your surgical or anesthesia team may give slightly different instructions depending on your child's age, health, and the type of procedure—**their instructions always take priority**.

After anesthesia

- **Breastfeeding can usually resume as soon as your child is awake, alert, and not vomiting**
- It's common for kids to be sleepy or mildly nauseated at first; once that settles, breastfeeding is fine
- Anesthetic medications used in children do **not** require you to "pump and dump" or delay feeds afterward

Important notes

- If your child was born prematurely or has medical conditions (airway issues, reflux, neurologic problems), fasting rules may be more conservative.
- Always confirm timing with the **anesthesiologist or pre-op team**, especially if instructions differ from what you've heard elsewhere.

If you'd like, tell me **your child's age** and whether this is **general anesthesia or sedation**, and I can help you interpret the typical instructions more specifically.

Q15.What should I do if my child takes daily medicines before anesthesia?

Answer:

If your child takes daily medicines and is scheduled for anesthesia, the most important rule is **don't stop or give anything differently unless the anesthesia team tells you to**. Here's how this is usually handled:

What to do before anesthesia

- **Make a complete medication list**
- Include **all prescription medicines**, inhalers, eye/ear drops, patches, **over-the-counter meds**, vitamins, and herbal supplements.
- Note the **dose, timing, and reason** for each.
- **Share the list early**
- Provide it to the surgeon and the anesthesia team as soon as possible (often during pre-op screening).
- **Follow the anesthesia team's instructions exactly**
- Some medicines are **continued** the morning of surgery (often with a small sip of water).
- Others may need to be **held** for a day or more beforehand.

- Instructions can vary based on your child's age, condition, and type of surgery.

Common examples (general guidance only)

- **Usually continued** (unless told otherwise):

  Asthma inhalers

  Seizure medications

  Heart and blood pressure medicines

- **Often adjusted or held**:

  Diabetes medicines (especially insulin)

  Blood thinners

  Certain behavioral/ADHD medications

  Herbal supplements (often stopped 1–2 weeks before)

Day of surgery

- Give **only the medicines you were specifically told to give**.
- Use **only a small sip of water** if permitted (to maintain fasting safety).
- Bring the medication list—and sometimes the medicines themselves—with you.

If you're unsure

- **Call the anesthesia or pre-op clinic**—they'd much rather clarify ahead of time than have uncertainty on surgery day.

If you'd like, you can tell me **your child's age and which medicines they take**, and I can help you think through what questions to ask the anesthesia team.

Q16.If my child has asthma, diabetes, or other health issues, do I need to do anything special before anesthesia?

Answer:

Yes. If your child has **asthma, diabetes, or other medical conditions**, it's very important to take a few extra steps **before anesthesia** so the anesthesia team can keep them safe. Here's what typically matters most:

What you should always do

• **Tell the surgical and anesthesia team** about *all* medical conditions, even if they seem mild or well-controlled.

• **List all medications** your child takes (including inhalers, insulin, supplements, and rescue meds).

• **Mention recent illnesses**, especially colds, cough, fever, or breathing problems.

• **Follow fasting (NPO) instructions exactly**, unless the team gives condition-specific instructions.

Condition-specific considerations

Asthma or other lung problems

• Tell them how often symptoms occur and if there have been **recent flare-ups or ER visits**.

• Your child may be asked to:

  • Use their **inhaler or nebulizer before surgery**

  • **Bring their rescue inhaler** on the day of surgery

• Surgery may be postponed if asthma is poorly controlled or your child has a respiratory infection.

Diabetes

• The team will give **specific instructions about insulin or oral medications** the night before and morning of surgery.

• Blood sugar will usually be checked **before, during, and after anesthesia**.

• Let them know:

  • Typical blood sugar ranges

  • Any recent low or high blood sugar episodes

• Do **not** adjust doses on your own unless instructed.

Other health conditions (heart, seizures, allergies, genetic conditions)

• Some medications may need to be **continued**, others held — the anesthesia team will guide you.

• **Seizure medications**, heart meds, and steroids are often continued.

• Tell them about **any past reactions to anesthesia** in your child or close relatives.

Reassurance

Anesthesiologists care for children with complex medical conditions every day. The most important thing you can do is **share complete and accurate information** — that allows them to plan the safest anesthesia approach.

If you'd like, I can help you **prepare a checklist** or explain what typically happens during the pre-anesthesia evaluation.

Q17.When should my child and I arrive at the hospital on surgery day for anesthesia?

Answer:

On surgery day, **most hospitals ask you and your child to arrive about *1–2 hours before* the scheduled surgery time** for anesthesia and pre-op preparation. That said, the exact timing can vary, so always follow the instructions your surgical team or hospital gave you.

Here's what that arrival window is usually for:

Why you arrive early

- Check-in and paperwork
- Nursing assessment (vitals, weight, review of fasting times)
- Meeting the anesthesiologist
- Starting an IV if needed (often after numbing cream)
- Giving any pre-medication to help with anxiety
- Final safety checks

Typical examples

- **Outpatient/day surgery:** arrive ~1–1.5 hours early
- **More complex surgery or very young children:** arrive closer to 2 hours early
- **First case of the day:** arrival may be very early (often 5:30–6:30 a.m.)

Very important reminders

- Follow **NPO (nothing by mouth)** rules exactly—this is critical for safe anesthesia
- Bring any requested documents, insurance info, and comfort items (favorite toy/blanket)
- Expect that surgery times can shift; arrival time usually stays the same even if surgery is delayed

If you'd like, tell me:

- Your child's age
- Whether it's outpatient or inpatient surgery
- The scheduled surgery time

I can help you estimate a more precise timeline for your day.

Q18.Can my child visit the surgery and recovery areas before anesthesia?

Answer:

In many hospitals, **yes—children can often visit the pre-operative (surgery prep) and recovery (PACU) areas before anesthesia**, but it depends on the facility and your child's situation.

Here's how it usually works:

Pre-operative area (before anesthesia)

- Parents are **almost always allowed** with their child.
- Many hospitals encourage this to reduce anxiety.
- Some offer **pre-surgery tours** or child-life specialists who explain what will happen in kid-friendly terms.
- Your child may be able to see the room, equipment, and meet staff ahead of time.

Recovery area (after anesthesia)

- Policies vary more here.
- Often **one parent is allowed** once the child is awake or stable.
- A **brief visit ahead of time** may be possible, especially if arranged in advance.
- Space, privacy, and infection-control rules can limit access.

Factors that affect access

- Your child's **age**
- Type of surgery
- Hospital safety and privacy policies
- Whether the hospital has a **pediatric unit or child-life program**

What you can do

- Call the hospital's **pre-op or anesthesia department** and ask specifically about:
    - Pre-surgery tours
    - Visiting the recovery area beforehand
    - Child-life services
- Ask if your child can **walk to the OR doors** or stay with you until anesthesia starts (common in pediatric cases).

If you'd like, tell me:

- Your child's age
- Whether this is a pediatric hospital or a general hospital

I can help you prepare questions or explain what your child is likely to see.

Q19.Will my child be awake or know what's happening during anesthesia?

Answer:

In most cases, **no**—your child will **not be awake or aware of what's happening** during anesthesia.

Here's what typically happens with **general anesthesia**, which is commonly used for children:

- **Your child will be fully asleep** and unconscious during the procedure.
- They **won't feel pain**, hear conversations, or remember what happens.
- The anesthesia team carefully monitors breathing, heart rate, blood pressure, and oxygen the entire time.

A few related points that may be helpful:

- **Before anesthesia:** Some children may feel sleepy, relaxed, or a little silly as the medicine starts working. Younger children may not even realize they're falling asleep.
- **After anesthesia:** It's normal for kids to wake up groggy, confused, or

emotional (crying or agitation). This usually passes within minutes to an hour.

- **Awareness during surgery** (being awake or remembering it) is **extremely rare**, especially in children.

If your child is having **sedation** (lighter than general anesthesia), they may be very sleepy but not fully unconscious—however, they usually **still don't remember the procedure**.

If you'd like, I can tailor this more closely if you tell me:

- your child's age
- what type of procedure they're having
- whether the doctor mentioned general anesthesia or sedation

Q20.Will my child feel any pain while under anesthesia?

Answer:

It's very natural to worry about this. The reassuring answer is **no—your child should not feel pain while they are under anesthesia**.

Here's how it works:

- **General anesthesia** (the kind that makes a child fully asleep) turns off awareness and pain sensation. During the procedure, the anesthesia team carefully monitors your child's depth of sleep, breathing, heart rate, and comfort the entire time.
- **Local or regional anesthesia** (numbing only part of the body) blocks pain signals from that area. If it's used with sedation, your child will also be relaxed or sleepy and not distressed.
- Anesthesiologists are specially trained to **anticipate pain and prevent it**, often giving pain medication *before* the procedure begins and adjusting it as needed throughout.

After the procedure:

- Your child may feel **some discomfort or soreness as the anesthesia wears off**, but this is expected and can usually be well controlled with medications recommended by the care team.
- The team will focus on keeping your child comfortable as they wake up.

If you'd like, you can tell me:

- What type of procedure your child is having, or
- How old your child is

and I can explain what anesthesia and pain control typically look like in that situation.

Q21.Will someone watch my child the whole time during anesthesia to keep them safe?

Answer:

Yes. **Your child will be watched continuously the entire time** they are under

anesthesia, with the sole focus of keeping them safe.

Here's what that typically looks like:

- **A dedicated anesthesia professional** (usually a pediatric anesthesiologist or a nurse anesthetist under their supervision) stays with your child **from the moment anesthesia starts until they are awake and stable**.
- They **do not leave your child at any point** during anesthesia.
- Your child is connected to monitors that track:
  - Heart rate
  - Blood pressure
  - Oxygen levels
  - Breathing
  - Carbon dioxide levels
- The anesthesia provider is trained to **recognize and respond immediately** to even small changes.

After the procedure:

- Your child is taken to a **recovery area (PACU)** where **trained nurses continue one-on-one monitoring** as they wake up.
- The anesthesia team remains available and checks on them.

For children especially, safety protocols are very strict, and pediatric anesthesia providers are specifically trained to care for kids of all ages.

If you'd like, I can also explain:

- What happens right before anesthesia starts
- How kids usually wake up afterward
- What questions are good to ask the anesthesia team beforehand

You're asking an important and very normal question—many parents worry about this.

Q22.Can I stay with my child during the anesthesia process?

Answer:

In many cases, **yes—parents are allowed to stay with their child for part of the anesthesia process**, but it depends on the hospital's policy, your child's age, and the type of procedure.

Here's how it usually works:

Before anesthesia (induction)

- **Commonly allowed:** Many hospitals let one parent or caregiver stay while anesthesia is started, especially for **infants and young children**, to help keep them calm.

- You may be asked to:
  - Wear a gown, mask, or hair cover
  - Leave once your child is asleep

When it may not be allowed

- Emergency situations
- Complex or high-risk procedures
- Older children or teens who prefer privacy
- Operating rooms with strict sterility or space limits

After anesthesia (recovery)

- **Almost always allowed:** Parents are typically invited to be with their child in the recovery room as soon as it's safe.

What you should do

- Ask the **anesthesiologist or surgical team ahead of time**:
  - "Can I stay with my child while anesthesia is started?"
  - "When can I be with them again after surgery?"
- Let them know if your child has anxiety or special needs—this often increases flexibility.

If you'd like, tell me **your child's age and the type of procedure**, and I can give more specific guidance.

Q23.How the anesthesia will be given to my child?

Answer:

I can explain this in a general way. The exact method depends on your child's age, health, and the type of procedure, but anesthesia for children is given very carefully and safely by a pediatric anesthesia team.

How anesthesia is usually given to children

1. Before anesthesia

- An anesthesiologist (a doctor who gives anesthesia) will review your child's medical history, allergies, and any previous reactions.
- Your child may be asked not to eat or drink for a certain number of hours before the procedure.
- Sometimes a calming medicine is given first, especially if the child is anxious.

2. How your child goes to sleep

There are two common ways:

a) Breathing anesthesia (most common for young children)

- Your child breathes anesthetic gas through a soft mask.
- They fall asleep gradually within a few minutes.

- Once asleep, an IV (small plastic tube in a vein) may be placed so medicines can be given during the procedure.

b) IV anesthesia (more common in older children or teens)

- An IV is placed first.
- Anesthesia medicine is given through the IV, and your child falls asleep quickly.

3. During the procedure

- Your child is continuously monitored (heart rate, breathing, blood pressure, oxygen level).
- The anesthesiologist stays with your child the entire time.
- Pain medicine is given so your child does not feel pain.

4. Waking up after anesthesia

- After the procedure, anesthesia is stopped.
- Your child wakes up in a recovery area.
- It's normal for children to be sleepy, confused, or a little irritable at first.
- Nausea or sore throat can happen but usually goes away quickly.

Safety reassurance

- Pediatric anesthesia is very safe when given by trained professionals.
- Doses are carefully calculated based on your child's weight and condition.
- You will be informed and asked for consent before anything is done.

If you'd like, tell me:

- your child's age, or
- what type of procedure is planned

and I can explain more specifically what to expect.

Q24.Will my child need help breathing during anesthesia?

Answer:

In most cases, **yes—but in a controlled and temporary way**, and it's very normal.

During anesthesia, medicines make children sleep deeply and relax their muscles. Because of that, they usually **can't maintain their airway or breathe effectively on their own**, so the anesthesia team **assists their breathing** to keep oxygen levels safe.

What that help looks like depends on the situation:

- **Light anesthesia or short procedures:**

  Your child may breathe on their own, with gentle support like extra oxygen and careful positioning.

- **General anesthesia:**

  The anesthesiologist typically uses:

  a **face mask**, or

  a **breathing tube** (such as an endotracheal tube or laryngeal mask airway)

  to make sure air moves in and out safely.

Key reassuring points:

- This is **routine and expected**, not an emergency measure.
- Pediatric anesthesiologists are specially trained to manage children's airways.
- Your child will be **continuously monitored** (oxygen level, heart rate, breathing) the entire time.
- Once the anesthesia wears off, **breathing returns to normal**, and the support is removed.

If you'd like, I can explain:

- how breathing is managed for a **specific age** (infant vs older child),
- what happens **before and after** anesthesia,
- or how anesthesiologists assess breathing safety ahead of time.

Q25.Will my child be sedated before getting anesthesia?

Answer:

In most cases, **yes—children are usually given something to help them relax or fall asleep *before* full anesthesia**, but how this is done depends on the child's age, anxiety level, and the type of procedure.

Here's what typically happens:

Before anesthesia

- **Young children** are often **not fully "sedated" ahead of time**, but they may receive:

  A **calming medicine** (often midazolam) by mouth, nose spray, or injection

  This makes them relaxed, sleepy, and less anxious

- **Infants and toddlers** may simply be comforted by a parent while anesthesia is started, sometimes using a **mask with sleepy gas** (no needle at first).

During anesthesia induction

- Many children go to sleep by:

  Breathing anesthesia gas through a mask, or

  IV anesthesia if an IV is already in place or needed

- Once asleep, they won't feel or remember what happens next.

For older children or teens

- They may receive:

  A calming medication beforehand **or**

  Go straight to anesthesia with an IV, depending on comfort and preference

Parent presence

- In some hospitals, **a parent can stay with the child** until they fall asleep—this varies by facility.

Important to know

- The anesthesia team's goal is **comfort, safety, and minimal stress**
- They tailor the approach to each child
- You'll usually discuss this plan with the anesthesiologist beforehand

If you'd like, tell me:

- Your child's **age**
- What kind of **procedure** they're having

I can explain exactly what is most likely to happen in your situation.

Q26.How soon will my child wake up after anesthesia?

Answer:

Most children start to **wake up within 15–60 minutes** after anesthesia is stopped, but the exact timing varies. Here's what usually happens:

Typical timeline

- **First signs of waking:** 10–30 minutes
  (moving, opening eyes, responding to voices)
- **More alert:** 30–60 minutes
  (able to talk or cry, recognize parents)
- **Back to baseline:** a few hours
  (still tired, but acting more like themselves)

What affects how fast they wake up

- **Type of anesthesia** (general anesthesia lasts longer than light sedation)
- **Length of the procedure**
- **Your child's age** (younger children may take a bit longer)
- **Medications used for pain or nausea**
- **Individual sensitivity** to anesthesia

What's normal after waking

- Sleepiness or grogginess
- Crying, confusion, or agitation ("emergence delirium"), especially in toddlers
- Nausea or mild dizziness

These usually improve within **30–90 minutes**.

When to tell the medical team

Let the recovery staff know right away if your child:

- Is hard to wake or not responding after **1–2 hours**
- Has trouble breathing
- Has persistent vomiting
- Seems unusually confused or weak for several hours

If you'd like, I can tailor this more closely—just tell me **your child's age** and whether this was **general anesthesia or sedation**.

Q27.When can I see my child after anesthesia and surgery?

Answer:

In most cases, **you can see your child fairly soon after surgery**, but the exact timing depends on how they're waking up and the hospital's policies.

Here's what usually happens:

Typical timeline

- **Recovery room (PACU):** After anesthesia, your child is taken to a recovery area and closely monitored.
- **When you're allowed in:** Many hospitals allow a parent to come back **within 15–30 minutes** once the child is stable and starting to wake up.
- **Sometimes longer:** If your child needs extra monitoring (for breathing, pain control, or nausea), staff may ask you to wait a bit longer.

What your child may be like

When you first see them, your child might:

- Be very sleepy or groggy
- Be confused, crying, or restless (this is common and temporary)
- Have an IV, monitors, or oxygen in place

This is all normal after anesthesia.

Factors that affect timing

- Your child's age
- Type and length of surgery
- How quickly they wake up from anesthesia
- Hospital or surgical center rules (especially in pediatric units)

If you're worried

You can always ask:

- **"When will I be allowed to see my child?"**

- **“Can I be with them as they wake up?”**

The recovery room staff will keep you updated and bring you back as soon as it’s safe.

If you’d like, tell me **your child’s age** or whether this is **outpatient or inpatient surgery**, and I can give more specific expectations.

Q28.Will my child have pain after anesthesia?

Answer:

Short answer: **most children do not have pain *from* the anesthesia itself**, but they may have **pain or discomfort from the procedure** once the anesthesia wears off.

Here’s what’s typical

After anesthesia, children may experience:

- **Soreness or pain** related to the surgery or procedure (this is the most common cause)
- **Headache**
- **Sore throat** (if a breathing tube was used)
- **Muscle aches**
- **Nausea or vomiting**
- **Sleepiness, grogginess, or irritability**

These effects are usually **temporary** and improve within **hours to a couple of days**.

Pain control

Doctors usually plan ahead for this and may:

- Give **pain medicine during or after the procedure**
- Recommend **acetaminophen (Tylenol)** or **ibuprofen** at home
- Prescribe stronger medication if needed for more painful surgeries

Always follow the dosing instructions given by your child’s care team.

When to contact the doctor

Call your child’s doctor if you notice:

- Pain that is **severe or getting worse**
- Pain **not relieved** by recommended medication
- **Persistent vomiting**, fever, or unusual behavior
- Trouble breathing or extreme sleepiness that doesn’t improve

Reassurance

Anesthesia itself **does not cause lasting pain**, and anesthesiologists are trained to keep children as comfortable and safe as possible before, during, and after the procedure.

If you’d like, tell me:

- Your child's **age**
- The **type of procedure or surgery**

I can give more specific expectations and pain-management tips.

Q29.Will my child feel sick or throw up after anesthesia?

Answer:

It's **possible**, but many children **do not** feel sick or throw up after anesthesia—and when it does happen, it's usually **mild and short-lived**.

How common is it?

- **Nausea or vomiting** after anesthesia (called *post-operative nausea and vomiting*, or PONV) happens in **some children**, especially in the first few hours after waking up.
- Most kids feel better within **a few hours**, and almost always within **24 hours**.

What increases the chance?

Your child may be a little more likely to feel sick if they:

- Are **older than 3 years**
- Have a **history of motion sickness**
- Have had nausea/vomiting after anesthesia before
- Had **eye, ear, throat, or abdominal surgery**
- Received certain pain medicines (like opioids)

What doctors do to prevent it

Anesthesia teams are very aware of this and often:

- Give **anti-nausea medication during surgery**
- Adjust anesthesia drugs to reduce risk
- Encourage **slow reintroduction of fluids and food**

What you might notice afterward

Normal and expected:

- Mild nausea
- One or two episodes of vomiting
- Sleepiness with decreased appetite

What you can do at home

- Start with **small sips of clear liquids** (water, apple juice, electrolyte drinks)
- Avoid heavy or greasy foods at first
- Encourage rest
- Follow pain-medicine instructions carefully

Call the doctor if:

- Vomiting is **repeated or severe**
- Your child can't keep fluids down
- There are signs of **dehydration** (very little urine, dry mouth, no tears)
- Vomiting lasts **more than 24 hours**

If you'd like, tell me your child's **age** and what kind of **procedure** they're having—I can give more specific guidance and what to expect

Q30.If my child had spinal or epidural anesthesia, could they get a headache afterward?

Answer:

Yes—**it's possible**, though **post-anesthesia headaches are less common in children than in adults**.

Why it can happen

After **spinal or epidural anesthesia**, a headache can occur if a small amount of **cerebrospinal fluid (CSF) leaks** from the puncture site in the spine. This is called a **post-dural puncture headache (PDPH)**.

What it's like

These headaches usually have some characteristic features:

- **Worse when sitting or standing**, better when lying flat
- Pain often in the **front or back of the head**
- May come with **neck stiffness, nausea, dizziness, or light sensitivity**
- Typically starts **within 1–3 days** after the procedure (sometimes up to 5 days)

How common is it in children?

- **Uncommon overall**
- More likely in **older children and adolescents** than in infants or toddlers
- Less common with modern techniques and smaller needles

What helps

Many cases improve on their own with:

- **Rest (lying flat)**
- **Fluids**
- **Pain relievers** recommended by your child's care team

If the headache is **severe, persistent, or clearly positional**, doctors may consider additional treatment (rarely, something called an **epidural blood patch**, which is very effective).

When to call the doctor

Contact your child's healthcare provider if:

- The headache is **strong or worsening**
- It lasts **more than a couple of days**
- Your child has **fever, confusion, vomiting, or trouble walking**
- The headache clearly improves when lying down but returns when upright

If you'd like, tell me **your child's age** and **whether they had a spinal vs. epidural**, and I can give more tailored guidance.

Q31.How soon can my child eat and drink after waking up from general anesthesia?

Answer:

After **general anesthesia**, most children can start eating and drinking **fairly soon**, but it should be done **gradually** to avoid nausea or vomiting.

General timeline (typical guidance)

Within 30–60 minutes after waking up

- Start with **small sips of clear liquids**

    Water

    Pedialyte

    Apple juice

    Ice chips or popsicles

- Only if your child is **fully awake**, able to sit up, and not nauseated.

After clear liquids are tolerated (usually 1–2 hours)

- Move to **light foods**, such as:

    Crackers

    Toast

    Applesauce

    Yogurt

    Rice or plain pasta

Later the same day

- If there's **no vomiting or stomach pain**, your child can usually return to a **normal diet**, avoiding greasy or very spicy foods for the rest of the day.

Important precautions

- Go **slow**—small amounts at first.
- Stop feeding if your child:

Feels nauseated

Vomits

Becomes very sleepy again

- Some children may not feel like eating much for **24 hours**, which is normal.

Follow your provider's instructions

Always follow the **surgeon or anesthesiologist's specific discharge instructions**, especially if:

- The surgery involved the **mouth, throat, or stomach**
- Your child received **opioid pain medication**
- There are conditions like reflux or swallowing problems

When to call the doctor

Contact your healthcare team if your child:

- Keeps vomiting and can't keep fluids down
- Has severe abdominal pain
- Seems unusually difficult to wake or confused hours later

If you'd like, tell me **your child's age** and **what procedure they had**, and I can give more tailored guidance.

Q32.How long will my child need to stay at the hospital after anesthesia?

Answer:

The length of time a child stays in the hospital after anesthesia depends on **the type of anesthesia**, **the procedure**, and **how quickly they recover**, but here are typical expectations:

Common timeframes

- **Outpatient / day surgery (most common):**

Your child usually goes home **1–3 hours after waking up**, once they're alert, drinking fluids, and pain or nausea is controlled.

- **Minor procedures with sedation (not full general anesthesia):**

Often **30 minutes to 2 hours** of observation.

- **Major surgery or complications:**

An **overnight stay or longer** may be required, especially for pain control or monitoring.

What doctors look for before discharge

Your child can usually go home when they:

- Are awake or easily arousable
- Have stable breathing, heart rate, and oxygen levels
- Can drink fluids without vomiting

- Have pain under control
- Have no concerning side effects

Normal after-effects

It's common for children to:

- Be sleepy or groggy for the rest of the day
- Be irritable or emotional
- Have mild nausea or a sore throat (after general anesthesia)

These usually improve within **24 hours**.

Call the doctor or seek care if you notice:

- Trouble breathing or bluish lips
- Persistent vomiting
- Severe pain not relieved by medication
- High fever
- Extreme difficulty waking your child

If you'd like, tell me **your child's age** and **the type of procedure or anesthesia** they're having, and I can give more specific guidance.

Q33.Who can I contact if I have more questions after my child's anesthesia?

Answer:

After your child's anesthesia, you usually have several good options for follow-up questions:

- **The anesthesia team**

Ask for the **anesthesiologist's or nurse anesthetist's contact number** before discharge. Many hospitals provide a direct line or an on-call anesthesia provider.

- **The recovery room (PACU) or day surgery unit**

If you're home and have questions the same day, the **post-anesthesia care unit** or **outpatient surgery center** can often answer common concerns or route you to anesthesia.

- **Your child's surgeon's office**

They can address recovery questions and coordinate with anesthesia if needed.

- **Your child's pediatrician**

Helpful for ongoing symptoms (sleepiness, nausea, behavior changes) in the days after anesthesia.

- **Emergency services**

Call **911 or go to the nearest emergency department** if your child has trouble breathing, bluish lips, severe or worsening vomiting, unresponsiveness, seizures, or any symptom that feels urgent.

If you'd like, tell me whether the procedure was **outpatient or inpatient**, and I can help you decide the *best* first call for your situation.

Model 3

Q1. What are the different types of anesthesia?

Answer:

The main categories of anesthesia are classified based on the area of the body they affect and the level of consciousness they induce.

Here are the four main types:

1.General Anesthesia

- **Effect:** Induces a state of **complete unconsciousness** and loss of sensation across the entire body. The patient is completely unaware and has no memory of the procedure.
- **Administration:** Typically given as a combination of intravenous (IV) medications and/or inhaled anesthetic gases.
- **Use:** Used for major surgeries and procedures where the patient needs to be completely still and pain-free. It often requires assistance with breathing.

2.Regional Anesthesia

- **Effect:** Blocks sensation (pain) in a **larger, specific region** of the body, but the patient remains awake or lightly sedated.
- **Administration:** Local anesthetic (numbing medication) is injected near a cluster of major nerves.
- **Types of Regional Anesthesia:**
    - **Spinal and Epidural Anesthesia (Neuraxial Blockade):** Injections near the spinal cord to numb the lower body (often used for childbirth, or lower abdominal/leg surgery).
    - **Peripheral Nerve Blocks:** Injections to numb a specific limb or area, such as an arm, hand, or leg (used for orthopedic surgeries).

3.Sedation (Monitored Anesthesia Care - MAC)

- **Effect:** Uses medication to make the patient feel **relaxed, drowsy, or sleepy** (often called "twilight sleep"). The level can range from minimal to deep.
- **Administration:** Medications are usually given through an IV.
- **Use:** Used for minor or minimally invasive procedures (like colonoscopies, minor cosmetic surgery) or to supplement regional or local anesthesia to help the patient relax. With minimal or moderate sedation, the patient may still be able to respond to commands.

4.Local Anesthesia

- **Effect:** Causes a **reversible loss of sensation** in a very **small, limited area** of the body. The patient is completely awake and alert.
- **Administration:** Local anesthetic medication (like lidocaine) is injected directly into the tissue at the site of the procedure or applied topically as a cream or spray.
- **Use:** Used for minor procedures, such as stitching a small wound, removing a

mole, or dental work.

The choice of anesthesia depends on the type and length of the procedure, the patient's health, and the anesthesiologist's recommendations.

Would you like to know more about the **medications** used for any of these specific types of anesthesia?

Sources:

https://www.apsf.org/patient-guide/what-are-the-types-of-anesthesia/

https://www.unasourcesurgery.com/types-of-anesthesia/

https://www.hopkinsmedicine.org/health/treatment-tests-and-therapies/types-of-anesthesia-and-your-anesthesiologist

https://www.uclahealth.org/medical-services/anesthesiology/types-anesthesia

https://medlineplus.gov/anesthesia.html

https://madeforthismoment.asahq.org/anesthesia-101/types-of-anesthesia/

https://www.childrensmn.org/educationmaterials/parents/article/10467/types-of-anesthesia/

Q2.What is general anesthesia？

Answer:

General anesthesia is a medically-induced state of controlled unconsciousness that is used for surgical procedures or other medical treatments.

It is a more profound state than mere sleep, as it renders a patient completely unaware, unable to feel pain, and unable to move or react to stimuli.

Key Components of General Anesthesia

The goal of general anesthesia is to achieve several key effects:

- **Unconsciousness:** The patient is in a deep, reversible sleep-like state and is not aware of the procedure.
- **Amnesia:** The patient will not remember the surgery or the events during the procedure.
- **Analgesia (Pain Relief):** The patient does not feel pain.
- **Muscle Relaxation:** Muscles are relaxed, allowing the surgeon to operate safely.
- **Suppression of Reflexes:** Protective reflexes, such as coughing or gagging, are temporarily lost.

How is it Administered?

General anesthesia is typically administered by an anesthesiologist or a certified registered nurse anesthetist (CRNA) using a combination of medications:

- **Induction:** Anesthetic agents are usually given through an **intravenous (IV) line** in your arm or hand, which makes you fall asleep very quickly (within about a minute). Sometimes, especially for children, it may be administered as a **gas inhaled through a mask**.
- **Maintenance:** Once you are unconscious, the anesthesia team continues to administer a precise mix of medicines (IV and/or inhaled gas) to keep you asleep and pain-free for the duration of the procedure.
- **Monitoring and Support:** Throughout the entire procedure, the anesthesia team constantly monitors your vital signs, including **heart rate, blood pressure, breathing, and oxygen levels**. They adjust the medication as needed. A tube is often placed in your throat to ensure proper breathing and oxygen supply while you are unconscious, and this is removed as you wake up.

Common Side Effects

While general anesthesia is generally very safe, minor and temporary side effects are common as the effects wear off:

- **Nausea and Vomiting** (Post-operative nausea and vomiting)
- **Sore throat** (from the breathing tube)
- **Shivering or feeling cold**
- **Temporary confusion or memory loss** upon waking up
- **Muscle aches**

It's important to discuss your full medical history and any concerns with your anesthesia provider before a procedure.

Would you like to know about other types of anesthesia, such as regional or local anesthesia?

Sources:

https://www.bupa.co.uk/health-information/surgery-and-procedures/anaesthesia

https://www.mayoclinic.org/tests-procedures/anesthesia/about/pac-20384568

https://www.webmd.com/a-to-z-guides/what-is-general-anesthesia

https://www.sgu.edu/school-of-medicine/blog/how-does-anesthesia-work/

https://www.nhs.uk/tests-and-treatments/general-anaesthesia/

https://www.ncbi.nlm.nih.gov/books/NBK557596

https://www.gosh.nhs.uk/wards-and-departments/departments/clinical-specialties/anaesthesia-information-parents-and-visitors/side-effects-and-risks-of-a-general-anaesthetic/

https://medschool.ucla.edu/news-article/anesthesia-side-effects

https://madeforthismoment.asahq.org/anesthesia-101/types-of-anesthesia/general-anesthesia/

https://www.nhsinform.scot/tests-and-treatments/medicines-and-medical-aids/types-of-medicine/general-anaesthetic/

Q3.How is anesthesia different for children compared to adults?

Answer:

Anesthesia for children, known as **pediatric anesthesia**, has significant differences compared to adult anesthesia due to children's unique anatomical, physiological, and psychological characteristics.

Here are the key ways pediatric anesthesia differs from adult anesthesia:

1. Physiological and Anatomical Differences

Children are **not simply small adults**, and their developing bodies respond differently to anesthetic agents.

Characteristic	Children (Especially Infants/Young Children)	Adults
**Airway Anatomy**	Relatively larger tongue, shorter neck, and higher/more anterior larynx. Airway is narrowest at the **cricoid cartilage** (unlike vocal cords in adults), making intubation and management more challenging and increasing the risk of obstruction.	Larynx is lower; vocal cords are the narrowest part.
**Oxygen Consumption**	**Significantly higher** metabolic rate and oxygen consumption relative to body weight (almost double an adult's).	Lower metabolic rate and oxygen consumption.
**Respiratory Reserve**	**Lower** Functional Residual Capacity (FRC) and a more compliant chest wall. This, combined with high oxygen consumption, means they **desaturate (oxygen levels drop) much faster** during periods of apnea or hypoventilation.	Higher FRC and more reserve, allowing for longer safe apnea time.
**Cardiovascular System**	**More dependent on heart rate** to maintain cardiac output, as their heart muscle is less contractile. **Bradycardia (slow heart rate) can rapidly lead to severe low blood pressure and cardiac instability.**	Cardiac output is more dependent on stroke volume.
**Temperature Regulation**	**Higher surface area to volume ratio** and less subcutaneous fat, making them highly susceptible to **hypothermia** (low body temperature).	Better ability to regulate body temperature.

**Renal/Hepatic Function**	Immature liver and kidney function, especially in infants. This alters the **pharmacokinetics** (how the body processes drugs), potentially causing some medications (like opioids) to have a longer duration of action.	Mature organ function.

2. Pharmacological Differences (Medication Dosing)

- **Dosing:** Due to differences like a larger volume of distribution and higher metabolic rates, children often require **higher doses of some anesthetic agents on a per-kilogram basis** than adults.
- **Induction: Inhalational induction** (breathing gas through a mask) is often preferred for younger children who are awake to avoid the distress of an intravenous (IV) needle insertion. Adults almost always receive IV induction.
- **Neurotoxicity Concerns:** There is ongoing research and concern regarding the potential long-term neurocognitive effects of anesthetic exposure in very young children (especially under 3 years old) or with multiple, long exposures. This necessitates careful planning to minimize exposure, sometimes using regional anesthesia instead of or in addition to general anesthesia.

3. Psychological and Emotional Factors

- **Anxiety Management:** Preoperative anxiety is a major factor. Pediatric anesthesiologists utilize special strategies, such as:
    - **Age-appropriate language and distraction techniques.**
    - **Premedication** to reduce anxiety before coming to the operating room.
    - Allowing a **parent to be present during induction** (when the child falls asleep) until the child is unconscious.
- **Postoperative Delirium:** Children have a higher risk of **emergence delirium** (being agitated, confused, or uncooperative) upon waking up from anesthesia, which requires specific management.

4. Equipment and Technique

- **Equipment Size:** Everything from monitoring equipment and blood pressure cuffs to breathing tubes and masks must be available in a wide range of pediatric sizes, down to micro-sizes for neonates.
- **Monitoring:** While basic vital signs are monitored, pediatric patients require heightened vigilance, particularly for heart rate and breathing, due to their limited reserves.

The complexity of these differences is why pediatric anesthesia is a recognized specialty, with pediatric anesthesiologists receiving specific advanced training to manage the unique needs of infants, children, and adolescents.

Would you like to know more about the **techniques used to help children feel less anxious** before surgery?

Sources:

https://radiusanesthesia.com/pediatric-vs-adult-anesthesia/

https://www.maskinduction.com/what-makes-pediatric-anesthesia-different.html#

https://www.droracle.ai/articles/607691/what-are-the-anesthesia-considerations-for-pediatric-vs-adult

https://www.hss.edu/health-library/conditions-and-treatments/effects-of-anesthesia-on-children#

https://www.ncbi.nlm.nih.gov/books/NBK559198

https://www.nyp.org/news/anesthesia-for-children-only#

https://pmc.ncbi.nlm.nih.gov/articles/PMC11566558

https://answers.childrenshospital.org/what-parents-should-know-pediatric-anesthesia

Q4. How is general anesthesia different from sedation?

Answer:

General anesthesia and sedation are both medically induced states that use medication to make a patient comfortable during a procedure, but they differ significantly in the **depth of consciousness**, the patient's **ability to respond**, and the **level of medical support** required.

General anesthesia is a state of **controlled, total unconsciousness**, while sedation is a spectrum of **reduced awareness and relaxation**.

Here is a breakdown of the key differences:

Feature	General Anesthesia	Sedation (Minimal to Deep)
**Consciousness**	**Complete loss of consciousness.** The patient is fully "asleep" and unaware.	**Reduced awareness.** Ranges from slightly drowsy but awake (minimal) to very sleepy but rousable (moderate/conscious) or nearly unconscious (deep).
**Response to Stimuli**	**Unarousable.** The patient cannot be woken up and does not respond to pain or verbal commands.	**Responsive.** The patient maintains some ability to respond, ranging from normal response to verbal commands (minimal) to only responding to repeated or painful stimulation (deep).
**Airway/Breathing**	**Often impaired.** The patient's natural breathing is frequently inadequate, and a breathing tube (like an endotracheal tube) and mechanical ventilation are commonly	**Usually maintained.** The patient typically maintains an open airway and can breathe on their own, although extra oxygen may be given and the airway must be closely

	required.	monitored, especially in deep sedation.
**Pain Control**	**Complete pain control** (analgesia) is a core component, in addition to amnesia and unconsciousness.	**Provides relaxation, but pain control is separate.** Often combined with a local or regional anesthetic (numbing the surgical site) to manage pain.
**Procedure Type**	Used for **major, lengthy, or highly invasive** surgeries that require complete immobility.	Used for **minor, short, or less invasive** procedures, such as endoscopies, colonoscopies, or some dental procedures.
**Recovery Time**	Generally **longer**, often requiring several hours of close monitoring, with grogginess lasting up to a day or more.	Generally **quicker**, with patients often able to be discharged within an hour or two after lighter levels.

In short, **general anesthesia** is a controlled medical coma with a loss of all protective reflexes, whereas **sedation** aims to keep the patient calm and comfortable while preserving some level of consciousness and the ability to breathe independently.

Do you have a specific medical procedure in mind that you'd like to know more about the typical type of anesthesia used for it?

Sources:

https://www.dradrianlo.com/blog/general-anesthesia-vs-sedation/

Q5. Is general anesthesia safe for my child?

Answer:

That is a very important and understandable concern. General anesthesia for children is considered **very safe**, and it's safer now than it has ever been due to advances in training, medications, and technology.

However, like any medical procedure, it does carry risks that vary based on the individual child, their health condition, and the specifics of the procedure.

Here is a summary of the key information regarding the safety and risks of general anesthesia for children:

Overall Safety and Common Side Effects

- **High Safety Record:** Anesthesia-related mortality (death) in developed countries is extremely rare, often cited as approximately **1 per 10,000 to 1 per 100,000** or more. The risk of serious injury is also very low.
- **Specialized Care:** Pediatric anesthesiologists are specially trained to manage

a child's unique physiological needs, closely monitoring heart rate, blood pressure, breathing, and oxygen levels throughout the procedure.

- **Common, Temporary Side Effects:** The most common side effects are usually mild and temporary, including:
    - Nausea and vomiting
    - Sore throat (from a breathing tube)
    - Headache or dizziness
    - Drowsiness or grogginess
    - Emergence Delirium (temporary restlessness, crying, or confusion upon waking)

Concern About Brain Development (Children under 3)

The most discussed risk is the potential effect of anesthetic drugs on a child's developing brain, especially in those **under 3 or 4 years old**.

- **Animal Studies:** Studies in young animals have suggested that **repeated or prolonged (long duration) exposure** to general anesthetic and sedation drugs could potentially lead to subtle, long-term problems with learning, memory, or behavior.
- **Human Studies:** Research in humans is less clear and often conflicting.
    - **Single, Short Exposure:** The general consensus from recent, high-quality human studies suggests that a **single, relatively short exposure** to general anesthesia is **unlikely** to cause adverse neurodevelopmental outcomes.
    - **Multiple/Long Exposure:** Some studies suggest that **multiple exposures** or **very lengthy procedures** in children under age three might be associated with a modest decrease in certain abilities, but this is often in children who already have serious underlying health issues that necessitated the surgery.
- **FDA Caution:** The U.S. Food and Drug Administration (FDA) has issued a warning to use caution regarding **repeated or lengthy use (more than 3 hours)** of these drugs in children under three.
- **Professional Guidance:** For most children, the risk of delaying a **necessary** surgery is far greater than the risk from the anesthesia. For **elective** procedures (not mandatory for health), doctors may recommend delaying them until after the child's third birthday, if safely possible.

What You Should Discuss with Your Child's Healthcare Team

The best way to ensure your child's safety is to have an in-depth conversation with their **pediatric anesthesiologist** and **surgeon**. You should ask:

- **Is the procedure absolutely necessary?** (Especially if the child is under 3).
- **Can the procedure be delayed?** If waiting carries no risk to your child's health.
- **What are the specific risks** for *my* child, given their age and medical condition? (Risk is higher for infants/neonates and children with severe underlying health issues).
- **Can regional anesthesia** (like a nerve block or spinal block) be used, or used

in addition to lighter sedation, to reduce the general anesthesia exposure?

- **Who will be administering the anesthesia?** (A pediatric anesthesiologist is recommended).

In short: General anesthesia is very safe, but the decision should be made in consultation with your child's doctors, carefully weighing the necessity and timing of the procedure against the small, known, and potential risks.

Would you like me to look up information on **regional anesthesia options** that may be used in place of or in combination with general anesthesia for children?

Sources:

https://med.stanford.edu/pedsanesthesia/patients/brain-dev-faqs.html#

https://www.singhealth.com.sg/symptoms-treatments/patient-information-general-anaesthesia#

https://answers.childrenshospital.org/what-parents-should-know-pediatric-anesthesia/

https://myhealth.alberta.ca/Health/aftercareinformation/pages/conditions.aspx?hwid=acm5237

https://www.healthdirect.gov.au/surgery/general-anaesthetic-child

https://www.frontiersin.org/journals/molecular-neuroscience/articles/

10.3389/fnmol.2022.972025/full

https://www.uclahealth.org/medical-services/anesthesiology/anesthesia-and-child-development#

https://www.kkh.com.sg/patient-care/areas-of-care/childrens-services/general-anaesthesia---possible-effects-on-children-s-brain-devel#

https://www.apsf.org/article/the-effect-of-general-anesthesia-on-the-developing-brain-is-it-time-to-temper-the-concern

https://www.hss.edu/health-library/conditions-and-treatments/effects-of-anesthesia-on-children#

https://health.clevelandclinic.org/is-anesthesia-safe-for-kids#

https://med.stanford.edu/pedsanesthesia/patients/brain-dev-faqs.html#

https://myhealth.alberta.ca/Health/aftercareinformation/pages/conditions.aspx?hwid=acm5237#

Q6.anesthesia harm my child's brain development?

Answer:

This is a very important and understandable concern that many parents have. The short answer is that the relationship between anesthesia and a child's brain development is a topic of ongoing research, and there are some nuanced findings you should discuss with your child's doctors.

Here is a summary of the current understanding based on research:

General Consensus

- **Single, Short Exposure is Generally Considered Safe:** Multiple large-scale clinical studies (like the PANDA, GAS, and MASK trials) have found that a **single, brief exposure** to general anesthesia in young children (even those under 3 or 4) is generally **not associated with significant, long-term adverse neurodevelopmental outcomes** like major differences in overall IQ, memory, or behavior.
- **The Risk Increases with Duration and Frequency:** The primary concern, and the focus of a 2017 **U.S. Food and Drug Administration (FDA) warning**, is related to:
    - **Multiple exposures** to general anesthesia.
    - Anesthesia exposure lasting **longer than 3 hours** in children, particularly those **younger than 3 years old**.
- **The Underlying Condition is a Factor:** Children who require multiple surgeries or longer procedures often have underlying medical conditions that could, themselves, affect brain development. It can be difficult for researchers to separate the effect of the anesthesia from the effect of the underlying condition or the stress of the illness/surgery.

Specific Findings

- **Potential Subtle Effects:** Some studies have suggested that multiple or prolonged exposures might be associated with a **modest decrease in fine motor abilities, processing speeds, or minor difficulties with reading or behavior** at school age, but the overall evidence is conflicting and not conclusive.
- **Pain and Stress are also Harmful:** Experts stress that **untreated pain and the extreme stress of undergoing a procedure while awake are also known to be harmful** to a child's developing nervous system. Anesthesia is often a necessity to safely perform a life-saving or essential procedure.
- **Regional Anesthesia:** For some procedures, a doctor may be able to use a regional block (like spinal or epidural anesthesia) instead of general anesthesia, which may help decrease general anesthetic exposure. These alternatives have not been shown to harm brain development.

What You Should Discuss with Your Child's Healthcare Team

If your child needs a procedure requiring anesthesia, it is essential to have an open conversation with the **surgeon** and the **pediatric anesthesiologist**. Key questions to ask include:

- **Is general anesthesia necessary, or can a regional/local anesthetic be used?**
- **How long is the surgery expected to last?**
- **Are additional surgeries anticipated in the future?**
- **Can the surgery be safely delayed** until my child is older (e.g., over 3 or 4 years old), or is it urgent?

The doctors can then balance the risks of the anesthesia against the risks of delaying the necessary procedure or allowing the child to experience untreated pain.

Would you like me to look for more information on the **alternatives** to general anesthesia, such as regional anesthesia, for pediatric procedures?

Sources:

https://www.hss.edu/health-library/conditions-and-treatments/effects-of-anesthesia-on-children#

https://pmc.ncbi.nlm.nih.gov/articles/PMC11005881/#

https://kidshealth.org/en/parents/anesthesia-brain.html#

https://med.stanford.edu/pedsanesthesia/patients/anes-child-brain.html#

https://www.kkh.com.sg/patient-care/areas-of-care/childrens-services/general-anaesthesia---possible-effects-on-children-s-brain-devel#

Q7.Is general anesthesia necessary for my child's dental treatment?

Answer:

That is a very important question, and the decision about general anesthesia (GA) for a child's dental treatment is made on a case-by-case basis by the pediatric dentist, often in consultation with you and potentially an anesthesiologist.

General anesthesia is **not necessary** for most routine dental treatments in children. It is typically recommended when alternatives are not suitable, which includes situations like:

When General Anesthesia is Usually Recommended

- **Extensive or Complex Treatment:** If your child needs a large number of fillings, crowns, or extractions that would take a long time to complete, GA allows all necessary work to be done safely and efficiently in one visit.
- **Age and Cooperation:** Very young children (like toddlers and infants) or those who are unable to follow instructions and stay still due to their developmental stage.
- **High Anxiety or Fear:** Children with extreme dental phobia or anxiety who are unable to tolerate treatment even with milder sedation options.
- **Special Healthcare Needs:** Children with physical, developmental, or behavioral conditions that make it difficult or unsafe for them to cooperate during treatment.
- **Failed Sedation Attempts**: If milder sedation methods (like laughing gas or oral sedation) have not been effective in managing your child's behavior and anxiety.

Alternatives to General Anesthesia

Pediatric dentists prioritize the least invasive method possible. Alternatives that may be considered include:

Method	Description	Child's Awareness
**Nitrous Oxide**	"Laughing Gas" inhaled through a nose mask. It is a mild sedative that reduces	**Awake and Responsive**

	anxiety and makes the child relaxed and happy.	(Most mild)
**Oral Sedation**	Medication (liquid syrup or pill) taken by mouth. It helps the child feel drowsy, relaxed, and more willing to cooperate.	**Awake and Drowsy** (Minimal to Moderate)
**Local Anesthesia**	A numbing injection used to eliminate pain in the specific area being treated (often used in combination with the above sedation methods).	**Fully Awake** (Used for pain control)
**IV Sedation**	Medication delivered through an intravenous line, resulting in a deeper sleep than oral sedation but not as deep as GA.	**Deeply Sedated**

Ultimately, the best approach depends on your child's specific **medical history, level of anxiety, and the extent of the dental work required.**

I highly recommend having an in-depth discussion with your child's **pediatric dentist**. They can explain the specific reasons for their recommendation, discuss the risks and benefits of all options (including GA), and help you make an informed decision that is safest and most comfortable for your child.

Would you like me to look up information about the **risks and preparation involved with general anesthesia** for children's dental work?

Sources:

https://brisbane-paediatric-dentist.au/general-anaesthesia-for-childrens-dental-care

https://drfatemehmobaraki.com/the-risks-and-benefits-of-general-anesthesia-in-pediatric-dentistry

Q8.Who will give my child anesthesia?

Answer:

That's a very important question. For a child's procedure, the anesthesia is given and monitored by a specialized team, always supervised by a doctor who is an **Anesthesiologist**, and most often, a **Pediatric Anesthesiologist**.

Here is a breakdown of the team members who might be involved:

The Anesthesia Care Team

- **Pediatric Anesthesiologist:** This is a doctor who has completed an anesthesiology residency and then had additional, specialized training (a fellowship) specifically in providing anesthesia to infants, children, and adolescents. They are responsible for your child's entire anesthesia plan, safety, and comfort.
- **Certified Registered Nurse Anesthetist (CRNA):** An advanced practice registered nurse who is board-certified and has specialized training in anesthesia.

They often work closely under the direct supervision of the Anesthesiologist.

- **Anesthesiologist Assistant (AA):** A skilled non-physician provider who works under the direction of an Anesthesiologist as part of the anesthesia care team.
- **Anesthesia Resident or Fellow:** These are doctors who are still in training. A **Resident** is completing their general anesthesiology training, and a **Fellow** is a fully trained anesthesiologist doing a subspecialty like pediatric anesthesia. Both are closely supervised by the attending Pediatric Anesthesiologist.

The **Pediatric Anesthesiologist** is ultimately in charge. They are specially qualified to:

- Evaluate your child's complex medical history and determine the safest type and dosage of anesthesia.
- Use equipment specifically designed for children.
- Monitor your child's vital signs (breathing, heart rate, blood pressure) moment-by-moment during the entire procedure.

You will typically have a chance to meet with the Pediatric Anesthesiologist or another member of the team on the day of the procedure or during a pre-operative consultation to discuss the plan and ask any questions you have.

Would you like me to find out more about the specific types of anesthesia that are used for children?

Sources:

https://www.med.unc.edu/surgery/pedssurgery/forpatients/faqs/anesthesia/

https://www.luriechildrens.org/en/specialties-conditions/pediatric-anesthesia/

https://www.anzca.edu.au/patient-information/about-anaesthesia/anaesthesia-and-children#

https://www.aegisanesthesiapartners.com/patient-information/faq-frequently-asked-questions-pediatric-anesthesia/

https://www.childrenscolorado.org/doctors-and-departments/departments/anesthesiology/

https://www.chop.edu/patients-and-visitors/guide-your-childs-surgery/day-your-childs-surgery/about-anesthesia#

https://www.healthychildren.org/English/family-life/health-management/pediatric-specialists/Pages/What-is-a-Pediatric-Anesthesiologist.aspx#

https://answers.childrenshospital.org/what-parents-should-know-pediatric-anesthesia/

Q9. What if my child won't cooperate for anesthesia?

Answer:

This is a very common and understandable concern for parents. If a child is uncooperative for anesthesia induction, the healthcare team has several established strategies—both psychological and pharmacological—to ensure the procedure can be performed safely and smoothly.

The approach is highly individualized, depending on the child's age, level of anxiety, medical history, and temperament.

Here is an overview of the main strategies used:

1. Psychological and Behavioral Techniques

These techniques aim to reduce anxiety and build trust *before* and *during* the induction process.

- **Pre-Hospital Preparation:**
  - **Honest, Age-Appropriate Explanation:** Giving simple, honest explanations of the procedure is crucial. For children under six, explanations are often given right before the procedure; for older children, two to three days in advance is better. Words like "magic air" or "sleepy juice" are often used instead of "gas" or "needle."
  - **Hospital Tours/Play Therapy:** Some facilities offer tours or play therapy where the child can interact with equipment (like the mask or IV line) in a non-threatening, play-based way.
- **Distraction and Comfort:**
  - **Use of Comfort Items:** Allowing the child to bring a favorite blanket, stuffed animal, or toy.
  - **Distraction at Induction:** Techniques are tailored by age:
    - **Infants:** Rocking, gentle patting, singing, or using sucrose solution on a pacifier.
    - **Toddlers/Preschoolers:** Blowing bubbles (sometimes using the anesthesia mask to blow them), using a pinwheel, or focusing on an interactive toy or video.
    - **Older Children:** Watching a favorite show/game on a tablet, listening to music with headphones, or using deep breathing/imagery techniques (e.g., imagining a favorite place).
  - **Parental Presence:** In many cases, a parent or support person is allowed to stay with the child during the initial moments of anesthesia induction to provide comfort, which can significantly ease the child's fear of separation.
- **Giving the Child Control:**
  - Offering small choices can give the child a sense of control, such as: "Do you want to walk or ride on the trolley?" or "Do you want the **strawberry** or **chocolate** scented mask?"

2. Pharmacological Premedication (Sedatives)

If behavioral techniques alone are not sufficient, or if the child is known to be highly anxious, a sedative medication (premedication) may be given *before* entering the operating room. The goal is to make the child relaxed and sleepy, facilitating a calm induction.

Commonly used premedication drugs include:

- **Midazolam:** A common agent that reduces anxiety and often provides amnesia, so the child won't remember the induction. It can be given orally (often

mixed with juice), nasally, or rectally.

- **Alpha-2 Agonists (e.g., Clonidine or Dexmedetomidine):** These provide sedation and are increasingly used as they also offer benefits like pain reduction and reduced post-anesthesia agitation. They are often given orally or nasally.
- **Ketamine:** In some cases, especially for older children who are extremely combative or who have developmental or behavioral disorders, a dose of oral or intramuscular ketamine may be used to achieve sedation quickly.

What to Discuss with Your Anesthesiologist

The most important step is to **have an open and detailed discussion with the pediatric anesthesiologist** well before the procedure.

You should discuss:

- **Your child's temperament** and what has worked (or not worked) in previous medical settings.
- **The plan for premedication** (if any) and the route of administration.
- **The option of parental presence** during induction (this varies by hospital policy and the anesthesiologist's discretion).
- **Which distraction tools** you should bring (e.g., a tablet, book, or toy).

They are highly trained in managing difficult and anxious pediatric inductions and will have a backup plan ready if the first strategy doesn't work.

Would you like me to look up information on **preparing your child** for an upcoming surgery or procedure?

Sources:

https://cliftonandmauney.com/blog/how-to-manage-anxiety-in-children-undergoing-anesthesia#

https://clinicalview.gehealthcare.com/article/pediatric-anesthesia-induction-debate-over-techniques-and-how-keep-little-patients-calm#

https://epickids.org.au/how-to-talk-to-kids-about-their-operation/#

https://www.rch.org.au/kidsinfo/fact_sheets/Reducing_your_childs_discomfort_during_procedures/

https://cliftonandmauney.com/blog/how-to-manage-anxiety-in-children-undergoing-anesthesia#

https://www.littlepeoplescove.com/blog/helping-kids-prepare-for-surgery-calming-activities-every-parent-should-know#

https://pmc.ncbi.nlm.nih.gov/articles/PMC6761781/

Q10. What if my child is allergic to anesthesia?

Answer:

This is a very important and serious question that any parent should discuss in depth

with their child's healthcare team, especially a **pediatric anesthesiologist**.

Allergic reactions to anesthesia are rare, but they can be severe. The good news is that medical teams are highly trained to recognize and manage these reactions, and there are many alternative medications and protocols available.

Here is a breakdown of what you should know and what steps the medical team will likely take:

1. Immediate Steps and Consultation

- **Communicate Everything:** Before any procedure, you *must* inform the doctor, surgeon, and especially the **anesthesiologist** about your child's suspected or confirmed allergy to any medication, including anesthesia, antibiotics, or even **latex** (a common culprit for perioperative allergies).
- **Detailed History:** Be prepared to provide as much detail as possible about the reaction:
    - What were the **symptoms**? (e.g., rash, hives, difficulty breathing, swelling, low blood pressure).
    - When exactly did the reaction occur? (Immediate, or delayed for hours/days?)
    - Which medications were given at that time?
- **Referral to an Allergist/Immunologist:** If there is a clear history of an allergic reaction to an anesthetic agent, the medical team will likely refer your child to an allergist. This specialist can perform tests (like **skin prick tests** or **blood tests**) to try and pinpoint the exact triggering agent.

2. Planning for a Safe Anesthetic

If an allergy is confirmed or strongly suspected, the anesthesiologist will create a highly customized and safe plan, often involving a **pediatric anesthesiologist** who has additional training for children.

- **Avoidance of Known Triggers:** The primary strategy is to **completely avoid** the specific drug or drug class that caused the reaction. Since many anesthetic drugs belong to different chemical groups, there are almost always **safe alternatives** available.
- **Alternative Anesthesia Techniques:** The team may opt for a different type of anesthesia:
    - **Regional Anesthesia:** Numbing only a specific part of the body (e.g., an epidural or a nerve block) can sometimes be used alone or in combination with very mild sedation, avoiding the need for a full general anesthetic.
    - **Alternative General Anesthetic Agents:** Some inhaled anesthetic agents (like **Sevoflurane**) are less commonly associated with allergic reactions than others. Certain intravenous agents, like **Ketamine** or **Etomidate**, can also be good options for children with a history of unknown allergies.
- **Latex-Safe Protocol:** Since **latex** is a common perioperative allergen, a "latex-free" environment will be used if your child has a known or suspected latex allergy
- **Close Monitoring:** During the procedure, your child will be monitored *continuously* and meticulously for any signs of an adverse reaction, with all necessary

emergency medications (like **adrenaline/epinephrine**) immediately on hand.

3. Key Triggers to Discuss

Allergies during surgery are often triggered by medications other than the primary anesthetic gas/IV drug. Be sure to discuss allergies to:

- **Neuromuscular Blocking Agents (NMBAs) or Muscle Relaxants:** These are the most common cause of true allergic reactions during general anesthesia.
- **Antibiotics:** Often given before or during surgery to prevent infection.
- **Latex:** Found in some gloves, tubing, and medical devices.
- **Chlorhexidine:** An antiseptic skin cleanser.
- **Food Allergies:** Some IV agents (like **Propofol**) contain components derived from eggs or soy, though true allergic cross-reactivity is rare and debated. You must still inform the team of any food allergies.

The most important takeaway is to be completely honest and thorough with your child's medical team. Their safety is the number one priority, and the anesthesiologist is the specialist who is prepared to manage this specific risk.

Would you like me to look up the typical **signs of an allergic reaction (anaphylaxis) in a child** to help you understand what to watch for?

Sources:

https://pmc.ncbi.nlm.nih.gov/articles/PMC12419412/

https://www.cas.ca/en/about-cas/advocacy/anesthesia-faq/allergic-reactions-during-surgery

https://www.apsf.org/patient-guide/can-i-be-allergic-to-anesthesia/

https://www.aegisanesthesiapartners.com/anesthesia-library/anaphylaxis-anesthesia/

https://www.medicalnewstoday.com/articles/anesthesia-allergy

https://pmc.ncbi.nlm.nih.gov/articles/PMC6137173/

Q11. Will my child need any evaluation before anesthesia?

Answer:

That's a very important question. **Yes, your child will absolutely need an evaluation before receiving anesthesia** (often called a pre-anesthesia or pre-operative assessment).

The purpose of this evaluation is to create the safest possible anesthesia plan for your child by identifying any potential risks and optimizing their health beforehand.

Here is a breakdown of what that evaluation typically involves, including information on testing:

Components of the Pre-Anesthesia Evaluation

This assessment is usually done by a member of the anesthesia care team (anesthesiologist or nurse practitioner/nurse specialist) and may be done in person, over the phone, or virtually.

1. Detailed Medical History (Most Critical Step)

The team will ask you extensive questions about your child's health, including:

- **Birth History:** Especially if your child was a former preterm infant or had complications.
- **Previous Anesthesia/Surgery:** Any complications with a previous procedure, such as difficult intubation, airway problems, nausea/vomiting, or fever.
- **Family History:** Any family history of problems with anesthesia, such as **Malignant Hyperthermia** or bleeding disorders.
- **Current/Chronic Conditions:** Conditions like asthma (is it controlled?), heart problems (heart murmurs, congenital defects), seizures, or diabetes.
- **Recent Illness:** Especially recent colds, fevers, or coughs, as these can increase the risk of breathing problems during or after anesthesia.
- **Medications & Allergies:** A complete list of all prescribed medications, over-the-counter drugs, vitamins, and herbal supplements. They need to know all allergies (drug, food, latex).

2. Physical Examination

The anesthesia provider will conduct a physical exam, focusing on:

- **Airway:** Looking for any airway anomalies or features that might make placing a breathing tube or mask difficult.
- **Heart and Lungs:** Assessing their overall cardiac and respiratory status.

3. Pre-Operative Testing (Lab Work/Imaging)

For healthy children undergoing low-risk, elective procedures, routine lab testing is often NOT required.

However, specific tests may be ordered if your child has an underlying medical condition or if the planned surgery carries a high risk of complications or blood loss. These tests might include:

- **Blood Tests:**
  - **Complete Blood Count (CBC):** To check for anemia (low red blood cells).
  - **Clotting Studies:** If there's a risk of significant bleeding or a history of bleeding disorders.
  - **Group & Screen:** To determine blood type and check for antibodies, in case a blood transfusion is needed.
- **Other Tests:**
  - **X-rays or EKG (Electrocardiogram):** May be needed if your child has a heart or lung condition.
- **Pregnancy Test:** Typically required for female patients who have started menstruating (usually 12 years and older), as a standard safety measure before any anesthesia.

Fasting Guidelines

Part of the pre-anesthesia preparation includes strict instructions on when your child must **stop eating and drinking (fasting/NPO)** to prevent the serious complication of aspirating stomach contents into the lungs during anesthesia.

You will be given specific times to follow, which are generally based on the type of food or liquid. A common, safe approach follows a timeframe like:

Substance	Minimum Fasting Time
**Clear Liquids** (water, apple juice, Pedialyte)	2 hours
**Breast Milk**	4 hours
**Infant Formula, Non-Human Milk**	6 hours
**Solid Food** (including chewing gum, candy, cereal)	6 to 8 hours

It is crucial to follow the exact instructions provided by your child's surgical team, as failure to do so may result in the procedure being delayed or canceled for safety reasons.

The most important takeaway is to be completely honest and thorough when providing your child's medical history. This information guides the team in providing the safest experience.

Would you like me to look up the typical fasting guidelines for a specific age group, or do you have any other questions about the preparation?

Sources:

https://www.healthychildren.org/English/health-issues/conditions/treatments/Pages/Procedures-With-Anesthesia.aspx

https://www.aboutkidshealth.ca/why-are-pre-anesthesia-assessments-helpful

https://www.sickkids.ca/en/care-services/clinics/pre-anesthesia-clinic/

https://www.aafp.org/pubs/afp/issues/2022/0600/p640.html

https://publications.aap.org/pediatrics/article/134/3/634/74202/The-Pediatrician-s-Role-in-the-Evaluation-and?autologincheck=redirected

https://kidshealth.org/en/parents/anesthesia.html

https://www.maskinduction.com/npo-guidelines.html

Q12. Can my child have anesthesia with a cold?

Answer:

That is a very important question, and the decision to proceed with anesthesia when a child has a cold involves careful consideration of risks versus benefits, and it is ultimately made by the anesthesiologist and surgeon.

Here is a summary of the key information and general guidelines:

Increased Risks with a Cold

A cold, or an Upper Respiratory Tract Infection (URTI), can increase the risk of respiratory complications during and after anesthesia, especially in children. These

complications can include:

- **Laryngospasm or Bronchospasm:** Spasms of the voice box or airways, which can make breathing difficult.
- **Increased Secretions:** More mucus can make it harder for the child to breathe.
- **Desaturation:** A drop in the oxygen level in the blood.
- **Post-operative Complications:** A higher risk of developing a chest infection or having a prolonged recovery.

When It Might Be Safe to Proceed (Mild Symptoms)

The surgery may often **proceed** if the child has **mild, uncomplicated symptoms** and appears otherwise healthy. This usually means:

- **Clear nasal discharge (runny nose)** only.
- **No fever** (usually defined as under 100^{\circ}\text{F} or 38^{\circ}\text{C}).
- **No deep cough, wheezing, or difficulty breathing.**
- **The child is active, alert, and has a good appetite.**
- The surgery is **minor** and **does not involve the airway** (e.g., tonsillectomy)**.**.

When Surgery Is Likely Postponed (Moderate to Severe Symptoms)

An elective (non-emergency) surgery will often be **postponed** if the child has more severe symptoms or specific risk factors. These include:

- **Fever** (over 100^{\circ}\text{F} or 38^{\circ}\text{C}).
- **Thick, yellow, or green secretions/phlegm.**
- **Productive or wet cough** or **wheezing**.
- **Appearing sick, lethargic, or refusing food.**
- **History of asthma** or other reactive airway diseases.
- **The child is under 1 year of age** (especially infants and ex-premature babies).
- The surgery is a **major procedure** or **involves the airway**.

Timing for Rescheduling

If the surgery is postponed, the medical team will usually recommend waiting for a specific period to allow the child's airways to recover completely, as the airways can remain sensitive even after the symptoms disappear:

- **Mild Cold:** Often postponed for about **2 weeks** after all symptoms have resolved.
- **Severe Cold/Respiratory Infection (like the flu, RSV, or with a high fever):** May be postponed for **4 or more weeks**.

What You Should Do

You must call your child's surgeon's office or the hospital's pre-operative assessment clinic immediately if your child develops a cold, cough, or fever in the days or weeks leading up to the procedure.

Your child's **pediatric anesthesiologist** will perform an individual risk-benefit

assessment on the day of surgery to determine if it is safe to proceed. They are the expert best positioned to make this critical decision.

Would you like me to find general information on how to prepare a child for an upcoming surgery to help ease any anxiety?

Sources:

https://www.nysora.com/education-news/managing-pediatric-anesthesia-for-children-with-upper-respiratory-tract-infections-urti/

https://pmc.ncbi.nlm.nih.gov/articles/PMC12567271/

http://www2.pedsanesthesia.org/patiented/uri.iphtml#

https://asa.org.au/preparing-your-child

https://www.hss.edu/health-library/conditions-and-treatments/why-do-anesthesiologists-care-about-a-common-cold

Q13. When does my child need to stop eating or drinking before anesthesia?

Answer:

This is a very important safety question. Fasting times for children before anesthesia are set to prevent the serious complication of aspirating (breathing in) stomach contents into the lungs.

The absolute best guidance will come directly from your child's medical team (anesthesiologist, surgeon, or nurse), who will give you specific, timed instructions based on the procedure, your child's age, and their medical history.

However, standard, common guidelines used by many pediatric hospitals and anesthesia societies generally follow these minimum fasting periods:

Item	Minimum Fasting Time Before Anesthesia
**Solid Food or Non-Human Milk** (including formula, cow's milk, dairy, nut milk, fried foods, and fatty foods)	**6 hours**
**Breast Milk**	**4 hours**
**Clear Liquids** (water, clear apple juice, Pedialyte, clear sports drinks, and clear broth—*no pulp, no milk/dairy, not fizzy/carbonated*)	**2 hours**

Key Considerations

- **Breast Milk vs. Formula:** Breast milk is generally allowed closer to the procedure (4 hours) than formula or solid food (6 hours) because it is digested faster.
- **Clear Liquids:** The goal is to allow clear liquids as close to the surgery as safely possible (often 2 hours) to keep the child comfortable and hydrated. Some modern guidelines, in select cases and centers, may allow clear fluids up to **1 hour**

before the procedure, especially in an effort to reduce distress from prolonged fasting, but this varies. **Always confirm the liquid cutoff time with your hospital.**

- **Medical Exceptions:** Children with certain conditions, like delayed stomach emptying, gastroesophageal reflux, or diabetes, may require *longer* fasting times or special instructions.
- **Medications:** Unless told otherwise by the anesthesiologist, regular medications can usually be taken with a small sip of clear fluid.
- **Cancellation Risk:** If your child consumes anything past the instructed cutoff time, the surgery will almost certainly be **delayed or cancelled** for safety reasons.

What you should do now:

- **Wait for the specific instructions** from the hospital or surgical center, as they will provide precise cut-off times tied to your child's scheduled procedure time.
- **Strictly follow those specific instructions** to ensure your child's safety and prevent delays.

Would you like me to look up contact information for a specific hospital or clinic to check their fasting guidelines, if you know where the procedure is scheduled?

Sources:

https://www.aboutkidshealth.ca/what-are-the-fasting-guidelines-for-your-child-before-surgery

https://www.openanesthesia.org/keywords/npo-guidelines-for-infants-and-children/

https://anaesthesia-singapore.org.sg/preop-fasting-guidelines/

https://www.starship.org.nz/guidelines/anaesthesia-fasting-guideline

https://www.hopkinsmedicine.org/johns-hopkins-howard-county/services/surgery/pediatric-anesthesia

https://www.childrens.health.qld.gov.au/health-a-to-z/preparing-for-surgery-fasting

https://www.maskinduction.com/npo-guidelines.html

Q14. If my child is breastfeeding, do we need to stop before their anesthesia?

Answer:

That is an excellent and very important question to ask your child's medical team.

For a child who is breastfeeding and needs to undergo anesthesia, here are the general guidelines for preoperative fasting. **It is crucial to follow the specific instructions given by your child's anesthetist or surgeon, as times can vary based on the child's age, health status, and the procedure.**

Fasting Guidelines for Breast Milk

Due to the quick digestion of breast milk, the fasting time for breastfed infants is typically shorter than for those on formula or solid food.

- **Breast Milk:** Most major guidelines (like those from the American Society of Anesthesiologists and various European societies) recommend that a baby can have **breast milk up to 3 or 4 hours** before the scheduled time of anesthesia.

- *The Academy of Breastfeeding Medicine and the American Society of Anesthesiologists often recommend 4 hours.*
- *Newer European guidelines have sometimes reduced this to 3 hours.*

- **Clear Fluids:** Water or clear juice (without pulp) is generally allowed up to **1 to 2 hours** before anesthesia.
- **Formula Milk and Solids:** These typically require a longer fasting time, often **6 hours** before anesthesia.

The goal is to minimize the amount of stomach contents to prevent the serious risk of aspiration (inhaling food or liquid into the lungs) while the child is unconscious.

Key Steps to Take

- **Consult the Anaesthetist: Always** confirm the exact fasting instructions (NPO, or "nothing by mouth," times) directly with the medical team managing your child's care, especially the anaesthetist. They will provide the official, final timing for your specific situation.
- **Time the Last Feed:** Try to time a breastfeed as close to the approved fasting cutoff time as possible. This helps ensure your baby is comfortable and well-hydrated for the maximum duration possible.
- **Waking the Child:** For infants, the team may advise waking the child to feed four hours before the scheduled procedure, even at night, to help prevent low blood sugar (hypoglycemia) and dehydration.

Please speak with your child's anaesthetist today to get their precise, individual plan.

Would you like me to look up information on **breastfeeding safety for a mother who is having anesthesia** for her own procedure? (This is a separate scenario, but also a common question.)

Sources:

https://breastfeeding.support/fasting-for-surgery/

https://www.openanesthesia.org/keywords/npo-guidelines-for-infants-and-children/

https://www.schn.health.nsw.gov.au/surgery-and-anaesthesia-handbook/preparing/fasting

Q15. What should I do if my child takes daily medicines before anesthesia?

Answer:

This is a very important question, and the single most crucial piece of advice is to **always follow the specific instructions provided by your child's surgeon and anesthesiologist.**

Medication management before anesthesia is highly individualized and depends on:

- The **type** of surgery/procedure.
- The **type** of daily medicine your child is taking (e.g., heart, seizure, asthma, blood thinners).

- Your child's **overall medical condition** and health history.

The Most Important Steps

- **Communicate All Medications:**
  - **Create a complete list** of **all** medications your child takes daily, including prescription drugs, over-the-counter (OTC) medicines (like Tylenol/acetaminophen or Motrin/ibuprofen), vitamins, herbal supplements, and dietary supplements**.**
  - Include the **name**, **dose**, and **last time** the dose was taken.
  - **Bring the original containers** of all medications with you on the day of the procedure.
- **Get Specific Instructions:**
  - The surgical team (especially the **anesthesiologist**) will provide clear, written instructions on which medications to **continue** and which to **stop** or **adjust**.
  - **Do not stop any prescription medication** without explicit instruction from the doctor who is managing the procedure. Stopping certain medications suddenly (like some heart or seizure medications) can be very dangerous.
- **Ask Targeted Questions:**
  - Ask specifically about the medication schedule for the **day before** the procedure and the **morning of** the procedure.
  - Clarify if they can take the medicine with a **sip of clear liquid** on the day of surgery (many essential medicines are permitted with a small sip of water, even while fasting).

General Guidelines (To Discuss with Your Doctor)

While you must consult the care team for personalized advice, there are general patterns for certain drug classes:

Medication Class	General Guideline (Confirm with Doctor)
**Blood Thinners (e.g., Aspirin, NSAIDs)**	**Often need to be stopped** several days (sometimes up to two weeks) before surgery to reduce the risk of bleeding. This includes non-steroidal anti-inflammatory drugs (NSAIDs) like ibuprofen (Motrin/Advil) and naproxen (Aleve).
**Essential Daily Medications**	Medications for chronic conditions like **asthma, seizures, heart conditions, thyroid issues (levothyroxine), and corticosteroids** are often **continued** right up until the morning of surgery with a tiny sip of water, as stopping them can be risky.
**Diabetes Medications (Insulin, Oral Agents)**	**Always require adjustment.** Doses are typically reduced or withheld the night before and the morning of surgery to prevent dangerously low

	blood sugar (hypoglycemia) during fasting.
**Herbal/Dietary Supplements**	**Almost always stopped** for one to two weeks before surgery, as they can interfere with blood clotting or interact with anesthetic drugs.

Note on Fasting

Even if a medicine is allowed on the morning of surgery, it must be taken with a **tiny sip of clear liquid** (water, clear apple juice, etc.) and before the absolute cutoff time for liquids. Fasting from solids and liquids is critical to prevent the aspiration of stomach contents into the lungs under anesthesia, which can be fatal.

Please contact the anesthesiology department or the surgeon's office today to review your child's specific medication list.

Do you have the name of the medicine your child takes, or would you like me to look up information on **pediatric fasting guidelines** (clear liquids, formula, solids) before anesthesia?

Sources:

https://www.sickkids.ca/en/patients-visitors/planning-visit/coming-for-surgery/days-before-surgery-important-instructions/

https://www.rch.org.au/kidsinfo/fact_sheets/Preparing_your_child_for_surgery_general_anaesthesia/

Q16. If my child has asthma, diabetes, or other health issues, do I need to do anything special before anesthesia?

Answer:

That's an excellent and crucial question. Yes, if your child has asthma, diabetes, or any other chronic health condition, you will need to take **special precautions and work closely with their entire healthcare team** before they receive anesthesia.

The primary goal is to **optimize their health condition** before the procedure to minimize risks.

Here is a summary of the special steps generally recommended for children with asthma and diabetes:

General Steps for Any Chronic Condition

Regardless of the specific condition, the most important steps are:

1. **Thorough Pre-Anesthesia Assessment:** Your child will have a consultation with a pediatric anesthesiologist or a pre-operative team well in advance of the procedure. Be prepared to discuss:

- **Detailed Medical History:** Include all diagnoses, past surgeries, and any previous issues with anesthesia.
- **Complete Medication List:** Provide *all* prescription, over-the-counter, vitamin, and herbal supplements. Some may need to be adjusted or stopped.

- **Specialist Coordination:** The anesthesia team will often coordinate with your child's endocrinologist (for diabetes), pulmonologist (for asthma), or other specialists for an individualized plan.

2.**Strict Fasting Guidelines:** Follow the specific instructions for when your child must stop eating and drinking, as these rules are critical for safety under anesthesia.

Special Steps for Asthma

Children with asthma are at a higher risk for respiratory complications (like bronchospasm or wheezing) during or after anesthesia.

- **Optimize Asthma Control:** It is highly recommended to ensure your child's asthma is **as well-controlled as possible** before the procedure.
  - You may be advised to consult your pediatrician or pulmonologist several weeks in advance.
  - If your child has had recent symptoms or a history of frequent exacerbations, the surgery may need to be postponed until their symptoms improve.
- **Medication Adjustments:**
  - **Continue Routine Medicines:** Your child should usually **continue their inhaled corticosteroids (ICS) and long-acting bronchodilators (LABA)** as prescribed, including on the morning of surgery, with a sip of water.
  - **Pre-treatment:** Your anesthesiologist may recommend starting or increasing inhaler/nebulizer treatments (like a short-acting beta-agonist/SABA, e.g., albuterol) a few days before the procedure, even if they have no current symptoms.
  - **Steroids:** In some cases, a short course of oral steroids may be recommended if the asthma is poorly controlled.
- **Bring Inhalers:** Always bring your child's **inhaler and spacer** to the hospital on the day of surgery.

Special Steps for Diabetes

The main concern is managing blood sugar levels during a period of fasting, stress, and medication changes to prevent dangerous **hypoglycemia** (low blood sugar) or **hyperglycemia** (high blood sugar).

- **Individualized Management Plan:** Your child's endocrinologist and the anesthesia team will create a **specific, written plan** for managing their diabetes before, during, and after surgery.
- **Insulin/Medication Adjustment:**
  - **Basal Insulin:** Long-acting (basal) insulin doses may need to be **reduced or kept the same** depending on the timing of the surgery.
  - **Rapid-Acting Insulin:** Rapid-acting (bolus) insulin for meals and corrections is typically **omitted** on the morning of the procedure since your child will be fasting.
  - **Other Medications:** Oral diabetes medications may also be omitted.
- **Blood Glucose Monitoring:** Blood glucose (BG) levels will be monitored **frequently** (often hourly) before, during, and after the surgery.

- **IV Fluids:** An intravenous (IV) line will often be started on admission to administer fluids containing **glucose (dextrose)** to prevent hypoglycemia while fasting, especially if the surgery is major or delayed.
- **Priority Scheduling:** Procedures for diabetic children are often scheduled as the **first case of the morning** to minimize the fasting period.

The most important step you can take is to communicate everything about your child's condition, routines, and medications to the anesthesiologist and pre-operative team as soon as possible.

Would you like me to look for information regarding special precautions for **another specific condition** your child might have?

Sources:

https://www.aboutkidshealth.ca/why-are-pre-anesthesia-assessments-helpful#

https://patient.uwhealth.org/education/preparing-your-child-for-anesthesia

https://madeforthismoment.asahq.org/preparing-for-surgery/prep/preparing-for-surgery-kids-checklist/

https://www.childrens.com/specialties-services/specialty-centers-and-programs/anesthesiology/preparing-your-child-for-anesthesia#

https://www.aafp.org/pubs/afp/issues/2022/0600/p640.html#

https://www.piernetwork.org/diabetes_surgery.html#

Q17. When should my child and I arrive at the hospital on surgery day for anesthesia?

Answer:

That is a great question! For your child's safety and to ensure there is enough time for all necessary preparations, **hospitals typically require you to arrive 1.5 to 2 hours before the scheduled surgery or procedure time.**

This time frame is crucial because it allows for several important steps before the anesthesia is administered:

- **Registration and Check-in:** Completing the final admission paperwork.
- **Nursing Assessment:** A nurse will check your child's vital signs (temperature, pulse, blood pressure, etc.) and review their health history, especially regarding the strict **fasting guidelines** (no eating or drinking after certain times).
- **Anesthesiologist Consultation:** The anesthesiologist who will be caring for your child will meet with you to:
    - Review your child's medical history, allergies, and current medications.
    - Answer any questions you have about the anesthesia.
    - Obtain your final consent for the anesthesia.
- **Surgeon Consultation:** The surgeon will often meet with you to review the procedure and get your consent.

• **Pre-operative Preparation:** Nurses will help your child change into a hospital gown and may apply numbing cream for any potential IV insertion.

The most important step is to confirm the exact time with the hospital or your doctor's office.

You should receive a call from the hospital's pre-surgery nursing or pre-admission clinic 1 to 7 days before the surgery to confirm your precise arrival time and review all the critical instructions, especially the fasting cut-off times.

If you haven't received that call, you should contact the clinic or hospital directly the day before the surgery to ensure you have the correct information.

Would you like me to help you look up the contact number for the hospital or clinic where your child is having surgery?

Sources:

https://www.rch.org.au/kidsinfo/fact_sheets/Preparing_your_child_for_surgery_%E2%80%93_fasting/

https://www.rch.org.au/kidsinfo/fact_sheets/Preparing_your_child_for_surgery_general_anaesthesia/

https://www.carolinapediatricsurgery.com/the-day-of-surgery#

https://www.nationwidechildrens.org/your-visit/overnight-and-extended-stays/before-you-arrive/surgery-guide#

Q18. Can my child visit the surgery and recovery areas before anesthesia?

Answer:

This is a common and excellent question, as helping a child understand and prepare for their surgery can significantly reduce anxiety.

Many pediatric hospitals and surgery centers offer programs, often called **pre-operative tours** or **pre-surgical preparation programs**, that allow children and their families to visit some of the areas they will encounter, including the pre-op and recovery areas, before the day of surgery.

• **Pre-Operative/Pre-Surgical Tours:** These tours are specifically designed to familiarize the child with the sights, sounds, and processes of the surgical area, which can make the actual day less scary. During the tour, your child may:

- **Visit a pre-op (holding) room** and sometimes a recovery room (PACU).
- **See and even touch** some medical equipment in a non-stressful way.
- **Meet a Child Life Specialist** who uses play and age-appropriate explanations to talk about the procedure, anesthesia, and coping strategies.
- **Potentially meet an anesthesiologist** or nurse anesthetist.

• **Access to the Operating Room (OR):** While a child may visit the pre-op and recovery areas, directly visiting the operating room itself is less common due to strict sterile environment protocols, though some virtual or video tours may show the OR.

• **On the Day of Surgery:** Even without a prior tour, you are typically allowed to stay with your child in the **pre-operative area** (where they get ready) until they are taken into the operating room and sometimes until they are asleep under anesthesia.

You can often rejoin them in the **Post-Anesthesia Care Unit (PACU)**, or recovery room, as they wake up.

My recommendation is to call the specific hospital or clinic where your child's surgery is scheduled and ask about their **Child Life Services** or **Pre-Operative Tour Program**. They can tell you exactly what they offer, the best age to do a tour, and how to schedule it.

Would you like me to try to find the contact information for the hospital's child life department, if you provide the hospital name?

Sources:

https://www.wolfsonchildrens.com/services/surgery/pre-operative-tours#

https://www.ummhealth.org/services-treatments/childrens-medical-center/pediatric-surgery/pre-op-tours#

https://www.rch.org.au/kidsinfo/fact_sheets/preparing_your_child_for_hospital/#

https://kidshealth.org/en/parents/anesthesia.html#

Q19. Will my child be awake or know what's happening during anesthesia?

Answer:

That is a very understandable and common concern for parents. The primary goal of general anesthesia is to ensure your child is **unconscious, pain-free, and has no memory** of the procedure.

While general anesthesia is highly effective and safe, a very rare complication known as **Accidental Awareness During General Anesthesia (AAGA)**, or simply "awareness," can occur.

Here is a breakdown of what the research suggests about awareness in children:

1. Incidence of Awareness

- **Awareness is rare, but higher in children than adults.** In adults, the risk is typically cited as 0.1% to 0.2% (1 to 2 out of every 1,000 patients).
- In children, studies using direct questioning techniques have suggested the incidence of true awareness is **higher**, estimated to be around 0.6% **to** 0.8% (roughly 6 to 8 times higher than adults, or about 1 in every 135 to 167 children).

2. What Children Experience

Interestingly, the reported experience of awareness in children is often different from the severe distress reported by some adults:

- **Most Common Sensations:** When awareness does occur, children most frequently report **auditory** (hearing noises or voices) and **sensory** (feeling touch) perceptions.
- **Less Distress:** Several studies have noted that children who experienced awareness often **did not report the event as distressing** or upsetting when interviewed later. In one study, no aware child reported distress, and they had similar rates of behavioral disturbance as non-aware children.

- **No Explicit Recall of Pain/Distress:** Pain, anxiety, and paralysis (a very stressful part of awareness in adults) were less often mentioned by children.

It's theorized that young children may not have the same adult expectation of being completely unconscious, which may affect how they process and recall the experience.

3. What the Anesthesiologist Does

Your child's anesthesiologist is a highly trained specialist focused entirely on maintaining the deepest level of safety and unconsciousness throughout the procedure.

- They use sophisticated monitoring equipment to continuously track your child's vital signs (heart rate, blood pressure, oxygen levels) and adjust the anesthetic agents moment-to-moment to ensure your child remains properly "asleep" and unaware.
- They are highly aware of the difference in how children process and respond to anesthesia compared to adults.

I highly recommend you **discuss this specific concern with your child's pediatric anesthesiologist** before the procedure. They can provide you with:

- Details about the **specific anesthetic plan** for your child.
- Reassurance about the **monitoring techniques** they will use.
- Information on your child's **individual risk factors**, which may be higher or lower depending on their health and the type of surgery.

Would you like me to look up information on **common side effects** your child might experience *after* the anesthesia wears off, such as nausea or confusion?

Sources:

https://www.gosh.nhs.uk/conditions-and-treatments/procedures-and-treatments/accidental-awareness-during-general-anaesthesia-aaga/

https://pmc.ncbi.nlm.nih.gov/articles/PMC3026393/

https://ctanesth.com/awareness-under-anesthesia-in-children/

https://academic.oup.com/bja/article-abstract/102/1/104/228808

https://www.researchgate.net/publication/8005697_Awareness_During_Anesthesia_in_Children_A_Prospective_Cohort_Study

https://www.sunshinecoastanaestheticgroup.com.au/PatientInfo/ChildrenandAnaesthesia-408/SafetyofAnaesthesiainChildren-434/

Q20. Will my child feel any pain while under anesthesia?

Answer:

That is a very important question, and it's understandable that you want to ensure your child is comfortable and safe.

The short answer is **no**, your child should not feel any pain while under **general anesthesia**.

Here is a more detailed breakdown of how anesthesia works to prevent pain during a

procedure:

Pain Prevention During General Anesthesia

- **Insensitivity to Pain:** General anesthesia is designed to create a state of unconsciousness and complete insensitivity to pain. The anesthesiologist administers a mix of special intravenous medications and/or anesthetic gases, which are constantly monitored and adjusted throughout the entire procedure.

- **Unawareness and No Memory:** Under general anesthesia, your child is completely asleep, is not aware of the surgery happening, and will not remember the procedure.

- **Pain Relief Started Early:** It is standard practice for the pediatric anesthesiologist to start pain relief medications *during* the surgery itself. This is done to ensure the medication is already working when your child begins to wake up in the recovery room, helping to manage any pain from the surgery right away.

Minimizing Discomfort Before Anesthesia

While the anesthesia itself is painless, the healthcare team takes steps to minimize discomfort during the process of starting the anesthesia:

- **Numbing Cream:** Often, a local anesthetic cream (sometimes called "magic cream") is applied to the skin where an intravenous (IV) line will be inserted. This numbs the area to reduce the pain of the needle stick.

- **Pre-medication:** Your child may be given a sedative medicine, called a "pre-med," to help them relax and reduce anxiety before going into the operating room. This can be given as a liquid to drink.

Post-Surgery Pain Management

Once your child is waking up in the recovery room (Post-Anesthesia Care Unit or PACU), the focus shifts to managing post-operative pain, which is normal after surgery.

- **Immediate Relief:** The recovery room staff will closely monitor your child for any signs of discomfort and will administer pain medication quickly if needed.

- **Range of Options:** The type of pain relief used after surgery depends on the procedure and your child's age, and can include:

    - Simple medications like **paracetamol** and/or **ibuprofen**.

    - Stronger medications like **morphine** for major surgery, often given through an IV drip or a patient-controlled analgesia (PCA) pump for older children.

    - **Regional Anesthesia** (e.g., nerve blocks or epidurals) which are sometimes used during surgery to provide excellent pain relief that lasts for several hours after the procedure.

I highly recommend that you **discuss any concerns** about your child's pain relief plan with the anesthesiologist before the procedure. They can provide specific details based on your child's health and the planned surgery.

Do you have any other questions about the anesthesia process or what to expect in recovery?

Sources:

https://kidshealth.org/en/parents/anesthesia.html

https://www.healthywa.wa.gov.au/Articles/A_E/Anaesthetic-for-children-information-for-parents

https://www.wbas.com.au/patient-info/your-child-and-anaesthesia

https://www.rch.org.au/kidsinfo/fact_sheets/Preparing_your_child_for_surgery_general_anaesthesia/

https://www.healthychildren.org/English/health-issues/conditions/treatments/Pages/Managing-a-Childs-Pain-After-Surgery.aspx

https://www.gosh.nhs.uk/conditions-and-treatments/having-a-procedure-or-treatment-at-gosh/information-about-general-anaesthetic/

https://www.gloshospitals.nhs.uk/your-visit/patient-information-leaflets/anaesthetic-for-children/

Q21. Will someone watch my child the whole time during anesthesia to keep them safe?

Answer:

This is a very important and understandable concern, and the answer is a reassuring **yes**.

Your child will be continuously and closely monitored by highly trained medical professionals throughout the entire process to ensure their safety.

Here is a breakdown of who is responsible and when they are monitoring your child:

1. During the Procedure (Operating Room)

The primary person responsible for your child's safety and monitoring during the surgery or procedure is the **Anesthesiologist** (or Anaesthetist).

- **Role of the Anesthesiologist:** This is a specialist doctor who is present in the operating room for the entire time your child is under general anesthesia. Their sole focus is on your child's well-being, while the surgeon focuses on the operation.
- **What they Monitor:** They use specialized equipment to constantly track your child's vital signs and adjust medication as needed, including:
    - **Heart rate and rhythm** (ECG)
    - **Blood pressure**
    - **Breathing and oxygen levels** (Pulse oximetry)
    - **Carbon dioxide levels** (Capnography)
    - **Body temperature**

They are supported by an **Anesthesia Assistant** or **Anesthesia Technician** and other operating room staff.

2. After the Procedure (Recovery Room / PACU)

After the surgery is complete, your child will be moved to a Post-Anesthesia Care

Unit (PACU), or recovery room.

• **Role of the Recovery Room Nurse:** Your child's care is handed over to a **specially trained recovery room nurse** (sometimes called a PACU nurse).

• **Continuous Monitoring:** This nurse continues to monitor your child closely as they wake up from the anesthesia. They look for signs of pain, nausea, confusion, and ensure their breathing and vital signs remain stable until they are fully awake and ready to be moved or discharged.

The standard of care for pediatric anesthesia is to have highly skilled staff continuously present and monitoring the child during this vulnerable time.

I recommend discussing any specific concerns you have with your child's **Anesthesiologist** during the pre-operative consultation. They can walk you through the entire process and explain the specific monitoring techniques they will use for your child.

Would you like to search for common questions to ask a pediatric anesthesiologist before a procedure?

Sources:

https://kidshealth.org/en/parents/anesthesia.html#

https://www.sunshinecoastanaestheticgroup.com.au/PatientInfo/ChildrenandAnaesthesia-408/SafetyofAnaesthesiainChildren-434/

https://www.rch.org.au/kidsinfo/fact_sheets/Preparing_your_child_for_surgery_general_anaesthesia/

https://www.healthychildren.org/English/health-issues/conditions/treatments/Pages/Anesthesia-Safety-Infants-Toddlers-Parent-FAQs.aspx#

https://www.gosh.nhs.uk/conditions-and-treatments/having-a-procedure-or-treatment-at-gosh/information-about-general-anaesthetic/

https://www.asahq.org/standards-and-practice-parameters/statement-on-practice-recommendations-for-pediatric-anesthesia#

https://www.anzca.edu.au/patient-information/about-anaesthesia/anaesthesia-and-children#

https://publications.aap.org/pediatrics/article/143/6/e20191000/37173/Guidelines-for-Monitoring-and-Management-of

Q22. Can I stay with my child during the anesthesia process?

Answer:

That is a very common and important question for parents.

In many hospitals and medical centers, a practice called **Parental Presence at Induction (PPI)** is offered, which allows one parent or guardian to stay with their child as the anesthesia is being administered, right up until the child is fully asleep.

Key Points About Parental Presence at Induction

Why it's offered:

- **Reduces Child Anxiety:** Your presence can significantly reduce your child's separation anxiety and stress during a crucial moment.
- **Improves Cooperation:** A calmer child is often more cooperative with the medical team, making the induction process smoother.
- **Increases Parent Satisfaction:** Parents often feel better and more satisfied with their child's care when they are allowed to provide comfort during this time.

When it might not be possible:

The final decision is always made by the **anesthesiologist** and surgical team, as patient safety is the top priority. Circumstances that may prevent parental presence include:

- **Emergency Surgery**
- **If the child is heavily sedated** from pre-medication.
- **If the parent is very anxious or disruptive** (as children can pick up on parental anxiety, which can make the child more distressed).
- **Children outside a specific age range** (e.g., some policies exclude children under 1 year old or over 12, though this varies).
- **If the parent is pregnant** (due to exposure to anesthetic gases).
- If your child's condition changes or your presence is distracting to the medical staff.

Your Role During Induction

If you are approved to stay, you will typically be asked to:

- **Wear appropriate sterile attire** (such as a disposable gown, hair cover, and mask).
- **Remain Calm:** This is the most important thing. Your calm demeanor directly helps your child stay calm.
- **Provide Comfort:** You can hold your child's hand, sing softly, or talk to them in a soothing voice.
- **Follow Instructions Immediately:** The anesthesiologist or a staff member will tell you when it is time to leave, which is usually right after your child falls asleep. You must leave quickly and quietly at that time so the medical team can fully focus on the procedure.

What You Should Do Next

Because policies vary greatly between hospitals and medical facilities, the best step is to **ask your child's care team, especially the pediatric anesthesiologist**, about their specific policy for Parental Presence at Induction.

Would you like me to help you formulate the questions you can ask the hospital staff about their PPI policy?

Sources:

https://www.chp.edu/our-services/anesthesiology/patient-procedures/parental-presence

https://www.tandfonline.com/doi/full/10.1080/11101849.2022.2069335

https://patient.uwhealth.org/healthfacts/8234

Q23. How the anesthesia will be given to my child?

Answer:

That is a great and very important question to ask. The way anesthesia is given to a child is carefully chosen by the pediatric anesthesiologist based on the child's age, medical history, anxiety level, and the type of procedure or surgery.

Here is a breakdown of the common ways anesthesia is administered to children, focusing on the common type, **General Anesthesia**, which makes your child completely unconscious:

1. Starting the Anesthesia (Induction)

The anesthesiologist will usually choose the method that is most comfortable and least scary for your child to help them fall asleep.

- **Inhalation/Mask Induction:**
  - This is often the **preferred method for young children** or those afraid of needles.
  - An anesthetic gas, usually with a slightly sweet or "flavored" smell (like bubblegum or cherry), is delivered through a small, clear face mask placed gently over their mouth and nose.
  - Your child breathes the gas for a minute or two until they fall asleep.
  - **Often, a parent can stay with the child** until they are asleep for comfort.
  - Once the child is asleep, an IV line (thin plastic tube in a vein) is placed without them feeling it.
- **Intravenous (IV) Injection:**
  - This is often used for **older children** or if the child already has an IV placed for another reason.
  - The anesthetic medicine is injected directly into a vein through an IV line.
  - The medication takes effect very quickly, often within seconds.
  - To reduce the pain of the needle, a **numbing cream** (like EMLA) may be applied to the skin about 45-60 minutes before the IV is inserted.

2. Pain and Anxiety Relief Beforehand

To help your child relax and ease anxiety before the procedure, the team might:

- **Give a Sedative ("Pre-medication"):** This is often a liquid medication (like a syrup) given by mouth 10-20 minutes before going to the operating room. It helps the child feel sleepy and relaxed, making separation from you smoother, and can often cause them to forget the trip to the operating room.
- **Apply Numbing Cream:** A local anesthetic cream is often applied to the back of the child's hand or arm to numb the skin, making the IV insertion much less painful if that route is chosen.

3. Other Types of Anesthesia

General anesthesia is often combined with other techniques to help with pain during and after the surgery:

- **Regional Anesthesia:** An anesthetic is injected near a cluster of nerves to numb a large area of the body (like an arm, leg, or lower body). This can significantly reduce the amount of general anesthesia needed and provide excellent pain relief after the procedure.
- **Local Anesthesia:** A shot, spray, or ointment is used to numb a small, specific area (like the skin where stitches are needed).

The most important step is to talk to your child's pediatric anesthesiologist. They will meet with you before the procedure to discuss all the options, review your child's health history, and finalize the safest and most comfortable plan for your child.

Do you have any specific questions about **General Anesthesia** or one of the other types, like **Regional Anesthesia**?

Sources:

https://www.childrensmn.org/educationmaterials/parents/article/10467/types-of-anesthesia/

https://www.mountsinai.org/care/pediatrics/services/anesthesia/about

https://www.rch.org.au/kidsinfo/fact_sheets/Preparing_your_child_for_surgery_general_anaesthesia/

https://www.cincinnatichildrens.org/health/a/anesthesia-ent

https://www.anzca.edu.au/patient-information/about-anaesthesia/anaesthesia-and-children

https://kidshealth.org/en/parents/anesthesia-basics.html

https://www.gosh.nhs.uk/conditions-and-treatments/having-a-procedure-or-treatment-at-gosh/information-about-general-anaesthetic/

https://www.urmc.rochester.edu/encyclopedia/content?contenttypeid=90&contentid=p03013

Q24. Will my child need help breathing during anesthesia?

Answer:

That's a very common and important question for any parent whose child is having surgery.

If your child is having **general anesthesia**, they will be completely unconscious, and their natural breathing reflexes may be slowed or stopped.

Here is a breakdown of what that means for breathing support:

1. General Anesthesia and Breathing Support

- **Breathing Support is Routine:** During general anesthesia, the anesthesiologist will always monitor and often support your child's breathing to make sure they get enough oxygen. This is a standard and expected part of the process for patient safety.
- **Methods of Support:** Breathing assistance is provided using a device that

manages the airway, which could be:

- **A Face Mask:** Often used for very short procedures.
- **A Laryngeal Mask Airway (LMA):** A device that fits over the voice box.
- **A Breathing Tube (Endotracheal Tube or ETT):** A tube placed down the windpipe (trachea) for a secure airway. This is common for longer or more complex surgeries.

- **The Tube is Placed After Sleep:** If a breathing tube is needed, it is always inserted **after** your child is already asleep from the anesthesia. They will not be aware of it being placed or removed. A temporary sore throat is a common, mild side effect of the tube.

2. Other Types of Anesthesia

If your child is having **local** or **regional** anesthesia, where only a small part of the body is numbed and they remain conscious (or only mildly sedated), a breathing tube or extensive support is usually **not** needed. However, the anesthesia team will still monitor their breathing very closely.

3. Key Takeaway

The goal of the anesthesia team is to keep your child comfortable, pain-free, and **safe** during the entire procedure. **Controlling their breathing is a fundamental part of that safety.**

The best thing to do is to **ask the pediatric anesthesiologist** who will be caring for your child. They will review your child's medical history and the specific procedure to explain exactly what type of anesthesia and breathing support they plan to use.

Would you like me to search for some questions you can ask the anesthesiologist about the specific type of surgery your child is having?

Sources:

https://www.chop.edu/patients-and-visitors/guide-your-childs-surgery/day-your-childs-surgery/about-anesthesia

https://www.cincinnatichildrens.org/health/a/anesthesia-ent

https://www.gosh.nhs.uk/conditions-and-treatments/having-a-procedure-or-treatment-at-gosh/information-about-general-anaesthetic/

https://www.rch.org.au/kidsinfo/fact_sheets/Preparing_your_child_for_surgery_general_anaesthesia/

https://mydoctor.kaiserpermanente.org/mas/structured-content/Procedure_Inhalation_Induction_for_Children_-

_Anesthesiology.xml?co=%2Fregions%2Fmas

https://www.chop.edu/patients-and-visitors/guide-your-childs-surgery/day-your-childs-surgery/about-anesthesia

Q25.Will my child be sedated before getting anesthesia?

Answer:

That's a very common question, as parents understandably want to make the experience as comfortable as possible for their child.

The answer is **often, yes, but not always, and it depends on the child and the situation.**

The use of a sedative medicine before anesthesia is called **premedication** (or pre-med).

Key Points on Premedication

- **Primary Goal: Reduce Anxiety:** The main reason for giving a child a sedative beforehand is to help them relax, reduce their anxiety, and make the process of separating from their parents and going into the operating room less traumatic.
- **Not Routine for Everyone:** Premedication is not given to every child. It is typically determined by the anesthesiologist based on the child's age, level of anxiety, medical history, and the procedure itself.
  - Children between **2 and 6 years old** are often the most anxious and may benefit significantly from a sedative.
- **Common Sedatives:** The most commonly used sedative is a benzodiazepine called **midazolam**, often given as a sweet-tasting syrup, but other options like clonidine may also be used.
- **How it's Given:** The sedative is usually given by mouth (mixed with juice or a syrup) or sometimes through the nose, to avoid having to give an injection to a child who is already scared.
- **Effect:** The goal is to make the child calm and sleepy, sometimes with an added benefit of causing amnesia so they won't remember the time right before the anesthesia started.

Your Next Step

The most important thing is to **discuss this directly with your child's anesthesiologist** during the pre-operative consultation.

You can ask:

- "Will my child be given a premedication to help them relax before the general anesthesia starts?"
- "What medication is used, and how is it given?"
- "Can I be with my child until they are asleep?" (This is another common strategy used to reduce anxiety.)

Would you like me to look up general advice on preparing your child for surgery and anesthesia?

Sources:

https://www.rch.org.au/kidsinfo/fact_sheets/Preparing_your_child_for_surgery_general_anaesthesia/

https://www.rch.org.au/kidsinfo/fact_sheets/Sedation_for_procedures/

https://www.healthychildren.org/English/health-issues/conditions/treatments/Pages/Anesthesia-and-Children-The-Day-of-the-Procedure.aspx

https://pmc.ncbi.nlm.nih.gov/articles/PMC6761781/

Q26. How soon will my child wake up after anesthesia?

Answer:

That's a very common and important question for parents. The time it takes for a child to fully wake up after general anesthesia can vary, but here is a general overview:

Typical Wake-Up Time

- In the Post-Anesthesia Care Unit (PACU), a child may start to become **alert within about 40 minutes** after the procedure is finished.
- It's common for children to be very sleepy or groggy and **drift in and out of sleep** during the first **one to three hours** in recovery as the medication fully wears off.
- Most children will stay in the PACU for about **two hours** for close monitoring before being moved to an inpatient room or discharged for day surgery.

Faktoren, die die Erholungszeit beeinflussen (Factors Affecting Recovery Time)

Several factors influence how quickly your child wakes up and returns to their normal self:

- **Type of Anesthesia:** Different anesthetic agents wear off at different rates. The anesthesiologist chooses the best type and dose for your child and the procedure.
- **Length of the Procedure:** Longer surgeries may require more anesthetic, which can lead to a longer period of drowsiness afterward.
- **Your Child's Age and Health:** A child's overall health and individual reaction to the medication play a role.
- **Type of Procedure:** The invasiveness of the surgery itself can affect the recovery time.

What to Expect in the Hours After Waking Up

Even after moving out of the PACU, your child will likely still be drowsy.

- **Drowsiness and Sleep:** It is normal for your child to be more sleepy than usual for the first **24 to 48 hours** after being discharged. They should, however, be **fairly easy to wake up** when you try.
- **Behavioral Changes:** Some children may be confused, clingy, irritable, or even upset and angry (sometimes called emergence delirium) immediately upon waking. This is a common and usually short-lived side effect of the anesthesia, often settling within about an hour.
- **Coordination:** Your child's balance and coordination may be affected for up to **24 hours**, so adult supervision is necessary to prevent falls and injuries.

The best advice is to follow the specific discharge instructions provided by your

child's pediatric anesthesiologist and surgical team, as they will tailor their guidance to your child's unique case.

Would you like me to search for general information on **caring for a child at home** after general anesthesia?

Sources:

https://nyulangone.org/locations/hassenfeld-childrens-hospital/your-childs-visit/prepare-for-your-childs-surgery/after-your-childs-surgery

https://www.kids-dentist.com.au/general-anaesthesia-aftercare-children/

https://www.apsf.org/patient-guide/how-long-does-it-take-to-wake-up-from-anesthesia/

https://www.austinpediatricsurgery.com/post/pediatric-surgery-recovery-what-to-expect-post-op

https://www.chop.edu/patients-and-visitors/guide-your-childs-surgery/going-home-after-your-childs-surgery/what-expect-home

https://www.gosh.nhs.uk/conditions-and-treatments/having-a-procedure-or-treatment-at-gosh/information-about-general-anaesthetic/

Q27.When can I see my child after anesthesia and surgery?

Answer:

That is a very important and understandable question. While every hospital and every child's recovery is unique, here is a general overview of what you can typically expect.

When You Can See Your Child

Most hospitals aim to reunite parents with their child in the **Post-Anesthesia Care Unit (PACU)**, also known as the recovery room, as soon as it is safely possible.

- **Timeline:** You can generally expect to see your child within **30 minutes to one hour** after the surgery is complete and they have been transferred to the PACU.
- **Notification:** The recovery nurse or a staff member will call or find you in the waiting area to bring you back when your child is stable enough for visitors.
- **The PACU Environment:** Keep in mind that the PACU is a critical care area where your child will be closely monitored. There may be monitors, IV lines, and sometimes oxygen. For safety and patient privacy, visitor numbers are often restricted (usually **one or two adults** at a time), and siblings are often not allowed.

What to Expect When You See Your Child

Children wake up from general anesthesia in many different ways. It is normal and helpful to be prepared for the following:

- **Sleepiness:** Your child will likely be very sleepy, groggy, or just drifting in and out of sleep. This is normal as the anesthesia wears off. **Nurses will generally ask you not to wake them** as they often have a more comfortable wake-up process on their own.

- **Irritability or Confusion (Emergence Delirium):** Especially in younger children (preschool age), they may wake up confused, agitated, crying, or thrashing. This is called "emergence delirium" and **it is usually not related to pain**, but rather to waking up in an unfamiliar environment. It can be upsetting to witness, but it is temporary and they rarely remember it later.
- **Physical Discomfort:** They might experience common side effects like a **sore throat** (from the breathing tube), **nausea/vomiting**, shivering, or pain at the surgical site. The PACU team will manage pain and nausea with medication.
- **Your Role:** The best thing you can do is to **stay calm, speak softly**, and offer comfort and reassurance (touching, holding their hand) as they continue to recover.

What Happens Next

The length of time your child spends in the PACU varies greatly, but is typically **30 minutes to an hour or more**, depending on the type of surgery and how quickly they become stable and alert.

Once they are fully awake, their vital signs are stable, and their pain is controlled, they will be moved to:

- **A day surgery unit** if they are going home the same day.
- **An inpatient hospital room (a ward)** if they need to stay overnight.

Your best source of accurate information will always be the surgical team and the PACU nurses at your specific hospital, as visitor guidelines and recovery protocols can vary.

Would you like me to find some information about **what questions you should ask the surgeon** when they come to speak with you after the procedure?

Sources:

https://www.sickkids.ca/en/patients-visitors/planning-visit/coming-for-surgery/recovery-after-surgery/

https://www.childrensmn.org/educationmaterials/childrensmn/article/17454/post-anesthesia-care-unit-recovery-room/

https://www.cuh.nhs.uk/patient-information/information-for-parents-whose-child-is-having-an-operation/

https://www.nicklauschildrens.org/medical-services/pediatric-anesthesiology/post-anesthesia-care-unit-pacu

https://kidshealth.org/en/parents/anesthesia.html

https://www.rwjbh.org/bristol-myers-squibb-childrens-hospital-at-rwjuh/treatment-care/surgery/family-teaching-program/what-to-expect-in-the-recovery-room/

https://nyulangone.org/locations/hassenfeld-childrens-hospital/your-childs-visit/prepare-for-your-childs-surgery/after-your-childs-surgery

https://myhealth.alberta.ca/your-childs-surgery/at-the-hospital/post-anesthetic-care-unit-(pacu)

https://www.rch.org.au/kidsinfo/fact_sheets/RCH_operation_and_recovery_room/

Q28.Will my child have pain after anesthesia?

Answer:

It's very common and **normal for a child to experience some pain or discomfort** after surgery, but modern medical teams are highly focused on **managing this pain effectively** to keep your child comfortable.

The pain level will vary greatly depending on the **type and extent of the surgery** they have had.

Here is a breakdown of what to expect and how the pain is managed:

Types of Post-Anesthesia Discomfort

- **Pain at the Surgery Site:** This is the most expected pain and can range from mild to severe. It usually lessens as your child recovers.
- **Sore Throat or Hoarse Voice:** This is a common side effect of the breathing tube used during general anesthesia and usually resolves within a day or two.
- **Headache or Body Aches:** General discomfort is possible but is often mild and easily treated.
- **Tenderness or Bruising:** This may occur where IVs or cannulas were inserted.
- **Numbness/Weakness:** If your child received a **regional anesthetic block** (like a nerve block or epidural), the numb sensation will wear off over time, and they may start to feel pain as it goes away.

Effective Pain Management

Pain control is a priority, and the care team will create an individualized plan for your child.

- **Pain Relief Given During Surgery:** The anesthesiologist often administers pain medication, sometimes including local or regional anesthetic blocks, to ensure your child wakes up with minimal discomfort.
- **Multimodal Approach:** Pain is typically managed using a combination of medications for the best effect:
    - **Non-Opioids:** Medications like acetaminophen and ibuprofen or Advil are often the first line of defense.
    - **Opioids:** For moderate to severe pain, a stronger prescription pain medicine like oxycodone or hydrocodone may be used, generally for a very short time.
- **Timing of Doses:** You will likely be advised to give painkillers **regularly** (around-the-clock) initially, rather than waiting for the pain to become severe.

Non-Medication Comfort Measures

In addition to medicine, there are many ways you can help your child feel better:

- **Comfort and Distraction:** Reading, playing games, watching TV, or singing can help take their mind off the discomfort.
- **Familiar Items:** A favorite stuffed animal, blanket, or toy can provide significant comfort.

- **Physical Comfort:** Gentle rubbing of the head or hands, or simply holding or rocking them (check with the provider first).
- **Ice/Heat Packs:** Ask the care team if an ice pack or warm pad is safe and helpful for the surgical site.

It is highly recommended that you **discuss the expected pain level and the specific pain management plan** with your child's surgeon and anesthesiologist *before* the procedure. They can answer any questions and help you know exactly what to expect.

Do you have any specific questions about the **type of surgery** your child is having, or would you like more information on **how to tell if a young child is in pain**?

Sources:

https://www.healthychildren.org/English/health-issues/conditions/treatments/Pages/Managing-a-Childs-Pain-After-Surgery.aspx

https://pmc.ncbi.nlm.nih.gov/articles/PMC11325993/

https://www.schn.health.nsw.gov.au/surgery-and-anaesthesia-handbook/risks-anaesthesia/side-effects

https://www.gosh.nhs.uk/wards-and-departments/departments/clinical-specialties/anaesthesia-information-parents-and-visitors/side-effects-and-risks-of-a-general-anaesthetic/

https://kidshealth.org/en/parents/anesthesia.html

https://www.aboutkidshealth.ca/post-operative-and-post-anesthetic-care-caring-for-your-child-at-home

https://www.facs.org/for-patients/recovering-from-surgery/safe-pain-management/pediatric-pain-control/

https://www.uspharmacist.com/article/management-of-postoperative-pain-in-children

https://www.nationwidechildrens.org/family-resources-education/health-wellness-and-safety-resources/helping-hands/treating-pain-after-outpatient-surgery

Q29. Will my child feel sick or throw up after anesthesia?

Answer:

It is a common and valid concern for parents. **Postoperative Nausea and Vomiting (PONV)** is a recognized side effect of anesthesia, and children are actually at a higher risk for it than adults, with incidence rates estimated to be between 30% and 80% depending on the patient and the procedure.

However, the medical team will be aware of this risk, and there are very effective strategies to prevent and treat it.

What is the Likelihood?

About **one in every 10 children** undergoing anesthesia will experience nausea and vomiting, though for certain high-risk groups, the chance can be significantly higher.

Key Risk Factors for Your Child

Your child may be at a higher risk of PONV if they have:

- **A history of sickness with anesthesia** (in the past).
- **A history of motion sickness** (e.g., getting carsick).
- **An older age** (risk increases sharply around age 3 and peaks between 6-10 years).
- **Certain types of surgery,** particularly:
  - Eye surgery (like strabismus correction).
  - Ear, Nose, and Throat (ENT) surgery (like tonsillectomy or adenoidectomy).
  - Prolonged surgery (over 30 minutes).
- **Postoperative pain** that is managed with **opioids** (strong pain medications).

What is Done to Prevent It?

Your child's anesthesiologist will assess their risk factors and can customize the anesthesia plan to lower the chances of sickness. Prevention strategies often include:

- **Prophylactic Anti-Emetic Medications:** Giving anti-nausea medication **before or during** the surgery is the most common and effective strategy. Often, a combination of two different drugs is used for children with multiple risk factors.
  - Common medications include **Ondansetron** and **Dexamethasone**.
- **Anesthetic Technique:**
  - Using **Total Intravenous Anesthesia (TIVA)** (using propofol) instead of volatile gases (inhalational anesthetics) can help reduce the risk.
  - **Regional anesthesia** (which numbs a specific part of the body instead of putting the whole body to sleep) can reduce the need for general anesthesia and opioids.
- **Pain Management:** Using non-opioid pain medications (like paracetamol) and regional pain blocks can reduce the reliance on opioids, which are a common cause of nausea.

What to Discuss with the Anesthesiologist

The most important thing you can do is have an open conversation with the anesthesiologist before the procedure. Be sure to mention:

- **Any history of motion sickness** your child has.
- **If they have had anesthesia before** and if they felt sick afterward.
- **If you or other close family members** have a history of bad nausea or vomiting after anesthesia.

They will use this information to create the safest and most comfortable experience for your child.

Would you like to know more about the **recovery process** after anesthesia for children, or are you interested in a specific **anti-nausea medication** mentioned?

Sources:

https://www.frontiersin.org/journals/pediatrics/articles/10.3389/fped.2023.1241304/f

ull

https://www.schn.health.nsw.gov.au/surgery-and-anaesthesia-handbook/risks-anaesthesia/side-effects

https://pmc.ncbi.nlm.nih.gov/articles/PMC7101192/

https://www.openanesthesia.org/keywords/postoperative-nausea-and-vomiting-in-children/

https://www.hss.edu/health-library/conditions-and-treatments/effects-of-anesthesia-on-children

Q30. If my child had spinal or epidural anesthesia, could they get a headache afterward?

Answer:

Yes, it is possible for a child to get a specific type of headache called a **Post-Dural Puncture Headache (PDPH)** after having spinal or epidural anesthesia.

Here is a breakdown of what you should know:

What is a Post-Dural Puncture Headache?

Cause: Both spinal and epidural procedures involve placing a needle near the spinal cord. This is contained within a protective sac called the dura, which holds cerebrospinal fluid (CSF).

- In a **spinal anesthetic**, a fine needle is intentionally passed through the dura.
- In an **epidural**, the needle is intentionally placed just outside the dura, but sometimes the dura is accidentally punctured (called an "accidental dural puncture").
- If CSF leaks out through this small hole, the pressure around the brain and spinal cord decreases, which can cause a PDPH.

Incidence in Children: The risk of PDPH in the pediatric population is generally considered lower than in adults, especially in younger children (under 12 years old), but it still occurs. Estimates for the incidence in children range from **1% to 15%**, depending on factors like age and the size of the needle used.

Key Symptoms of PDPH

The hallmark sign of a PDPH is that the headache is **positional**, meaning it is related to whether the child is sitting up or lying down.

- **Worsens:** The headache typically **worsens** when the child sits or stands up.
- **Improves:** The headache typically **improves** when the child lies flat.

Other common symptoms can include:

- Nausea and/or vomiting
- Neck stiffness
- Dizziness
- Changes in hearing (like ringing or muffled sound) or vision

- The headache usually starts **within 5 days** of the procedure.

When to Contact a Healthcare Provider

It is important to contact the anesthesia or medical team if your child develops a severe headache after the procedure, especially if it:

- **Fits the postural pattern** (gets worse when sitting/standing).
- **Does not improve** with over-the-counter pain relievers.
- Is accompanied by fever, confusion, or severe nausea/vomiting.

If you have any concerns at all about a headache your child develops after the procedure, **always consult with their healthcare provider** right away.

Would you like information on how PDPH is typically managed or treated?

Sources:

https://rcoa.ac.uk/patients/patient-information-resources/anaesthesia-risk/headache-after-spinal-or-epidural-anaesthetic

https://rapm.bmj.com/content/49/7/471

https://www.cuh.nhs.uk/patient-information/headache-after-an-epidural-or-spinal-anaesthetic/

https://pmc.ncbi.nlm.nih.gov/articles/PMC10960508/

https://www.dovepress.com/management-of-post-dural-puncture-headaches-in-pediatric-patients-with-peer-reviewed-fulltext-article-JPR

https://pmc.ncbi.nlm.nih.gov/articles/PMC11260652/

https://www.childrensmercy.org/health-care-providers/evidence-based-practice/cpgs-cpms-and-eras-pathways/post-dural-puncture-headache-for-only-pediatric-patients-care-process-model/

https://rapm.bmj.com/content/49/7/471

https://rcoa.ac.uk/patients/patient-information-resources/anaesthesia-risk/headache-after-spinal-or-epidural-anaesthetic

Q31.How soon can my child eat and drink after waking up from general anesthesia?

Answer:

This is an important question, and the most critical thing is to **follow the specific instructions given by your child's healthcare team** (surgeon, anesthesiologist, or nurse) because guidelines can vary based on:

- **The type of surgery or procedure** your child had (e.g., abdominal surgery may require a longer delay).
- **The individual child's recovery** and whether they are fully awake and alert.
- **Any specific risks** your child may have.

However, here are the general guidelines for children after general anesthesia:

1. Wait Until the Child is Fully Awake and Alert

- Children should **wake naturally and be alert** before being offered anything to eat or drink.
- They may be sleepy, irritable, or confused immediately after waking up, which is normal.

2. Start with Clear Liquids, Slowly

- If your child is alert and doesn't feel sick, they are usually offered **clear liquids first** (like water, apple juice, or popsicles).
- This is typically done in the recovery room or shortly after returning to the ward or going home (for day-stay surgery).
- The goal is to ensure they can tolerate fluids without nausea or vomiting.

3. Advance to Light Food

- If the clear liquids are tolerated without vomiting, they can usually **advance to a light meal** (such as toast, crackers, soup, or cereal) or resume breast milk/formula within a few hours.
- For day surgery, most children should be able to eat and drink as usual within **1 to 2 hours** of the procedure, provided they are tolerating the initial clear fluids.
- **Avoid forcing** your child to eat; their appetite may be small at first.

Why The Caution?

The main reasons for starting slowly are to avoid:

- **Nausea and Vomiting:** Anesthesia and some pain medications can cause a child to feel sick. Eating or drinking too soon or too quickly increases the risk of vomiting.
- **Aspiration:** Vomiting increases the risk of inhaling stomach contents into the lungs, which is a serious complication.
- **"Sleepy Bowels":** Especially after abdominal surgery, the intestines can be slow to wake up, and doctors may advise a longer fast until the child has passed gas or stool.

In summary, your child should not be given anything until they are fully awake, and then you should start with small amounts of clear fluids as directed by the hospital staff.

Do you have the specific post-operative instructions from your healthcare team, or are you looking for general information?

Sources:

https://www.nationwidechildrens.org/family-resources-education/700childrens/2019/10/what-can-kids-eat-before-and-after-surgery

https://kidshealth.org/en/parents/anesthesia.html

https://www.chop.edu/patients-and-visitors/guide-your-childs-surgery/your-childs-recovery-after-surgery

https://www.aboutkidshealth.ca/post-operative-and-post-anesthetic-care-caring-for-your-child-at-home

https://www.cuh.nhs.uk/patient-information/information-for-children-having-had-general-anaesthesia/

https://www.healthychildren.org/English/health-issues/conditions/treatments/Pages/Anesthesia-and-Children-The-Day-of-the-Procedure.aspx

Q32. How long will my child need to stay at the hospital after anesthesia?

Answer:

That's a very important question. The length of time your child needs to stay in the hospital after anesthesia depends heavily on **the type of procedure/surgery** they had and **their specific recovery** from the anesthetic.

Here is a breakdown of what you can generally expect:

Immediate Recovery (PACU)

Right after the procedure, your child will be taken to the Post-Anesthesia Care Unit (PACU), also known as the recovery room.

- **Duration:** Most children stay in the PACU for **30 minutes to a few hours**, typically **around one to two hours**.
- **What Happens:** Doctors and nurses closely monitor them as they wake up from the anesthesia. They ensure their vital signs are stable, their pain is managed, and any immediate side effects (like nausea or vomiting) are controlled.
- **Parents:** You can usually join your child in the PACU once they start waking up a bit more.

Total Hospital Stay Before Discharge

Once they meet the PACU discharge criteria, their further stay depends on whether the procedure was **day surgery** or if they require **inpatient admission**:

- **Day Surgery (Ambulatory):**
  - f the procedure was minor (like some dental procedures or very small surgeries), they may be discharged from the hospital **a few hours** after leaving the PACU.
  - The goal for day surgery is often to get them home the **same day**. They will need to be fully awake, have stable pain management, and be able to take fluids by mouth without excessive vomiting.
- **Inpatient Stay:**
  - If the surgery was major, or if your child has a complex medical condition, they will be transferred to a hospital ward to **stay overnight or for several days** for continued observation.
  - **Infants:** Very young infants, especially those born prematurely and under **55 weeks Post-Conceptual Age (PCA)**, are often required to stay overnight for respiratory monitoring, even for minor procedures.

Key Discharge Criteria

Before your child can go home after a general anesthetic, they usually need to meet certain criteria, which include:

- **Alertness:** Being fully conscious and alert (or back to their pre-operative baseline).
- **Vitals:** Stable vital signs (heart rate, breathing, blood pressure).
- **Nausea/Vomiting:** Minimal to no nausea or vomiting.
- **Pain Control:** Pain is well-managed with oral medication.
- **Fluids:** They can drink fluids without difficulty.
- **Mobility:** Able to sit up and walk (or return to their baseline mobility).
- **Responsible Adult:** A responsible adult must be present to accompany them home and stay with them for the next **24 hours**.

For the most accurate information, I highly recommend discussing the expected recovery and discharge timeline **directly with your child's care team**, including the **anesthesiologist** and **surgeon**, as it is specific to their unique situation.

Would you like me to look up information about **common side effects** your child might experience in the first 24 hours after general anesthesia?

Sources:

https://www.austinpediatricsurgery.com/post/pediatric-surgery-recovery-what-to-expect-post-op

https://kidshealth.org/en/parents/anesthesia.html

https://www.openanesthesia.org/keywords/pacu-discharge-criteria/

https://www.maskinduction.com/mandatory-admission-criteria.html

https://www.southtees.nhs.uk/resources/discharge-advice-general-anaesthetic/

Q33.Who can I contact if I have more questions after my child’s anesthesia?

Answer:

This is a very important question, as having concerns after a child's procedure is completely normal.

The most appropriate person or service to contact usually depends on whether the question is about **immediate recovery** or **general follow-up**.

Here are the typical contacts, and which one to prioritize:

1. The Hospital or Surgery Center

- **During the first 24-48 hours after discharge (especially if for a day procedure):**
    - **The Post-Anesthesia Care Unit (PACU) Nurse:** If you went home the same day, they often give you a direct phone number or a number for the **Day Surgery Unit** or **Recovery Room** for immediate, non-emergency questions (e.g.,

managing nausea, pain that is worse than expected, or excessive sleepiness).

- **The Ward/Floor Nurse:** If your child was admitted overnight, the nurse on the unit where your child stayed is the best first point of contact after you go home, as they are familiar with your child's case and discharge instructions.

2. Your Child's Healthcare Providers

- **For non-urgent concerns or follow-up after the initial recovery period:**

  - **The Anesthesiologist's Office:** They are the specialists in the anesthesia itself and can answer detailed questions about medications, lingering side effects (like sore throat or minor sleep disturbances), and specific aspects of the anesthetic plan.

  - **The Surgeon or Proceduralist's Office:** They can answer questions about the surgical site, wound care, and general recovery from the *procedure itself*, which often relates to pain management that was started by the anesthesiologist.

  - **Your Child's Pediatrician/Primary Care Provider:** For general health concerns, fever, or questions that feel less related to the immediate post-operative period.

When to Seek Immediate Medical Attention

If your child is experiencing **severe symptoms** or you have an **emergency concern**, do not wait to call one of the offices above. **Call the emergency number (e.g., 911 or your local equivalent) or go to the nearest emergency department.**

Emergency signs can include (but are not limited to):

- Difficulty breathing or labored, fast, or shallow breathing.
- Persistent, uncontrollable vomiting (e.g., more than 3 times within a few hours).
- High fever (often defined as 101.3° F or 38.5° C or higher).
- Difficulty waking your child up, or if they remain excessively sleepy after 6 hours.
- Uncontrolled or severe pain not helped by prescribed medicine.

Action to Take

When you leave the hospital, **always confirm the best contact number for post-anesthesia questions** and make sure you receive a written copy of the discharge instructions. This document is your most important resource.

Would you like me to look up the non-emergency contact information for a specific hospital or clinic where your child had the procedure?

Sources:

https://kidshealth.org/en/parents/anesthesia.html

Model 4

Q1.What are the different types of anesthesia?

Answer:
There are four main categories of anesthesia, each used for different procedures and levels of pain control. They differ in how much of the body is numbed and whether a person is awake or unconscious.

General anesthesia

General anesthesia causes complete loss of consciousness, so the patient is "asleep," feels no pain, and usually has no memory of the procedure. It is typically delivered through IV medications and/or inhaled gases and is used for major or long surgeries involving internal organs or large areas of the body.

Regional anesthesia

Regional anesthesia numbs a larger region of the body, such as everything below the waist or an entire arm or leg, while the patient may remain awake or lightly sedated. Common forms include spinal and epidural anesthesia (often used in childbirth or lower-body surgery) and peripheral nerve blocks for limb operations.

Local anesthesia

Local anesthesia numbs only a small, specific area, such as a tooth, a patch of skin, or a small wound. The patient stays fully awake and aware, and this approach is usually used for minor procedures like dental work, skin biopsy, or stitching a small cut.

Sedation / monitored anesthesia care

Sedation (often called monitored anesthesia care or "twilight" anesthesia) uses medications to help a patient relax and feel sleepy, with pain control tailored to the procedure. Sedation can range from minimal (relaxed but fully awake) through moderate and deep levels, which are commonly used for procedures like endoscopy or colonoscopy where full general anesthesia is not needed.

Summary table

Type	Conscious?	Area affected	Typical uses
General anesthesia	Unconscious, no awareness	Whole body	Major or long surgeries
Regional anesthesia	Usually awake or sedated	Large region (e.g., limb, below waist)	Childbirth, limb, or lower-body surgery
Local anesthesia	Fully awake	Small, specific area	Minor skin, dental, or office procedures
Sedation / MAC	Ranges from drowsy to deeply sleepy but not fully under like general anesthesia	Works with local/regional to control discomfort	Endoscopy, minor surgery, some imaging tests

1. https://www.nm.org/conditions-and-care-areas/surgical-services/types-of-anesthesia
2. https://www.uclahealth.org/medical-services/anesthesiology/types-anesthesia
3. https://www.apsf.org/patient-guide/what-are-the-types-of-anesthesia/
4. https://www.nigms.nih.gov/education/fact-sheets/Pages/anesthesia
5. https://medlineplus.gov/anesthesia.html

6. https://www.goodrx.com/conditions/anesthesia/types-of-anesthesia
7. https://www.unasourcesurgery.com/types-of-anesthesia/
8. https://madeforthismoment.asahq.org/anesthesia-101/types-of-anesthesia/
9. https://my.clevelandclinic.org/health/treatments/15286-anesthesia
10. https://kidshealth.org/en/parents/anesthesia-basics.html

Q2. What is general anesthesia？

Answer:
General anesthesia is a medically induced, controlled state of unconsciousness used mainly during surgery so that you feel no pain, have no awareness, and do not remember the procedure. It affects the entire body and is managed throughout the operation by specialized anesthesia providers.

Basic definition
General anesthesia uses medicines given through a vein, by inhaled gases, or by both, to put you into a sleep-like state where the brain does not respond to pain signals or reflexes. During this state, you are unconscious, do not move, and do not experience or remember what happens in the operation.

Main purposes
The goals of general anesthesia are to keep you unconscious, pain-free, still, and free of memory during procedures that would otherwise be intolerable. This allows surgeons to perform major operations safely, including those that require muscle relaxation and control of breathing.

How it is given
General anesthesia is usually started with medicines into a vein, a mask with anesthetic gas, or both, causing you to fall asleep within a few minutes. During surgery, additional anesthetic drugs and gases are continuously adjusted to maintain the desired level of unconsciousness and pain control.

Monitoring and safety
While under general anesthesia, a trained anesthesia professional monitors breathing, heart rate, blood pressure, oxygen levels, and other vital signs and supports them if needed. Modern techniques and monitoring make general anesthesia very safe for most people, although risks and side effects still exist and are assessed before surgery.

Difference from other anesthesia types
Unlike regional anesthesia, which numbs a larger body area (such as from the waist down), or local anesthesia, which numbs only a small spot, general anesthesia affects the whole body and causes complete unconsciousness. It also differs from sedation, where you are relaxed and may be drowsy or lightly asleep but not fully unconscious in the same way as with general anesthesia.

1. https://en.wikipedia.org/wiki/General_anaesthesia
2. https://www.webmd.com/a-to-z-guides/what-is-general-anesthesia
3. https://www.mayoclinic.org/tests-procedures/anesthesia/about/pac-20384568
4. https://www.yalemedicine.org/conditions/general-anesthesia
5. https://www.betterhealth.vic.gov.au/health/conditionsandtreatments/general-anaesthetics

6. https://madeforthismoment.asahq.org/anesthesia-101/types-of-anesthesia/general-anesthesia/
7. https://my.clevelandclinic.org/health/treatments/15286-anesthesia
8. https://www.uclahealth.org/medical-services/anesthesiology/types-anesthesia
9. https://medlineplus.gov/ency/article/007410.htm
10. https://www.nhs.uk/tests-and-treatments/general-anaesthesia/

Q3. How is anesthesia different for children compared to adults?

Answer:
Anesthesia in children is tailored to their developing bodies and minds, so it differs in important ways from anesthesia in adults. These differences affect drug doses, monitoring, techniques, and how the team prepares the child and family.

Body and organ differences

Children are not just "small adults"; their airway, heart, lungs, liver, and kidneys work differently and are still maturing. For example, young children have smaller airways, higher oxygen use, and less reserve, so they can develop breathing or oxygen problems faster than adults if something goes wrong.

Because infants and young children rely more on heart rate to maintain blood pressure, changes in heart rate under anesthesia can affect their circulation more quickly than in adults. Their immature liver and kidney function and different body water and fat content also change how anesthetic drugs are distributed and cleared.

Drug dosing and choice

In adults, many anesthetic drugs are given in fairly standard ranges, but in children almost all doses are calculated very precisely by weight and sometimes by age. After the newborn period, children often need higher doses per kilogram for some anesthetics than adults because of higher metabolism and larger volume of distribution.

Newborns and very young infants can be more sensitive to certain drugs, so doses may be smaller and increased slowly while the team watches responses closely. The choice of drugs can also differ, with extra attention to side effects such as effects on breathing, blood pressure, and blood sugar.

Airway and breathing management

Pediatric airway anatomy (larger tongue relative to mouth, higher larynx, different narrowest point) makes securing and protecting the airway more technically challenging than in adults. Because children desaturate faster when they are not breathing, the team prepares carefully with appropriate equipment sizes and backup plans.

Oxygen and ventilation settings are adjusted to the child's size and lung characteristics rather than using adult-style settings scaled down. Extra care is taken to avoid airway swelling, laryngospasm (spasm of the vocal cords), and other breathing complications, which are relatively more common in children.

How anesthesia is started

Many adults receive anesthesia through an intravenous (IV) line while awake, but small children often start anesthesia with a flavored mask so the IV can be placed after they are asleep to reduce distress. This "mask induction" uses inhaled anesthetic gases and is a standard pediatric technique but is used less often in adults.

Older children and teenagers may be managed more like adults but still with age-appropriate

explanations and sometimes medications by mouth or nose to ease anxiety before IV placement. The overall plan is adapted to the child's developmental stage and ability to cooperate.

Monitoring, safety, and side effects

Monitoring standards (for things like heart rate, blood pressure, oxygen levels, and breathing) are similar, but anesthesiologists pay closer attention to rapid changes in children because their condition can deteriorate faster. Children are also more prone to losing body heat, so warming measures are used more aggressively than in most adults.

Postoperative nausea and vomiting can be more common in children, so preventive medications are often used. Pain control may rely more on carefully dosed weight-based medications and sometimes on regional anesthesia (like nerve blocks or caudals), with doses and techniques adjusted for pediatric anatomy and drug handling.

Emotional and psychological aspects

Children often have strong fears about separation, needles, and the unknown, so the anesthesia team spends more time preparing and reassuring both child and parents. Techniques can include child-friendly explanations, play therapy, parental presence during induction in some settings, and anxiety-reducing medicines.

Communication after surgery is also adapted; younger children may show pain or nausea through behavior rather than words, so nurses and anesthesiologists use age-appropriate pain scales and close observation. Pediatric anesthesiologists receive specific training to handle these medical and emotional needs safely.

1. https://radiusanesthesia.com/pediatric-vs-adult-anesthesia/
2. https://blog.cincinnatichildrens.org/patient-family-experience/sedation-general-anesthesia/
3. https://www.uspharmacist.com/article/general-anesthesia-in-pediatric-patients
4. https://www.youtube.com/watch?v=9En-YvHnXTY
5. https://www.pedsurglibrary.com/apsa/view/Pediatric-Surgery-NaT/829462/all/Anesthesia
6. https://www.maskinduction.com/what-makes-pediatric-anesthesia-different.html
7. https://asra.com/news-publications/asra-updates/blog-landing/legacy-b-blog-posts/2024/07/15/pediatric-regional-anesthesia-considerations-for-adult-anesthesiologists
8. https://www.hss.edu/health-library/conditions-and-treatments/effects-of-anesthesia-on-children
9. https://www.nyp.org/news/anesthesia-for-children-only
10. https://www.youtube.com/watch?v=eSVyMCRQJB0

Q4. How is general anesthesia different from sedation?

Answer:

General anesthesia and sedation are points on the same spectrum of how "asleep" and unaware you are during a procedure, but they differ in depth of unconsciousness, breathing support needs, and typical uses. Understanding the differences helps clarify why a clinician might recommend one over the other for a specific procedure.

Consciousness and awareness

- Sedation makes you relaxed and drowsy, ranging from minimally sleepy to almost but not fully unconscious; you may respond to voices or touch and might remember little or nothing of the procedure.
- General anesthesia produces a complete, drug-induced loss of consciousness in which you

are not aware of your surroundings and do not respond even to painful stimuli.

Breathing and vital functions

- With minimal or moderate sedation, you usually breathe on your own and maintain protective airway reflexes; heart and blood pressure are typically stable.
- Under general anesthesia, breathing is often impaired, so anesthetic staff usually support or fully control breathing (for example with a breathing tube), and blood pressure or heart function may be more affected and require closer monitoring.

Typical uses and procedures

- Sedation is commonly used for less invasive procedures (such as endoscopy, minor surgery, or many dental and office-based procedures) and is often combined with local or regional anesthesia to control pain while you remain somewhat responsive.
- General anesthesia is usually chosen for more invasive, longer, or painful operations where complete unconsciousness, immobility, and a secured airway are needed (for example major abdominal, orthopedic, or complex facial surgeries).

Memory, pain control, and recovery

- Sedation often causes partial or complete amnesia for the procedure but does not itself fully block pain, so local anesthetics or analgesics are added; recovery is usually quicker, especially with lighter levels.
- General anesthesia includes loss of consciousness plus strong pain control and amnesia; waking up can take longer, and grogginess or other side effects after the procedure are more common.

Side-by-side overview

Aspect	Sedation	General anesthesia
Consciousness	Drowsy to deeply sleepy but not fully unconscious; may respond to stimuli	Completely unconscious; no response even to pain
Breathing	Often spontaneous; airway reflexes usually maintained	Frequently needs breathing support and airway device
Pain control	Mainly reduces anxiety; pain blocked with added local/other meds	Strong systemic pain control built into anesthetic plan
Typical procedures	Minor or moderate procedures, many dental or endoscopy cases	Major or long surgeries needing full unconsciousness
Recovery	Usually faster; less post-procedure grogginess	Slower; more postoperative drowsiness possible

If you share the type of procedure you are facing, more tailored pros and cons for sedation versus general anesthesia can be outlined.

1. https://www.medicinenet.com/sedation_vs_general_anesthesia/article.htm
2. https://www.emedicinehealth.com/sedation_vs_general_anesthesia_difference/article_em.htm
3. https://www.asahq.org/standards-and-practice-parameters/statement-on-continuum-of-depth-of-sedation-definition-of-general-anesthesia-and-levels-of-sedation-analgesia
4. https://myvibedentistry.com/difference-between-anesthesia-and-sedation/
5. https://roh.nhs.uk/supporting-services/anaesthetic/general-anaesthetic-and-sedation
6. https://pcoms.ca/2019/12/the-main-differences-between-sedation-and-anesthesia/

7. https://briargateadvancedfamilydental.com/resources/difference-sedation-anesthesia
8. https://saxonmd.com/blog/general-anesthesia-vs-sedation/
9. https://blog.cincinnatichildrens.org/sedation-general-anesthesia/
10. https://www.denverfaceandbody.com/blog/iv-sedation-vs-general-anesthesia/

Q5. Is general anesthesia safe for my child?

Answer:
General anesthesia for children today is generally very safe, especially when a child is reasonably healthy and the care team includes a pediatric anesthesiologist.

Overall safety
Modern monitoring, equipment, and medications have made serious complications from anesthesia in children rare, particularly for brief, routine procedures in otherwise healthy kids. Major medical groups such as the American Academy of Pediatrics emphasize that anesthesia is necessary to safely perform painful or stressful procedures and is usually the safest approach for most surgeries in infants and children.

Risks and side effects
Common short-term side effects in kids include drowsiness, nausea, vomiting, sore throat, or irritability, which usually resolve within hours. Serious problems such as breathing issues, allergic reactions, or heart complications are uncommon, and the individual risk depends on the child's age, overall health, and the type and length of the procedure.

Brain development concerns
The U.S. Food and Drug Administration warns that repeated or very long (more than about 3 hours) exposures to general anesthesia or sedation in children under 3 could potentially affect brain development, based largely on animal studies. Human studies so far suggest that a single, short exposure is unlikely to cause learning or behavior problems, and large trials have not shown clear neurodevelopmental harm from brief anesthesia in infancy.

When to be extra cautious
Extra caution and monitoring are especially important for premature infants, very young children, or those with serious heart, lung, or neurological conditions, because their baseline risk is higher. For some purely elective procedures in very young children, professional guidelines often recommend considering delaying surgery until after age 3 if it is medically reasonable, to reduce theoretical long-term brain risks.

How to talk with your child's doctors
Before the procedure, ask to speak with the anesthesiologist about your child's specific risks, the expected duration of anesthesia, and any alternatives such as regional blocks that might reduce the total amount of anesthetic needed. It is also important to review your child's medical history, medications, allergies, and any prior anesthesia experiences, so the team can tailor the plan and further improve safety.
If you share your child's age, health conditions, and the type of procedure, more tailored risk information and question prompts for the doctor can be provided.

1. https://health.clevelandclinic.org/is-anesthesia-safe-for-kids
2. https://www.healthychildren.org/English/health-

issues/conditions/treatments/Pages/Anesthesia-Safety-Infants-Toddlers-Parent-FAQs.aspx
3. https://answers.childrenshospital.org/what-parents-should-know-pediatric-anesthesia/
4. https://www.pedsurglibrary.com/apsa/view/Pediatric-Surgery-NaT/829462/all/Anesthesia
5. https://www.hss.edu/health-library/conditions-and-treatments/effects-of-anesthesia-on-children
6. https://www.uclahealth.org/medical-services/anesthesiology/anesthesia-and-child-development
7. https://www.uclahealth.org/news/article/anesthesia-in-young-children-know-the-risks
8. https://kidshealth.org/en/parents/anesthesia.html
9. https://www.healthychildren.org/English/health-issues/conditions/treatments/Pages/Anesthesia-and-Children-The-Day-of-the-Procedure.aspx
10. https://myhealth.alberta.ca/Health/aftercareinformation/pages/conditions.aspx?hwid=acm5237

Q6. Can anesthesia harm my child's brain development?

Answer:
Most evidence so far is reassuring for single, short anesthesia exposures in children, but there may be some risk with long or repeated exposures in very young kids. The decision to do surgery still usually favors treating the medical problem rather than delaying necessary care.

What is known from human studies
Large human studies show that a single, relatively brief general anesthesia in early childhood does not appear to lower overall intelligence or cause broad learning disabilities later on. Trials comparing babies who had a short general anesthetic with those who had regional (spinal) anesthesia did not find differences in overall IQ or most developmental measures at follow-up. At the same time, some studies report small differences in specific areas such as working memory or processing speed, especially when exposure occurred in the first few years of life.

When risk seems higher
Concerns are greatest for children under about 3 years old who need anesthesia many times or for very long operations (often discussed as longer than about 3 hours). In this group, research links multiple or prolonged anesthetics with modest increases in problems like fine motor skills, processing speed, or behavior, although general intelligence typically remains in the normal range. The U.S. FDA has warned that repeated or lengthy use of anesthesia in children under 3 or in the third trimester of pregnancy may affect brain development, and recommends weighing risks and benefits carefully.

Animal data and why doctors are cautious
In animals, common anesthetic drugs can trigger nerve cell changes and long-term learning or behavior problems when given during rapid brain growth. These findings raise concern but do not directly predict what happens in humans, where doses, timing, and many other factors differ. Because of this uncertainty, specialists try to avoid unnecessary anesthesia in very young children and to limit duration and number of exposures when possible.

Practical points for parents

- Treating the underlying condition (for example, a bad infection, blocked airway, or painful surgical problem) is usually more important for long-term health than the small, uncertain

anesthesia risk.

- For elective (non-urgent) procedures in children under 3, many teams will discuss whether the procedure can be safely delayed or combined with other needed procedures to reduce total exposures.
- Pediatric anesthesiologists use the lowest effective doses, monitor closely, and choose techniques aimed at maximizing safety and comfort.

Questions to ask your child's doctors

- Is this surgery or procedure urgent, or can it safely be delayed until my child is older?
- Approximately how long will my child be under anesthesia, and is there a plan to minimize the time?
- Does my child have any additional risk factors (prematurity, other medical or developmental issues)?
- Will a pediatric anesthesiologist be caring for my child, and are there alternatives (like regional or local anesthesia) for all or part of the procedure?

For a specific child, discussing age, medical history, the urgency of the procedure, and anesthesia plan directly with the surgeon and anesthesiologist is the best way to understand real-world risk and make a balanced decision.

1. https://www.frontiersin.org/journals/neuroscience/articles/10.3389/fnins.2024.1406172/full
2. https://pmc.ncbi.nlm.nih.gov/articles/PMC9551616/
3. https://www.apsf.org/article/the-effect-of-general-anesthesia-on-the-developing-brain-is-it-time-to-temper-the-concern/
4. https://pmc.ncbi.nlm.nih.gov/articles/PMC7511469/
5. https://kidshealth.org/en/parents/anesthesia-brain.html
6. https://pmc.ncbi.nlm.nih.gov/articles/PMC3275974/
7. https://www.childrenscolorado.org/just-ask-childrens/articles/anesthesia-and-developing-brains/
8. https://med.stanford.edu/pedsanesthesia/patients/anes-child-brain.html
9. https://www.apsf.org/article/the-effect-of-general-anesthesia-on-the-developing-brain-appreciating-parent-concerns-while-allaying-their-fears/
10. https://kidshealth.org/HumanaSouthCarolina/en/parents/anesthesia-brain.html

Q7. Is general anesthesia necessary for my child's dental treatment?

Answer:
General anesthesia is not automatically necessary for every child's dental treatment, but it can be the right choice in certain situations. The decision depends on your child's age, anxiety level, medical history, and how extensive the dental work is.

When it is usually needed

General anesthesia is typically considered a "last resort" option, used when regular local numbing and lighter sedation are not enough to safely and comfortably complete treatment. Common reasons include:

- Very young children who cannot understand or follow instructions well enough to sit still.
- Children who need a lot of work (for example many cavities or full-mouth rehabilitation) that would otherwise require several long visits.
- Children with significant anxiety, strong gag reflex, or special health or behavioral needs that make treatment while awake unsafe or impossible.

Alternatives to general anesthesia

In many cases, dentists can use local anesthesia (numbing injections) plus behavior techniques or lighter forms of sedation so the child stays awake but more relaxed. Options can include:

- Nitrous oxide ("laughing gas") to reduce anxiety.
- Oral or IV sedation in appropriate settings, depending on the child's needs and the provider's training.

Safety and risks

When provided by trained professionals in a proper setting, general anesthesia for children's dental work is generally considered safe, with continuous monitoring of breathing, heart rate, and oxygen. However, it does carry more risk and a longer recovery time than local anesthesia or mild sedation, so the benefits and risks should be weighed carefully in your child's specific case.

How to decide for your child

The best next step is to ask your child's dentist:

- Why general anesthesia is being recommended instead of lighter options.
- Exactly what treatment is planned and whether it could be broken into shorter visits with local anesthesia or mild sedation.

If you still feel unsure, it is reasonable to ask for a second opinion from another pediatric dentist or anesthesiologist, bringing your child's treatment plan and medical history to that visit.

1. https://www.kitsfamilydental.com/is-it-really-necessary-to-use-general-anesthesia-for-kids-dental-work/
2. https://pmc.ncbi.nlm.nih.gov/articles/PMC3341905/
3. https://pmc.ncbi.nlm.nih.gov/articles/PMC4904485/
4. https://motownchildrens.com/why-general-anesthesia-is-crucial-for-full-mouth-rehabilitation-in-young-children/
5. https://polkadotdental.com/children-and-general-anesthesia-faqs/
6. https://www.nth.nhs.uk/resources/childrens-dental-treatment-under-general-anaesthetic/
7. https://www.joyfulsmilespediatricdentistry.com/general-anesthesia-crucial-pediatric-dentistry/
8. https://www.caringtreechildrensdentistry.com/blog/how-safe-is-general-anesthesia-in-dental-procedures-for-children/
9. https://everykidsmilesdental.com/wp-content/uploads/2025/10/sedation_information_migrated.pdf
10. https://www.1stsmiles.com/blog-posts/when-is-general-anesthesia-recommended-for-kids-at-the-dentist-and-what-we-do-instead-at-first-smiles

Q8. Who will give my child anesthesia?

Answer:
For most children, anesthesia is given and closely watched by specialists whose entire job is keeping kids safe and comfortable during procedures.
Main anesthesia specialist
The primary person responsible is usually an anesthesiologist, a medical doctor (or sometimes a dentist-physician) with several years of extra training in anesthesia and often additional specialization in pediatric anesthesia. This doctor meets you before the procedure, reviews your child's health, explains the plan, and then stays involved throughout the procedure to adjust medications and handle any emergencies if they arise.
Other professionals involved
In many hospitals and surgery centers, a certified registered nurse anesthetist (CRNA) or nurse anesthetist may give and monitor anesthesia, usually working in a team with an anesthesiologist supervising or available if needed. For some dental or office procedures, trained anesthesia professionals may include dentist anesthesiologists or oral and maxillofacial surgeons who hold special permits to provide deep sedation or general anesthesia in children.
What this means for your child
Regardless of the exact title, your child's anesthesia is provided by clinicians with specific training in anesthesia and in caring for children, and your child's heart rate, blood pressure, breathing, and oxygen levels are monitored continuously. It is always appropriate to ask, "Who exactly will be giving and monitoring my child's anesthesia, and what pediatric training do they have?" so you know who is responsible and can feel more confident about your child's care.

1. https://www.radiologyinfo.org/en/info/safety-pediatric-sedation
2. https://www.med.unc.edu/surgery/pedssurgery/forpatients/faqs/anesthesia/
3. https://childrens.dartmouth-health.org/your-visit/anesthesia-and-sedation-faqs
4. https://www.healthychildren.org/English/healthy-living/oral-health/Pages/Anesthesia-or-Sedation-for-Your-Childs-Dental-Work.aspx
5. https://mydoctor.kaiserpermanente.org/ncal/structured-content/understanding-anesthesia-for-your-childs-procedure-2791259
6. https://mydoctor.kaiserpermanente.org/ncal/article/information-about-pediatric-anesthesia-575533
7. https://health.ucdavis.edu/treatments/pediatric-anesthesia
8. https://www.balboapediatrics.com/safety/Anesthesia-and-Your-Child
9. https://med.stanford.edu/pedsanesthesia/patients/brain-dev-faqs.html
10. https://www.ucsfbenioffchildrens.org/clinics/pediatric-prepare-clinic

Q9. What if my child won't cooperate for anesthesia?

Answer:
If a child refuses to cooperate for anesthesia, the team usually focuses on calming fear, adjusting the plan, and only rarely postponing or cancelling the procedure. Parents play a big role in helping the child feel safe and understood.
Why children resist
Children often refuse anesthesia because they are afraid of pain, separation from parents, loss of control, or not understanding what will happen. Younger children may show this as crying,

clinging, or trying to escape rather than calmly saying “no.”

What the team can do

The anesthesia and surgical team can:

- Spend extra time explaining what will happen in simple, honest language and answering questions.
- Offer options like flavored masks, distraction (videos, toys, music), or letting a parent stay during mask induction if appropriate.
- Use premedication (for example, a sedative given by mouth or nose) to reduce anxiety in very distressed children when it is safe and indicated.

When the procedure is urgent vs. elective

For urgent or emergency surgery, the team may need to proceed despite distress, using the safest combination of medication, staff support, and gentle but firm limits. For elective procedures, if the child is extremely distressed and cannot be calmed, the team may recommend delaying, involving a child-life specialist, psychologist, or desensitization sessions before trying again.

How parents can help beforehand

- Talk about the procedure ahead of time using age-appropriate, truthful explanations, avoiding scary details but not pretending “nothing” will happen.
- Practise at home with “medical play” (stuffed animals, toy masks, pretend monitors) and step-by-step “fear ladders” so the child gradually gets used to each part (talking about it, visiting the hospital, trying on a mask).
- Model calm behavior, bring a comfort item, and agree on small rewards for cooperation with each step.

When to ask for extra support

If a child has intense fear of medical procedures, past traumatic experiences, neurodivergence, or anxiety disorders, ask the clinic in advance about child-life services, psychology support, and plans for sedation or anesthesia tailored to your child. If you feel the child is too distressed on the day, speak up; decisions about proceeding, postponing, or changing the plan should be made together with the team, balancing the urgency of treatment with the child’s emotional well-being.

1. https://onlinelibrary.wiley.com/doi/10.1111/j.1460-9592.2009.03010.x
2. https://ucalgary.ca/news/if-your-child-afraid-or-refusing-medical-procedure-heres-how-help
3. https://kidshealth.org/en/parents/anesthesia.html
4. https://www.carlislepediatric.com/safety/Anesthesia-and-Your-Child
5. https://pmc.ncbi.nlm.nih.gov/articles/PMC5605147/
6. https://pubmed.ncbi.nlm.nih.gov/19422599/
7. https://www.reddit.com/r/Parenting/comments/1gy888o/anxiety_over_child_being_put_under_general/
8. https://www.reddit.com/r/Parenting/comments/13j5aj5/terrified_about_my_sons_upcoming_dental_procedure/
9. https://www.rch.org.au/kidsinfo/fact_sheets/Preparing_your_child_for_surgery_general_anaesthesia/
10. https://platinumpediatricdentistry.com/what-should-i-do-if-my-child-is-not-cooperative-for-dental-treatment/

Q10. What if my child is allergic to anesthesia?

Answer:
Allergic reactions to anesthesia in children are possible but rare, and there are usually safe alternatives even if a true allergy exists. The most important step is to talk with your child's doctors early so an anesthesiologist can plan a safer approach tailored to your child.

How common is this?
True immune allergies to anesthetic drugs in children are unusual; much more often, kids are allergic to things given around surgery, like antibiotics or latex, rather than the anesthetic itself. Many "allergies" recorded in charts turn out to be side effects (like nausea or agitation) rather than true allergies, which an anesthesiologist can help sort out by asking exactly what happened before.

What does an allergy look like?
Mild reactions can start with rash, flushing, runny nose, or feeling unwell, and can progress to hives, swelling, or wheezing. Severe reactions (anaphylaxis) can cause trouble breathing, low blood pressure, fast heart rate, or collapse and require immediate treatment in an operating room or intensive care setting.

What if my child has reacted before?
If your child has ever had a concerning reaction to anesthesia or a drug (rash, breathing problems, swelling, or collapse), the next step is referral to an allergist/anesthesia clinic for evaluation before any future surgery. Testing may include blood work and skin tests to identify the specific trigger and to outline which anesthetic and related drugs are safest to use next time.

How doctors reduce the risk
Before surgery, the team will review all allergies, past anesthetics, other medical conditions (like asthma or eczema), and any family history of anesthesia problems. With that information, the anesthesiologist can avoid suspected drugs, choose alternative medications, sometimes give premedication (such as antihistamines or steroids), and closely monitor your child throughout the procedure.

What you should do as a parent

- Tell every provider exactly what happened during any past reaction (timing, symptoms, and treatment), not just the name of the drug.
- Ask for a written allergy/anesthesia plan from the allergist or anesthesia clinic and bring it to any future hospital or surgery center.
- If your child has an emergency surgery without prior testing, remind staff of any suspected allergies so they can choose safer alternatives and monitor especially closely.

If you are worried that your child may be allergic to anesthesia, contact your pediatrician or a pediatric anesthesiologist soon and ask for a formal allergy and anesthesia risk evaluation before any planned procedure.

1. https://nortonchildrens.com/news/avoiding-anesthesia-complications-for-kids/
2. https://www.stonybrookmedicine.edu/patientcare/anesthesia/pediatric
3. https://www.apsf.org/patient-guide/can-i-be-allergic-to-anesthesia/
4. https://www.medicalnewstoday.com/articles/anesthesia-allergy
5. https://www.healthline.com/health/anesthesia-allergy
6. https://pmc.ncbi.nlm.nih.gov/articles/PMC6137173/

7. https://www.wsh.nhs.uk/CMS-Documents/Patient-leaflets/AnaestheticDepartment/6121-1-Risks-Associated-with-your-Anaesthetics-Anaphylaxis.pdf
8. https://kidshealth.org/en/parents/anesthesia.html
9. https://www.verywellhealth.com/anesthesia-allergy-5220938

Q11. Will my child need any evaluation before anesthesia?

Answer:
Yes, children typically require a preoperative evaluation before anesthesia to assess risks and ensure safety. This process helps tailor the anesthesia plan to your child's specific health needs.
Evaluation Components
A standard preanesthesia assessment includes a review of your child's medical history, birth history, and any prior anesthesia experiences, along with a physical exam focusing on airway, heart, lungs, and overall condition. For routine procedures like tonsillectomies, it may only involve history discussion and exam, but complex cases could add tests.
Possible Tests
Depending on health factors, tests such as blood work, ECG, echocardiograms, lung function tests, or imaging like X-rays may be ordered to check heart, lung, or blood compatibility issues. These are not routine for healthy kids but target symptoms like murmurs or prematurity complications.
Timing and Process
Evaluations often occur days before surgery via nurse calls, clinic visits, or on the procedure day, scheduled by the surgeon's office. The anesthesiologist meets you to discuss allergies, medications, and family history.

1. https://www.childrens.com/specialties-services/specialty-centers-and-programs/surgical-services/preoperative-clinic
2. https://www.massgeneral.org/children/anesthesia/pediatric-general-anesthesia
3. https://www.aafp.org/pubs/afp/issues/2022/0600/p640.html
4. https://madeforthismoment.asahq.org/preparing-for-surgery/prep/preparing-for-surgery-kids-checklist/
5. https://health.choc.org/10-questions-anesthesia/
6. https://www.ncbi.nlm.nih.gov/books/NBK559198/
7. https://www.ucsfbenioffchildrens.org/your-stay/your-childs-surgery-what-to-expect
8. https://www.advocarecornerstonepeds.com/safety/Anesthesia-and-Your-Child
9. https://nrpeds.com/Resources/Medical-Conditions/Anesthesia-and-Your-Child

https://www.childrensal.org/services/anesthesia-pre-admit-screening-service-apass

Q12. Can my child have anesthesia with a cold?

Answer:
Whether a child can safely receive anesthesia with a cold depends on symptom severity. Mild cases limited to clear nasal discharge without fever or lethargy often allow proceeding with

adjusted care. Severe symptoms like wheezing, fever, or purulent secretions typically require postponing for 2-4 weeks.

Key Risk Factors

Children with colds face higher chances of perioperative respiratory issues, such as coughing, airway spasm, or low oxygen, due to excess secretions and sensitive airways. Factors increasing complications include asthma history, heavy secretions, breathing tube use, or airway surgery. These events remain mild and manageable for experienced anesthesiologists.

When to Postpone

Postpone if the child has fever, wheezing, thick secretions, or feels unwell; wait at least 2 weeks after symptoms resolve, or 4 weeks for bacterial infections. Airways may stay sensitive for weeks post-cold. Always consult the anesthesiologist and surgeon for elective procedures.

Care Adjustments

For mild colds, options include pre-op inhalers like salbutamol, IV fluids, secretion suctioning, and avoiding intubation with masks or laryngeal masks instead. Anesthesia does not typically worsen or prolong the cold. Continuous oxygen monitoring ensures safety.

1. https://pubmed.ncbi.nlm.nih.gov/22499163/
2. https://www2.pedsanesthesia.org/patiented/uri.iphtml
3. https://pmc.ncbi.nlm.nih.gov/articles/PMC12567271/
4. http://www2.pedsanesthesia.org/patiented/uri.iphtml
5. https://anesthesiaboise.com/faqs-pediatrics
6. https://health.clevelandclinic.org/is-anesthesia-safe-for-kids
7. https://pubmed.ncbi.nlm.nih.gov/24063129/
8. https://www.hss.edu/health-library/conditions-and-treatments/why-do-anesthesiologists-care-about-a-common-cold
9. https://www.aegisanesthesiapartners.com/patient-information/faq-frequently-asked-questions-pediatric-anesthesia/
10. https://www.healthychildren.org/English/health-issues/conditions/treatments/Pages/Procedures-With-Anesthesia.aspx

Q13. When does my child need to stop eating or drinking before anesthesia?

Answer:

Children typically need to stop eating solid foods and dairy about 8 hours before anesthesia to prevent aspiration risks during the procedure.

Age-Specific Guidelines

Infants under 6 months should stop breast milk 4 hours prior and formula or solids 6 hours prior, while clear liquids can continue up to 2 hours before arrival.

For children over 1 year, solids and non-clear liquids stop 8 hours before, breast milk or formula 6 hours before, and clear liquids like water or apple juice up to 2 hours prior.

Clear Liquids Defined

Allowed clear liquids include water, apple juice without pulp, Pedialyte, or Gatorade until the 2-hour cutoff to keep kids hydrated safely.

Always confirm exact times with your child's anesthesiologist, as guidelines can vary by hospital

or procedure.

1. https://www.massgeneral.org/children/anesthesia/preparing-your-child-for-general-anesthesia
2. https://www.uclahealth.org/medical-services/anesthesiology/patient-resources/when-stop-eating-and-drinking
3. https://www.uclahealth.org/medical-services/pediatric-neurosurgery/resources-families/guide-preparing-surgery/day-before-surgery/eating-drinking-restrictions-before-surgery
4. https://uihc.org/childrens/educational-resources/fasting-guidelines-children
5. https://kidshealth.org/en/parents/anesthesia.html
6. https://www.nationwidechildrens.org/family-resources-education/700childrens/2018/07/npo-eating-and-drinking-before-surgery
7. https://www.rothschillermd.com/preparing-your-child-for-anesthesia/
8. https://pubmed.ncbi.nlm.nih.gov/10201666/
9. https://www.rch.org.au/kidsinfo/fact_sheets/Preparing_your_child_for_surgery_fasting/
10. https://www.childrens.com/specialties-services/specialty-centers-and-programs/anesthesiology/preparing-your-child-for-anesthesia

Q14. If my child is breastfeeding, do we need to stop before their anesthesia?

Answer:
No, breastfeeding does not need to stop entirely before a child's anesthesia, but feedings must be timed according to pre-surgery fasting guidelines to minimize risks like aspiration.

Fasting Guidelines
Breast milk is typically allowed up to 4 hours before surgery for infants, as it empties from the stomach faster than formula or solids. Clear liquids may be permitted until 2 hours prior, but always confirm with the anesthesiologist or surgical team, as protocols can vary by hospital and child's age. Solid foods or formula usually stop 6 hours before arrival.

Resuming Breastfeeding
Breastfeeding can generally resume once the child wakes from anesthesia and is alert, often right in the recovery area, since most anesthetic drugs pass into breast milk in negligible amounts safe for infants. Exceptions might apply for certain medications like codeine or tramadol, but standard anesthetics like propofol or fentanyl have low relative infant doses under 10%.

Key Precautions
Discuss the child's feeding schedule and any medications with the care team beforehand to avoid delays in surgery. Pain management post-op favors non-opioids when possible to support quick resumption of breastfeeding.

1. https://www.asahq.org/standards-and-practice-parameters/statement-on-resuming-breastfeeding-after-anesthesia
2. https://kellymom.com/bf/can-i-breastfeed/illness-surgery/baby-surgery/
3. https://www.chop.edu/pages/surgery-and-breastfeeding-infant
4. https://www.texaschildrens.org/sites/default/files/PresurgeryPacket_vF.pdf
5. https://www.uclahealth.org/medical-services/anesthesiology/patient-resources/when-stop-

eating-and-drinking
6. https://innovationdistrict.childrensnational.org/breast-feeding-anesthesia-and-analgesics-whats-safe/
7. https://infantrisk.com/content/breastfeeding-surgery-and-anesthesia
8. https://www.stanfordchildrens.org/en/topic/default?id=surgery-and-the-breastfeeding-infant-90-P03009
9. https://abm.memberclicks.net/assets/DOCUMENTS/PROTOCOLS/15-analgesia-and-anesthesia-protocol-english.pdf
10. https://www.urmc.rochester.edu/MediaLibraries/URMCMedia/breastfeeding/documents/breastfeeding-anesthesia-brochure.pdf

Q15. What should I do if my child takes daily medicines before anesthesia?

Answer:
Contact your child's anesthesiologist or surgeon immediately if your child takes daily medicines before anesthesia, as guidelines vary by medication type and procedure. Most routine prescription medications can often continue with a small sip of water on the day of surgery, but specific drugs like blood thinners, NSAIDs (e.g., ibuprofen), or aspirin require stopping days in advance to reduce bleeding risks. Always bring a full list of medications, dosages, and instructions to the hospital.

Key Medication Rules
Daily prescriptions such as those for heart conditions, blood pressure, seizures, or inhalers are typically allowed unless directed otherwise, taken at least two hours before arrival. Stop NSAIDs like ibuprofen or naproxen 2-7 days prior, and herbal supplements one week before. Acetaminophen (Tylenol) is usually permitted up to the night before.

Fasting and Prep Steps
Follow facility-specific fasting: no solid food after midnight, clear liquids up to 2 hours before, to prevent vomiting under anesthesia. Inform the medical team of all medicines during pre-op calls, and confirm adjustments for conditions like diabetes. Delaying or canceling may occur if instructions aren't followed.

When to Call Ahead
Discuss any changes in your child's health or meds promptly, as even routine daily drugs need personalized approval. This ensures safety tailored to your child's needs.

1. https://www.childrens.com/specialties-services/specialty-centers-and-programs/anesthesiology/preparing-your-child-for-anesthesia
2. https://medlineplus.gov/ency/patientinstructions/000637.htm
3. https://www.healthychildren.org/English/health-issues/conditions/treatments/Pages/Anesthesia-and-Children-The-Day-of-the-Procedure.aspx
4. https://www.stlouischildrens.org/visit-us/preparing-for-your-visit/planning-for-surgery/before-you-arrive-for-surgery
5. https://www.chop.edu/centers-programs/kohl-s-gi-nutrition-and-diagnostic-center/pre-procedure-prep
6. https://www.uclahealth.org/sites/default/files/documents/Peds-Preop-Instructions_0.pdf

7. https://www.ecuhealth.org/services/surgery/surgicenter-what-to-expect/preparing-children-for-surgery/
8. https://www.med.unc.edu/anesthesiology/patients/pediatrics-2/
9. https://www.choa.org/parent-resources/surgery/what-to-do-before-surgery
10. https://www.chp.edu/-/media/chp/patient-procedures/documents/anesthesiology/general-anesthesia-with-caudal-anesthesia.pdf

Q16. If my child has asthma, diabetes, or other health issues, do I need to do anything special before anesthesia?

Answer:
Yes, special preparations are often needed before anesthesia for children with asthma, diabetes, or other health issues to minimize risks like bronchospasm, blood sugar fluctuations, or complications from comorbidities.

Asthma Considerations
Children with asthma should continue controller inhalers on the day of surgery and use albuterol prophylactically beforehand to reduce intubation-related bronchospasm. Bring all inhalers to the facility, and consult the pediatrician if wheezing or recent symptoms occur, as steroids may be recommended days prior. Optimize lung function by avoiding recent respiratory infections.

Diabetes Management
Diabetic children require an individualized plan, often involving consultation with an endocrinologist for insulin or medication adjustments to maintain stable glucose levels perioperatively. Inform staff upon arrival and follow specific instructions on morning doses, as uncontrolled diabetes can delay surgery.

General Steps
Disclose full medical history, medications, and allergies during pre-anesthesia evaluation, which may include tests or exams. Follow strict fasting guidelines (e.g., no clear liquids 2 hours prior) and take approved routine meds with sips of water unless directed otherwise. For other issues like seizures or metabolic conditions, preoperative optimization and specialist input are advised.

1. https://www.aafp.org/pubs/afp/issues/2022/0600/p640.html
2. https://www.childrens.com/specialties-services/specialty-centers-and-programs/anesthesiology/preparing-your-child-for-anesthesia
3. https://www.metrohealth.org/en/medical-services/surgery/preparing/
4. https://www.umms.org/ummc/health-services/perioperative-services/pre-surgery-information
5. https://www.archildrens.org/-/media/Files/for-healthcare-professionals/pediatric-guidelines/Common_Pediatric_Preoperative_Concerns.pdf
6. https://kidshealth.org/HumanaSouthCarolina/en/parents/anesthesia.html
7. https://www.healthychildren.org/English/health-issues/conditions/treatments/Pages/Anesthesia-and-Children-The-Day-of-the-Procedure.aspx
8. https://www.pedsurglibrary.com/apsa/view/Pediatric-Surgery-NaT/829462/all/Anesthesia
9. https://madeforthismoment.asahq.org/anesthesia-101/types-of-anesthesia/anesthesia-risks/

10. https://www.ncbi.nlm.nih.gov/books/NBK559198/

Q17. When should my child and I arrive at the hospital on surgery day for anesthesia?

Answer:
The surgeon's office or a nurse will specify the exact arrival time for your child's surgery, typically communicated a day or two in advance.

Typical Arrival Window
Arrival is usually set 1 to 2 hours before the scheduled surgery start time to allow for check-in, vital checks, anesthesia discussions, and pre-op preparations. This timeframe accounts for safety verifications like confirming your child's identity, procedure details, and health status. Being late risks delaying or rescheduling the surgery.

Anesthesia Preparation
The anesthesia team meets you on arrival to review the plan, often including a physical exam and consent signing. For children, parents may accompany them into the operating room until anesthesia takes effect via mask or IV. Expect the full day to involve waiting, so arrange childcare and bring comfort items like a favorite toy.

1. https://medlineplus.gov/ency/patientinstructions/000579.htm
2. https://childrens.dartmouth-health.org/your-visit/surgery-faqs-parents
3. https://www.carolinapediatricsurgery.com/the-day-of-surgery
4. https://www.chla.org/gen-surg-pre-op-instructions
5. https://childrenswi.org/teaching-sheet/surgery/surgery-at-chw-packet-1107-1018-11911061
6. https://www.stanfordchildrens.org/en/child-visit/preop-instructions.html
7. https://www.healthychildren.org/English/health-issues/conditions/treatments/Pages/Anesthesia-and-Children-The-Day-of-the-Procedure.aspx
8. https://mydoctor.kaiserpermanente.org/ncal/structured-content/inhalation-induction-for-children-571402
9. https://nrpeds.com/Resources/Medical-Library/Safety/Anesthesia-and-Your-Child
10. https://www.childrens.com/specialties-services/specialty-centers-and-programs/anesthesiology/preparing-your-child-for-anesthesia

Q18. Can my child visit the surgery and recovery areas before anesthesia?

Answer:
No, children typically cannot visit surgery and recovery areas before anesthesia due to strict sterile protocols and safety rules in hospitals.

Preoperative Access
Parents often accompany children to a preoperative holding area, where they meet the anesthesiologist to review plans, but actual operating rooms remain off-limits until induction. Some hospitals allow pre-surgery tours of surgical units during planning visits, but not on the

procedure day before anesthesia.
Day-of Restrictions
Sterile environments prevent entry into surgery or recovery zones beforehand; parents may wear protective suits to stay until the child falls asleep, then leave. Recovery visits occur post-surgery once anesthesia wears off.
Preparation Tips
Discuss anxieties with the care team during pre-op assessments, which can happen days ahead. Child life specialists may provide play activities in waiting areas to ease fears.

1. https://www.chop.edu/patients-and-visitors/guide-your-childs-surgery/day-your-childs-surgery
2. https://www.childrens.com/specialties-services/specialty-centers-and-programs/anesthesiology/faq
3. https://www.chop.edu/centers-programs/division-pediatric-general-thoracic-and-fetal-surgery/your-childs-surgery
4. https://www.archildrens.org/blog/preparing-your-child-for-surgery
5. https://www.chp.edu/our-services/anesthesiology/patient-procedures/parental-presence
6. https://www.massgeneral.org/children/visit/surgery-visits
7. https://childrenswi.org/teaching-sheet/surgery/getting-ready-for-surgery-at-childrens-hospital-of-wisconsin
8. https://mydoctor.kaiserpermanente.org/mas/news/7-easy-ways-to-prepare-your-child-for-anesthesia-1759132
9. https://greenvillekidsdental.com/general-anesthesia-instructions
10. https://nrpeds.com/Resources/Medical-Conditions/Anesthesia-and-Your-Child

Q19. Will my child be awake or know what’s happening during anesthesia?

Answer:
Under general anesthesia, your child will be unconscious and unaware of what's happening during the procedure.
Induction Phase
Children often receive anesthesia via a mask or IV, falling asleep within 60-90 seconds. They may show brief movements, eye rolling, or noisy breathing as they drift off, but these are normal and not remembered.
During Procedure
The anesthesiologist monitors vital signs and adjusts medication to ensure your child remains fully asleep and pain-free throughout. Awareness is extremely rare in children under proper care.
Recovery Phase
Waking varies; some children stir quickly, while others stay groggy for minutes to hours and may cry or seem confused (emergence delirium), but they typically remember nothing.

1. https://mydoctor.kaiserpermanente.org/ncal/structured-content/understanding-anesthesia-for-your-childs-procedure-2791259
2. https://www.stonybrookmedicine.edu/patientcare/anesthesia/pediatric
3. https://childrens.dartmouth-health.org/your-visit/anesthesia-and-sedation-faqs

4. https://www.massgeneral.org/children/anesthesia/pediatric-general-anesthesia
5. https://www.rileychildrens.org/health-info/surgical-anesthesia
6. https://www.uclahealth.org/hospitals/mattel/preparing-surgery/checking-surgery/what-know-about-anesthesia
7. https://pmc.ncbi.nlm.nih.gov/articles/PMC3026393/
8. https://kidshealth.org/en/parents/anesthesia.html
9. https://www.chp.edu/our-services/anesthesiology/patient-procedures/parental-presence
10. https://www.akronchildrens.org/kidshealth/en/parents/anesthesia-basics.html

Q20. Will my child feel any pain while under anesthesia?

Answer:
No, your child will not feel pain while under anesthesia. Anesthesia is specifically designed to block pain signals and induce unconsciousness or deep sedation during procedures. Reliable medical sources confirm that children remain completely pain-free throughout the process.

Types of Anesthesia
General anesthesia renders the child fully unconscious and insensitive to pain, often used for surgeries requiring stillness. Local or regional anesthesia numbs specific areas, preventing discomfort there while the child may stay partially awake, though it's commonly combined with sedation. Most pediatric cases prioritize safety, with providers tailoring the approach to minimize any awareness or sensation.

Post-Anesthesia Recovery
After the procedure, children recover in a monitored area, where they might feel groggy, sore at the surgical site, or experience temporary nausea, but these are managed separately from intraoperative pain. Emergence delirium—temporary agitation upon waking—is common in young kids but unrelated to pain and resolves quickly. Pain control post-procedure uses medications like acetaminophen or regional blocks to ensure comfort.

1. https://kidshealth.org/en/parents/anesthesia.html
2. https://www.smilefirstdental.com/blog/will-my-child-feel-pain-dental-sedation
3. https://www.uclahealth.org/hospitals/mattel/preparing-surgery/checking-surgery/what-know-about-anesthesia
4. https://pmc.ncbi.nlm.nih.gov/articles/PMC6761783/
5. https://childrens.dartmouth-health.org/your-visit/anesthesia-and-sedation-faqs
6. https://mydoctor.kaiserpermanente.org/ncal/structured-content/understanding-anesthesia-for-your-childs-procedure-2791259
7. https://www.stonybrookmedicine.edu/sites/default/files/40259824.pdf
8. https://www.hss.edu/health-library/conditions-and-treatments/effects-of-anesthesia-on-children
9. https://www.med.unc.edu/anesthesiology/patients/pediatrics-2/
10. https://www.uclahealth.org/medical-services/anesthesiology/anesthesia-and-child-development

Q21. Will someone watch my child the whole time during anesthesia to keep them safe?

Answer:
Yes, trained medical professionals continuously monitor children during anesthesia to ensure their safety.
Monitoring Practices
An anesthesiologist or certified nurse anesthetist stays with the child throughout the procedure, tracking vital signs like heart rate, blood pressure, breathing, oxygen levels, and temperature. Devices such as monitors and alarms provide real-time data, allowing immediate adjustments to medications or interventions if needed. This constant vigilance applies whether under general anesthesia, where the child is unconscious, or lighter sedation.
Team Responsibilities
The anesthesia team, often including technicians or assistants, focuses solely on the child's well-being, using child-specific equipment and protocols. Parents typically cannot remain in the operating room to avoid distractions, but staff update them via waiting areas or communication systems. Safety is enhanced by pre-procedure assessments, like fasting and health checks, to minimize risks.
Safety Outcomes
Pediatric anesthesia has improved significantly, with low complication rates when handled by specialists, though risks vary by age, health, and procedure. Continuous monitoring catches issues like airway problems early, contributing to overall low injury risk for healthy children.

1. https://www.radiologyinfo.org/en/info/safety-pediatric-sedation
2. https://www.emuhealth.com/blog/1361720-from-what-age-why-take-a-child-for-general-anesthesia-dentistry/
3. https://www.healthychildren.org/English/health-issues/conditions/treatments/Pages/Anesthesia-Safety-Infants-Toddlers-Parent-FAQs.aspx
4. https://www.aegisanesthesiapartners.com/patient-information/faq-frequently-asked-questions-pediatric-anesthesia/
5. https://www.baby-blocks.com/parents-detail/parental-presence-in-the-operating-room
6. https://www.chp.edu/our-services/anesthesiology/patient-procedures/parental-presence
7. https://pmc.ncbi.nlm.nih.gov/articles/PMC7699483/
8. https://answers.childrenshospital.org/what-parents-should-know-pediatric-anesthesia/
9. https://childlifeoncall.com/blog/ppi/
10. https://www.texaschildrens.org/sites/default/files/uploads/documents/Anesthesia_final2022.pdf

Q22. Can I stay with my child during the anesthesia process?

Answer:
Whether parents can stay with their child during anesthesia depends on the hospital's policy, the anesthesiologist's discretion, and the child's age or procedure type. Many facilities allow one parent to accompany the child into the operating room until anesthesia takes effect, often via mask, to reduce anxiety. Parents must then leave before the surgery begins.
Key Policies
Policies vary by institution. For example, some children's hospitals permit presence for toddlers until asleep, while others restrict it for infants under one year, emergencies, or pregnant parents.

Dental sedation may depend on the provider's rules and child comfort.
Reasons for Limits
Anesthesia induction requires full staff focus on the child, and parental distress could distract or require additional care. Space constraints and infection control also play roles during procedures.
Preparation Tips
Discuss with the anesthesiologist beforehand; they decide based on safety. Comfort your child with touch, voice, or songs if allowed, and be ready to exit quickly if asked.

1. https://www.med.unc.edu/surgery/pedssurgery/forpatients/faqs/before/
2. https://www.mountsinai.org/care/pediatrics/services/anesthesia/for-parents
3. https://www.massgeneral.org/children/anesthesia/pediatric-general-anesthesia
4. https://www.childrenshospitalvanderbilt.org/patient-resource/more-information-about-anesthesia-services
5. https://www.marshfieldpediatricdentistry.com/post/can-i-stay-with-my-child-during-a-dental-sedation-procedure
6. https://www.chp.edu/our-services/anesthesiology/patient-procedures/parental-presence
7. https://childrens.dartmouth-health.org/your-visit/surgery-faqs-parents
8. https://www.aegisanesthesiapartners.com/patient-information/faq-frequently-asked-questions-pediatric-anesthesia/
9. http://www.socaldentalsedation.com/blog/anesthesia/why-cant-i-be-in-the-procedure-room-with-my-child-under-anesthesia/
10. https://www.baby-blocks.com/parents-detail/parental-presence-in-the-operating-room

Q23. How the anesthesia will be given to my child?

Answer:
Anesthesia for children is typically administered by a specialized pediatric anesthesiologist to ensure safety and comfort during procedures like surgery. The method chosen depends on your child's age, anxiety level, medical history, and the type of procedure.
Common Methods
General anesthesia, which makes your child fully unconscious, is most often used. It can be delivered via inhalation through a flavored mask (like cherry or bubble gum scents for younger kids) or injected through an IV line in a vein. For mask induction, common in children under 13, your child breathes the gas until asleep, avoiding initial needle sticks.
IV induction suits older children or those with IVs already placed, often after numbing cream to reduce discomfort. Sedatives may be given first by mouth, nose, or injection if your child is anxious.
What to Expect
Monitors track heart rate, oxygen, and breathing throughout. After falling asleep, an IV or breathing tube may be added for support. Your anesthesiologist will discuss the best plan beforehand, and parents often stay until induction. Recovery occurs in a monitored room as effects wear off.

1. https://www.chop.edu/patients-and-visitors/guide-your-childs-surgery/day-your-childs-surgery/about-anesthesia
2. https://www.childrenshospitalvanderbilt.org/patient-resource/more-information-about-

anesthesia-services
3. https://childrens.dartmouth-health.org/your-visit/anesthesia-and-sedation-faqs
4. https://www.mountsinai.org/care/pediatrics/services/anesthesia/about
5. https://www.rch.org.au/kidsinfo/fact_sheets/Preparing_your_child_for_surgery_general_anaesthesia/
6. https://www.nationwidechildrens.org/conditions/health-library/types-of-anesthesia-for-children
7. https://healthy.kaiserpermanente.org/health-wellness/health-encyclopedia/he.learning-about-general-anesthesia-in-children.acm5239
8. https://www.youtube.com/watch?v=oncKQcMubgY
9. https://greenvillekidsdental.com/general-anesthesia-instructions
10. https://www.radiologyinfo.org/en/info/safety-pediatric-sedation

Q24. Will my child need help breathing during anesthesia?

Answer:
Whether a child needs help breathing during anesthesia depends on the type of procedure, the child's age, health, and the anesthesiologist's assessment. General anesthesia often requires breathing support to ensure safety, but it's not always a tube—many cases use a mask initially.

Breathing Support Methods

Children typically receive anesthesia via inhaled gas through a mask, which supports breathing without a tube for shorter or simpler surgeries. For longer procedures or those involving the airway, a breathing tube (endotracheal tube) or laryngeal mask airway may be inserted after the child is asleep to maintain an open airway and deliver oxygen effectively.

Factors Influencing Need

Premature infants, those with respiratory issues, or surgeries like airway reconstruction more commonly require tubes, sometimes even post-surgery in ICU. Healthy children under general anesthesia for routine operations (e.g., ear tubes) often manage with mask ventilation alone, minimizing invasive aids.

Safety Monitoring

Anesthesia teams continuously monitor oxygen levels, heart rate, and breathing, adjusting support as needed to prevent complications like low oxygen. Preoxygenation and careful induction reduce risks, and tubes are removed before waking.

1. https://www.chop.edu/patients-and-visitors/guide-your-childs-surgery/day-your-childs-surgery/about-anesthesia
2. https://www.uclahealth.org/hospitals/mattel/preparing-surgery/checking-surgery/what-know-about-anesthesia
3. https://www.cincinnatichildrens.org/health/a/anesthesia-airway
4. https://www.foxcitiesaha.com/surgeries/pediatric/
5. https://www.childrenshospitalvanderbilt.org/patient-resource/more-information-about-anesthesia-services
6. https://www.anesthesiologynews.com/Review-Articles/Article/08-19/10-Common-Pediatric-Airway-Problems-And-Their-Solutions/55657?sub=B9BFD2B22AAB91C738BEFA44BD6987A27AB424466CD7E097

40FF4A4786780
7. http://www2.pedsanesthesia.org/patiented/uri.iphtml
8. https://www.radiologyinfo.org/en/info/safety-pediatric-sedation
9. https://kidshealth.org/en/parents/anesthesia.html
10. https://www.choa.org/medical-services/radiology/sedation-and-anesthesia

Q25. Will my child be sedated before getting anesthesia?

Answer:
Sedation before anesthesia is often used for children to reduce anxiety.
When Sedation Occurs
Sedation may be given if the child appears nervous or anxious prior to anesthesia induction. It is typically administered as a liquid to swallow, through the nose, or via injection, depending on age and needs. This helps calm the child without full unconsciousness until anesthesia begins.
Factors Influencing Use
The decision depends on the child's age, anxiety level, medical condition, and hospital protocols. Not all children receive it; some go straight to anesthesia, especially if calm. Always consult the anesthesiologist for your child's specific case, as practices vary.
Preparation Notes
Children must fast before sedation or anesthesia to prevent vomiting risks. Guidelines include no solids for 8 hours, clear fluids up to 2 hours prior. Discuss any allergies or prior reactions with the care team.

1. https://www.nationwidechildrens.org/specialties/pediatric-surgery/surgery--clinic-locations/main-or-inpatient--outpatient-surgery/the-day-of-surgery
2. https://www.healthychildren.org/English/health-issues/conditions/treatments/Pages/Anesthesia-and-Children-The-Day-of-the-Procedure.aspx
3. https://www.mountsinai.org/care/pediatrics/services/anesthesia/about
4. https://www.radiologyinfo.org/en/info/safety-pediatric-sedation
5. https://www.chop.edu/services/pediatric-sedation-unit
6. https://www.chp.edu/our-services/sedation-services/preparing-for-sedation
7. https://www.rchsd.org/health-article/anesthesia-what-to-expect/
8. https://www.choa.org/medical-services/radiology/sedation-and-anesthesia
9. https://flagstaffdentistry4kids.com/general-anesthesia-instructions
10. https://www.rch.org.au/kidsinfo/fact_sheets/Sedation_for_procedures/

Q26. How soon will my child wake up after anesthesia?

Answer:
Children typically begin waking up from anesthesia within a few minutes after the procedure ends, though full alertness can take 30 minutes to an hour or more.
Factors Affecting Wake-Up Time
Recovery speed varies by anesthesia type, with short-acting drugs like propofol allowing quicker

emergence. Children's age, health, procedure length, and individual metabolism also influence timing; some wake before parents arrive, while others sleep longer. Emergence delirium—crying or agitation—is common but temporary and often unremembered.

Recovery Room Monitoring

In the post-anesthesia care unit (PACU), staff closely watch vital signs as kids stir, often sleepy, nauseous, or irritable for about an hour. Parents usually join soon after, and discharge follows once stable, typically 1-3 hours post-procedure. Full effects like drowsiness or imbalance may linger up to 24 hours.

Home Care Tips

Keep activity low, lights dim, and supervise closely, as fall risk persists for 24 hours. Avoid waking a sleeping child forcibly; let them rouse naturally. Contact a doctor if hard to arouse after 6 hours or breathing issues arise.

1. https://www.apsf.org/patient-guide/how-long-does-it-take-to-wake-up-from-anesthesia/
2. https://kidshealth.org/en/parents/anesthesia.html
3. https://childrens.dartmouth-health.org/your-visit/anesthesia-and-sedation-faqs
4. https://www.cincinnatichildrens.org/-/media/Cincinnati-Childrens/Home/patients/visit/after-treatment/discharge/after-surgery-discharge-folder-PDF.pdf
5. https://nyulangone.org/locations/hassenfeld-childrens-hospital/your-childs-visit/prepare-for-your-childs-surgery/after-your-childs-surgery
6. https://www.chp.edu/our-services/sedation-services/after-sedation
7. https://www.choa.org/-/media/Files/Childrens/teaching-sheets/after-anesthesia-care---child.pdf
8. https://www.uchicagomedicine.org/comer/conditions-services/pediatric-surgery/recovery
9. https://www.cincinnatichildrens.org/-/media/Cincinnati-Childrens/Home/service/c/colorectal/treatments/preparing-surgery/preparing-article-expect-after-pdf.pdf
10. https://www.chop.edu/patients-and-visitors/guide-your-childs-surgery/going-home-after-your-childs-surgery/what-expect-home

Q27. When can I see my child after anesthesia and surgery?

Answer:

You can typically see your child shortly after surgery, once they are moved to the recovery area and initial assessments are complete.

Recovery Timeline

Children go to a Post-Anesthesia Care Unit (PACU) right after surgery, where nurses monitor vital signs and anesthesia recovery. Parents are usually allowed in soon after, often within minutes to an hour, depending on the child's stability and hospital protocols.

Visitor Guidelines

Only one or two adults may visit at a time in the PACU for safety, with siblings often restricted until later. The surgical team will notify you when it's time to join your child.

Factors Affecting Access

Timing varies by surgery type, child's age, and condition—pre-term infants or complex cases may require longer monitoring. Full recovery from anesthesia averages 45 minutes, but discharge home could take hours.

1. https://www.stlouischildrens.org/conditions-treatments/anesthesia-services/frequently-asked-questions
2. https://www.uclahealth.org/hospitals/mattel/preparing-surgery/after-your-childs-surgery
3. https://www.advocatechildrenshospital.com/services/pediatric-general-surgery/preparing-for-your-visit/after-surgery
4. https://www.chla.org/gen-surg-post-op-instructions
5. https://www.adventisthealthcare.com/services/pediatrics/surgery/after/
6. https://www.austinpediatricsurgery.com/post/pediatric-surgery-recovery-what-to-expect-post-op
7. https://kidshealth.org/en/parents/anesthesia.html
8. https://plymouth.truenorthdocs.com/Anesthesia-and-Your-Child-1
9. https://www.chop.edu/patients-and-visitors/guide-your-childs-surgery/going-home-after-your-childs-surgery
10. https://www.choa.org/-/media/Files/Childrens/teaching-sheets/after-anesthesia-care---child.pdf

Q28. Will my child have pain after anesthesia?

Answer:
Children often experience some discomfort or pain after anesthesia wears off, mainly from the surgery itself rather than the anesthesia. This can include soreness at the incision site, throat irritation from breathing tubes, or general achiness, but severity varies by procedure and child's age. Most recover fully within hours to days with proper pain management.

Common Post-Anesthesia Sensations

Soreness or mild pain is typical as numbness fades, especially with local or regional anesthesia. Nausea, restlessness, or thirst may also occur alongside pain, peaking in the first few hours post-recovery. Serious complications from anesthesia are rare in children.

Pain Management Tips

Follow your doctor's instructions for prescribed pain relievers like ibuprofen or acetaminophen to keep discomfort minimal. Comfort measures such as holding your child, changing positions, or offering clear liquids can help ease symptoms. Contact the healthcare provider if pain seems severe or persists beyond expected recovery time.

1. https://kidshealth.org/en/parents/anesthesia.html
2. https://www.nationwidechildrens.org/conditions/health-library/discomforts-and-complications-after-a-child-has-surgery
3. https://www.chop.edu/patients-and-visitors/guide-your-childs-surgery/your-childs-recovery-after-surgery
4. https://pmc.ncbi.nlm.nih.gov/articles/PMC4675339/
5. https://greenvillekidsdental.com/general-anesthesia-instructions
6. https://www.healthychildren.org/English/health-issues/conditions/treatments/Pages/Managing-a-Childs-Pain-After-Surgery.aspx

7. https://mydoctor.kaiserpermanente.org/ncal/article/information-about-pediatric-anesthesia-575533
8. https://www.hss.edu/health-library/conditions-and-treatments/effects-of-anesthesia-on-children
9. https://www.austinpediatricsurgery.com/post/pediatric-surgery-recovery-what-to-expect-post-op
10. https://www.choa.org/-/media/Files/Childrens/teaching-sheets/after-anesthesia-care---child.pdf

Q29. Will my child feel sick or throw up after anesthesia?

Answer:
Children may experience nausea or vomiting after anesthesia, but it's not guaranteed for every child. These are common but typically mild side effects that resolve within hours to a day.
Common Side Effects
Nausea and vomiting occur more frequently in children than adults, with rates up to 33-82% depending on factors like surgery type, anesthesia used, and pain medications. Other minor effects include drowsiness, sore throat, and dizziness.
Risk Factors
Higher risks arise from procedures longer than 30 minutes, eye or ENT surgeries, volatile anesthetics, opioids, child age over 3, or family history of motion sickness. Preventive medications like antiemetics are often given to reduce these.
Management Tips
Offer small amounts of clear liquids after 1-2 hours if no vomiting, and monitor closely; contact a doctor if vomiting persists beyond 24 hours or exceeds 3 times in 4 hours. Rest in a quiet, low-light environment helps recovery.

1. https://nortonchildrens.com/news/avoiding-anesthesia-complications-for-kids/
2. https://childrenswi.org/teaching-sheet/surgery/care-of-your-child-after-general-anesthesia
3. https://pmc.ncbi.nlm.nih.gov/articles/PMC7101192/
4. https://www.chop.edu/patients-and-visitors/guide-your-childs-surgery/your-childs-recovery-after-surgery
5. https://www.nationwidechildrens.org/conditions/health-library/discomforts-and-complications-after-a-child-has-surgery
6. https://www.arch4kids.com/after-surgery/
7. https://www.hss.edu/health-library/conditions-and-treatments/effects-of-anesthesia-on-children
8. https://www.choa.org/-/media/Files/Childrens/teaching-sheets/after-anesthesia-care---child.pdf
9. https://www.cincinnatichildrens.org/-/media/Cincinnati-Childrens/Home/patients/visit/after-treatment/discharge/after-surgery-discharge-folder-PDF.pdf
10. https://www.texaschildrens.org/sites/tc/files/uploads/documents/AnesthesiaAndYourChild-2025.pdf

Q30. If my child had spinal or epidural anesthesia, could they get a headache afterward?

Answer:
Yes, children can develop a headache after spinal or epidural anesthesia, known as post-dural puncture headache (PDPH). This occurs due to cerebrospinal fluid leakage from the puncture site, lowering pressure around the brain and causing symptoms like a severe positional headache that worsens when upright.

Symptoms
PDPH typically starts 24-48 hours after the procedure and may include neck stiffness, nausea, sensitivity to light, or hearing changes. In younger children, diagnosis can be challenging if they can't verbalize symptoms clearly.

Risk and Likelihood
Young patients, including children, face a higher risk than adults, though it's relatively uncommon overall—around 1 in 100 for epidurals. Factors like needle size influence incidence.

Management
Conservative treatments like bed rest and hydration are first-line, often resolving it within days to weeks. Persistent cases may need an epidural blood patch, which is safe and effective in children at doses of 0.2-0.3 mL/kg.

1. https://www.cuh.nhs.uk/patient-information/headache-after-an-epidural-or-spinal-anaesthetic/
2. https://pedsanesthesia.org/wp-content/uploads/2018/11/Post-dural-Puncture-Headache-in-Pediatric-Anesthesia-08_18.pptx
3. https://www.hdft.nhs.uk/wp-content/uploads/2016/02/headache-after-epidural-or-spinal-injection.pdf
4. https://criticalcarepartners.squarespace.com/s/CCP-Headache-after-spinal-or-epidural-injection.pdf
5. https://pubmed.ncbi.nlm.nih.gov/12655411/
6. https://thewomens.r.worldssl.net/images/uploads/fact-sheets/Headache_after_an_epidural_251003.pdf
7. https://pmc.ncbi.nlm.nih.gov/articles/PMC4727636/
8. https://pmc.ncbi.nlm.nih.gov/articles/PMC10960508/
9. https://soap.memberclicks.net/assets/infographic/SOAP Headaches Health Infographic (8 x 10 in).pdf
10. https://my.clevelandclinic.org/health/diseases/17927-spinal-headaches

Q31. How soon can my child eat and drink after waking up from general anesthesia?

Answer:
Children can typically start sipping clear liquids soon after fully waking from general anesthesia, often within 10-30 minutes if their swallowing reflex has returned and they're alert, to ease thirst without risking nausea. Solid foods or heavier meals usually follow once liquids are tolerated well, progressing to a light diet within 1-2 hours and normal eating in 12-24 hours, depending on the procedure.

Key Factors
Medical staff monitor vital signs, cough/swallow reflexes, and alertness before allowing intake; early small sips of water are safe for non-abdominal surgeries and improve comfort. For abdominal surgery, wait until gas or stool passes, as anesthesia slows bowels for up to 48 hours.
Starting Fluids
Begin with clear options like water, apple juice, or popsicles in tiny amounts frequently; avoid milk, citrus, or straws initially to prevent vomiting. Advance slowly if no nausea occurs.
Precautions
Watch for vomiting, dizziness, or sore throat—offer cool fluids for relief but stop and notify staff if issues arise. Always follow your healthcare team's specific instructions, as they tailor based on surgery type and your child's response.

1. https://pmc.ncbi.nlm.nih.gov/articles/PMC7368717/
2. https://monashchildrenshospital.org/wp-content/uploads/2023/03/childrens-anaesthetics-faq_pt-info-final_v1_may-2022.pdf
3. https://www.chop.edu/patients-and-visitors/guide-your-childs-surgery/your-childs-recovery-after-surgery
4. https://www.nationwidechildrens.org/family-resources-education/700childrens/2019/10/what-can-kids-eat-before-and-after-surgery
5. https://www.childrens.com/specialties-services/specialty-centers-and-programs/anesthesiology/faq
6. https://childrenswi.org/teaching-sheet/surgery/care-of-your-child-after-general-anesthesia
7. https://www.salidasurgerycenter.com/post-anesthesia-instructions
8. https://www.cincinnatichildrens.org/-/media/Cincinnati-Childrens/Home/patients/visit/after-treatment/discharge/after-surgery-discharge-folder-PDF.pdf
9. https://www.choa.org/-/media/Files/Childrens/teaching-sheets/after-anesthesia-care---child.pdf
10. https://www.nationwidechildrens.org/family-resources-education/700childrens/2018/07/npo-eating-and-drinking-before-surgery

Q32. How long will my child need to stay at the hospital after anesthesia?

Answer:
The length of a child's hospital stay after anesthesia varies based on factors like the procedure type, child's age, health history, and whether it's general or sedation anesthesia. Most children recover in the post-anesthesia care unit (PACU) for 20 minutes to a few hours before discharge if it's a minor procedure. For major surgeries or young infants, an overnight stay or longer monitoring may be required.
Recovery Room Time
Children typically spend 45 minutes to 1-2 hours in the PACU waking up, with nurses monitoring breathing, vital signs, and alertness. Infants under 60 weeks post-conceptual age often need 6-12 hours of observation due to apnea risk.
Discharge Criteria

Kids go home once fully awake, pain-controlled, tolerating fluids without vomiting, and stable. Preterm or full-term infants under 6 months may require extended stays; major procedures can mean 1+ nights.

Special Cases

Premature infants or those with health issues face longer monitoring, sometimes overnight. Always follow your child's medical team's specific guidelines, as they tailor based on individual needs.

1. https://together.stjude.org/en-us/medical-care/medication-management/travel-after-anesthesia.html
2. https://www.austinpediatricsurgery.com/post/pediatric-surgery-recovery-what-to-expect-post-op
3. https://kidshealth.org/en/parents/anesthesia.html
4. https://www.luriechildrens.org/globalassets/media/pages/appointments/observation-policy-for-infants-after-anesthesia-for-families_final_03-05-18.pdf
5. https://www.chp.edu/our-services/sedation-services/after-sedation
6. https://childrenswi.org/family-resources/parenting-resources/health-resources/your-childs-surgery/general-surgery-guide/after-surgery
7. https://www.chop.edu/patients-and-visitors/guide-your-childs-surgery/going-home-after-your-childs-surgery
8. https://www.advocatechildrenshospital.com/services/pediatric-general-surgery/preparing-for-your-visit/after-surgery
9. https://health.ucdavis.edu/children/patient-education/Anesthesia-after-care
10. https://www.chop.edu/patients-and-visitors/guide-your-childs-surgery/going-home-after-your-childs-surgery/what-expect-home

Q33. Who can I contact if I have more questions after my child’s anesthesia?

Answer:

Contact your child's hospital or surgical facility directly for post-anesthesia questions, as they provide specific contacts like the anesthesia team or on-call anesthesiologist. Many pediatric centers offer dedicated lines, such as a preoperative assessment center during business hours or the operator for after-hours pediatric anesthesiologist support. Your surgeon's office can also connect you to the right anesthesia personnel.

Key Contacts

- Call the hospital's Center for Preoperative Assessment and Planning (e.g., weekdays) or operator for the on-call pediatric anesthesiologist (evenings/weekends).
- Reach out to the anesthesiology department or preoperative clinic at the facility where surgery occurred.
- In urgent cases like persistent vomiting or breathing issues, contact your doctor immediately or go to the ER.

When to Seek Help

Monitor for issues like fever over 101°F, uncontrolled pain, or agitation lasting hours, and call the provided contacts or your health care provider. Discharge instructions typically include who

to call for follow-up.

1. https://www.stlouischildrens.org/conditions-treatments/anesthesia-services/frequently-asked-questions
2. https://pedsanesthesia.org/wp-content/uploads/2021/07/SPA-FAQs-Final-V2-1.pdf
3. https://kidshealth.org/en/parents/anesthesia.html
4. https://www.choa.org/-/media/Files/Childrens/teaching-sheets/after-anesthesia-care---child.pdf
5. https://www.uclahealth.org/hospitals/mattel/preparing-surgery/after-your-childs-surgery
6. https://www.healthychildren.org/English/health-issues/conditions/treatments/Pages/Anesthesia-Safety-Infants-Toddlers-Parent-FAQs.aspx
7. https://medlineplus.gov/ency/patientinstructions/000184.htm
8. https://www.chop.edu/patients-and-visitors/guide-your-childs-surgery/your-childs-recovery-after-surgery
9. https://madeforthismoment.asahq.org/preparing-for-surgery/prep/preparing-for-surgery-kids-checklist/
10. https://health.ucdavis.edu/children/patient-education/Anesthesia-after-care

Model 5

Q1.What are the different types of anesthesia?

Answer:
There are four main types of anesthesia: general, regional, local, and sedation. Each works differently to control pain and awareness during medical procedures.
The Four Types of Anesthesia

- **General anesthesia**:
  - Makes you completely unconscious and unaware of your surroundings.
  - Used for major surgeries like heart, brain, or abdominal operations.
  - Administered via IV or inhaled gases.
  - Side effects may include nausea, sore throat, or drowsiness.
- **Regional anesthesia**:
  - Blocks pain in a larger area of the body, such as everything below the waist.
  - Examples: **epidural** (common in childbirth), **spinal block** (used in C-sections), or nerve blocks for limbs.
  - You may remain awake or receive sedation alongside it.
- **Local anesthesia**:
  - Numbs a small, specific area (like a tooth, skin biopsy site, or minor wound).
  - You stay fully awake and alert.
  - Least likely to cause side effects, though mild soreness or itching at the injection site can occur.
- **Sedation (monitored anesthesia care)**:
  - Also called “twilight sleep.”

- Relaxes you and may make you drowsy or lightly asleep, but you can often respond if needed.
    - Common for procedures like colonoscopies, dental surgery, or minor outpatient operations.
    - Levels range from mild (awake but relaxed) to deep (nearly unconscious).

The choice depends on:

- **Type of procedure** (minor vs. major surgery).
- **Patient health** (age, medical conditions, allergies).
- **Preferences** (some patients prefer sedation over full unconsciousness).

Quick Comparison Table

Type	Consciousness	Area Affected	Common Uses
**General**	Unconscious	Whole body	Major surgeries (heart, brain, abdomen)
**Regional**	Awake or sedated	Large region (waist down, limb)	Childbirth, orthopedic surgery
**Local**	Awake	Small area	Dental work, skin biopsy
**Sedation**	Drowsy/light sleep	Variable	Colonoscopy, minor outpatient surgery

Key Takeaway

Anesthesia isn't one-size-fits-all. **General anesthesia** is strongest, **regional anesthesia** targets larger areas, **local anesthesia** is for small procedures, and **sedation** bridges the gap by calming you without full unconsciousness.

Would you like me to dive deeper into the **risks and side effects of each type** or explore **how anesthesia is administered and monitored**?

Sources:

References (3)

1*Anesthesia: What It Is, Side Effects, Risks & Types*.
https://my.clevelandclinic.org/health/treatments/15286-anesthesia

2*Types of Anesthesia - Anesthesiology | UCLA Health*. https://www.uclahealth.org/medical-services/anesthesiology/types-anesthesia

3*4 Types of Anesthesia and How They're Used - Verywell Health*.
https://www.verywellhealth.com/types-of-anesthesia-8671639

Q2.What is general anesthesia?

Answer:

General anesthesia is a medically induced state of unconsciousness where you are completely unaware and unable to feel pain during surgery or other medical procedures. It is achieved using a combination of intravenous drugs and inhaled gases, carefully monitored by an anesthesiologist or nurse anesthetist.

Key Features of General Anesthesia

- **Unconsciousness**: You are placed in a sleep-like state, unresponsive even to painful

stimuli.

- **Pain relief**: Your brain does not process pain signals, so you feel no pain during the procedure.
- **Amnesia**: You typically have no memory of the surgery.
- **Muscle relaxation**: Muscle reflexes are suppressed, making surgery easier and safer.
- **Controlled breathing**: Often requires a breathing tube, as natural breathing may be insufficient.

How It's Administered

- **IV medications**: Drugs are injected into your veins to induce unconsciousness.
- **Inhaled anesthetics**: Gases delivered through a mask or breathing tube help maintain anesthesia.
- **Monitoring**: Vital signs like heart rate, blood pressure, oxygen levels, and temperature are continuously tracked.

Risks and Side Effects

Most people tolerate general anesthesia well, but possible effects include:

- **Grogginess and confusion** after waking.
- **Nausea or vomiting**.
- **Sore throat** from the breathing tube.
- **Shivering or muscle aches**.
- Rare but serious risks: **allergic reactions**, **anesthesia awareness** (waking during surgery), or complications in people with conditions like sleep apnea, obesity, or heart disease.

Why It's Used

General anesthesia is chosen for:

- **Long or complex surgeries**.
- **Procedures involving major organs** (heart, lungs, abdomen).
- **Operations requiring complete stillness**, such as neurosurgery.

In short, **general anesthesia allows doctors to perform invasive procedures safely and painlessly while keeping patients unconscious and stable**.

Would you like me to break down the **differences between general, local, and regional anesthesia**, or explore **how the body recovers after anesthesia**?

Sources:

References (3)

1*General anesthesia - Mayo Clinic*. https://www.mayoclinic.org/tests-procedures/anesthesia/about/pac-20384568

2*Anesthesia: What It Is, Side Effects, Risks & Types*. https://my.clevelandclinic.org/health/treatments/15286-anesthesia

3*General anaesthesia - Wikipedia*. https://en.wikipedia.org/wiki/General_anaesthesia

Q3.How is anesthesia different for children compared to adults?

Answer:

Children require anesthesia that is carefully tailored to their unique anatomy, physiology, and psychology, making it significantly different from adult anesthesia. Key differences include airway structure, drug dosing, metabolism, emotional needs,

Major Differences Between Pediatric and Adult Anesthesia

Airway Anatomy

- **Smaller, more complex airway**: Children have proportionally larger tongues, omega-shaped epiglottises, and higher-positioned larynxes, making airway management more challenging.
- **Narrowest point**: In children, the cricoid ring is the narrowest part of the airway, while in adults it's the vocal cords.
- **Higher risk of obstruction**: These differences increase the likelihood of airway-related complications, with respiratory events accounting for up to 30% of pediatric anesthesia incidents.

Physiology & Metabolism

- **Higher oxygen consumption**: Children consume 6–8 mL/kg/min of oxygen, nearly double that of adults, leading to faster desaturation during apnea.
- **Cardiac output dependence**: Children rely more on heart rate than stroke volume to maintain cardiac output, so bradycardia can quickly destabilize them.
- **Drug metabolism differences**: Higher metabolic rates and larger distribution volumes mean children often need higher per-kg doses of anesthetics (e.g., propofol 2.5–3.5 mg/kg vs. 1.5–2.5 mg/kg in adults).

Pharmacology & Dosing

- **Age-specific dosages**: Pediatric anesthesia requires careful weight- and age-based dosing to avoid overdosing or underdosing.
- **Variable drug responses**: Neonates, infants, and older children respond differently, requiring individualized titration.

Psychological & Behavioral Needs

- **Preoperative anxiety**: Up to 65% of children experience significant anxiety before surgery, which can lead to postoperative behavioral changes in 60% of cases.
- **Special strategies**: Techniques like parental presence, distraction, play therapy, and age-appropriate sedation are often used.

Temperature Regulation

- **Greater risk of hypothermia**: Children have a larger surface area-to-volume ratio and less fat, making them more prone to perioperative hypothermia (seen in up to 20% of cases).

Postoperative Complications

- **Higher incidence of nausea/vomiting**: Children face up to 42% risk in high-risk groups, compared to ~30% in adults.
- **Pain management differences**: Pediatric anesthesiologists often use multimodal or regional techniques to minimize opioid use.

Summary Table

**Aspect**	**Children**	**Adults**
**Airway anatomy**	Larger tongue, higher larynx, cricoid ring narrowest	Vocal cords narrowest
**Oxygen consumption**	6–8 mL/kg/min (rapid desaturation)	~3–4 mL/kg/min
**Cardiac output**	HR-dependent, bradycardia dangerous	Stroke volume more important
**Drug dosing**	Higher per-kg doses, variable responses	More predictable dosing
**Psychological needs**	High anxiety, parental presence helpful	Less anxiety, self-coping
**Temperature**	High hypothermia risk	Lower risk

**regulation**		
**Post-op nausea/vomiting**	Up to 42%	~30%

In short, **children are not "small adults" when it comes to anesthesia**. Their unique physiology, airway structure, and emotional needs demand specialized approaches to ensure safety and comfort.

Would you like me to dive deeper into drug dosing differences, airway management techniques, or psychological strategies for children?

Sources:

References (3)

1*Overview of Pediatric vs. Adult Anesthesia - Anesthesia Experts*. https://anesthesiaexperts.com/overview-pediatric-vs-adult-anesthesia/

2*Pediatric Anesthesiology Vs. Adult Anesthesiology (Decoded)*. https://thepainpantry.com/pediatric-anesthesiology-vs-adult-anesthesiology-decoded/

3*Overview of Pediatric vs. Adult Anesthesia*. https://radiusanesthesia.com/pediatric-vs-adult-anesthesia/

Q4. How is general anesthesia different from sedation?

Answer:

General anesthesia makes you completely unconscious and unable to respond, while sedation keeps you relaxed with varying levels of awareness.

Levels of Consciousness

- **Sedation**: Exists on a spectrum from *minimal* (awake but calm) to *deep* (nearly unconscious but still rousable). Patients may still breathe on their own and sometimes respond to verbal or tactile stimulation.
- **General anesthesia**: Produces a *complete loss of consciousness*. Patients cannot be awakened even by painful stimuli, and breathing usually requires assistance through intubation or ventilation.

Typical Uses

- **Sedation procedures**: Colonoscopy, dental work, biopsies, minor fracture repairs, or skin surgeries. It's often paired with local anesthesia for pain control.
- **General anesthesia procedures**: Major surgeries involving the heart, brain, abdomen, or large areas of the body. It's chosen when immobility, deep pain control, or airway management is essential.

Key Differences

**Aspect**	**Sedation**	**General anesthesia**
**Consciousness**	Reduced awareness, spectrum from minimal to deep	Complete unconsciousness
**Responsiveness**	May respond to stimuli depending on depth	No response even to pain
**Breathing**	Often independent, though deep sedation may impair	Requires airway support

**Cardiovascular function**	Usually stable	May be impaired, requires close monitoring
**Recovery**	Faster, often within an hour for lighter sedation	Slower, grogginess may last 24–48 hours
**Risk level**	Lower, but can deepen unintentionally	Higher, requires anesthesiologist and full monitoring

Sources:

Risks and Side Effects

Both methods can cause **low blood pressure, respiratory depression, nausea, or sore throat**, but risks are generally greater with general anesthesia because of its depth and airway involvement.

In short, **sedation is like dimming the lights, while general anesthesia is switching them off entirely**. Sedation is chosen for shorter, less invasive procedures, while general anesthesia is reserved for complex surgeries requiring full unconsciousness.

Would you like me to break down the **three levels of sedation** in detail, or compare the **recovery experiences** between the two?

References (5)

1*What's the Difference Between Sedation vs. General Anesthesia?*.
https://www.emedicinehealth.com/sedation_vs_general_anesthesia_difference/article_em.htm
2*Sedation vs. Anesthesia: Differences, Types, Medications*.
https://www.medicinenet.com/sedation_vs_general_anesthesia/article.htm
3*4 Types of Anesthesia and How They're Used - Verywell Health*.
https://www.verywellhealth.com/types-of-anesthesia-8671639
4*What Is the Difference Between Sedation and General Anesthesia ...*.
https://biologyinsights.com/what-is-the-difference-between-sedation-and-general-anesthesia/
5*General Anesthesia vs Sedation | Key Differences, Benefits & Risks*.
https://myvibedentistry.com/difference-between-anesthesia-and-sedation/

Q5.Is general anesthesia safe for my child?

Answer:

General anesthesia is considered very safe for children when administered by trained specialists, though caution is advised for repeated or lengthy use in children under 3 years old.

Key Safety Insights

- **Modern anesthesia safety**: Advances in technology, medications, and monitoring have made anesthesia safer than ever for kids.
- **FDA guidance**: The U.S. Food and Drug Administration warns that *repeated or lengthy use* (over 3 hours) of general anesthetics in children younger than 3 may affect brain development.
- **Single short exposure**: Research suggests that one short procedure is unlikely to cause long-term behavioral or learning problems.
- **Specialist care**: Pediatric anesthesiologists tailor doses to a child’s age and weight, and continuously monitor vital signs to ensure safety.
- **Risks vs. benefits**: For serious or life-threatening conditions (e.g., congenital heart issues,

bowel obstruction, cleft palate), delaying surgery is riskier than the small risks of anesthesia.
- **Common side effects**: Temporary grogginess, mild nausea, or sore throat are the most frequent after-effects.

What Parents Should Know

- **Discuss timing**: If surgery is elective and your child is under 3, ask whether it can safely be delayed.
- **Ask about duration**: Procedures lasting longer than 3 hours carry more risk for very young children.
- **Request a pediatric anesthesiologist**: Especially important for infants and toddlers, or complex surgeries.
- **Prepare your child**: Honest explanations, comfort items (like a favorite toy), and relaxation techniques can ease anxiety.
- **Monitor recovery**: Watch for persistent vomiting, breathing difficulty, or unusual drowsiness after anesthesia, and contact your doctor if these occur.

Bottom Line

General anesthesia is **safe and necessary** for children when surgery or procedures are required. The main concern is **repeated or prolonged exposure in children under 3**, but for most procedures, the benefits outweigh the risks. Always talk with your child's healthcare team to understand the specific plan, risks, and safety measures.

Would you like me to break down the **steps anesthesiologists take to keep children safe during surgery**, or explore the **long-term research on anesthesia and child brain development**?

Sources:

References (3)

1*Is Anesthesia Safe for Kids? - Cleveland Clinic Health Essentials*. https://health.clevelandclinic.org/is-anesthesia-safe-for-kids

2*Anesthesia for Children: Safety, Myths, and Facts*. https://continentalhospitals.com/blog/anesthesia-for-children-safety-myths-and-facts/

3*FDA Drug Safety Communication*. https://www.fda.gov/drugs/drug-safety-and-availability/fda-drug-safety-communication-fda-review-results-new-warnings-about-using-general-anesthetics-and

Q6.Can anesthesia harm my child's brain development?

Answer:

Short answer: A single, brief exposure to anesthesia is generally considered safe for children, but repeated or prolonged use—especially in those under 3 years old—may carry risks for brain development.

What the research shows

- **Single brief exposure**: Most studies, including large trials, show that one short procedure with anesthesia does *not* cause long-term learning or behavior problems in children.
- **Repeated or prolonged exposure**: The U.S. FDA warns that children under 3 who receive anesthesia for more than 3 hours, or multiple times, may be at higher risk of subtle developmental issues such as learning or memory problems.

- **Animal studies**: Experiments in young animals suggest anesthesia can alter brain connections during critical developmental windows, raising concerns about similar effects in humans.
- **Recent human studies**: Newer research confirms that repeated anesthesia in newborns may shift brain development timelines, potentially affecting how the brain wires itself for sensory and cognitive functions.

Balancing risks and benefits

- **Untreated pain**: Pain itself can harm a child's nervous system, so anesthesia is often the safer choice.
- **Life-saving surgeries**: Procedures for heart, lung, or digestive problems cannot be delayed, even if anesthesia carries some risk.
- **Elective procedures**: For non-urgent surgeries (like ear tubes or eye corrections), parents may discuss with doctors whether waiting until the child is older could reduce risk.

What parents can do

- **Ask about anesthesia type**: Sometimes spinal or local anesthesia can be used instead of general, which may avoid brain risks.
- **Discuss duration and frequency**: Longer or repeated surgeries increase risk, so understanding the surgical plan is important.
- **Talk to specialists**: Pediatricians, anesthesiologists, and surgeons can help weigh the risks and benefits for your child's specific situation.

Key takeaway

Anesthesia is *necessary and generally safe*, but **children under 3 years old are more vulnerable if exposed repeatedly or for long periods**. Parents should always discuss options, timing, and alternatives with their child's medical team before surgery.

Would you like me to break down the **FDA's official guidance for parents** or explore **recent clinical trial findings** in more detail?

Sources: UCLA Health Nemours KidsHealth Harvard University

References (3)

1*Anesthesia and Child Development - UCLA Health*. https://www.uclahealth.org/medical-services/anesthesiology/anesthesia-and-child-development

2*Can Anesthesia Hurt Brain Development? | Nemours KidsHealth*. https://kidshealth.org/en/parents/anesthesia-brain.html

3*Early Exposure to Anesthesia May Shift Brain Development*. https://www.mcb.harvard.edu/department/news/early-exposure-to-anesthesia-may-shift-brain-development/

Q7.Is general anesthesia necessary for my child's dental treatment?

Answer:

General anesthesia is not always necessary for children's dental treatment—it depends on the child's age, medical history, anxiety level, and the complexity of the procedure. In many cases, mild or moderate sedation or local anesthesia is sufficient, but general anesthesia may be recommended for very young, highly anxious, or medically complex children, or when extensive dental work is required.

When General Anesthesia May Be Needed

- **Very young children**: Kids under 6 often struggle to stay still or cooperate, making general anesthesia safer for complex procedures.
- **Severe anxiety or phobia**: Children with extreme fear of dental care may benefit from being fully asleep.
- **Extensive dental work**: Multiple extractions, crowns, or oral surgery are often completed in one session under anesthesia.
- **Special needs or medical conditions**: Children with developmental delays or complex health issues may require anesthesia for safe treatment.

Safety Considerations

- **Protocols**: The American Academy of Pediatrics and Pediatric Dentistry require at least two trained professionals present—one performing the dental work and another monitoring anesthesia.
- **Monitoring**: Vital signs such as heart rate, oxygen levels, and breathing are continuously tracked during anesthesia.
- **Recovery**: Most children go home the same day, though drowsiness, nausea, or irritability may last 24–48 hours.

Alternatives to General Anesthesia

- **Nitrous oxide ("laughing gas")**: Mild sedation that relaxes children without putting them fully to sleep.
- **Moderate sedation**: IV or oral medications make children sleepy but responsive, suitable for older or calmer kids.
- **Local anesthesia**: Numbs only the treatment area, often enough for simple fillings or minor procedures.

What Parents Should Do

- **Discuss options**: Ask your dentist whether sedation or local anesthesia could be sufficient.
- **Review medical history**: Share any conditions, allergies, or medications with the dental team.
- **Ask safety questions**: Who will monitor your child? What protocols are in place? What side effects should you expect?

Bottom line: General anesthesia is reserved for situations where other methods aren't safe or effective. If your child needs only minor dental work and can cooperate, lighter sedation or local anesthesia may be enough. For complex cases, general anesthesia can ensure a safe, pain-free experience when administered by trained professionals.

Would you like me to break down the **different anesthesia options by age group** or focus on **questions you should ask your child's dentist before agreeing to anesthesia**?

References (3)

1*General Anesthesia Protocol for Pediatric Dentistry: A Clinical Guide*. https://medicalhubnews.com/surgery/anesthesia/procedures/general-anesthesia-pediatric-dentistry-2/

2*Anesthesia or Sedation for Your Child's Dental Work?*. https://www.healthychildren.org/English/healthy-living/oral-health/Pages/Anesthesia-or-Sedation-for-Your-Childs-Dental-Work.aspx

3*Safe Pediatric Dental Anesthesia: What Parents Must Know*. https://worldofdentistry.org/pediatric-dental-anesthesia-parents-must-know/

Q8.Who will give my child anesthesia?

Answer:

When a child needs anesthesia, it is typically administered and carefully monitored by a pediatric anesthesiologist or an anesthesiologist trained to work with children. Here's how it usually works:

Pediatric anesthesiologist: A doctor who specializes in giving anesthesia to infants, children, and teenagers. They have extra training in pediatric medicine and anesthesia safety.

Anesthesiologist: A physician who provides anesthesia care for patients of all ages. In many hospitals, anesthesiologists with pediatric experience handle children's cases.

Nurse anesthetist (CRNA): In some hospitals, a certified registered nurse anesthetist may give anesthesia under the supervision of a physician anesthesiologist.

Anesthesia team: Often, it's a coordinated effort. The anesthesiologist or CRNA administers the anesthesia, while nurses and other staff monitor your child's vital signs throughout the procedure.

The exact person depends on the hospital, the type of procedure, and your child's needs. Pediatric hospitals almost always have anesthesiologists who specialize in children.

Would you like me to walk you through what a pediatric anesthesiologist does before, during, and after surgery so you know what to expect?

Q9.What if my child won't cooperate for anesthesia?

Answer:

That's a really important concern, and you're not alone—many children feel anxious or resist when it's time for anesthesia. While I can't give medical advice specific to your child, I can share some **general information** about what usually happens in these situations:

Common Approaches When a Child Resists Anesthesia

- **Parental presence:** Sometimes parents are allowed to stay with their child until anesthesia takes effect, which can reduce fear.
- **Distraction techniques:** Toys, videos, music, or storytelling can help shift focus away from the medical setting.
- **Pre-medication:** Doctors may give a mild sedative (often orally or nasally) before anesthesia to help the child relax.
- **Child life specialists:** Hospitals often have trained staff who use play therapy and age-appropriate explanations to prepare children.
- **Gentle restraint:** In rare cases, if safety is at risk, staff may need to hold the child still—but this is usually a last resort and handled with care.

What You Can Do as a Parent

- **Stay calm and reassuring:** Children often mirror their parents' emotions. Your calm presence can help.
- **Use simple, honest language:** Explain what's happening in terms they can understand, without overwhelming details.
- **Bring comfort items:** A favorite blanket, stuffed animal, or even a parent's shirt can provide security.
- **Ask the team about options:** Every hospital has protocols, but many are flexible in finding the least stressful approach.

Important Note

If you're worried about how your child will react, it's best to **discuss this openly with the anesthesiologist before the procedure**. They can tailor the plan to your child's age, temperament, and medical needs.
Would you like me to share some **specific strategies hospitals use for different age groups** (toddlers vs. older kids)? That way you'll know what to expect depending on your child's stage.

Q10.What if my child is allergic to anesthesia?

Answer:
That's an important and serious concern. I'm not a medical professional, but I can share some general information that may help you understand the situation better:

Key Points About Anesthesia Allergies
True allergies are rare: Most reactions to anesthesia are not caused by allergies but by side effects, sensitivities, or other medical conditions.

Types of reactions:
Mild: Rash, itching, or nausea.
Severe (anaphylaxis): Difficulty breathing, drop in blood pressure, swelling — this is a medical emergency.
Different anesthetics exist: There are many types (general, regional, local), and doctors can often choose alternatives if one causes a reaction.
Pre-surgery precautions:
Inform the anesthesiologist about any past reactions.
Provide a detailed medical history, including allergies to medications, foods, or latex.
Allergy testing may be recommended to identify safe options.
What You Should Do
Consult a specialist: An allergist can help confirm whether your child has a true allergy and identify safe alternatives.
Work with the anesthesiologist: They can tailor the anesthesia plan, sometimes using different drugs or techniques.
Emergency preparedness: Hospitals are equipped to handle allergic reactions during surgery, but advance planning reduces risks.

Important
If your child has ever had a severe reaction to anesthesia, it's critical to tell every healthcare provider before any procedure. Only a qualified doctor can determine the safest approach.
Would you like me to look up the latest medical guidelines on managing anesthesia allergies in children, so you have the most up-to-date information to discuss with your doctor?

Q11.Will my child need any evaluation before anesthesia?

Answer:
Yes — children almost always need a pre-anesthesia evaluation before surgery. This assessment helps ensure your child's safety by reviewing their medical history, identifying risks, and preparing them for anesthesia and surgery.

What the Evaluation Involves

- **Medical history review**: Doctors will ask about past illnesses, surgeries, anesthesia experiences, allergies, medications, and family history of anesthesia complications.
- **Physical exam**: Includes checking vital signs, airway, heart, lungs, and overall development.
- **Risk classification**: Your child will be assigned an **ASA (American Society of Anesthesiologists) Physical Status** score to summarize anesthesia risk.
- **Lab tests**: Depending on age, health, and type of surgery, bloodwork or other tests may be ordered.
- **Special considerations**: Infants younger than 50–60 weeks corrected age, or those with conditions like prematurity, heart disease, or respiratory problems, may not be eligible for outpatient anesthesia.

Why It's Important

- **Safety first**: Identifies hidden risks (e.g., asthma, congenital heart disease, epilepsy) that could complicate anesthesia.
- **Timing decisions**: Helps determine if surgery should proceed, be delayed, or require extra precautions.
- **Preparation**: Families receive guidance on what to expect on surgery day, including fasting instructions and emotional support for the child.

What Parents Can Do

- Bring a **complete list of medications** (including over-the-counter and supplements).
- Share any history of **breathing problems, sleep apnea, or unusual reactions to anesthesia**.
- Ask questions about the process so you and your child feel prepared.
- Follow instructions on **fasting and pre-surgery routines** carefully to avoid delays.

Bottom line: Your child will need a pre-anesthesia evaluation, and it's a routine but crucial step to keep them safe during surgery. It's not just about checking boxes — it's about tailoring anesthesia care to your child's unique health needs.

Would you like me to walk you through the **specific questions doctors usually ask parents during this evaluation** so you can be ready?

Sources:

References (4)

1*Preoperative Evaluation in Children - AAFP*. https://www.aafp.org/pubs/afp/issues/2022/0600/p640.html

2*Preoperative Assessment - American Academy of Pediatrics*. https://publications.aap.org/pediatriccare/article/doi/10.1542/aap.ppcqr.396033/102/Preoperative-Assessment

3*Optimizing preoperative assessment and preparation for safe pediatric ...*. https://www.anesthesiologyjournals.com/archives/2025.v7.i1.A.26/optimizing-preoperative-assessment-and-preparation-for-safe-pediatric-anaesthesia

4*Why are pre-anesthesia assessments helpful?*. https://www.aboutkidshealth.ca/why-are-pre-anesthesia-assessments-helpful

Q12.Can my child have anesthesia with a cold?

Answer:
Short answer: Children with a mild cold *sometimes* can still undergo anesthesia, but it depends on the severity of symptoms. If your child has fever, wheezing, or chest congestion, surgery is often postponed. Always consult the anesthesiologist before proceeding.

Why a Cold Matters for Anesthesia

- **Airway sensitivity:** A cold inflames the respiratory tract, making the airways more reactive. This increases the risk of coughing, laryngospasm (spasm of the vocal cords), or breathing difficulties during anesthesia.
- **Excess secretions:** Runny nose and mucus can obstruct breathing tubes or complicate airway management.
- **Increased complications:** Children with colds are more likely to experience oxygen desaturation, bronchospasm, or prolonged recovery.

When Surgery May Still Proceed

- **Mild symptoms only:** If your child only has a runny nose without fever, chest congestion, or significant cough, anesthesia may still be safe.
- **No lower airway involvement:** Upper respiratory symptoms (like nasal congestion) are less risky than lower airway issues (like wheezing or deep cough).
- **Elective vs. urgent surgery:** For urgent procedures, anesthesiologists may proceed despite a cold, but for elective surgeries, postponement is common.

When Surgery Is Usually Postponed

- **Fever present:** Indicates active infection; anesthesia is generally delayed.
- **Wheezing or chest congestion:** Suggests lower respiratory tract involvement, which raises complication risks.
- **Recent severe illness:** If your child has had flu, bronchitis, or pneumonia recently, doctors often wait several weeks before anesthesia.

What Parents Should Do

- **Inform the anesthesiologist:** Always disclose your child's symptoms before surgery.
- **Expect flexibility:** Surgeons and anesthesiologists may reschedule to ensure safety.
- **Monitor symptoms:** Track fever, cough severity, and breathing changes leading up to the procedure.

Bottom Line
Children with mild colds may still safely undergo anesthesia, but those with fever, wheezing, or chest congestion usually need to postpone surgery. The anesthesiologist will make the final call based on your child's health and the urgency of the procedure.
Would you like me to give you a **checklist of questions to ask your child's anesthesiologist** so you feel prepared before the surgery discussion?
Sources:
References (5)
1*Anesthesia While a Child has a Common Cold - SPA*. https://www2.pedsanesthesia.org/patiented/uri.iphtml
2*Can I Have Surgery if I Have a Cold? - HSS*. https://www.hss.edu/health-library/conditions-and-treatments/why-do-anesthesiologists-care-about-a-common-cold
3*The Common Cold Risks with Anesthesia Explained*. https://www.acibademhealthpoint.com/the-common-cold-risks-with-anesthesia-explained/
4*What Happens If You Go Under Anesthesia With a Cold?*. https://biologyinsights.com/what-happens-if-you-go-under-anesthesia-with-a-cold/

5*What happens if you have a cold and go under anesthesia?*. https://www.calendar-canada.ca/frequently-asked-questions/what-happens-if-you-have-a-cold-and-go-under-anesthesia

Q13.When does my child need to stop eating or drinking before anesthesia?

Answer:
Quick Answer: Most guidelines recommend that children stop solid food **6–8 hours** before anesthesia, formula or breast milk **4–6 hours**, and clear fluids (like water or apple juice) up to **2 hours** before.

Standard Pediatric Fasting Guidelines

Here's a breakdown of the most widely accepted recommendations:

- **Solid food & fatty meals:**
  Stop **6–8 hours** before anesthesia. This includes meat, fried foods, or heavy meals.
- **Infant formula:**
  Stop **6 hours** before anesthesia.
- **Breast milk:**
  Stop **4 hours** before anesthesia.
- **Clear fluids (water, apple juice, clear tea):**
  Allowed up to **2 hours** before anesthesia.

These rules are designed to reduce the risk of aspiration (stomach contents entering the lungs) during anesthesia.

Why Guidelines Differ

- **American Society of Anesthesiologists (ASA):** Recommends the 6-4-2 rule (6 hours solids, 4 hours breast milk, 2 hours clear fluids).
- **European Society of Anaesthesiology & Intensive Care (ESAIC):** Supports more liberal clear fluid intake, sometimes allowing fluids up to 1 hour before anesthesia, with no increase in aspiration risk.
- **Canadian Pediatric Anesthesia Society (2023):** Endorses similar fasting times but emphasizes flexibility and minimizing unnecessary prolonged fasting.

Important Notes

- **Always follow your hospital or anesthesiologist's specific instructions.** Local policies may be stricter or more flexible.
- **Clear fluids are encouraged up to 2 hours before anesthesia** because they help keep children hydrated and reduce irritability.
- **Chewing gum, candy, or milk-based drinks** count as solids and should be avoided after the cutoff.

Practical Example

If your child's surgery is scheduled at **8:00 AM**:

- Last solid food: **midnight (12:00 AM)**
- Last formula: **2:00 AM**
- Last breast milk: **4:00 AM**
- Last clear fluids: **6:00 AM**

Bottom line: The safest approach is the **6-4-2 rule**, but check with your child's anesthesiologist since some centers allow clear fluids closer to surgery.

Would you like me to create a **simple chart or checklist** you can print out for surgery day so it's easy to follow?

Sources:

References (4)

1*Pre-operative fasting in children - ESAIC*. https://esaic.org/wp-content/uploads/2023/12/pre_operative_fasting_in_children__a_guidelin.pdf

2*Practice Guidelines for Preoperative Fasting and the Use of …*. https://www.asahq.org/~/media/sites/asahq/files/public/resources/standards-guidelines/practice-guidelines-for-preoperative-fasting.pdf

3*2023 Canadian Pediatric Anesthesia Society Statement on fasting for …*. https://link.springer.com/article/10.1007/s12630-023-02509-z

4*Guidelines Archive - European Society for Paediatric Anaesthesiology*. https://www.euroespa.com/science-education/specialized-sections/espa-guidelines/guidelines-archive/

Q14.If my child is breastfeeding, do we need to stop before their anesthesia?

Answer:

Yes — your child will need to stop breastfeeding for a period of time before anesthesia. This is because anesthesia temporarily suppresses the body's protective reflexes, and having milk in the stomach increases the risk of aspiration (milk entering the lungs). The exact fasting time depends on your hospital's guidelines and your anesthesiologist's instructions.

Why Fasting Is Needed

- **Aspiration risk:** During anesthesia, swallowing and coughing reflexes are reduced. If the stomach isn't empty, milk or food could be regurgitated and inhaled into the lungs.
- **NPO orders:** Hospitals use "nil per os" (NPO) rules, meaning "nothing by mouth," to keep the stomach clear before anesthesia.

Typical Fasting Guidelines

Many hospitals follow the **"2-4-6-8 rule"** for children:

- **2 hours**: Clear fluids (like water or electrolyte drinks)
- **4 hours**: Breast milk
- **6 hours**: Infant formula
- **8 hours**: Solid food

This means your child usually can breastfeed up until **4 hours before anesthesia**, but not closer. Formula-fed babies need longer fasting because formula digests more slowly.

After Surgery

- **Resuming breastfeeding:** Once your child is awake, alert, and cleared by the medical team, breastfeeding can usually resume right away.
- **Comfort and recovery:** Breastfeeding after surgery provides comfort, hydration, and pain relief, and is encouraged as soon as it's safe.

What You Should Do

- **Tell your anesthesiologist** that your child is breastfed — they will give you precise fasting instructions.
- **Do not fast longer than recommended.** Extended fasting can be stressful and unnecessary for infants.
- **Plan ahead:** If surgery is scheduled early in the morning, you may be asked to wake your child for a last feed before the cutoff time.

Bottom line: Your child will need to stop breastfeeding about **4 hours before anesthesia**, but

can usually resume once they are awake and stable. Always follow the exact instructions from your anesthesiologist, since fasting times can vary slightly by hospital.
Sources:
Would you like me to share a **sample pre-surgery feeding schedule** (e.g., how to time the last feed before an early morning procedure)? That might make the instructions easier to follow.

References (5)

1*My baby is having surgery - La Leche League International*. https://llli.org/breastfeeding-info/my-baby-is-having-surgery/
2*Breastfeeding Before Baby's Surgery - Ask Dr Sears*. https://www.askdrsears.com/topics/feeding-eating/breastfeeding/special-situations/breastfeeding-babys-surgery/
3*Breastfeeding, Surgery, and Anesthesia | InfantRisk Center*. https://www.infantrisk.com/content/breastfeeding-surgery-and-anesthesia
4*Surgery and the Breastfeeding Infant | Children's Hospital of Philadelphia*. https://www.chop.edu/pages/surgery-and-breastfeeding-infant
5*Breastfeeding and anaesthesia*. https://www.breastfeeding.asn.au/resources/breastfeeding-and-anaesthesia

Q15.What should I do if my child takes daily medicines before anesthesia?

Answer:
That's a really important question—thank you for asking it. I can share **general information** about how daily medicines are usually managed before anesthesia, but remember: every child's situation is unique, and the anesthesiologist or surgeon must give the final instructions.

General Guidance

- **Bring a full medication list**: Include prescription drugs, over-the-counter medicines, vitamins, and supplements. Write down doses and timing.
- **Do not stop medicines on your own**: Some daily medicines (like those for seizures, heart conditions, or asthma) are often continued even on the day of surgery. Others may need to be paused because they interact with anesthesia or increase bleeding risk.
- **Ask about morning doses**: The anesthesiologist will tell you whether your child should take their usual morning medicine with a sip of water, delay it, or skip it.
- **Special caution with blood thinners or diabetes medicines**: These often require specific adjustments before anesthesia.
- **Avoid surprises**: Always disclose everything your child takes, even herbal remedies or supplements, since some can affect anesthesia safety.

Why This Matters

- Medicines can change how anesthesia works in the body.
- Stopping some medicines suddenly can be dangerous.
- Continuing others without adjustment can increase risks during surgery.

The safest step is to **call your child's surgeon or anesthesiologist before the procedure** and ask for clear instructions about each medication. They'll tailor the plan to your child's health needs.
Would you like me to outline **common categories of medicines** (like asthma inhalers, seizure meds, antibiotics, etc.) and how they're usually handled before anesthesia? That way you'll have a framework to discuss with your doctor.

Q16.If my child has asthma, diabetes, or other health issues, do I need to do anything special before anesthesia?

Answer:
Yes — children with chronic conditions like asthma or diabetes need special preparation before anesthesia. The key is to ensure their condition is well controlled, medications are reviewed, and the anesthesiologist is fully informed so they can tailor care to minimize risks.

Asthma

- **Control symptoms first:** Children with well-controlled asthma usually tolerate anesthesia safely. If asthma is poorly controlled or there's a recent respiratory infection, anesthesia may be delayed.
- **Medication review:** Inhalers (like bronchodilators or steroids) should be continued up to the day of surgery. Sometimes, a preventive dose of inhaled bronchodilator is given before anesthesia.
- **Risk factors:** Severe asthma increases the risk of airway complications such as bronchospasm during anesthesia.

Diabetes

- **Blood sugar management:** Fasting before surgery can complicate glucose control. The anesthesiologist will adjust insulin or other medications to prevent hypoglycemia or hyperglycemia.
- **Monitoring:** Blood glucose is checked before, during, and after anesthesia. IV fluids may be tailored to maintain stable sugar levels.
- **Communication:** Parents should provide a detailed history of the child's diabetes management plan, including insulin doses and recent glucose readings.

Other Health Issues

- **Heart conditions, seizures, or allergies:** Each requires specific precautions. For example, seizure medications should not be skipped, and allergies to medications must be clearly communicated.
- **Recent illness:** Even a mild cold or fever can increase anesthesia risks, especially for children with underlying conditions.
- **Specialist involvement:** Pediatric anesthesiologists often consult with the child's regular doctors to coordinate care.

What Parents Should Do

- **Inform the team:** Share a complete medical history, including medications, allergies, and recent symptoms.
- **Bring medications:** Have inhalers, insulin, or other essential medicines with you on the day of surgery.
- **Ask questions:** Discuss anesthesia risks and the plan for managing your child's condition with the anesthesiologist.
- **Follow instructions:** Carefully adhere to fasting and medication guidelines provided before surgery.

Bottom line: Children with asthma, diabetes, or other chronic health issues can safely undergo anesthesia, but only if their condition is well managed and the anesthesia team is fully prepared.
Would you like me to create a **checklist for parents** that covers what to bring and what to

confirm with the anesthesiologist before surgery? That way you'll have a practical guide ready.

References (4)

1*anaesthesia in patients with asthma, bronchiolitis and other ...*. https://resources.wfsahq.org/wp-content/uploads/uia30-Anaesthesia-in-patients-with-asthma-bronchiolitis-and-other-respiratory-diseases.pdf
2*Safe Anesthesia for Pediatric Asthma Patients*. https://www.numberanalytics.com/blog/safe-anesthesia-pediatric-asthma
3*Asthma and Anesthesia: What Are the Risks? - Healthline*. https://www.healthline.com/health/asthma/asthma-and-anesthesia
4*Children and Anesthesia - American Society of Anesthesiologists (ASA)*. https://www.asahq.org/~/media/sites/asahq/files/public/resources/patient-brochures/asa_children-anesthesia_final.pdf?la=en

Q17.When should my child and I arrive at the hospital on surgery day for anesthesia?

Answer:

You and your child should arrive at the hospital well before the scheduled surgery time—usually about 1–2 hours in advance—so the anesthesia and surgical team can complete pre-op checks, paperwork, and preparation. The exact arrival time will be given by your surgeon's office or hospital staff.

Typical Arrival Guidelines

- **Hospital staff will call you 1–2 days before surgery** to confirm the exact arrival time.
- **Arrival is often early in the morning**, especially if your child's surgery is scheduled for the first case of the day.
- **Plan for 1–2 hours before the procedure** to allow time for:
  - Registration and check-in
  - Pre-anesthesia evaluation (vital signs, medical history review)
  - Meeting with the anesthesiologist to discuss the plan and answer questions
  - Changing into hospital gowns and final preparation

Why Early Arrival Matters

- **Safety checks:** The anesthesia team must ensure your child is healthy enough for anesthesia.
- **NPO (nothing by mouth) confirmation:** Staff will verify that your child followed fasting instructions (no food or drink for a set period before surgery).
- **Calming nerves:** Extra time helps reduce stress for both you and your child.
- **Flexibility:** Hospitals often schedule multiple surgeries, so arriving early prevents delays if the schedule shifts slightly.

What to Expect

Step	What Happens	Timing
Check-in	Registration, ID, insurance	Immediately upon arrival
Pre-op prep	Vitals, gown, IV placement	30–60 minutes before surgery
Anesthesia consult	Meet anesthesiologist, review plan	Before surgery begins
Surgery start	Child taken to OR	At scheduled time

Since every hospital has slightly different protocols, **always follow the exact arrival instructions given by your child's surgeon or hospital staff.**

Would you like me to also outline **what you and your child should do the night before**

surgery (fasting, bathing, medications) so you're fully prepared?

References (5)

1*Pre-Surgery Instruction Guide - Texas Children's*. https://www.texaschildrens.org/sites/tc/files/uploads/documents/2025-SurgeryGuide For PDF ENG_fillable.pdf

2*The day of surgery for your child: MedlinePlus Medical Encyclopedia*. https://medlineplus.gov/ency/patientinstructions/000579.htm

3*Preparing for Your Child's Surgery - UCSF Benioff Children's Hospital*. https://www.ucsfbenioffchildrens.org/your-stay/your-childs-surgery-what-to-expect

4*Day Surgery Program | Anesthesia Guidelines - Boston Children's Hospital*. https://www.childrenshospital.org/services/day-surgery-program/anesthesia-guidelines

5*Before You Arrive for Surgery - St. Louis Children's Hospital*. https://www.stlouischildrens.org/visit-us/preparing-for-your-visit/planning-for-surgery/before-you-arrive-for-surgery

Q18.Can my child visit the surgery and recovery areas before anesthesia?

Answer:

Direct Answer: In most hospitals, children are *not allowed to visit the operating room or recovery area before anesthesia*. Instead, parents and staff help prepare them in a pre-op area. After surgery, parents are usually permitted to join their child in the recovery room once the child is stable.

What to Expect Before Anesthesia

- **Pre-op area:** Children typically wait here before surgery. Nurses and anesthesiologists explain what will happen, check vital signs, and sometimes allow parents to stay until anesthesia begins.
- **Comfort measures:** Hospitals often provide toys, videos, or child-life specialists to reduce anxiety. Parents may be present until the child is asleep, depending on hospital policy.
- **Operating room access:** For safety and sterility, children (and parents) are not permitted to "visit" the OR beforehand. The anesthesia team usually escorts the child directly into surgery from pre-op.

Recovery Area (PACU)

- **Post-anesthesia care unit (PACU):** After surgery, children are taken to recovery where nurses and anesthesiologists closely monitor them.
- **Parent access:** Once the child is stable, parents are usually allowed to join them in recovery. Staff will check vital signs, consciousness, wound sites, and IVs while keeping the child comfortable.
- **Duration:** Time in recovery depends on the type of surgery and how the child responds to anesthesia.

Key Takeaways

- **No pre-anesthesia visits** to surgery or recovery areas for children.
- **Parents may accompany** their child in pre-op and later in recovery, depending on hospital rules.
- **Child-life specialists** or staff can help prepare your child with explanations, play, or

tours of non-sterile areas.

If you'd like, I can outline **child-friendly strategies to reduce anxiety before anesthesia**—things like role-play, picture books, or hospital tours of safe areas. Would you like me to share those?

Sources:

References (5)

1*Recovery Room/Post-Anesthesia Care Unit - Stanford Medicine Children's ...*. https://www.stanfordchildrens.org/en/topic/default?id=recovery-roompost-anesthesia-care-unit-90-P03025

2*What to Expect Before, During, & After Anesthesia - KidsHealth*. https://kidshealth.org/Partnership/en/parents/anesthesia.html

3*Children and Anesthesia - American Society of Anesthesiologists (ASA)*. https://www.asahq.org/~/media/sites/asahq/files/public/resources/patient-brochures/asa_children-anesthesia_final.pdf?la=en

4*Anesthesia and Your Child: What to Expect | Gateway Pediatric Surgery ...*. https://www.gatewaypediatricsurgery.com/patients/anesthesia

5*Your Child's Recovery After Surgery*. https://www.chop.edu/patients-and-visitors/guide-your-childs-surgery/your-childs-recovery-after-surgery

Q19.Will my child be awake or know what's happening during anesthesia?

Answer:

That's an important question, and I can share some general information to help you understand what typically happens.

General anesthesia:

- Your child will be completely unconscious. They will not be awake, aware, or able to feel pain during the procedure.
- They won't remember what happened once the anesthesia takes effect.

Sedation (lighter anesthesia):

- Depending on the type, your child may be drowsy but not fully unconscious.
- In this case, they may be somewhat aware of their surroundings but usually won't feel pain and often won't remember much afterward.

What matters most:

- The anesthesiologist tailors the type and depth of anesthesia to the procedure and your child's needs.
- Safety monitoring is continuous, and the team ensures your child is comfortable and unaware during surgery.

If you're feeling anxious, it's completely normal. The best step is to ask your child's anesthesiologist directly—they can explain exactly what type of anesthesia will be used and what your child will experience.

Would you like me to break down the differences between general anesthesia and sedation in children in a simple table so you can see the contrast clearly?

Q20.Will my child feel any pain while under anesthesia?

Answer:
That's an important and very caring question. In general, when a child is under anesthesia, they do **not feel pain** during the procedure. Anesthesia works by blocking pain signals and awareness, so the child is unconscious or deeply sedated and unable to sense what's happening.
Here are some key points to help clarify:

- **General anesthesia**: Your child is fully asleep and unaware. They won't feel pain or remember the procedure.
- **Regional anesthesia (like spinal or epidural)**: Numbs a specific part of the body. The child may be awake but won't feel pain in the numbed area.
- **Sedation with local anesthesia**: Used for smaller procedures. The area is numbed, and sedation helps them stay calm and relaxed.

After the anesthesia wears off, some discomfort or pain may occur depending on the procedure, but doctors usually manage this with pain relief medications.
It's natural to feel concerned, and anesthesiologists are specially trained to keep children safe and comfortable throughout. If you're preparing for a procedure, you can ask the anesthesiologist to explain exactly what type of anesthesia will be used and how they'll manage pain afterward.
Would you like me to break down the **different types of anesthesia commonly used in children** and what each one feels like from the child's perspective? That way you'll have a clearer picture of what to expect.

Q21.Will someone watch my child the whole time during anesthesia to keep them safe?

Answer:
Yes — during anesthesia, your child will be continuously monitored by trained medical professionals whose sole responsibility is to keep them safe.
Here's what typically happens:

- Anesthesiologist or nurse anesthetist: A specialist stays with your child throughout the procedure, from the moment anesthesia is started until they wake up.
- Vital signs monitoring: Heart rate, blood pressure, oxygen levels, and breathing are tracked in real time using specialized equipment.
- Immediate response: If anything changes, the anesthesia team can quickly adjust medications, breathing support, or other interventions.
- Recovery oversight: After surgery, your child is watched in a recovery area until they are stable and alert.

This constant supervision is a standard of care in hospitals and surgical centers to ensure children remain safe under anesthesia.
Would you like me to walk you through what parents usually see before and after anesthesia so

you know what to expect?

Q22.Can I stay with my child during the anesthesia process?

Answer:
Short Answer: In many hospitals, parents *may* be allowed to stay with their child during the induction of anesthesia, but it depends on the anesthesiologist's decision, hospital policy, and the child's specific situation.

Parental Presence During Anesthesia Induction
When It's Allowed

- **Purpose:** Parental presence is offered to reduce a child's anxiety and fear before surgery.
- **Process:** A parent may accompany the child into the operating room until the child falls asleep from anesthesia.
- **Age Limits:** Some hospitals allow parental presence only for children above a certain age (e.g., 12 months or older).
- **Number of Parents:** Usually, only **one parent or guardian** is permitted in the operating room.
- **Approval Needed:** The anesthesiologist and surgical team must agree that parental presence is appropriate.

When It's Not Allowed

- **Medical or Safety Concerns:** If the child's condition or the procedure requires strict sterility or rapid intervention, parents may not be permitted.
- **Parental Anxiety:** If a parent is visibly nervous or upset, their presence can make induction harder for the child.
- **Hospital Policy:** Some hospitals restrict parental presence to certain procedures or facilities.

What to Expect If You're Allowed

- You'll be asked to follow staff instructions carefully.
- You may need to wear protective clothing or stay in a designated area.
- Your role is to comfort your child calmly—holding their hand, speaking softly, or helping them feel safe.
- Once your child is asleep, you'll be escorted out of the operating room.

Comparison of Policies

Factor	Allowed Scenario	Restricted Scenario
**Child's Age**	≥ 12 months	Infants < 12 months
**Parent's Role**	Calm, supportive	Anxious, disruptive
**Hospital Policy**	PPI program in place	No parental presence policy
**Medical Situation**	Routine surgery	Emergency/complex cases

**Number of Parents**	One parent	More than one not allowed

Bottom Line: You *might* be able to stay with your child during anesthesia induction, but it's not guaranteed. The anesthesiologist and hospital staff will decide based on safety, policy, and your child's needs.

Would you like me to share **tips on how to prepare your child emotionally** for anesthesia, whether or not you're allowed to be present?

References (5)

1*Parental Presence at Induction of Anesthesia | Children's Pittsburgh*. https://www.chp.edu/our-services/anesthesiology/patient-procedures/parental-presence

2*Parental Presence for Induction of Anaesthesia (PPIA)*. https://www31.ha.org.hk/hkch/share/doc/dept_anaes/Parental Presence for Induction of Anaesthesia_Eng.pdf

3*Parental Presence During Anesthesia Inductions - Child Life On Call*. https://childlifeoncall.com/blog/ppi/

4*Parental Presence for Anesthesia Induction - MUSC Health*. https://children.muschealth.org/medical-services/anesthesia/parental-presence

5*Parental Presence in the Operating Room | WakeMed*. https://www.wakemed.org/wakemed-physician-practices/specialties/pediatric-surgery/education-for-parents-and-families/parental-presence-in-the-operating-room

Q23.How the anesthesia will be given to my child?

Answer:

When a child needs anesthesia, doctors use methods that are safe and tailored to their age, health, and the type of procedure. Here's a clear overview of how it's usually given:

Common Ways Anesthesia Is Given to Children

- Breathing mask: For many children, anesthesia starts with a soft mask placed over the nose and mouth. They breathe in a special gas that helps them fall asleep gently.
- IV (intravenous) line: Sometimes, anesthesia medicine is given through a small needle in the arm or hand. This is more common for older children or when deeper anesthesia is needed.
- Oral or nasal medicine: In certain cases, a liquid or spray medicine may be used first to help the child relax before the mask or IV is placed.
- Regional anesthesia: For some surgeries, doctors may numb only part of the body with an injection, often combined with sedation so the child stays calm.

What to Expect

- A pediatric anesthesiologist (a doctor trained in children's anesthesia) will decide the safest method.
- Parents are often allowed to stay with their child until they are asleep, especially if a mask is used.
- Monitoring equipment (like heart rate and oxygen sensors) will be used throughout to keep your child safe.

Every child's situation is unique, so the exact approach depends on their age, comfort level, and the procedure.

Would you like me to explain how doctors decide between mask and IV anesthesia or what happens during recovery after anesthesia?

Q24.Will my child need help breathing during anesthesia?

Answer:
That's a very important question. In general, anesthesia can affect breathing, and whether a child needs help depends on the type of anesthesia being used:

- **General anesthesia**: This usually makes a person fully unconscious. Because it relaxes the muscles (including those that help with breathing), anesthesiologists often provide breathing support. This may involve a breathing tube, mask, or ventilator to ensure oxygen and airflow are maintained safely.
- **Sedation or regional anesthesia**: If only part of the body is numbed and the child is not fully unconscious, they usually continue breathing on their own. The anesthesiologist still monitors breathing closely and may give oxygen through a mask or nasal cannula if needed.
- **Monitoring**: Regardless of the type, anesthesiologists continuously monitor oxygen levels, heart rate, and breathing throughout the procedure. Their role is to anticipate and manage any changes immediately.

So, in short: **yes, children often need some level of breathing support during general anesthesia**, but the exact approach depends on the procedure and the anesthetic plan. The anesthesiologist will tailor this to your child's age, health, and the surgery being done.
Would you like me to break down the **different breathing support methods** (like masks vs. tubes) so you know what each involves?

Q25.Will my child be sedated before getting anesthesia?

Answer:
That's a thoughtful question. In most cases, children are **not sedated before anesthesia**—instead, anesthesia itself is what provides sedation and unconsciousness. However, there are some situations where doctors may give a child a **premedication** (often a mild sedative) before anesthesia to help them feel calm, reduce anxiety, or make separation from parents easier.
Here's how it usually works in pediatric anesthesia:

- **Comfort first**: Many hospitals allow children to bring a toy, blanket, or even use distraction techniques (like videos or games) to reduce anxiety before anesthesia.
- **Premedication (optional)**: If a child is very anxious, a small dose of oral or intranasal sedative (like midazolam) may be given before anesthesia. This is not routine for every child—it depends on age, temperament, and medical needs.
- **Induction methods**: Children often receive anesthesia through a mask with flavored gas (like "bubblegum air"), so they don't need an IV right away. In some cases, an IV is placed first, and anesthesia medicine is given directly.
- **Safety priority**: The anesthesiologist decides whether pre-sedation is appropriate, balancing comfort with safety.

So, your child won't automatically be sedated before anesthesia, but if anxiety is high, the care team may use gentle medication or calming strategies to make the process smoother.
Would you like me to walk you through **what the anesthesia process looks like step by step for children**, so you know exactly what to expect on the day?

Q26.How soon will my child wake up after anesthesia?

Answer:
Most children begin waking up within minutes after anesthesia, but full alertness can take 30–60 minutes. They may be groggy, confused, or irritable at first, and recovery speed depends on the type of anesthesia, the procedure, and your child's individual response.

What to Expect After Anesthesia

- **Initial recovery**: Children who receive *general anesthesia* are taken to a post-anesthesia care unit (PACU). They usually start to wake up within minutes, but remain sleepy for about an hour.
- **Common reactions**: Confusion, restlessness, or not recognizing parents can occur for 5–15 minutes. Some children may feel nauseated or vomit.
- **Physical effects**: A dry throat (from the breathing tube), irritability, or mild soreness are typical.
- **Discharge timing**: Depending on the procedure, children may go home within a few hours, or stay overnight if monitoring is needed.
- **Parental presence**: Parents are usually allowed in the PACU to comfort their child during recovery.

⏱ Recovery Timeline Comparison

**Type of Anesthesia**	**Wake-up Time**	**Typical Recovery Signs**
**General anesthesia**	Minutes to 1 hour	Sleepiness, confusion, nausea, sore throat
**Regional anesthesia**	Immediate awareness, numbness fades over hours	Tingling or discomfort as sensation returns
**Local anesthesia**	Awake throughout	Only the numbed area feels different

Key Takeaways

- **Children usually wake up quickly** after anesthesia, but may not be fully alert for up to an hour.
- **Temporary side effects** like confusion, nausea, or irritability are common but short-lived.
- **Recovery speed varies** depending on anesthesia type, procedure length, and your child's health.
- **Medical staff monitor closely** to ensure safety before discharge.

Would you like me to outline **tips for helping your child feel comfortable during recovery** or explain **how anesthesia differs for younger vs. older children**?

References (2)

1*What to Expect with Anesthesia | Nemours KidsHealth*.
https://kidshealth.org/en/parents/anesthesia.html
2*Pediatric General Anesthesia: What to Expect*.
https://www.massgeneral.org/children/anesthesia/pediatric-general-anesthesia

Q27.When can I see my child after anesthesia and surgery?

Answer:
After your child's surgery, you'll usually be able to see them once they are safely in the recovery area (often called the Post-Anesthesia Care Unit, or PACU). Here's what typically happens:

Timing

- **Immediately after surgery**: Children are transferred to the PACU where nurses and anesthesiologists closely monitor them as they wake up.
- **First 15–30 minutes**: Staff often focus on stabilizing vital signs, ensuring breathing is steady, and managing pain or nausea.
- **When stable**: Parents are usually allowed in as soon as the child is awake enough and medically stable. This can vary depending on hospital policy, the type of surgery, and how your child responds to anesthesia.

Hospital Policies

- Some hospitals allow parents to join their child in the PACU right away.
- Others may ask you to wait until the child is more alert to reduce stress and allow staff to complete initial assessments.
- For outpatient procedures, you'll often be reunited fairly quickly—sometimes within 30 minutes.

What to Expect

- Your child may be groggy, confused, or upset when they first wake up.
- They may have monitors attached (heart rate, oxygen, blood pressure) and sometimes an IV line.
- Nurses will guide you on how best to comfort your child and what to expect during recovery.

The exact timing depends on the hospital's rules and your child's condition. If you want clarity, the best step is to ask your child's surgical team or the recovery room staff beforehand—they can give you a precise expectation for that facility.
Would you like me to outline **tips for comforting your child when you first see them in recovery**? That way you'll feel prepared for those first moments together.

Q28.Will my child have pain after anesthesia?

Answer:
It's a very thoughtful question. After anesthesia, children don't usually feel pain while they are still under its effects, but once the anesthesia wears off, they may experience discomfort depending on the type of surgery or procedure performed.
Here are some general points:

- **During anesthesia**: Your child will not feel pain because the medicine blocks sensation.
- **After waking up**: Some children may feel groggy, confused, or nauseated, but pain itself depends on the surgery.
- **Post-procedure pain**: If the procedure is minor (like ear tubes), pain is often mild. For more invasive surgeries, pain can be moderate to significant.
- **Pain management**: Doctors usually plan ahead with pain relief strategies—this may include medications given during or after surgery to keep your child comfortable.
- **Emotional factors**: Children may cry or seem distressed after anesthesia, but this can be due to confusion or fear rather than pain.

Since every child and procedure is different, the best source of guidance is your child's anesthesiologist or surgeon. They can explain what level of discomfort to expect and how they'll manage it.
Would you like me to share some common comfort strategies parents use to help children feel better after anesthesia?

Q29.Will my child feel sick or throw up after anesthesia?

Answer:
Yes, nausea and vomiting are among the most common side effects children may experience after anesthesia. The likelihood depends on factors such as the type of anesthesia, the surgery performed, and your child's individual risk profile. Doctors often take steps to reduce this risk with medications and tailored anesthetic plans.

Key Points About Nausea and Vomiting After Anesthesia in Children

- **Common side effect**: Postoperative nausea and vomiting (PONV) are frequent in children, sometimes occurring at rates *twice as high* as in adults.
- **Risk factors**: Higher risk is seen in children over age 3, those with a history of motion sickness or migraines, females (post-menarchal), and kids undergoing certain surgeries like tonsillectomy, strabismus, or abdominal procedures.
- **Medication influence**: Pain medicines, especially opioids, can increase nausea and vomiting.
- **Preventive strategies**: Anesthesiologists often give anti-nausea medications (like ondansetron or dexamethasone) before or during surgery. Regional anesthesia, when possible, also reduces nausea risk.
- **Other side effects**: Besides nausea, children may feel drowsy, dizzy, or "loopy," and may experience sore throat or muscle aches.
- **Serious complications are rare**: While vomiting is unpleasant, more severe issues (like aspiration or airway problems) are uncommon and closely monitored.

Comparison Table: Nausea Risk Factors vs. Prevention

Factor	Effect on Risk	Prevention Strategy
**Age ≥ 3 years**	Higher risk of PONV	Anti-nausea medication, IV fluids
**History of motion sickness**	Strong predictor	Ondansetron, dexamethasone
**Type of surgery**	Tonsillectomy, strabismus, abdominal → higher risk	Dual anti-nausea therapy
**Opioid pain meds**	Increase nausea/vomiting	Use alternatives (acetaminophen, ibuprofen)
**Regional anesthesia**	Lower risk	Nerve blocks, epidurals

What Parents Can Do

- **Discuss concerns with the anesthesiologist**: They can tailor medications to reduce nausea.
- **Follow fasting instructions carefully**: Proper pre-surgery fasting lowers aspiration risk.
- **Monitor at home**: If vomiting persists or your child seems dehydrated, contact the

surgeon.

In short: It's quite possible your child may feel sick or vomit after anesthesia, but anesthesiologists anticipate this and use proven strategies to minimize it.

Would you like me to outline the **specific anti- nausea medications commonly used in children** or the **non- drug approaches like acupressure and hydration** that can help?

Sources: Hospital for Special Surgery – Effects of Anesthesia on Children Pediatric Anesthesia Society – Managing PONV International Journal of Science and Research Archive – Anesthesiology Complications in Children

References (3)

1*How Does Anesthesia Affect Children? - HSS*. https://www.hss.edu/health-library/conditions-and-treatments/effects-of-anesthesia-on-children

2*Managing PONV in Infants and Children - pedsanesthesia.org*. https://www.pedsanesthesia.org/wp-content/uploads/2020/04/Managing-Post-Operative-Nausea-and-Vomiting-in-Infants-and-Children.pptx

3*Anesthesiology complications in children*. https://ijsra.net/sites/default/files/IJSRA-2024-1179.pdf

Q30.If my child had spinal or epidural anesthesia, could they get a headache afterward?

Answer:

Yes — children can develop a headache after spinal or epidural anesthesia. This is called a *post-dural puncture headache (PDPH)*, and while it's more commonly discussed in adults, it can also occur in children.

What is a Post-Dural Puncture Headache?

- **Cause:** It happens when the needle punctures the dura (the membrane around the spinal fluid), allowing cerebrospinal fluid (CSF) to leak. This lowers CSF pressure, leading to headache.
- **Typical features:**
  - Pain at the front or back of the head
  - Worse when sitting or standing, better when lying down
  - May be accompanied by neck stiffness, nausea, sensitivity to light, or hearing changes

Children and PDPH

- **Incidence:** PDPH is less common in children than in adults, but it does occur. Risk increases with larger needles, multiple punctures, or accidental dural puncture during epidural placement.
- **Symptoms in kids:** Children may describe it as a "bad headache" that improves when lying flat. Younger children might show irritability, reluctance to sit up, or sensitivity to light.
- **Duration:** Headaches usually appear within 24–48 hours after the procedure and can last days if untreated.

Risks and Management

- **Conservative treatment:** Bed rest, hydration, caffeine, and pain relief (acetaminophen or ibuprofen, as advised by a doctor).
- **Medical treatment:** If severe or persistent, an *epidural blood patch* may be considered

— this involves injecting a small amount of the child's blood into the epidural space to seal the leak.

- **Prevention:** Using smaller needles and careful technique reduces risk.

Key Takeaway

While not every child will experience a headache after spinal or epidural anesthesia, **post-dural puncture headache is a recognized complication**. It's usually positional, can be distressing, but is treatable. If your child develops a severe or persistent headache after anesthesia, it's important to contact their healthcare team promptly.

Would you like me to walk you through **warning signs that mean you should seek medical help right away** (e.g., when a headache is more than just uncomfortable)? That way you'll know what's urgent versus what can be managed at home.

Sources:

References (5)

1*Anaesthetics – risks and side effects Headache after a spinal or ...*. https://www.rcoa.ac.uk/sites/default/files/documents/2025-11/Risk-HeadacheSpEp2025.pdf
2*Post dural puncture headache - UpToDate*. https://www.uptodate.com/contents/113316
3*Post-dural puncture headache in children: Etiology and treatment - SPA*. https://www2.pedsanesthesia.org/lecture_series/documents/Post-dural-Puncture-Headache-in-Pediatric-Anesthesia.pptx
4*Post-Spinal Anesthesia Headache Prevention and Management: A Brief ...*. https://www.cmjpublishers.com/wp-content/uploads/2025/04/post-spinal-anesthesia-headache-prevention-and-management-a-brief-literature-review.pdf
5*Consensus Practice Guidelines on Postdural Puncture Headache*. https://jamanetwork.com/journals/jamanetworkopen/fullarticle/2808365

Q31. How soon can my child eat and drink after waking up from general anesthesia?

Answer:

Quick Answer: Most children can start drinking clear fluids soon after waking from general anesthesia, once they are fully awake and able to swallow safely. If they tolerate fluids without nausea or vomiting, small amounts of food can follow. Appetite may be reduced for the first 24 hours, so fluids are often the priority.

Eating and Drinking After Anesthesia

- **Start with clear fluids:** Once your child is awake and alert, offer small sips of water, apple juice, or an electrolyte drink (like Pedialyte). This helps prevent dehydration and is easier on the stomach.
- **Progress gradually:** If fluids are tolerated without vomiting or stomach upset, you can introduce light foods such as toast, crackers, or soup. Avoid heavy, greasy, or spicy foods initially.
- **Appetite may be low:** Many children don't feel hungry right away. Fluids may be enough for the first several hours, and appetite usually returns within 24 hours.
- **Watch for nausea/vomiting:** If your child vomits repeatedly or has stomach upset lasting more than 6 hours, contact your healthcare provider.
- **Safety first:** Because anesthesia can temporarily affect swallowing and gag reflexes, eating or drinking too soon can increase the risk of choking or aspiration. Always wait

until your child is fully awake and responsive.

Risks and What to Monitor

- **Nausea and vomiting** are common after anesthesia. Start slowly with fluids to minimize this risk.
- **Aspiration risk:** If food or drink is given before swallowing reflexes return, it can enter the lungs. That's why waiting until your child is fully awake is critical.
- **Hydration:** Encourage fluids even if appetite is low. Dehydration can slow recovery.
- **Emergency signs:** Persistent vomiting, severe abdominal pain, or inability to keep fluids down should prompt a call to your child's doctor or a visit to the emergency department.

Practical Tips for Parents

- Offer **small, frequent sips** rather than large amounts at once.
- Stick to **bland, easy-to-digest foods** for the first meal.
- Keep your child resting quietly for the first 24 hours; activity can worsen nausea.
- Monitor closely for the first hour at home, then check in regularly throughout the day.

Summary: Your child can usually drink clear fluids soon after waking from anesthesia, once they are alert and able to swallow. If tolerated, light foods can follow, but appetite may be limited for the first day. Always watch for nausea or vomiting, and contact a healthcare provider if symptoms persist.

Sources:

Would you like me to create a **step-by-step recovery checklist** for parents so you have a clear guide to follow at home?

References (5)

1*Post-operative and post-anesthetic care: Caring for your child at home*. https://www.aboutkidshealth.ca/post-operative-and-post-anesthetic-care-caring-for-your-child-at-home

2*Information for children having had general anaesthesia*. https://www.cuh.nhs.uk/patient-information/information-for-children-having-had-general-anaesthesia/

3*Post-Operative Care Following a General Anesthesia sleepy and drowsy ...*. https://www.dentalanesthesiadds.com/uploads/b/29c871d0-bc63-11ee-880b-397610e8bb8d/Post Anesthesia InstructionsEnglish_Njc4MD.pdf

4*Eating and Drinking After Anesthesia*. https://www.anesthesiaservicesar.com/eating-and-drinking-after-anesthesia/

5*After anesthesia – caring for your child - Children's Healthcare of ...*. https://www.choa.org/-/media/Files/Childrens/teaching-sheets/after-anesthesia-care---child.pdf

Q32.How long will my child need to stay at the hospital after anesthesia?

Answer:

Quick Answer: Most children only stay at the hospital for a few hours after anesthesia, but the exact time depends on the type of procedure, how your child recovers, and whether the surgery requires overnight monitoring.

Typical Recovery Timeline After Anesthesia

- **Immediate Recovery (PACU):** After surgery, your child will go to the **post-anesthesia care unit (PACU)**. Here, nurses monitor breathing, heart rate, and alertness until the anesthesia wears off.

- **Short Stay / Outpatient Procedures:** If the surgery is minor (like ear tubes or dental work), your child may be discharged the **same day** once they are awake, stable, and able to drink fluids.
- **Inpatient Stay:** For more complex surgeries, children may be admitted to a hospital unit for **overnight or longer recovery**.

How Long Before Going Home?

- **Few Hours:** Many children are ready to leave within **2–4 hours** after anesthesia if the procedure is simple and there are no complications.
- **24 Hours of Caution:** Even after going home, children may feel **sleepy, dizzy, or irritable** for up to a day. Parents are advised to keep activity low and watch closely.
- **Longer Stays:** If the surgery is major (heart, abdominal, orthopedic), or if your child has underlying health issues, the hospital stay could be **overnight to several days**.

Risks & What to Watch For

- **Common Side Effects:** Sleepiness, nausea, sore throat, or restlessness.
- **When to Seek Help:** Difficulty breathing, persistent vomiting, unusual sleepiness (hard to wake), or severe pain should be reported immediately.
- **Diet & Activity:** Start with clear liquids, then gradually return to normal foods. Avoid strenuous activity for at least 24 hours.

Key Takeaway

Your child's hospital stay after anesthesia is usually **short (a few hours)** for minor procedures, but **overnight or longer** if the surgery is complex. Even after discharge, close monitoring at home for the first 24 hours is essential.

Would you like me to break down **what signs show your child is ready to go home** versus when doctors recommend staying longer? That way you'll know exactly what to expect.

Sources: Children's Hospital of Philadelphia – Recovery After Surgery UC Davis Children's Hospital – Anesthesia After Care Children's Healthcare of Atlanta – After Anesthesia Care

References (3)

1*Your Child's Recovery After Surgery | Children's Hospital of Philadelphia*. https://www.chop.edu/patients-and-visitors/guide-your-childs-surgery/your-childs-recovery-after-surgery

2*Anesthesia after care | Patient Education | UC Davis Children Hospital*. https://health.ucdavis.edu/children/patient-education/Anesthesia-after-care

3*After anesthesia – caring for your child - Children's Healthcare of …*. https://www.choa.org/-/media/Files/Childrens/teaching-sheets/after-anesthesia-care---child.pdf

Q33.Who can I contact if I have more questions after my child's anesthesia?

Answer:

After your child has anesthesia, it's completely normal to have follow-up questions or concerns. Here are the best points of contact:

Who to Reach Out To

- **Your child's anesthesiologist** – They are the most knowledgeable about the anesthesia given and can answer specific questions about side effects, recovery, or risks.
- **Your child's surgeon or procedure doctor** – If the questions relate to the surgery itself or how anesthesia interacts with healing.

- **Pediatrician or family doctor** – For general health concerns, monitoring recovery, or if you're unsure whether a symptom is related to anesthesia.
- **Hospital or surgical center's recovery team** – Most facilities provide a discharge phone number for post-anesthesia questions.
- **Emergency services (911 in the U.S.)** – If your child has trouble breathing, persistent vomiting, severe pain, or anything that feels urgent.

Practical Tip

When your child is discharged, you should receive written instructions with a **24-hour contact number**. Keep that paper handy—it's the fastest way to reach the right medical team.

Would you like me to share a checklist of **common symptoms after anesthesia** that are usually normal versus ones that mean you should call a doctor right away?
