# Supplementary figures and images for: Evaluation of Five Large Language Models for Parental Education in Pediatric Anesthesia: Reliability and Readability Study
Source: JMIR Med Inform. 2026 Jun 18;14:e93054. doi: 10.2196/93054 (PMC13278617; doi:10.2196/93054)

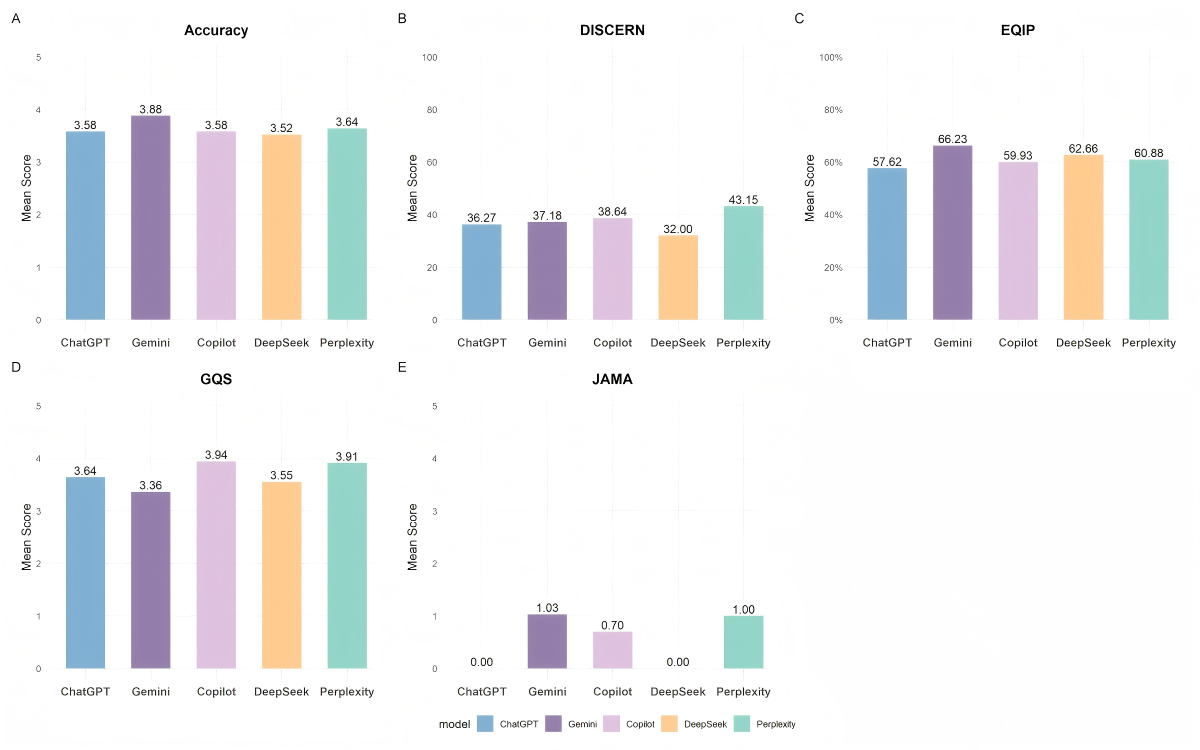

Supplement: Multimedia Appendix 3 [file medinform-v14-e93054-s003.png]
